# Supplementary figures and images for: Repeat mediated excision of gene drive elements for restoring wild-type populations
Source: PLoS Genet. 2024 Nov 7;20(11):e1011450. doi: 10.1371/journal.pgen.1011450 (PMC11584131; doi:10.1371/journal.pgen.1011450)

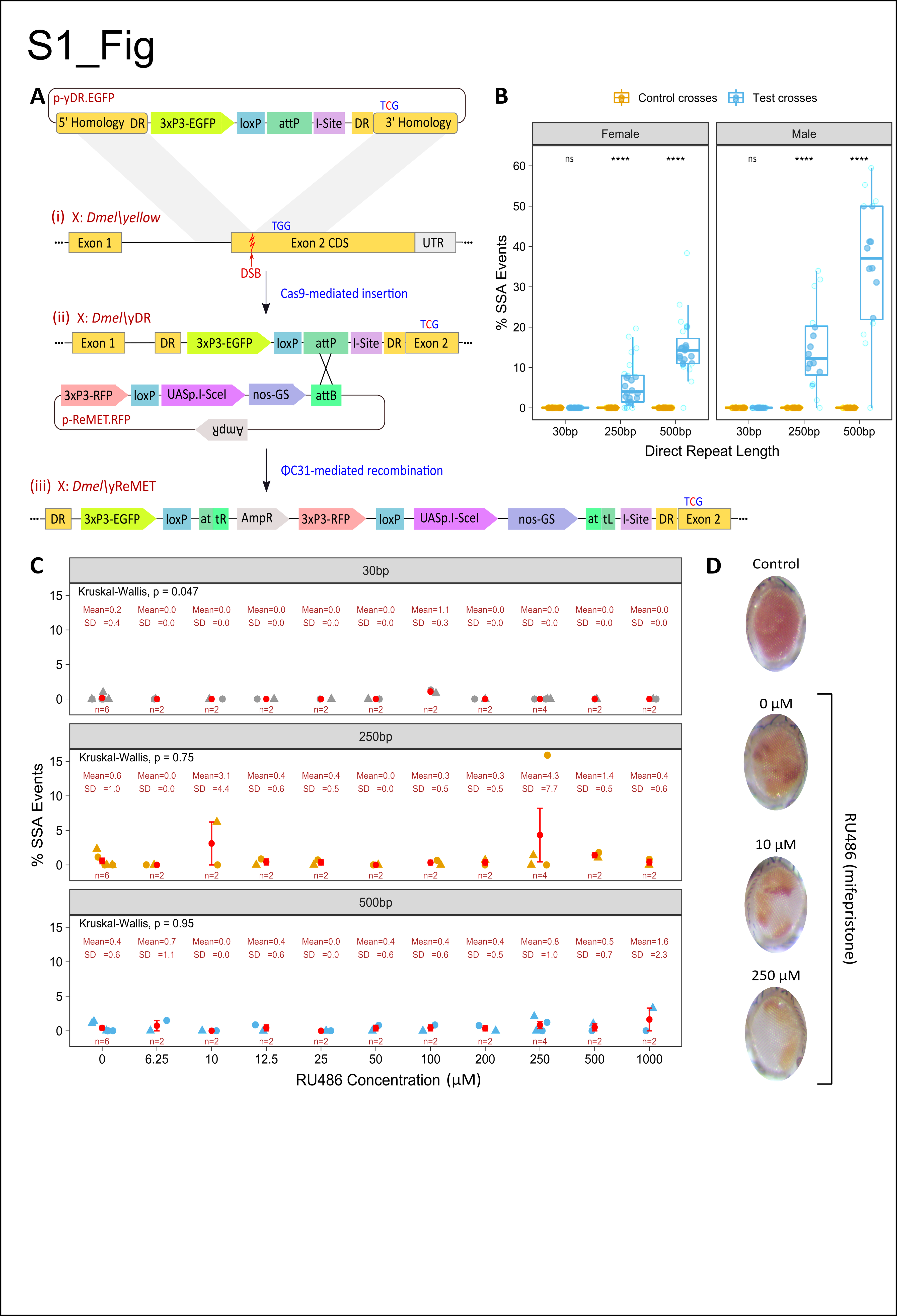

Supplement: S1 Fig — (A) Schematic depicting the two-step process for the generation of yReMET (in cis) transgenic lines. In the first step, a donor plasmid, p-yDR.EGFP, with DRs of varying lengths (30, 250 or 500 bp), identical in sequence to the 3’ end of the 5’ homology arm, were inserted into the D. melanogaster yellow gene at a Cas9 target site. The donor construct contained 3xP3-EGFP, which served as a marker of transgenesis, a recognition site for the I-SceI endonuclease (I-Site), a loxP site, and an attP landing site. The resulting yDR transgenic fly lines were also engineered to contain the silent PAM mutation TGG->TcG. In the second step, a donor plasmid, p-ReMET.RFP, containing an attB site was inserted into the yDR lines through ϕC31-mediated recombination. The p-ReMET.RFP donor contained 3xP3-RFP, a loxP site, as well as the nos-GS and UASp.I-SceI components. In another configuration, used for the in trans experiments, only the UASp.I-SceI is included and not the nos-GS. Integration of the p-ReMET.RFP plasmid into the yDR lines generated the various yReMET lines containing DRs of different lengths. (B) Percentage of F2 progeny exhibiting SSA-mediated transgene excision by DR length and sex, confirmed through the presence of wild-type body pigmentation and the engineered silent mutation (TcG). Gold dots represent individual pair-mated crosses of F1 yReMET (in trans configuration) flies with wild-type flies. Blue dots represent individual pair-mated crosses of F1 yReMET (in trans configuration) flies with transgenic nos-Gal4 flies. P-value **** < 0.0001; ns = not significant. (C) Percentage of F1 progeny exhibiting SSA-mediated transgene excision by DR length and RU486 concentration, confirmed through the presence of wild type body pigmentation and the engineered silent mutation (TcG). Each dot/triangle represents a separate pair-mated cross (mean and ± s.e.m. are shown in red). (D) Images of adult eye pigmentation in F1 progeny resulting from pair-mated crosses of yReMET (in [file pgen.1011450.s002.tif]

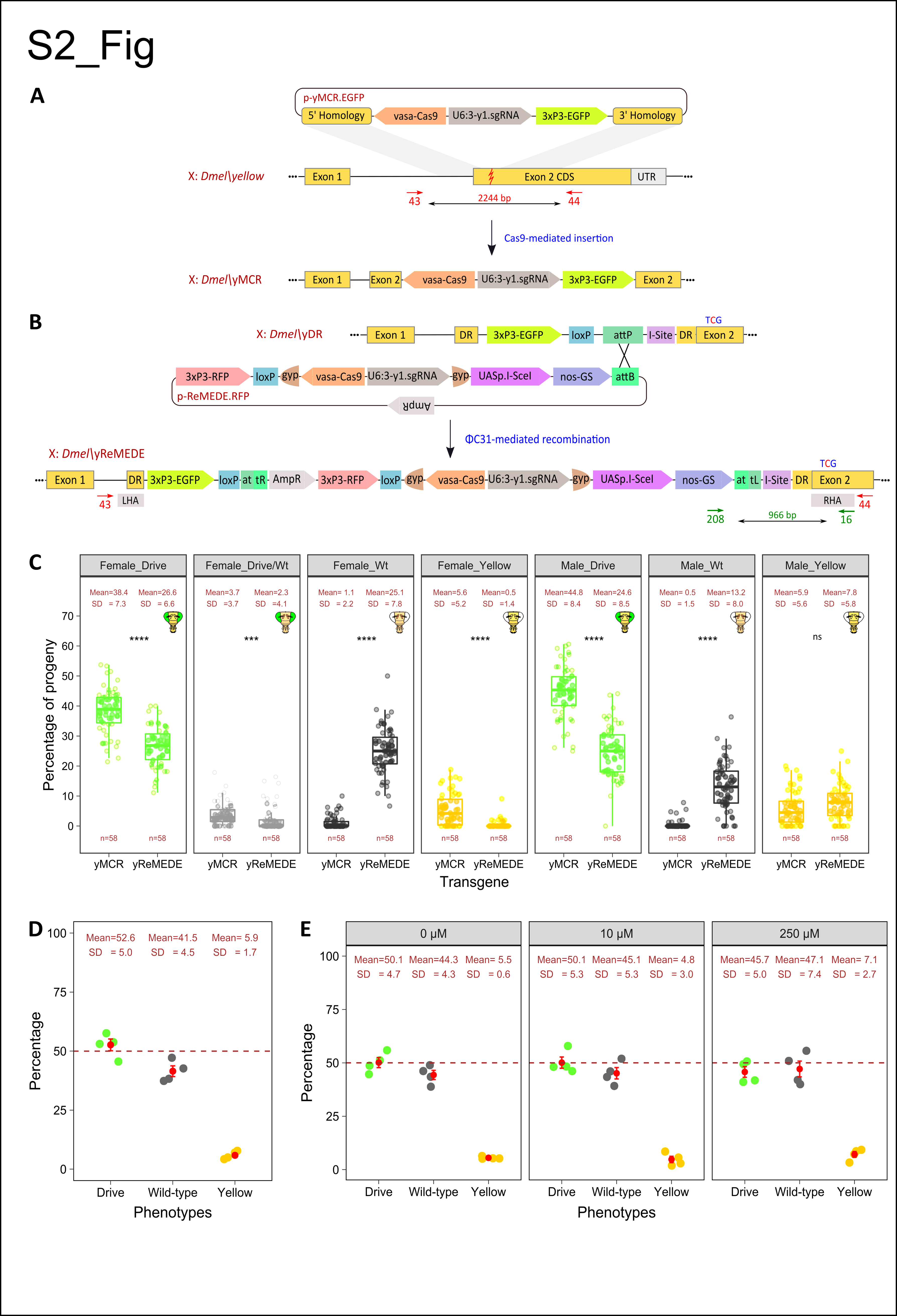

Supplement: S2 Fig — (A) Schematic depicting insertion of p-yMCR.EGFP into the yellow gene of D. melanogaster. The plasmid p-yMCR.EGFP contains Cas9 (under the control of a vasa promoter), an sgRNA targeting exon 2 of the wild-type yellow gene (under the control of a U6:3 promoter), and EGFP (under the control of the eye-specific 3xP3 promoter). The primer annealing sites 43 and 44 are located outside the homology arms in order to permit genotyping of target alleles in flies exhibiting a wild-type phenotype. (B) Schematic depicting the generation of the yReMEDE line through ϕC31-mediated recombination of p-ReMEDE.RFP into the yDR line. The plasmid p-ReMEDE.RFP contains the yMCR drive element flanked by a pair of gypsy insulators (gyp), nos-GS > UASp-I-SceI, a loxP site, and 3xP3-RFP. Primer annealing sites 208 and 16 permit genotyping of the PAM site in the drive allele. (C) Maternal and paternal inheritance of drive elements. Percentage of progeny exhibiting scored phenotypes (i.e., body pigmentation and fluorescent eye color) as indicated by cartoon fly heads. Each dot represents a separate pair-mated cross. (D) Percentage of progeny exhibiting wild-type phenotypes in replicate en masse crosses of yReMEDE flies. Each dot represents a separate en masse cross (mean and ± s.e.m. are shown in red). (E) Percentage of progeny exhibiting wild-type phenotypes in replicate en masse crosses of yReMEDE flies in the presence of RU486 at the concentrations specified. LHA = Left Homology Arm; RHA = Right Homology Arm; p values = ** < 0.01, *** < 0.001, and **** < 0.0001; ns = not significant (Wilcoxon signed-rank test). (TIF) [file pgen.1011450.s003.tif]

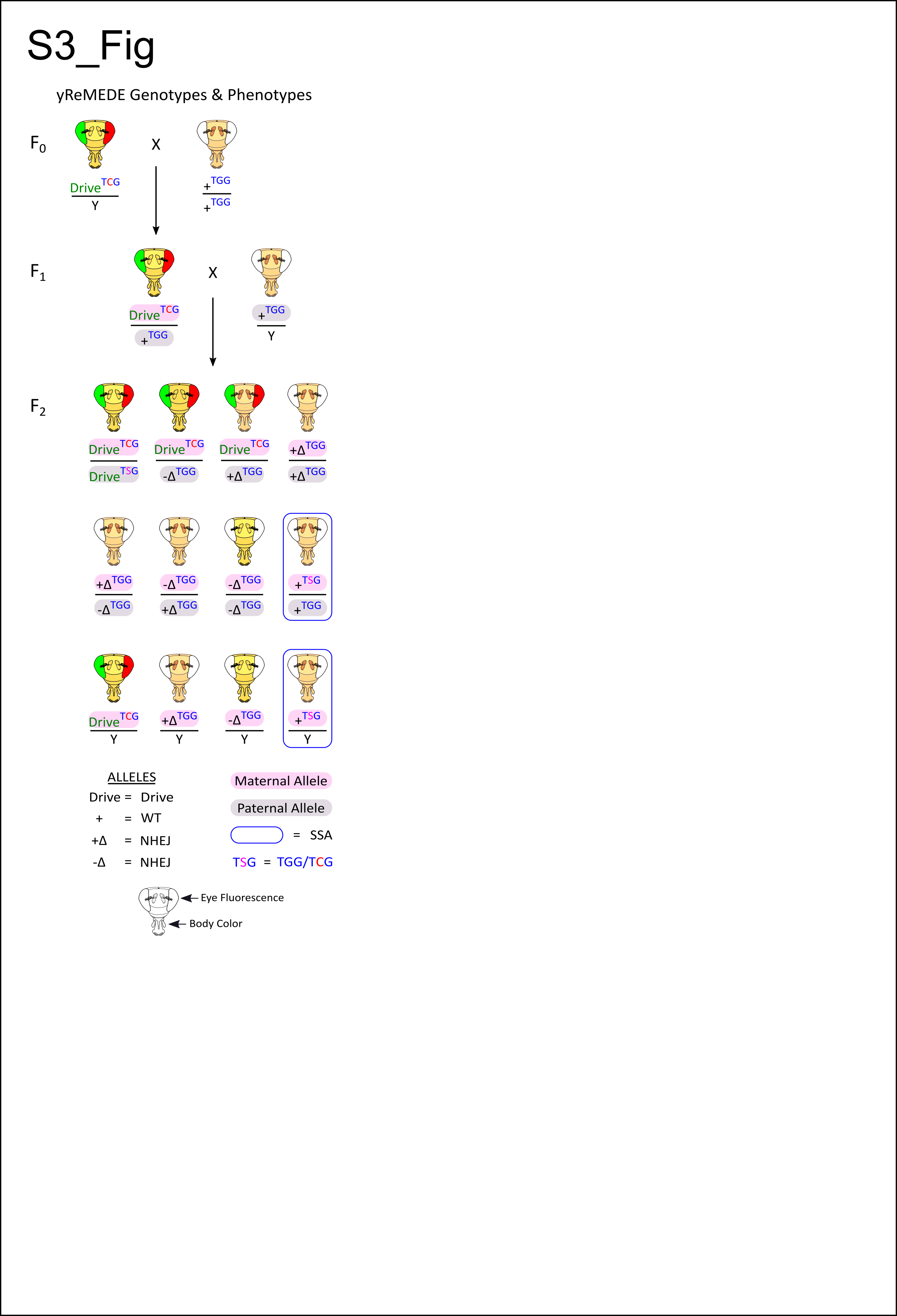

Supplement: S3 Fig — Mating scheme for generation of F1 yReMEDE females and their F2 progeny. Images of fly heads indicate scored phenotypes of eye fluorescence (white = no marker, green = EGFP, red = RFP) and body color (brown = + or +Δ, yellow = Drive or -Δ). Possible allelic combinations are shown below head images (ReMEDE = Drive, wild type = +, in-frame indel = +Δ, out-of-frame indel = -Δ). Male flies are indicated by the presence of the Y chromosome. Blue boxes enclose genotypes and phenotypes that confirm SSA-mediated transgene excision, regardless of the PAM sequence. (TIF) [file pgen.1011450.s004.tif]

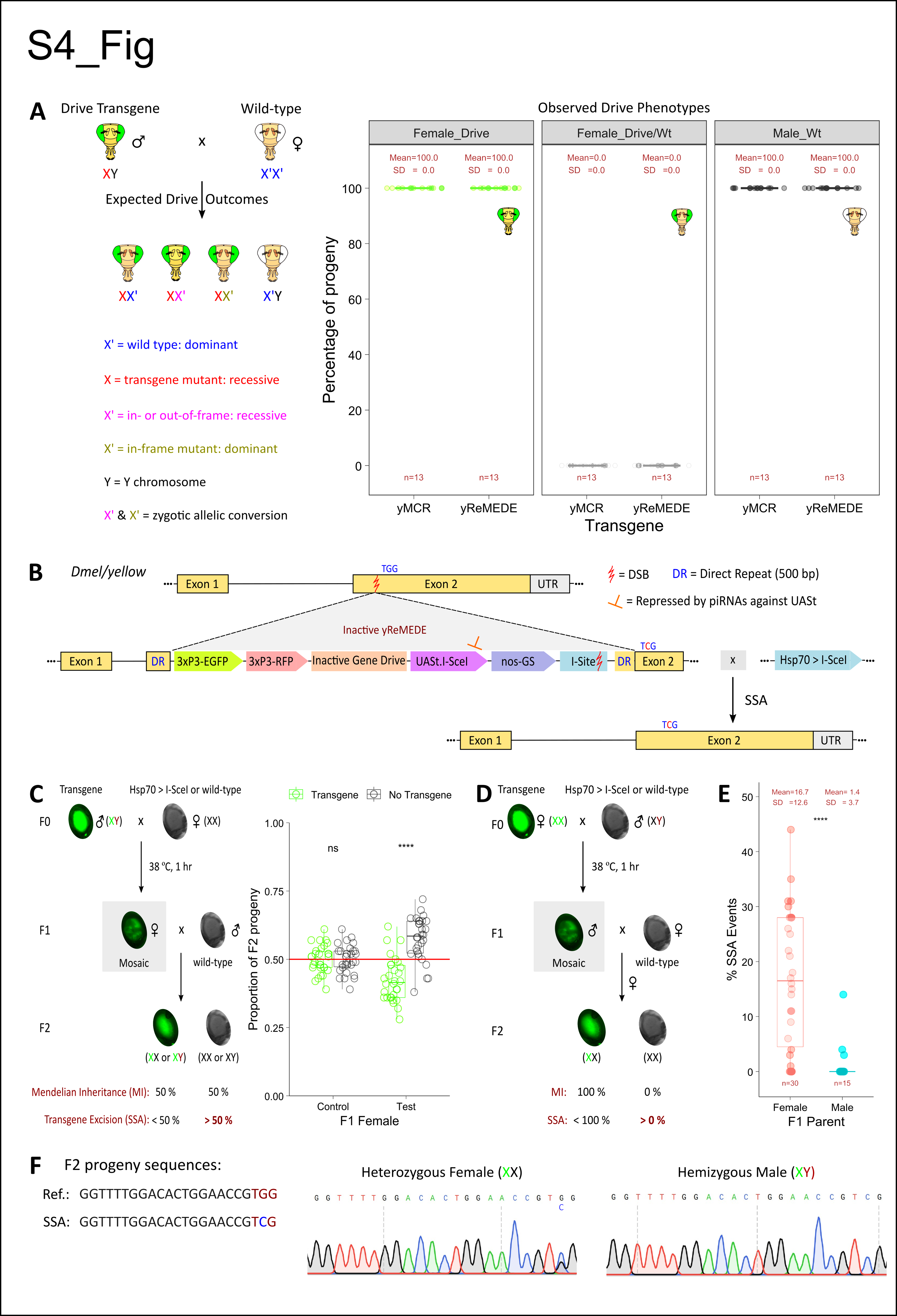

Supplement: S4 Fig — (A) Schematic illustrating the expected genetic outcomes and associated phenotypes resulting from patrilineal inheritance of yReMEDE. (B) Diagram depicting the inactive yReMEDE construct and its removal through SSA after expression of the I-SceI endonuclease (provided in trans) under the control of the Hsp70 promoter. (C) Schematic of the in trans mating scheme and phenotypic identification of germline SSA events. Transgenic F0 males were crossed with either wild-type (control) or Hsp70 > I-SceI (test) females. Embryos were subjected to heat shock at 38°C for 1 hour to induce Hsp70-driven expression of I-SceI. Germline SSA events occurring in F₁ individuals were identified by scoring F₂ offspring for fluorescent markers. Statistical significance was determined using the exact binomial test (P-values: ns, not significant; ****, P < 0.0001). (D) Schematic of the reciprocal cross where transgenic F0 females were crossed with either wild-type (control) or Hsp70 > I-SceI (test) males. (E) Percentage of SSA events observed in maternal or paternal germlines, estimated by comparing the number of fluorescent and non-fluorescent progeny. P-value: **** = P < 0.0001 (Wilcoxon signed-rank test). (F) Representative sequencing data confirming the presence of the engineered TcG-resistant allele in all non-fluorescent progeny from a randomly selected family from either female or male test crosses. (TIF) [file pgen.1011450.s005.tif]

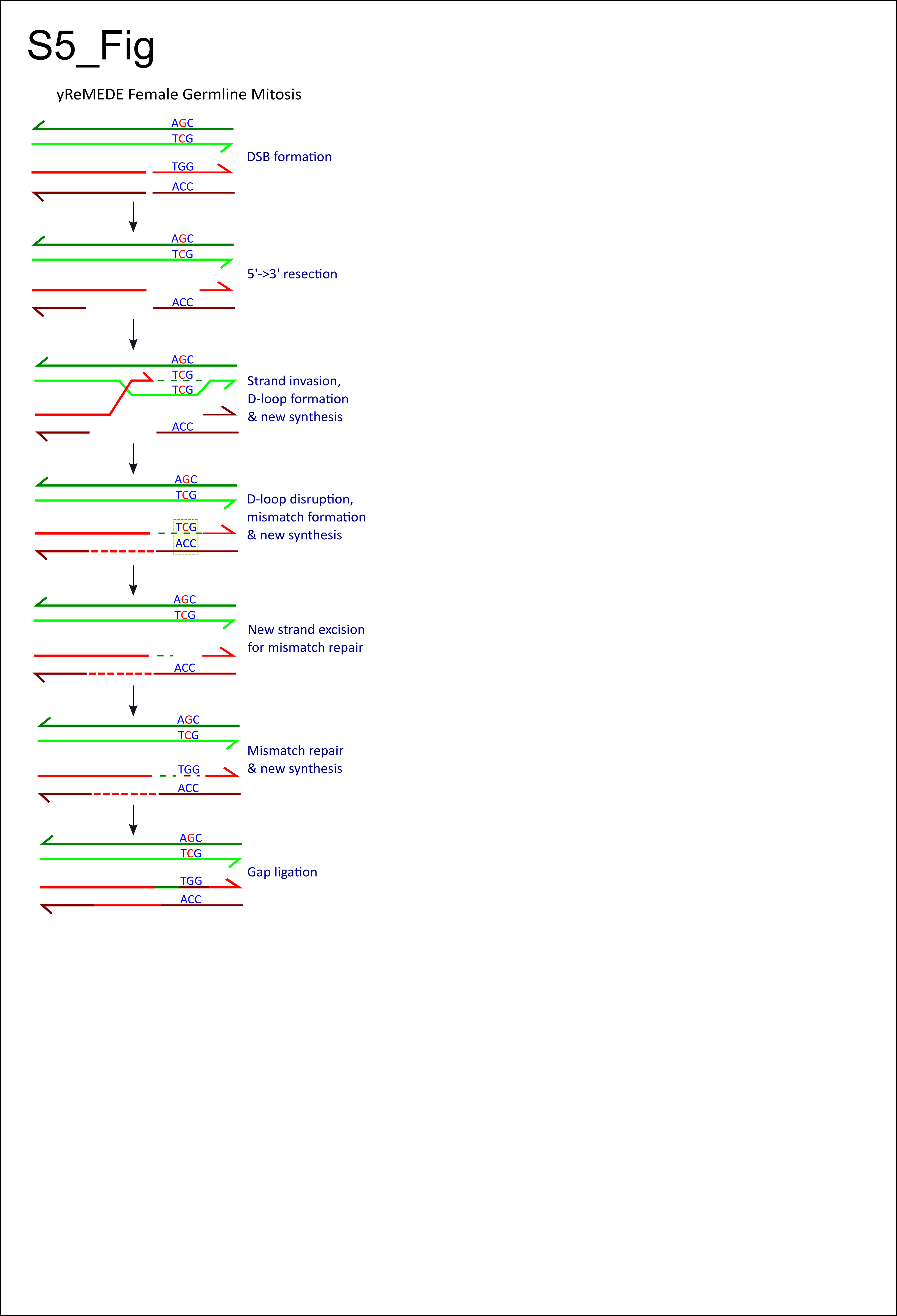

Supplement: S5 Fig — The SDSA model appears to be the primary DSB repair mechanism in mitotic cells and is shown for simplicity. Production of Cas9 RNP from the gene drive (donor) allele (shown in green) generates a DSB in the wild-type (recipient) allele (shown in red). Resection of the 5’ DNA ends produces 3’ single-stranded tails. Strand invasion of the donor drive allele by one of the recipient allele’s 3’ tail results in base pairing of complementary strands generating a tract of heteroduplex DNA (hetDNA). This process displaces the originally duplexed strand forming a displacement (D)-loop. Elongation of the invading 3’ end increases the size of the D-loop, eventually producing a sequence that extends beyond the DSB. Dismantling of the D-loop frees the newly synthesized sequence to anneal with the other 3’ tail of the recipient allele, generating a new tract of hetDNA with the opposite strand, i.e., SDSA. Mismatches present in the hetDNA are repaired by MMR. Excision followed by resynthesis and ligation repairs the mis-paired bases, restoring the original wild-type sequence of the recipient and eliminating heterozygosity at the locus. SDSA = Synthesis Mediated Strand Annealing, DSB = Double Strand Break, RNP = RiboNucleoProtein. (TIF) [file pgen.1011450.s006.tif]

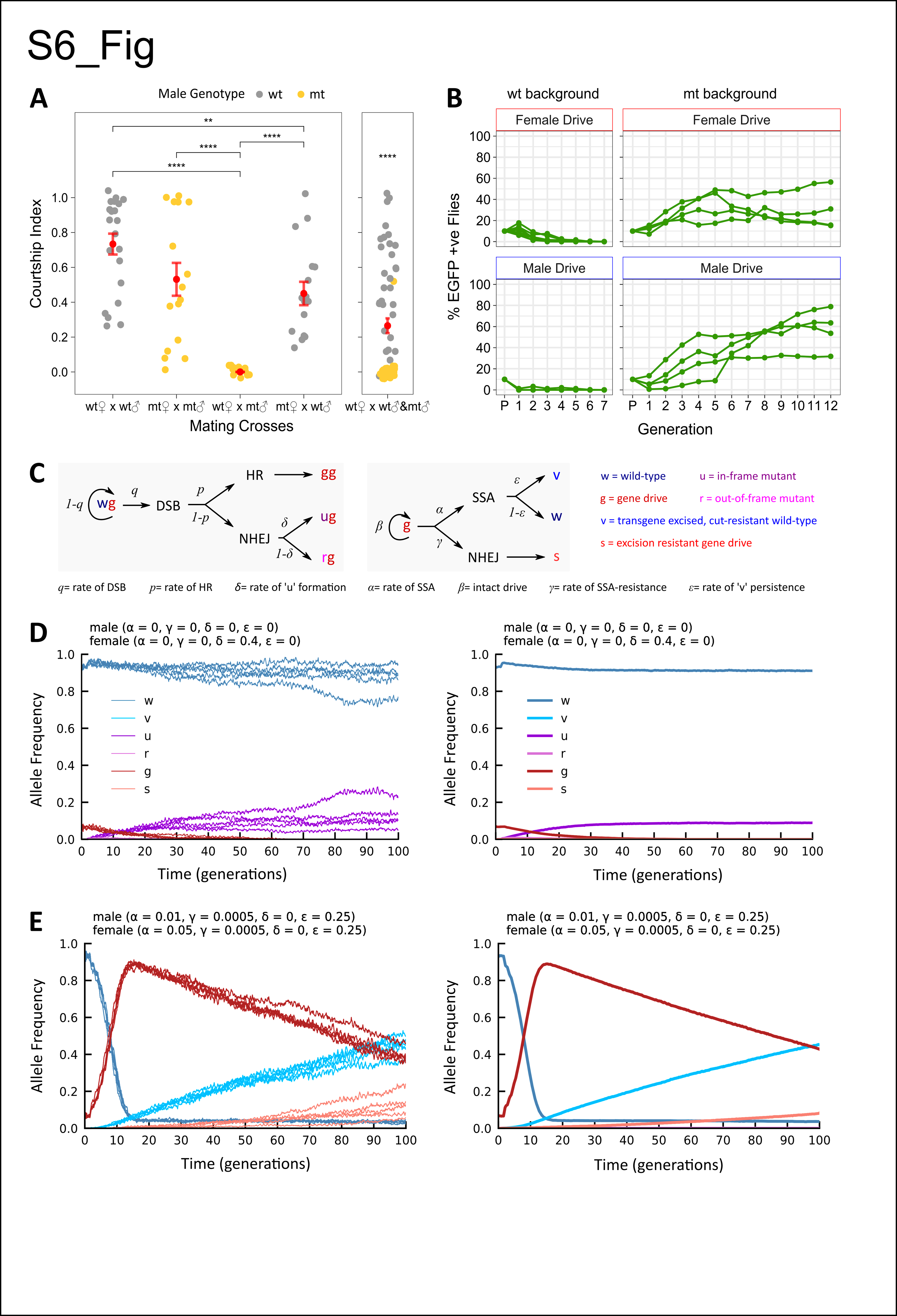

Supplement: S6 Fig — (A) Courtship indices calculated for yellow mutant (mt) and w1118 (wt) flies in the crossing schemes shown. P-values = ** < 0.01, and **** < 0.0001 (Wilcoxon signed-rank test). (B) Cage trials with the yMCR gene drive in either a w1118 (wt) or yellow mutant (mt) population. F1 females or males carrying the yMCR allele initially made up 10% of the total population. Graphs show the prevalence of the drive phenotype (green line) in both male and female progeny over time. Each line represents a separate replicate cage trial. (C) Theoretical model of allelic conversion dynamics occurring within the F1 female germline during oogenesis. In gene drive, a DSB occurs independently at each susceptible target allele (w) with a probability of q, after which repair occurs by HR with a probability of p, or by NHEJ with a probability of 1-p. HR results in allelic conversion of w to g, while NHEJ produces in-frame alleles (u) with a probability of δ, or out-of-frame alleles (r) with a probability of 1-δ. In the presence of ReMEDE, a second DSB occurs and is repaired by SSA with a probability of α, or by NHEJ with a probability of γ. SSA retains the engineered silent PAM mutation (TcG) with a probability of ε, or TcG reverts back to TGG through MMR with a probability of 1-ε. (D) Stochastic modeling of the yMCR gene drive in a wild-type population. All models were run with the X-linked inheritance module that outputs allele frequencies in two plots. One plot shows five simulations randomly selected from 100 independently run simulations (Left), while the other plot shows the mean of all simulations (Right). Simulations of the yMCR gene drive were run in a low threshold release scenario (10% of flies containing a drive allele), and generating resistant alleles at frequencies (δ = 0.4) comparable to those observed experimentally. Similarly, mating cost parameters were set at levels consistent with empirically determined values for yellow male (0.97) and female (0.31) flies in a wild-typ [file pgen.1011450.s007.tif]
