# Supplementary material for: Repeat mediated excision of gene drive elements for restoring wild-type populations
Source: PLoS Genet. 2024 Nov 7;20(11):e1011450. doi: 10.1371/journal.pgen.1011450 (PMC11584131; doi:10.1371/journal.pgen.1011450)
Supplement: S7 Fig — (DOCX) [file pgen.1011450.s008.docx]

S7_Fig

p-yDR(30bp).EGFP

**30 bp DR = GTGGTCGGCTGTGGGTTTTGGACACTGGAA**

TGG = PAM

tcgcgcgtttcggtgatgacggtgaaaacctctgacacatgcagctcccggagacggtcacagcttgtctgtaagcggatgccgggagcagacaagcccgtcagggcgcgtcagcgggtgttggcgggtgtcggggctggcttaactatgcggcatcagagcagattgtactgagagtgcaccatatgcggtgtgaaataccgcacagatgcgtaaggagaaaataccgcatcaggcgccattcgccattcaggctgcgcaactgttgggaagggcgatcggtgcgggcctcttcgctattacgccagctggcgaaagggggatgtgctgcaaggcgattaagttgggtaacgccagggttttcccagtcacgacgttgtaaaacgacggccagtCTCGAGgacgtcGTTGCGAGGTTTTAGGACTGAAAGAGCACATGTCAAAATATAAATTTGTTCAAATACTTTATATTTGACTGAATTAGATTGTTATTTTAAAAGTTATGAATTAAATAAAGATTGAAAGGTGCATTATGCTCAAATGTATATTTATCGCAACCCCCGGTTACTTTGTAAAGCAAAAACGCCTGGTTTGATTTTTAAGAAGATGGGTCGGTAAATCGATAAAAGCTATATTTTCTGGTCGTTGCAGTCTCACTCGCCTGCTATAAAAACATTAAAAGTTCCCAGAAACAATAAATGTCTTTAAATTCAATTAACGAAGAAATAAAGAAGGAAAAGAACTGGAGCGGAAATCGGTCGAAATACTGCCAATGGCCACATATACATTTAACAGCGATATATGGTATACATATTGATAATGATGTCAGACGCAATTGCTTCAGACGGCTAATGACATCGCAAATTGCACGCAACTTGCAATAGTGCCAATTATGACTGAAGTACATATAGCCGGGGATCTTTTAACATAAACTTCCAGTAGATGTACAAGCAGAAAAAAGAGCCATTAGCACGGCAGTTACCATTGCTTATGATTCCTTGTGTCCAAAATAATGACAAATAGGTATATAAATAATTAAATGCCAAACATAAGCGATTCTAATTTACCTTTACATCTGTATGCATTTACATATTATCCAGAAAACAGACAGCGATAACTTGCAACATTGCTTAGTATAATAATCCAAAGAAGGAATTTAGGCAGAAATTCCAGTTAATTAAATATTCAAAACAAACTTTATTTAGTGCCTCAATAATAGTTTGGCCCTGCTAATTCTCCTATTTTATTTTTTAGGGATTCCGGCCACTCTGACCTATATAAACATGGACCGCAGTTTGACGGGTTCACCGGAGCTAATTCCGTATCCAGATTGGCGCTCAAATACAGCTGGAGATTGCGCCAACAGTATTACCACTGCCTACCGCATTAAAGTGGATGAGT**GTGGTCGGCTGTGGGTTTTGGACACTGGAA**GCGGCCGCCCTAGGCGTACGGGATCTAATTCAATTAGAGACTAATTCAATTAGAGCTAATTCAATTAGGATCCAAGCTTATCGATTTCGAACCCTCGACCGCCGGAGTATAAATAGAGGCGCTTCGTCTACGGAGCGACAATTCAATTCAAACAAGCAAAGTGAACACGTCGCTAAGCGAAAGCTAAGCAAATAAACAAGCGCAGCTGAACAAGCTAAACAATCGGGGTACCGCTAGAGTCGACGGTACCGCGGGCCCGGGATCCACCGGTCGCCACCATGGTGAGCAAGGGCGAGGAGCTGTTCACCGGGGTGGTGCCCATCCTGGTCGAGCTGGACGGCGACGTAAACGGCCACAAGTTCAGCGTGTCCGGCGAGGGCGAGGGCGATGCCACCTACGGCAAGCTGACCCTGAAGTTCATCTGCACCACCGGCAAGCTGCCCGTGCCCTGGCCCACCCTCGTGACCACCCTGACCTACGGCGTGCAGTGCTTCAGCCGCTACCCCGACCACATGAAGCAGCACGACTTCTTCAAGTCCGCCATGCCCGAAGGCTACGTCCAGGAGCGCACCATCTTCTTCAAGGACGACGGCAACTACAAGACCCGCGCCGAGGTGAAGTTCGAGGGCGACACCCTGGTGAACCGCATCGAGCTGAAGGGCATCGACTTCAAGGAGGACGGCAACATCCTGGGGCACAAGCTGGAGTACAACTACAACAGCCACAACGTCTATATCATGGCCGACAAGCAGAAGAACGGCATCAAGGTGAACTTCAAGATCCGCCACAACATCGAGGACGGCAGCGTGCAGCTCGCCGACCACTACCAGCAGAACACCCCCATCGGCGACGGCCCCGTGCTGCTGCCCGACAACCACTACCTGAGCACCCAGTCCGCCCTGAGCAAAGACCCCAACGAGAAGCGCGATCACATGGTCCTGCTGGAGTTCGTGACCGCCGCCGGGATCACTCTCGGCATGGACGAGCTGTACAAGTAAAGCGGCCTAAGCGATCGCTCAGGCCGCGACTCTAGATCATAATCAGCCATACCACATTTGTAGAGGTTTTACTTGCTTTAAAAAACCTCCCACACCTCCCCCTGAACCTGAAACATAAAATGAATGCAATTGTTGTTGTTAACTTGTTTATTGCAGCTTATAATGGTTACAAATAAAGCAATAGCATCACAAATTTCACAAATAAAGCATTTTTTTCACTGCATTCTAGTTGTGGTTTGTCCAAACTCATCAATGTATCTTAAAGCTTATCGATACGCGTACGGCGCGCCAAAGCTTTGTCTCGAGTCCGGAATAACTTCGTATAGCATACATTATACGAAGTTATGGTCCCAGGTCAGAAGCGGTTTTCGGGAGTAGTGCCCCAACTGGGGTAACCTTTGAGTTCTCTCAGTTGGGGGCGTAGGGTCGCCGACATGACACAAGGGGTTTGGTCTAGGGATAACAGGGTAATAGCGGCCGGCC**GTGGTCGGCTGTGGGTTTTGGACACTGGAA**CCGTGGGCATCGGCAATACCACCACTAATCCGTGCCCCTATGCGGTAAATGTCTTTGACTTGACCACGGATACGCGAATTCGGAGATACGAGCTACCTGGCGTGGACACAAATCCAAATACTTTCATAGCTAACATTGCCGTGGATATAGGCAAAAATTGCGATGATGCATATGCCTATTTTGCCGATGAATTGGGATACGGCTTGATTGCTTACTCCTGGGAACTGAACAAGTCCTGGAGATTCTCGGCACATTCGTATTTTTTCCCCGATCCATTGAGGGGCGATTTCAATGTCGCTGGTATTAACTTCCAATGGGGCGAGGAGGGTATATTTGGTATGTCCCTTTCGCCCATTCGATCGGATGGTTATCGTACCCTGTACTTTAGTCCGTTAGCAAGTCATCGACAATTTGCCGTATCCACGAGGATTTTGAGGGATGAAACCAGGACGGAAGATAGCTATCATGACTTTGTTGCCTTAGATGAACGGGGTCCAAACTCCCATACCACTTCACGTGTGATGAGCGATGATGGAATTGAGCTGTTCAATTTAATAGATCAAAATGCAGTGGGTTGCTGGCACTCATCAATGCCGTACTCACCGCAATTTCATGGCATTGTGGATCGCGATGACGTTGGCTTAGTTTTTCCGGCCGATGTGAAAATTGATGAGAACAAAAACGTTTGGGTTCTATCCGATAGGATGCCCGTTTTCTTGCTGTCTGACTTGGATTATTCAGATACTAATTTCCGAATTTACACGGCTCCCTTGGCCACTTTAATTGAGAATACTGTGTGTGATTTGAGGAATAACGCCTATGGGCCGCCAAATACCGTTTCAATACCAAAACAAGCCGTTTTGCCAATGGGTCCACCGTTATATACGAAACAATATCGTCCTGTCTTGCCACAGAAACCTCAGACCAGCTGGGCTTCCTCGCCGCCTCCTCCAAGTCGCACTTATTTGCCCGCCAATTCAGGCAATGTAGTCTCCAGgctagcggcgtaatcatggtcatagctgtttcctgtgtgaaattgttatccgctcacaattccacacaacatacgagccggaagcataaagtgtaaagcctggggtgcctaatgagtgagctaactcacattaattgcgttgcgctcactgcccgctttccagtcgggaaacctgtcgtgccagctgcattaatgaatcggccaacgcgcggggagaggcggtttgcgtattgggcgctcttccgcttcctcgctcactgactcgctgcgctcggtcgttcggctgcggcgagcggtatcagctcactcaaaggcggtaatacggttatccacagaatcaggggataacgcaggaaagaacatgtgagcaaaaggccagcaaaaggccaggaaccgtaaaaaggccgcgttgctggcgtttttccataggctccgcccccctgacgagcatcacaaaaatcgacgctcaagtcagaggtggcgaaacccgacaggactataaagataccaggcgtttccccctggaagctccctcgtgcgctctcctgttccgaccctgccgcttaccggatacctgtccgcctttctcccttcgggaagcgtggcgctttctcatagctcacgctgtaggtatctcagttcggtgtaggtcgttcgctccaagctgggctgtgtgcacgaaccccccgttcagcccgaccgctgcgccttatccggtaactatcgtcttgagtccaacccggtaagacacgacttatcgccactggcagcagccactggtaacaggattagcagagcgaggtatgtaggcggtgctacagagttcttgaagtggtggcctaactacggctacactagaagaacagtatttggtatctgcgctctgctgaagccagttaccttcggaaaaagagttggtagctcttgatccggcaaacaaaccaccgctggtagcggtggtttttttgtttgcaagcagcagattacgcgcagaaaaaaaggatctcaagaagatcctttgatcttttctacggggtctgacgctcagtggaacgaaaactcacgttaagggattttggtcatgagattatcaaaaaggatcttcacctagatccttttaaattaaaaatgaagttttaaatcaatctaaagtatatatgagtaaacttggtctgacagttaccaatgcttaatcagtgaggcacctatctcagcgatctgtctatttcgttcatccatagttgcctgactccccgtcgtgtagataactacgatacgggagggcttaccatctggccccagtgctgcaatgataccgcgagacccacgctcaccggctccagatttatcagcaataaaccagccagccggaagggccgagcgcagaagtggtcctgcaactttatccgcctccatccagtctattaattgttgccgggaagctagagtaagtagttcgccagttaatagtttgcgcaacgttgttgccattgctacaggcatcgtggtgtcacgctcgtcgtttggtatggcttcattcagctccggttcccaacgatcaaggcgagttacatgatcccccatgttgtgcaaaaaagcggttagctccttcggtcctccgatcgttgtcagaagtaagttggccgcagtgttatcactcatggttatggcagcactgcataattctcttactgtcatgccatccgtaagatgcttttctgtgactggtgagtactcaaccaagtcattctgagaatagtgtatgcggcgaccgagttgctcttgcccggcgtcaatacgggataataccgcgccacatagcagaactttaaaagtgctcatcattggaaaacgttcttcggggcgaaaactctcaaggatcttaccgctgttgagatccagttcgatgtaacccactcgtgcacccaactgatcttcagcatcttttactttcaccagcgtttctgggtgagcaaaaacaggaaggcaaaatgccgcaaaaaagggaataagggcgacacggaaatgttgaatactcatactcttcctttttcaatattattgaagcatttatcagggttattgtctcatgagcggatacatatttgaatgtatttagaaaaataaacaaataggggttccgcgcacatttccccgaaaagtgccacctgacgtctaagaaaccattattatcatgacattaacctataaaaataggcgtatcacgaggccctttcgtc

p-yDR(250bp).EGFP

**250 bp DR = ATATTCAAAACAAACTTTATTTAGTGCCTCAATAATAGTTTGGCCCTGCTAATTCTCCTATTTTATTTTTTAGGGATTCCGGCCACTCTGACCTATATAAACATGGACCGCAGTTTGACGGGTTCACCGGAGCTAATTCCGTATCCAGATTGGCGCTCAAATACAGCTGGAGATTGCGCCAACAGTATTACCACTGCCTACCGCATTAAAGTGGATGAGTGTGGTCGGCTGTGGGTTTTGGACACTGGAA**

p-yDR(500bp).EGFP

**500 bp DR = ACTTCCAGTAGATGTACAAGCAGAAAAAAGAGCCATTAGCACGGCAGTTACCATTGCTTATGATTCCTTGTGTCCAAAATAATGACAAATAGGTATATAAATAATTAAATGCCAAACATAAGCGATTCTAATTTACCTTTACATCTGTATGCATTTACATATTATCCAGAAAACAGACAGCGATAACTTGCAACATTGCTTAGTATAATAATCCAAAGAAGGAATTTAGGCAGAAATTCCAGTTAATTAAATATTCAAAACAAACTTTATTTAGTGCCTCAATAATAGTTTGGCCCTGCTAATTCTCCTATTTTATTTTTTAGGGATTCCGGCCACTCTGACCTATATAAACATGGACCGCAGTTTGACGGGTTCACCGGAGCTAATTCCGTATCCAGATTGGCGCTCAAATACAGCTGGAGATTGCGCCAACAGTATTACCACTGCCTACCGCATTAAAGTGGATGAGTGTGGTCGGCTGTGGGTTTTGGACACTGGAA**

p-ReMET.RFP_*in trans*

GAAGTTCCTATTCCGAAGTTCCTATTCTCTAGAAAGTATAGGAACTTCgtaGGATCTAATTCAATTAGAGACTAATTCAATTAGAGCTAATTCAATTAGGATCCAAGCTTATCGATTTCGAACCCTCGACCGCCGGAGTATAAATAGAGGCGCTTCGTCTACGGAGCGACAATTCAATTCAAACAAGCAAAGTGAACACGTCGCTAAGCGAAAGCTAAGCAAATAAACAAGCGCAGCTGAACAAGCTAAACAATCGGGCCACCATGAGGTCTTCCAAGAATGTTATCAAGGAGTTCATGAGGTTTAAGGTTCGCATGGAAGGAACGGTCAATGGGCACGAGTTTGAAATAGAAGGCGAAGGAGAGGGGAGGCCATACGAAGGCCACAATACCGTAAAGCTTAAGGTAACCAAGGGGGGACCTTTGCCATTTGCTTGGGATATTTTGTCACCACAATTTCAGTATGGAAGCAAGGTATATGTCAAGCACCCTGCCGACATACCAGACTATAAAAAGCTGTCATTTCCTGAAGGATTTAAATGGGAAAGGGTCATGAACTTTGAAGACGGTGGCGTCGTTACTGTAACCCAGGATTCCAGTTTGCAGGATGGCTGTTTCATCTACAAGGTCAAGTTCATTGGCGTGAACTTTCCTTCCGATGGACCTGTTATGCAAAAGAAGACAATGGGCTGGGAAGCCAGCACTGAGCGTTTGTATCCTCGTGATGGCGTGTTGAAAGGAGAGATTCATAAGGCTCTGAAGCTGAAAGACGGTGGTCATTACCTAGTTGAATTCAAAAGTATTTACATGGCAAAGAAGCCTGTGCAGCTACCAGGGTACTACTATGTTGACTCCAAACTGGATATAACAAGCCACAACGAAGACTATACAATCGTTGAGCAGTATGAAAGAACCGAGGGACGCCACCATCTGTTCCTTTAGCGGCCAACGTCGACTAAAGCCAAATAGAAATTATTCAGTTCTGGCTTAAGTTTTTAAAAGTGATATTATTTATTTGGTTGTAACCAACCAAAAGAATGTAAATAACTAATACATAATTATGTTAGTTTTAAGTTAGCAACAAATTGATTTTAGCTATATTAGCTACTTGGTTAATAAATAGAATATATTTATTTAAAGATAATTCGTTTTTATTGTCAGGGAGTGAGTTTGCTTAAAAACTCGTTTAAGCTTATCGATACGCGTACGGCGCGCCAAAGCTTTGTTCgCCTAGGatctgCGGAGTACTGTCCTCCGAGCGGAGTACTGTCCTCCGAGCGGAGTACTGTCCTCCGAGCGGAGTACTGTCCTCCGAGCGGAGTACTGTCCTCCGAGCGGAGACTCTAGCGAGCGAGCCGTAGCTTACCGAAGTATACACTTAAATTCAGTGCACGTTTGCTTGTTGAGAGGAAAGGTTGTGTGCGGACGAATTTTTTTTTGAAAACCGGTGATAGAGCCTGAACCAGAAAAGATAAAAGAAGGCTATACCAGTGGGAGTACACAAACAGAGTAAGTTTGAATAGTAAAAAAAATCATTTATGTAAACAATAACGTGACTGTGCGTTAGGTCCTGTTCATTGGTACCCGCCCGGGGATCATCTGTTCTAGAATGAAAAACATCAAAAAAAACCAGGTAATGAACCTGGGTCCGAACTCTAAACTGCTGAAAGAATACAAATCCCAGCTGATCGAACTGAACATCGAACAGTTCGAAGCAGGTATCGGTCTGATCCTGGGTGATGCTTACATCCGTTCTCGTGATGAAGGTAAAACCTACTGTATGCAGTTCGAGTGGAAAAACAAAGCATACATGGACCACGTATGTCTGCTGTACGATCAGTGGGTACTGTCCCCGCCGCACAAAAAAGAACGTGTTAACCACCTGGGTAACCTGGTAATCACCTGGGGCGCCCAGACTTTCAAACACCAAGCTTTCAACAAACTGGCTAACCTGTTCATCGTTAACAACAAAAAAACCATCCCGAACAACCTGGTTGAAAACTACCTGACCCCGATGTCTCTGGCATACTGGTTCATGGATGATGGTGGTAAATGGGATTACAACAAAAACTCTACCAACAAATCGATCGTACTGAACACCCAGTCTTTCACTTTCGAAGAAGTAGAATACCTGGTTAAGGGTCTGCGTAACAAATTCCAACTGAACTGTTACGTAAAAATCAACAAAAACAAACCGATCATCTACATCGATTCTATGTCTTACCTGATCTTCTACAACCTGATCAAACCGTACCTGATCCCGCAGATGATGTACAAACTGCCGAACACTATCTCCTCCGAAACTTTCCTGAAACCAAAAAAGAAGAGAAAGGTATAAGAAGACCCCAAGGACTTTCCTTCAGAATTGCTAAGTTTTTTGAGTCATGCTGTGTTTAGTAATAGAACTCTTGCTTGCTTTGCTATTTACACCACAAAGGAAAAAGCTGCACTGCTATACAAGAAAATTATGGAAAAATATTTGATGTATAGTGCCTTGACTAGAGATCATAATCAGCCATACCACATTTGTAGAGGTTTTACTTGCTTTAAAAAACCTCCCACACCTCCCCCTGAACCTGAAACATAAAATGAATGCAATTGTTGTTGTTAACTTGTTTATTGCAGCTTATAATGGTTACAAATAAAGCAATAGCATCACAAATTTCACAAATAAAGCATTTTTTTCACTGCATTCTAGTTGTGGTTTGTCCAAACTCATCAATGTATCTTATCATGTCTGGATCCGGTCTCGAAGCCGCGGTGCGGGTGCCAGGGCGTGCCCTTGGGCTCCCCGGGCGCGTACTCCACCTCACCCATCTGGTCGAAGTTCCTATTCCGAAGTTCCTATTCTCTAGAAAGTATAGGAACTTCCCAATGATATCGGAAAGAACATGTGAGCAAAAGGCCAGCAAAAGGCCAGGAACCGTAAAAAGGCCGCGTTGCTGGCGTTTTTCCATAGGCTCCGCCCCCCTGACGAGCATCACAAAAATCGACGCTCAAGTCAGAGGTGGCGAAACCCGACAGGACTATAAAGATACCAGGCGTTTCCCCCTGGAAGCTCCCTCGTGCGCTCTCCTGTTCCGACCCTGCCGCTTACCGGATACCTGTCCGCCTTTCTCCCTTCGGGAAGCGTGGCGCTTTCTCATAGCTCACGCTGTAGGTATCTCAGTTCGGTGTAGGTCGTTCGCTCCAAGCTGGGCTGTGTGCACGAACCCCCCGTTCAGCCCGACCGCTGCGCCTTATCCGGTAACTATCGTCTTGAGTCCAACCCGGTAAGACACGACTTATCGCCACTGGCAGCAGCCACTGGTAACAGGATTAGCAGAGCGAGGTATGTAGGCGGTGCTACAGAGTTCTTGAAGTGGTGGCCTAACTACGGCTACACTAGAAGAACAGTATTTGGTATCTGCGCTCTGCTGAAGCCAGTTACCTTCGGAAAAAGAGTTGGTAGCTCTTGATCCGGCAAACAAACCACCGCTGGTAGCGGTGGTTTTTTTGTTTGCAAGCAGCAGATTACGCGCAGAAAAAAAGGATCTCAAGAAGATCCTTTGATCTTTTCTACGGGGTCTGACGCTCAGTGGAACGAAAACTCACGTTAAGGGATTTTGGTCATGAGATTATCAAAAAGGATCTTCACCTAGATCCTTTTAAATTAAAAATGAAGTTTTAAATCAATCTAAAGTATATATGAGTAAACTTGGTCTGACAGTTACCAATGCTTAATCAGTGAGGCACCTATCTCAGCGATCTGTCTATTTCGTTCATCCATAGTTGCCTGACTCCCCGTCGTGTAGATAACTACGATACGGGAGGGCTTACCATCTGGCCCCAGTGCTGCAATGATACCGCGAGACCCACGCTCACCGGCTCCAGATTTATCAGCAATAAACCAGCCAGCCGGAAGGGCCGAGCGCAGAAGTGGTCCTGCAACTTTATCCGCCTCCATCCAGTCTATTAATTGTTGCCGGGAAGCTAGAGTAAGTAGTTCGCCAGTTAATAGTTTGCGCAACGTTGTTGCCATTGCTACAGGCATCGTGGTGTCACGCTCGTCGTTTGGTATGGCTTCATTCAGCTCCGGTTCCCAACGATCAAGGCGAGTTACATGATCCCCCATGTTGTGCAAAAAAGCGGTTAGCTCCTTCGGTCCTCCGATCGTTGTCAGAAGTAAGTTGGCCGCAGTGTTATCACTCATGGTTATGGCAGCACTGCATAATTCTCTTACTGTCATGCCATCCGTAAGATGCTTTTCTGTGACTGGTGAGTACTCAACCAAGTCATTCTGAGAATAGTGTATGCGGCGACCGAGTTGCTCTTGCCCGGCGTCAATACGGGATAATACCGCGCCACATAGCAGAACTTTAAAAGTGCTCATCATTGGAAAACGTTCTTCGGGGCGAAAACTCTCAAGGATCTTACCGCTGTTGAGATCCAGTTCGATGTAACCCACTCGTGCACCCAACTGATCTTCAGCATCTTTTACTTTCACCAGCGTTTCTGGGTGAGCAAAAACAGGAAGGCAAAATGCCGCAAAAAAGGGAATAAGGGCGACACGGAAATGTTGAATACTCATACTCTTCCTTTTTCAATATTATTGAAGCATTTATCAGGGTTATTGTCTCATGAGCGGATACATATTTGAATGTATTTAGAAAAATAAACAAATAGGGGTTCCGCGCACATTTCCCCGAAAAGTGCCACCTGACGTC

p-ReMET.RFP_*in cis*

aggtggcacttttcggggaaatgtgcgcggaacccctatttgtttatttttctaaatacattcaaatatgtatccgctcatgagacaataaccctgataaatgcttcaataatattgaaaaaggaagagtatgagtattcaacatttccgtgtcgcccttattcccttttttgcggcattttgccttcctgtttttgctcacccagaaacgctggtgaaagtaaaagatgctgaagatcagttgggtgcacgagtgggttacatcgaactggatctcaacagcggtaagatccttgagagttttcgccccgaagaacgttttccaatgatgagcacttttaaagttctgctatgtggcgcggtattatcccgtattgacgccgggcaagagcaactcggtcgccgcatacactattctcagaatgacttggttgagtactcaccagtcacagaaaagcatcttacggatggcatgacagtaagagaattatgcagtgctgccataaccatgagtgataacactgcggccaacttacttctgacaacgatcggaggaccgaaggagctaaccgcttttttgcacaacatgggggatcatgtaactcgccttgatcgttgggaaccggagctgaatgaagccataccaaacgacgagcgtgacaccacgatgcctgtagcaatggcaacaacgttgcgcaaactattaactggcgaactacttactctagcttcccggcaacaattaatagactggatggaggcggataaagttgcaggaccacttctgcgctcggcccttccggctggctggtttattgctgataaatctggagccggtgagcgtgggtctcgcggtatcattgcagcactggggccagatggtaagccctcccgtatcgtagttatctacacgacggggagtcaggcaactatggatgaacgaaatagacagatcgctgagataggtgcctcactgattaagcattggtaactgtcagaccaagtttactcatatatactttagattgatttaaaacttcatttttaatttaaaaggatctaggtgaagatcctttttgataatctcatgaccaaaatcccttaacgtgagttttcgttccactgagcgtcagaccccgtagaaaagatcaaaggatcttcttgagatcctttttttctgcgcgtaatctgctgcttgcaaacaaaaaaaccaccgctaccagcggtggtttgtttgccggatcaagagctaccaactctttttccgaaggtaactggcttcagcagagcgcagataccaaatactgttcttctagtgtagccgtagttaggccaccacttcaagaactctgtagcaccgcctacatacctcgctctgctaatcctgttaccagtggctgctgccagtggcgataagtcgtgtcttaccgggttggactcaagacgatagttaccggataaggcgcagcggtcgggctgaacggggggttcgtgcacacagcccagcttggagcgaacgacctacaccgaactgagatacctacagcgtgagctatgagaaagcgccacgcttcccgaagggagaaaggcggacaggtatccggtaagcggcagggtcggaacaggagagcgcacgagggagcttccagggggaaacgcctggtatctttatagtcctgtcgggtttcgccacctctgacttgagcgtcgatttttgtgatgctcgtcaggggggcggagcctatggaaaaacgccagcaacgcggcctttttacggttcctggccttttgctggccttttgctcacatgttctttccgatatcattgggacgtcggatctaattcaattagagactaattcaattagagctaattcaattaggatccaagcttatcgatttcgaaccctcgaccgccggagtataaatagaggcgcttcgtctacggagcgacaattcaattcaaacaagcaaagtgaacacgtcgctaagcgaaagctaagcaaataaacaagcgcagctgaacaagctaaacaatcgggccaccatgaggtcttccaagaatgttatcaaggagttcatgaggtttaaggttcgcatggaaggaacggtcaatgggcacgagtttgaaatagaaggcgaaggagaggggaggccatacgaaggccacaataccgtaaagcttaaggtaaccaaggggggacctttgccatttgcttgggatattttgtcaccacaatttcagtatggaagcaaggtatatgtcaagcaccctgccgacataccagactataaaaagctgtcatttcctgaaggatttaaatgggaaagggtcatgaactttgaagacggtggcgtcgttactgtaacccaggattccagtttgcaggatggctgtttcatctacaaggtcaagttcattggcgtgaactttccttccgatggacctgttatgcaaaagaagacaatgggctgggaagccagcactgagcgtttgtatcctcgtgatggcgtgttgaaaggagagattcataaggctctgaagctgaaagacggtggtcattacctagttgaattcaaaagtatttacatggcaaagaagcctgtgcagctaccagggtactactatgttgactccaaactggatataacaagccacaacgaagactatacaatcgttgagcagtatgaaagaaccgagggacgccaccatctgttcctttagcggccaacgtcgactaaagccaaatagaaattattcagttctggcttaagtttttaaaagtgatattatttatttggttgtaaccaaccaaaagaatgtaaataactaatacataattatgttagttttaagttagcaacaaattgattttagctatattagctacttggttaataaatagaatatatttatttaaagataattcgtttttattgtcagggagtgagtttgcttaaaaactcgtttaagcttatcgatacgcgtacggcgcgccaaagctttgttcataacttcgtatagcatacattatacgaagttatgtccggaatgcccgggcatgcgcctaggatctgcggagtactgtcctccgagcggagtactgtcctccgagcggagtactgtcctccgagcggagtactgtcctccgagcggagtactgtcctccgagcggagactctagcgagcgagccgtagcttaccgaagtatacacttaaattcagtgcacgtttgcttgttgagaggaaaggttgtgtgcggacgaatttttttttgaaaaccggtgatagagcctgaaccagaaaagataaaagaaggctataccagtgggagtacacaaacagagtaagtttgaatagtaaaaaaaatcatttatgtaaacaataacgtgactgtgcgttaggtcctgttcattggtacccgcccggggatcatctgttctagaatgaaaaacatcaaaaaaaaccaggtaatgaacctgggtccgaactctaaactgctgaaagaatacaaatcccagctgatcgaactgaacatcgaacagttcgaagcaggtatcggtctgatcctgggtgatgcttacatccgttctcgtgatgaaggtaaaacctactgtatgcagttcgagtggaaaaacaaagcatacatggaccacgtatgtctgctgtacgatcagtgggtactgtccccgccgcacaaaaaagaacgtgttaaccacctgggtaacctggtaatcacctggggcgcccagactttcaaacaccaagctttcaacaaactggctaacctgttcatcgttaacaacaaaaaaaccatcccgaacaacctggttgaaaactacctgaccccgatgtctctggcatactggttcatggatgatggtggtaaatgggattacaacaaaaactctaccaacaaatcgatcgtactgaacacccagtctttcactttcgaagaagtagaatacctggttaagggtctgcgtaacaaattccaactgaactgttacgtaaaaatcaacaaaaacaaaccgatcatctacatcgattctatgtcttacctgatcttctacaacctgatcaaaccgtacctgatcccgcagatgatgtacaaactgccgaacactatctcctccgaaactttcctgaaaccaaaaaagaagagaaaggtataagaagaccccaaggactttccttcagaattgctaagttttttgagtcatgctgtgtttagtaatagaactcttgcttgctttgctatttacaccacaaaggaaaaagctgcactgctatacaagaaaattatggaaaaatatttgatgtatagtgccttgactagagatcataatcagccataccacatttgtagaggttttacttgctttaaaaaacctcccacacctccccctgaacctgaaacataaaatgaatgcaattgttgttgttaacttgtttattgcagcttataatggttacaaataaagcaatagcatcacaaatttcacaaataaagcatttttttcactgcattctagttgtggtttgtccaaactcatcaatgtatcttatcatgtctggatcgcgcttattgattggctggtccatcatgatgaacgggtcgaggtggcggtagttgatcccggcgaacgcgcggcgcaccgggaagccctcgccctcgaaaccgctgggcgcggtggtcacggtgagcacgggacgtgcgacggcgtcggctggtgcggatacgcggggcagcgtcagcgggttctcgacggtcacggcgggcatgtcgacaagccgaattgatccactagaaggcctaattcgtctagcaggatctctagggcttcgaccgttttaacctcgaaatatgcacatgtaaggacggatgtgagcgaacgccagtgatgaccgggatcagaggtaacctaccatggtggggattaggtgaccgttcgcaggtagtttgatcggagcgaatgttcggggggtctggcgtcagaggctctaaactttatgtaattcctgccgcgaaacacgcacgtatcaagcagtcagctgttctcttcgttcagcgcgcgccggtgttgcaaaacgagcgctcttcgccggcggtggctcgtgcgatagttcgttttgtcggtaatccgatgttgccgcgccgatatcatgtgatgttgtcacagtgcgcgaaattcgaatggtggtgtgcagtgattgtgttgtgacggcgagtggcgcgtgtgggtgcttagttttgggagatgttttcgtatttttttgttgataactcaggctttgttgctgtgttgtagtactattttccattgcgcggtgtccagcttttaattagtggcacatattcttagcaagtaaaaattattttgcatactattaaatttcttataaattattttctaaaattaagtttaccttttcaattttactaaaaatatcgatatatttattatcgctggaaaactacattattccacctctaagcaagaaccgttagttggcgcgtagctttaccacaaaattcctggaattgccgtacgcttcgcagttgtttcaagttgtctaagggacatacgattttttttgcctctgcgtcacgattttaacccaaaagcgagtttagttacatgtacattattattagataaagaagtatcgcgaatacttcagttgaataaactgtgcttggtttttgggtgaggatttgtggaaagtagagtgcgcgataaccgtaactttcgacccggattttcgcctgaattgagatctctctagaggtaccgccaccatggccccaaagaagaagcggaaggtcggtatccacggtgtcccagcagcccaaatggactcccagcagccagatctgaagctactgtcttctatcgaacaagcatgcgatatttgccgacttaaaaagctcaagtgctccaaagaaaaaccgaagtgcgccaagtgtctgaagaacaactgggagtgtcgctactctcccaaaaccaaaaggtctccgctgactagggcacatctgacagaagtggaatcaaggctagaaagactggaacagctatttctactgatttttcctcgagaagaccttgacatgattttgaaaatggattctttacaggatataaaagcattgttagaattcccgggtgtcgaccagaaaaagttcaataaagtcagagttgtgagagcactggatgctgttgctctcccacagccagtgggcgttccaaatgaaagccaagccctaagccagagattcactttttcaccaggtcaagacatacagttgattccaccactgatcaacctgttaatgagcattgaaccagatgtgatctatgcaggacatgacaacacaaaacctgacacctccagttctttgctgacaagtcttaatcaactaggcgagaggcaacttctttcagtagtcaagtggtctaaatcattgccaggttttcgaaacttacatattgatgaccagataactctcattcagtattcttggatgagcttaatggtgtttggtctaggatggagatcctacaaacacgtcagtgggcagatgctgtattttgcacctgatctaatactaaatgaacagcggatgaaagaatcatcattctattcattatgccttaccatgtggcagatcccacaggagtttgtcaagcttcaagttagccaagaagagttcctctgtatgaaagtattgttacttcttaatacaattcctttggaagggctacgaagtcaaacccagtttgaggagatgaggtcaagctacattagagagctcatcaaggcaattggtttgaggcaaaaaggagttgtgtcgagctcacagcgtttctatcaacttacaaaacttcttgataacttgcatgatcttgtcaaacaacttcatctgtactgcttgaatacatttatccagtcccgggcactgagtgttgaatttccagaaatgatgtctgaagttattgctgggtcgacgagatatcaagcagaattccagtacctgccagatacagacgatcgtcaccggattgaggagaaacgtaaaaggacatatgagaccttcaagagcatcatgaagaagagtcctttcagcggacccaccgacccccggcctccacctcgacgcattgctgtgccttcccgcagctcagcttctgtccccaagccagcaccccagccctatccctttacgtcatccctgagcaccatcaactatgatgagtttcccaccatggtgtttccttctgggcagatcagccaggcctcggccttggccccggcccctccccaagtcctgccccaggctccagcccctgcccctgctccagccatggtatcagctctggcccaggccccagcccctgtcccagtcctagccccaggccctcctcaggctgtggccccacctgcccccaagcccacccaggctggggaaggaacgctgtcagaggccctgctgcagctgcagtttgatgatgaagacctgggggccttgcttggcaacagcacagacccagctgtgttcacagacctggcatccgtcgacaactccgagtttcagcagctgctgaaccagggcatacctgtggccccccacacaactgagcccatgctgatggagtaccctgaggctataactcgcctagtgacaggggcccagaggccccccgacccagctcctgctccactgggggccccggggctccccaatggcctcctttcaggagatgaagacttctcctccattgcggacatggacttctcagccctgctgagtcagatcagctcctaatctagcacctaggaggatccactcgaggtttagagagggcgaatccagctctggagcagaggctctggcagcttttgcagcgtttatataacatgaaatatatatacgcattccgatcaaagctgggttaaccagatagatagatagtaacgtttaaatagcgcctggcgcgttcgattttaaagagatttagagcgttatcccgtgcctatagatcatatagtatagacaacgaacgatcactcaaatccaagtcaataattcaagaatttatgtctgtttctgtgaaagggaaactaattttgttaaagaagacttacaatatcgtaatacttgttcaatcgtcgtggccgatagaaatatcttacaatccgaaagttgatgaatggaattggtctgcaactggtcgccttcatttcgtaaaatgttcgcttgcggccgaaaaatttcgatatatctacaattcatctacaatctttactaaattttgaaaaaggaacactttgaatttcgaactgtcaatcgtatcattagaatttaatctaaatttaaatcttgctaaaggaaatagcaaggaacactttcgtcgtcggctacgcattcattgtaaaattttaaattttgacattccgcactttttgatagataagcgaagagtatttttattacatgtatcgcaagtattcatttcaacacacatatctatatatatatatatatatatatatatatatatatatatatatgttatatatttattcaattttgtttaccattgatcaatttttcacacatgaaacaaccgccagcattatataatttttttatttttttaaaaaatgtgtacacatattctgaaaatgaaaaattcaatggctcgactgccaaataaagaaatggttacaatttaaggaaacaaatgtccttcttgcactaggggggtgacatccctaggtccggagaatgtttaaacagaagttcctattccgaagttcctattctctagaaagtataggaacttctcgtagtgccccaactggggtaacctttgagttctctcagttgggggcgtagcggtctcgaagccgcggtgcgggtgccagggcgtgcccttgggctccccgggcgcgtactccacctcacccatctggtccacctgcagggacgtc

p-yMCR.EGFP

ATACGCAAACCGCCTCTCCCCGCGCGTTGGCCGATTCATTAATGCAGCTGGCACGACAGGTTTCCCGACTGGAAAGCGGGCAGTGAGCGCAACGCAATTAATGTGAGTTAGCTCACTCATTAGGCACCCCAGGCTTTACACTTTATGCTTCCGGCTCGTATGTTGTGTGGAATTGTGAGCGGATAACAATTTCACACAGGAAACAGCTATGACCATGATTACGCCAAGCTATTTAGGTGACACTATAGAATACTCAAGCTATGCATCAAGCTTGGTACCGAGCTCGGATCCACTAGTAACGGCCGCCAGTGTGCTGGAATTCGCCCTTGTTGCGAGGTTTTAGGACTGAAAGAGCACATGTCAAAATATAAATTTGTTCAAATACTTTATATTTGACTGAATTAGATTGTTATTTTAAAAGTTATGAATTAAATAAAGATTGAAAGGTGCATTATGCTCAAATGTATATTTATCGCAACCCCCGGTTACTTTGTAAAGCAAAAACGCCTGGTTTGATTTTTAAGAAGATGGGTCGGTAAATCGATAAAAGCTATATTTTCTGGTCGTTGCAGTCTCACTCGCCTGCTATAAAAACATTAAAAGTTCCCAGAAACAATAAATGTCTTTAAATTCAATTAACGAAGAAATAAAGAAGGAAAAGAACTGGAGCGGAAATCGGTCGAAATACTGCCAATGGCCACATATACATTTAACAGCGATATATGGTATACATATTGATAATGATGTCAGACGCAATTGCTTCAGACGGCTAATGACATCGCAAATTGCACGCAACTTGCAATAGTGCCAATTATGACTGAAGTACATATAGCCGGGGATCTTTTAACATAAACTTCCAGTAGATGTACAAGCAGAAAAAAGAGCCATTAGCACGGCAGTTACCATTGCTTATGATTCCTTGTGTCCAAAATAATGACAAATAGGTATATAAATAATTAAATGCCAAACATAAGCGATTCTAATTTACCTTTACATCTGTATGCATTTACATATTATCCAGAAAACAGACAGCGATAACTTGCAACATTGCTTAGTATAATAATCCAAAGAAGGAATTTAGGCAGAAATTCCAGTTAATTAAATATTCAAAACAAACTTTATTTAGTGCCTCAATAATAGTTTGGCCCTGCTAATTCTCCTATTTTATTTTTTAGGGATTCCGGCCACTCTGACCTATATAAACATGGACCGCAGTTTGACGGGTTCACCGGAGCTAATTCCGTATCCAGATTGGCGCTCAAATACAGCTGGAGATTGCGCCAACAGTATTACCACTGCCTACCGCATTAAAGTGGATGAGTGTGGTCGGCTGTGGGTTTTGGACACTGGAAGCTAGCAACACGAAGAGCAGCAGTGTGGTGGATTTCTCAGTTATTGATTTTGCTTCTGCAAAGACAGCAAACGCAAAAGGGCAACATTTGCTGGTCGGCATGCTTTTCGGGGGAGGAGGACTGGGCGCGAGCAGGGCGAGGCATGCATTCAAAAAGCCGGCTCCAACGGAGAGCGAGAGAGCGCAAGAGCCAAGTGTACAGCAAGGCAAACAACAAACAACGAAACACGAAGGATGAGTACTTAAAACCAAACACGTGATAGTACACGGTATTAGTTGGGATCAAAGTGCACGTTTCTGTTAAGTTTTGCAACCGCTCGTTATTAAAAGCAGTTCACAAAATACTTAAAAGAAATCGAATTAGAGCCAGAACATTTTTTCAAATTATATTTTGTTATACATTTCTTTGTAAAAGGAAATTAAAATAGAAAATTCGAATGGCTTGCATATCCTAAATATGCATTTTATTATTATATGCGTTATAATACATATAATTAATATGTTATTAATATCTAACTTATTTAACTAATTACATCAATTACTGCATTACATTTAATTATTTGAAATCTATGTCCATACATTgagctcaggcctagattaCTTTTTCTTTTTTGCCTGGCCGGCCTTTTTCGTGGCCGCCGGCCTTTTGTCGCCTCCCAGCTGAGACAGGTCGATCCGTGTCTCGTACAGGCCGGTGATGCTCTGGTGGATCAGGGTGGCGTCCAGCACCTCTTTGGTGCTGGTGTACCTCTTCCGGTCGATGGTGGTGTCAAAGTACTTGAAGGCGGCAGGGGCTCCCAGATTGGTCAGGGTAAACAGGTGGATGATATTCTCGGCCTGCTCTCTGATGGGCTTATCCCGGTGCTTGTTGTAGGCGGACAGCACTTTGTCCAGATTAGCGTCGGCCAGGATCACTCTCTTGGAGAACTCGCTGATCTGCTCGATGATCTCGTCCAGGTAGTGCTTGTGCTGTTCCACAAACAGCTGTTTCTGCTCATTATCCTCGGGGGAGCCCTTCAGCTTCTCATAGTGGCTGGCCAGGTACAGGAAGTTCACATATTTGGAGGGCAGGGCCAGTTCGTTTCCCTTCTGCAGTTCGCCGGCAGAGGCCAGCATTCTCTTCCGGCCGTTTTCCAGCTCGAACAGGGAGTACTTAGGCAGCTTGATGATCAGGTCCTTTTTCACTTCTTTGTAGCCCTTGGCTTCCAGAAAGTCGATGGGATTCTTCTCGAAGCTGCTTCTTTCCATGATGGTGATCCCCAGCAGCTCTTTCACACTCTTCAGTTTCTTGGACTTGCCCTTTTCCACTTTGGCCACCACCAGCACAGAATAGGCCACGGTGGGGCTGTCGAAGCCGCCGTACTTCTTAGGGTCCCAGTCCTTCTTTCTGGCGATCAGCTTATCGCTGTTCCTCTTGGGCAGGATAGACTCTTTGCTGAAGCCGCCTGTCTGCACCTCGGTCTTTTTCACGATATTCACTTGGGGCATGCTCAGCACTTTCCGCACGGTGGCAAAATCCCGGCCCTTATCCCACACGATCTCCCCGGTTTCGCCGTTTGTCTCGATCAGAGGCCGCTTCCGGATCTCGCCGTTGGCCAGGGTAATCTCGGTCTTGAAAAAGTTCATGATGTTGCTGTAGAAGAAGTACTTGGCGGTAGCCTTGCCGATTTCCTGCTCGCTCTTGGCGATCATCTTCCGCACGTCGTACACCTTGTAGTCGCCGTACACGAACTCGCTTTCCAGCTTAGGGTACTTTTTGATCAGGGCGGTTCCCACGACGGCGTTCAGGTAGGCGTCGTGGGCGTGGTGGTAGTTGTTGATCTCGCGCACTTTGTAAAACTGGAAATCCTTCCGGAAATCGGACACCAGCTTGGACTTCAGGGTGATCACTTTCACTTCCCGGATCAGCTTGTCATTCTCGTCGTACTTAGTGTTCATCCGGGAGTCCAGGATCTGTGCCACGTGCTTTGTGATCTGCCGGGTTTCCACCAGCTGTCTCTTGATGAAGCCGGCCTTATCCAGTTCGCTCAGGCCGCCTCTCTCGGCCTTGGTCAGATTGTCGAACTTTCTCTGGGTAATCAGCTTGGCGTTCAGCAGCTGCCGCCAGTAGTTCTTCATCTTCTTCACGACCTCTTCGGAGGGCACGTTGTCGCTCTTGCCCCGGTTCTTGTCGCTTCTGGTCAGCACCTTGTTGTCGATGGAGTCGTCCTTCAGAAAGCTCTGAGGCACGATATGGTCCACATCGTAGTCGGACAGCCGGTTGATGTCCAGTTCCTGGTCCACGTACATATCCCGCCCATTCTGCAGGTAGTACAGGTACAGCTTCTCGTTCTGCAGCTGGGTGTTTTCCACGGGGTGTTCTTTCAGGATCTGGCTGCCCAGCTCTTTGATGCCCTCTTCGATCCGCTTCATTCTCTCGCGGCTGTTCTTCTGTCCCTTCTGGGTGGTCTGGTTCTCTCTGGCCATTTCGATCACGATGTTCTCGGGCTTGTGCCGGCCCATCACTTTCACGAGCTCGTCCACCACCTTCACTGTCTGCAGGATGCCCTTCTTAATGGCGGGGCTGCCGGCCAGATTGGCAATGTGCTCGTGCAGGCTATCGCCCTGGCCGGACACCTGGGCTTTCTGGATGTCCTCTTTAAAGGTCAGGCTGTCGTCGTGGATCAGCTGCATGAAGTTTCTGTTGGCGAAGCCGTCGGACTTCAGGAAATCCAGGATTGTCTTGCCGGACTGCTTGTCCCGGATGCCGTTGATCAGCTTCCGGCTCAGCCTGCCCCAGCCGGTGTATCTCCGCCGCTTCAGCTGCTTCATCACTTTGTCGTCGAACAGGTGGGCATAGGTTTTCAGCCGTTCCTCGATCATCTCTCTGTCCTCAAACAGTGTCAGGGTCAGCACGATATCTTCCAGAATGTCCTCGTTTTCCTCATTGTCCAGGAAGTCCTTGTCCTTGATAATTTTCAGCAGATCGTGGTATGTGCCCAGGGAGGCGTTGAACCGATCTTCCACGCCGGAGATTTCCACGGAGTCGAAGCACTCGATTTTCTTGAAGTAGTCCTCTTTCAGCTGCTTCACGGTCACTTTCCGGTTGGTCTTGAACAGCAGGTCCACGATGGCCTTTTTCTGCTCGCCGCTCAGGAAGGCGGGCTTTCTCATTCCCTCGGTCACGTATTTCACTTTGGTCAGCTCGTTATACACGGTGAAGTACTCGTACAGCAGGCTGTGCTTGGGCAGCACCTTCTCGTTGGGCAGGTTCTTATCGAAGTTGGTCATCCGCTCGATGAAGCTCTGGGCGGAAGCGCCCTTGTCCACCACTTCCTCGAAGTTCCAGGGGGTGATGGTTTCCTCGCTCTTTCTGGTCATCCAGGCGAATCTGCTGTTTCCCCTGGCCAGAGGGCCCACGTAGTAGGGGATGCGGAAGGTCAGGATCTTCTCGATCTTTTCCCGGTTGTCCTTCAGGAATGGGTAAAAATCTTCCTGCCGCCGCAGAATGGCGTGCAGCTCTCCCAGGTGGATCTGGTGGGGGATGCTGCCGTTGTCGAAGGTCCGCTGCTTCCGCAGCAGGTCCTCTCTGTTCAGCTTCACGAGCAGTTCCTCGGTGCCGTCCATCTTTTCCAGGATGGGCTTGATGAACTTGTAGAACTCTTCCTGGCTGGCTCCGCCGTCAATGTAGCCGGCGTAGCCGTTCTTGCTCTGGTCGAAGAAAATCTCTTTGTACTTCTCAGGCAGCTGCTGCCGCACGAGAGCTTTCAGCAGGGTCAGGTCCTGGTGGTGCTCGTCGTATCTCTTGATCATAGAGGCGCTCAGGGGGGCCTTGGTGATCTCGGTGTTCACTCTCAGGATGTCGCTCAGCAGGATGGCGTCGGACAGGTTCTTGGCGGCCAGAAACAGGTCGGCGTACTGGTCGCCGATCTGGGCCAGCAGGTTGTCCAGGTCGTCGTCGTAGGTGTCCTTGCTCAGCTGCAGTTTGGCATCCTCGGCCAGGTCGAAGTTGCTCTTGAAGTTGGGGGTCAGGCCCAGGCTCAGGGCAATCAGGTTGCCGAACAGGCCATTCTTCTTCTCGCCGGGCAGCTGGGCGATCAGATTTTCCAGCCGTCTGCTCTTGCTCAGTCTGGCAGACAGGATGGCCTTGGCGTCCACGCCGCTGGCGTTGATGGGGTTTTCCTCGAACAGCTGGTTGTAGGTCTGCACCAGCTGGATGAACAGCTTGTCCACGTCGCTGTTGTCGGGGTTCAGGTCGCCCTCGATCAGGAAGTGGCCCCGGAACTTGATCATGTGGGCCAGGGCCAGATAGATCAGCCGCAGGTCGGCCTTGTCGGTGCTGTCCACCAGTTTCTTTCTCAGGTGGTAGATGGTGGGGTACTTCTCGTGGTAGGCCACCTCGTCCACGATGTTGCCGAAGATGGGGTGCCGCTCGTGCTTCTTATCCTCTTCCACCAGGAAGGACTCTTCCAGTCTGTGGAAGAAGCTGTCGTCCACCTTGGCCATCTCGTTGCTGAAGATCTCTTGCAGATAGCAGATCCGGTTCTTCCGTCTGGTGTATCTTCTTCTGGCGGTTCTCTTCAGCCGGGTGGCCTCGGCTGTTTCGCCGCTGTCGAACAGCAGGGCTCCGATCAGGTTCTTCTTGATGCTGTGCCGGTCGGTGTTGCCCAGCACCTTGAATTTCTTGCTGGGCACCTTGTACTCGTCGGTGATCACGGCCCAGCCCACAGAGTTGGTGCCGATGTCCAGGCCGATGCTGTACTTCTTGTCGGCTGCTGGGACTCCGTGGATACCGACCTTCCGCTTCTTCTTTGGGGCCATCTTATCGTCATCGTCTTTGTAATCAATATCATGATCCTTGTAGTCTCCGTCGTGGTCCTTATAGTCCATtctaggcgcgccagatctggtaccgATTGATATTTTTTTTTTAATTTGGCCTGCCTTTCAAGAGCAAATTAATTCTTTTTCACTTTATGTATTACCTTACAAACTTTGATAAGTTCAGATCACTTCGCGCAACGCAAAACCGAAAAGGATCTGTTAAAAGTACCAAAAATCTAGTGGTGGAATTTCCCATTGTGCTATCGCAATTCGATATTCACAATTTCGATAATTTATATATATAGCCGTTTTCCACTAACACATCAAATGTGATTTAGCTATTAATGAAATTGTAATATTCAAGCAACCTAAGTGATTCATTCATATGTACATACTTATATGTAAAGATGGTGGTGGCATTTTTAACATGGGGAAGTTCTTATTTATTTACTTTTTCGTTTGTTATAGGTGAATTCCTCTTTATTTTTATACAATATAATTTTATTTTACGTTAAGCAACCAATTTAAGGGCTATGAAACGTAGTAACTAACAGGAGCAACGAAGATAACATGGAAACATACAAAATTTGTATAAAAATATTGATTAGATAATAGTTTGGCTTGTTTATGCCACGTGTCCAAAGATATCTTTGATGACGCCTCCTCAGTGTTCTCCTGGTTGTCTGGTATGTGCCTGATTTAATTTGTTCTCAAACTGAAGGTGGATGCTGAGGTCATGTGCGATATCAACATTTCTCGCGTACCAGGGCGCGTTGGTGATTGTTCGCAACGCTTGGTTTTGGAGCCGTTGCCCTCTCACTATGTCCCACCGCCGATTTGACCCGCAGACGTATCCCGCTTGTCCCCAACAACCGCACTTCATCATAATACATCATCTCTAGACCACACTCAAACGATTTATTTATTAAAACTAAGTGATATATAGGGAATTGTTATTGAAGGTAATTAGTATAATTCAAAAACGCCCAACACGCTGTTCTTTCATTGAAATACAGTGGAGCCCTGCTTGCAAGCAGTGCAAGTTTAAAAAACATCGCAATGCTATGTGTGTGTACTCGCTTAAAAGCCAGATGTACATTAAAATACGGTAACACTGTTGACTTCTGAAACCCAAACTACTTCCAGTTGGGAATTGTATTAGCCATGGGAAGGCATTTCTTGACAAGCCACCGCCCGGTAACACTAAAACGCGTTAGGGTGTTCTGTCAACTCGTTCTGCGAATACAGAAACGAAAAAAATAGTAAACAACATTTATTGGAGGGAAAAAAAGAAATAGCCCGGCTTCCGCATAAACAACTGTCGCAGACCCGCAGCACCGATTAAAGCCGACAGCCGACGCCAGTTAACCTAACCTCTGATCAAATTAAGTGGGCCAAAATGAGCGTGGAGGAGGAAGTGTTTCGAATCCAAAAGAAGATGAGCAAGATGGCCAGCGACGGCACAGTAAGTATCGGGATTAGCTAACGTGCCGCGCATCATGTCACAAATATACATATATCCACCGTACAGGGACAGGATCAGGCTCTGGACCTGCTGAAGGCCCTGCAAACGCTTAACATCAATCTCGACATTCTGACCAAAACGCGCATCGGCATGACCGTAAACGAGCTGCGCAAGAGTAGCAAGGACGACGAGGTGATCGCTCTGGCCAAGACGCTGATCAAGAACTGGAAACGATTCCTCGCCAGCCCGGCGCCAACCACACCGAACAACAGCTCCGCTAAGGAGGGCTCCTCCAACAACAGCAGTGCCTCGAAGTCCACCAGTGCCGCCAAGTCGTCGTCGTCAATCTCCGGCAAGGATAAGTCCAGTTCATCCAGCTCATCCAAGGATAAAGAGAAGAAAGGCTCGACATCCTCGTCACAGACCTCGTTTCCCTCCGGCGGCATGACAGATGCGGTCCGCATCAAATGCCGCGAAATGCTGGCCACCGCACTGAAGATTGGTGAAGTGCCCGAAGGATGCGGTGAGCCGGAGGAAATGGCCGCCGAACTGGAGGATGCCATTTACTCCGAGTTCAATAACACGGATATGAAGTACAAGAATCGCATTAGGTCACGCGTGGCCAATCTGAAGGACCCCAAGAATCCTGGATTGCGCGGCAACTTTATGTGCGGCGCCGTCACTGCCAAGCAGCTGGCCAAAATGACGCCGGAGGAGATGGCCAGCGACGAGATGAAGAAGCTGCGCGAGAAGTTCGTCAAGGAGGCCATCAACGATGCCCAGCTGGCCACCGTGCAGGGCACCAAGACCGATCTCCTCAAGTGCGCCAAGTGCAAGAAGCGCAACTGCACCTACAACCAGCTGCAGGGATCCTTTTTTGCTCACCTGTGATTGCTCCTACTCAAATACAAAAACATCAAATTTTCTGTCAATAAAGCATATTTATTTATATTTATTTTACAGGAAAGAATTCCTTTTAAAGTGTATTTTAACCTATAATGAAAAACGATTAAAAAAAATACATAAAATAATTCGAAAATTTTTGAATAGCCCAGGTTGATAAAAATTCATTTCATACGTTTTATAACTTATGCCCCTAAGTATTTTTTGACCATAGTGTTTCAATTCTACATTAATTTTACAGAGTAGAATGAAACGCCACCTACTCAGCCAAGAGGCGAAAAGGTTAGCTCGCCAAGCAGAGAGGGCGCCAGTGCTCACTACTTTTTATAATTCTCAACTTCTTTTTCCAGACTCAGTTCGTATATATAGACCTATTTTCAATTTAACGTCGGTTTTGGACACTGGAACCGGTTTTAGAGCTAGAAATAGCAAGTTAAAATAAGGCTAGTCCGTTATCAACTTGAAAAAGTGGCACCGAGTCGGTGCTTTTTTGCCTACCTGGAGCCTGAGAGTTGTTCAATAAAATAAAAATGTTTCGTTTTTTTGCTTTCGCCAGTATTTATTATTTTTCATCAATATGTATTCAATTTGGTATGTATTTAGTAATTGTAATATATAGACAATGGTTTTCCGTTGACGTACATACATCTGACGTGTGTTTATTTAGACATAATAGTTATGTTTTCACATCTTTTTAATGTTCGCTTAATGCGTATGCATTTGACGTGGATCTAATTCAATTAGAGACTAATTCAATTAGAGCTAATTCAATTAGGATCCAAGCTTATCGATTTCGAACCCTCGACCGCCGGAGTATAAATAGAGGCGCTTCGTCTACGGAGCGACAATTCAATTCAAACAAGCAAAGTGAACACGTCGCTAAGCGAAAGCTAAGCAAATAAACAAGCGCAGCTGAACAAGCTAAACAATCGGGGTACCGCTAGAGTCGACGGTACCGCGGGCCCGGGATCCACCGGTCGCCACCATGGTGAGCAAGGGCGAGGAGCTGTTCACCGGGGTGGTGCCCATCCTGGTCGAGCTGGACGGCGACGTAAACGGCCACAAGTTCAGCGTGTCCGGCGAGGGCGAGGGCGATGCCACCTACGGCAAGCTGACCCTGAAGTTCATCTGCACCACCGGCAAGCTGCCCGTGCCCTGGCCCACCCTCGTGACCACCCTGACCTACGGCGTGCAGTGCTTCAGCCGCTACCCCGACCACATGAAGCAGCACGACTTCTTCAAGTCCGCCATGCCCGAAGGCTACGTCCAGGAGCGCACCATCTTCTTCAAGGACGACGGCAACTACAAGACCCGCGCCGAGGTGAAGTTCGAGGGCGACACCCTGGTGAACCGCATCGAGCTGAAGGGCATCGACTTCAAGGAGGACGGCAACATCCTGGGGCACAAGCTGGAGTACAACTACAACAGCCACAACGTCTATATCATGGCCGACAAGCAGAAGAACGGCATCAAGGTGAACTTCAAGATCCGCCACAACATCGAGGACGGCAGCGTGCAGCTCGCCGACCACTACCAGCAGAACACCCCCATCGGCGACGGCCCCGTGCTGCTGCCCGACAACCACTACCTGAGCACCCAGTCCGCCCTGAGCAAAGACCCCAACGAGAAGCGCGATCACATGGTCCTGCTGGAGTTCGTGACCGCCGCCGGGATCACTCTCGGCATGGACGAGCTGTACAAGTAAAGCGGCCTAAGCGATCGCTCAGGCCGCGACTCTAGATCATAATCAGCCATACCACATTTGTAGAGGTTTTACTTGCTTTAAAAAACCTCCCACACCTCCCCCTGAACCTGAAACATAAAATGAATGCAATTGTTGTTGTTAACTTGTTTATTGCAGCTTATAATGGTTACAAATAAAGCAATAGCATCACAAATTTCACAAATAAAGCATTTTTTTCACTGCATTCTAGTTGTGGTTTGTCCAAACTCATCAATGTATCTTACTCCGTGGGCATCGGCAATACCACCACTAATCCGTGCCCCTATGCGGTAAATGTCTTTGACTTGACCACGGATACGCGAATTCGGAGATACGAGCTACCTGGCGTGGACACAAATCCAAATACTTTCATAGCTAACATTGCCGTGGATATAGGCAAAAATTGCGATGATGCATATGCCTATTTTGCCGATGAATTGGGATACGGCTTGATTGCTTACTCCTGGGAACTGAACAAGTCCTGGAGATTCTCGGCACATTCGTATTTTTTCCCCGATCCATTGAGGGGCGATTTCAATGTCGCTGGTATTAACTTCCAATGGGGCGAGGAGGGTATATTTGGTATGTCCCTTTCGCCCATTCGATCGGATGGTTATCGTACCCTGTACTTTAGTCCGTTAGCAAGTCATCGACAATTTGCCGTATCCACGAGGATTTTGAGGGATGAAACCAGGACGGAAGATAGCTATCATGACTTTGTTGCCTTAGATGAACGGGGTCCAAACTCCCATACCACTTCACGTGTGATGAGCGATGATGGAATTGAGCTGTTCAATTTAATAGATCAAAATGCAGTGGGTTGCTGGCACTCATCAATGCCGTACTCACCGCAATTTCATGGCATTGTGGATCGCGATGACGTTGGCTTAGTTTTTCCGGCCGATGTGAAAATTGATGAGAACAAAAACGTTTGGGTTCTATCCGATAGGATGCCCGTTTTCTTGCTGTCTGACTTGGATTATTCAGATACTAATTTCCGAATTTACACGGCTCCCTTGGCCACTTTAATTGAGAATACTGTGTGTGATTTGAGGAATAACGCCTATGGGCCGCCAAATACCGTTTCAATACCAAAACAAGCCGTTTTGCCAATGGGTCCACCGTTATATACGAAACAATATCGTCCTGTCTTGCCACAGAAACCTCAGACCAGCTGGGCTTCCTCGCCGCCTCCTCCAAGTCGCACTTATTTGCCCGCCAATTCAGGCAATGTAGTCTCCAGAAGGGCGAATTCTGCAGATATCCATCACACTGGCGGCCGCTCGAGCATGCATCTAGAGGGCCCAATTCGCCCTATAGTGAGTCGTATTACAATTCACTGGCCGTCGTTTTACAACGTCGTGACTGGGAAAACCCTGGCGTTACCCAACTTAATCGCCTTGCAGCACATCCCCCTTTCGCCAGCTGGCGTAATAGCGAAGAGGCCCGCACCGATCGCCCTTCCCAACAGTTGCGCAGCCTGAATGGCGAATGGACGCGCCCTGTAGCGGCGCATTAAGCGCGGCGGGTGTGGTGGTTACGCGCAGCGTGACCGCTACACTTGCCAGCGCCCTAGCGCCCGCTCCTTTCGCTTTCTTCCCTTCCTTTCTCGCCACGTTCGCCGGCTTTCCCCGTCAAGCTCTAAATCGGGGGCTCCCTTTAGGGTTCCGATTTAGTGCTTTACGGCACCTCGACCCCAAAAAACTTGATTAGGGTGATGGTTCACGTAGTGGGCCATCGCCCTGATAGACGGTTTTTCGCCCTTTGACGTTGGAGTCCACGTTCTTTAATAGTGGACTCTTGTTCCAAACTGGAACAACACTCAACCCTATCTCGGTCTATTCTTTTGATTTATAAGGGATTTTGCCGATTTCGGCCTATTGGTTAAAAAATGAGCTGATTTAACAAAAATTTAACGCGAATTTTAACAAAATTCAGGGCGCAAGGGCTGCTAAAGGAAGCGGAACACGTAGAAAGCCAGTCCGCAGAAACGGTGCTGACCCCGGATGAATGTCAGCTACTGGGCTATCTGGACAAGGGAAAACGCAAGCGCAAAGAGAAAGCAGGTAGCTTGCAGTGGGCTTACATGGCGATAGCTAGACTGGGCGGTTTTATGGACAGCAAGCGAACCGGAATTGCCAGCTGGGGCGCCCTCTGGTAAGGTTGGGAAGCCCTGCAAAGTAAACTGGATGGCTTTCTTGCCGCCAAGGATCTGATGGCGCAGGGGATCAAGATCTGATCAAGAGACAGGATGAGGATCGTTTCGCATGATTGAACAAGATGGATTGCACGCAGGTTCTCCGGCCGCTTGGGTGGAGAGGCTATTCGGCTATGACTGGGCACAACAGACAATCGGCTGCTCTGATGCCGCCGTGTTCCGGCTGTCAGCGCAGGGGCGCCCGGTTCTTTTTGTCAAGACCGACCTGTCCGGTGCCCTGAATGAACTGCAGGACGAGGCAGCGCGGCTATCGTGGCTGGCCACGACGGGCGTTCCTTGCGCAGCTGTGCTCGACGTTGTCACTGAAGCGGGAAGGGACTGGCTGCTATTGGGCGAAGTGCCGGGGCAGGATCTCCTGTCATCCCACCTTGCTCCTGCCGAGAAAGTATCCATCATGGCTGATGCAATGCGGCGGCTGCATACGCTTGATCCGGCTACCTGCCCATTCGACCACCAAGCGAAACATCGCATCGAGCGAGCACGTACTCGGATGGAAGCCGGTCTTGTCGATCAGGATGATCTGGACGAAGAGCATCAGGGGCTCGCGCCAGCCGAACTGTTCGCCAGGCTCAAGGCGCGCATGCCCGACGGCGAGGATCTCGTCGTGACCCATGGCGATGCCTGCTTGCCGAATATCATGGTGGAAAATGGCCGCTTTTCTGGATTCATCGACTGTGGCCGGCTGGGTGTGGCGGACCGCTATCAGGACATAGCGTTGGCTACCCGTGATATTGCTGAAGAGCTTGGCGGCGAATGGGCTGACCGCTTCCTCGTGCTTTACGGTATCGCCGCTCCCGATTCGCAGCGCATCGCCTTCTATCGCCTTCTTGACGAGTTCTTCTGAATTGAAAAAGGAAGAGTATGAGTATTCAACATTTCCGTGTCGCCCTTATTCCCTTTTTTGCGGCATTTTGCCTTCCTGTTTTTGCTCACCCAGAAACGCTGGTGAAAGTAAAAGATGCTGAAGATCAGTTGGGTGCACGAGTGGGTTACATCGAACTGGATCTCAACAGCGGTAAGATCCTTGAGAGTTTTCGCCCCGAAGAACGTTTTCCAATGATGAGCACTTTTAAAGTTCTGCTATGTGGCGCGGTATTATCCCGTATTGACGCCGGGCAAGAGCAACTCGGTCGCCGCATACACTATTCTCAGAATGACTTGGTTGAGTACTCACCAGTCACAGAAAAGCATCTTACGGATGGCATGACAGTAAGAGAATTATGCAGTGCTGCCATAACCATGAGTGATAACACTGCGGCCAACTTACTTCTGACAACGATCGGAGGACCGAAGGAGCTAACCGCTTTTTTGCACAACATGGGGGATCATGTAACTCGCCTTGATCGTTGGGAACCGGAGCTGAATGAAGCCATACCAAACGACGAGCGTGACACCACGATGCCTGTAGCAATGGCAACAACGTTGCGCAAACTATTAACTGGCGAACTACTTACTCTAGCTTCCCGGCAACAATTAATAGACTGGATGGAGGCGGATAAAGTTGCAGGACCACTTCTGCGCTCGGCCCTTCCGGCTGGCTGGTTTATTGCTGATAAATCTGGAGCCGGTGAGCGTGGGTCTCGCGGTATCATTGCAGCACTGGGGCCAGATGGTAAGCCCTCCCGTATCGTAGTTATCTACACGACGGGGAGTCAGGCAACTATGGATGAACGAAATAGACAGATCGCTGAGATAGGTGCCTCACTGATTAAGCATTGGTAACTGTCAGACCAAGTTTACTCATATATACTTTAGATTGATTTAAAACTTCATTTTTAATTTAAAAGGATCTAGGTGAAGATCCTTTTTGATAATCTCATGACCAAAATCCCTTAACGTGAGTTTTCGTTCCACTGAGCGTCAGACCCCGTAGAAAAGATCAAAGGATCTTCTTGAGATCCTTTTTTTCTGCGCGTAATCTGCTGCTTGCAAACAAAAAAACCACCGCTACCAGCGGTGGTTTGTTTGCCGGATCAAGAGCTACCAACTCTTTTTCCGAAGGTAACTGGCTTCAGCAGAGCGCAGATACCAAATACTGTTCTTCTAGTGTAGCCGTAGTTAGGCCACCACTTCAAGAACTCTGTAGCACCGCCTACATACCTCGCTCTGCTAATCCTGTTACCAGTGGCTGCTGCCAGTGGCGATAAGTCGTGTCTTACCGGGTTGGACTCAAGACGATAGTTACCGGATAAGGCGCAGCGGTCGGGCTGAACGGGGGGTTCGTGCACACAGCCCAGCTTGGAGCGAACGACCTACACCGAACTGAGATACCTACAGCGTGAGCTATGAGAAAGCGCCACGCTTCCCGAAGGGAGAAAGGCGGACAGGTATCCGGTAAGCGGCAGGGTCGGAACAGGAGAGCGCACGAGGGAGCTTCCAGGGGGAAACGCCTGGTATCTTTATAGTCCTGTCGGGTTTCGCCACCTCTGACTTGAGCGTCGATTTTTGTGATGCTCGTCAGGGGGGCGGAGCCTATGGAAAAACGCCAGCAACGCGGCCTTTTTACGGTTCCTGGCCTTTTGCTGGCCTTTTGCTCACATGTTCTTTCCTGCGTTATCCCCTGATTCTGTGGATAACCGTATTACCGCCTTTGAGTGAGCTGATACCGCTCGCCGCAGCCGAACGACCGAGCGCAGCGAGTCAGTGAGCGAGGAAGCGGAAGAGCGCCCA

p-ReMEDE.RFP

GACGTCGGATCTAATTCAATTAGAGACTAATTCAATTAGAGCTAATTCAATTAGGATCCAAGCTTATCGATTTCGAACCCTCGACCGCCGGAGTATAAATAGAGGCGCTTCGTCTACGGAGCGACAATTCAATTCAAACAAGCAAAGTGAACACGTCGCTAAGCGAAAGCTAAGCAAATAAACAAGCGCAGCTGAACAAGCTAAACAATCGGGCCACCATGAGGTCTTCCAAGAATGTTATCAAGGAGTTCATGAGGTTTAAGGTTCGCATGGAAGGAACGGTCAATGGGCACGAGTTTGAAATAGAAGGCGAAGGAGAGGGGAGGCCATACGAAGGCCACAATACCGTAAAGCTTAAGGTAACCAAGGGGGGACCTTTGCCATTTGCTTGGGATATTTTGTCACCACAATTTCAGTATGGAAGCAAGGTATATGTCAAGCACCCTGCCGACATACCAGACTATAAAAAGCTGTCATTTCCTGAAGGATTTAAATGGGAAAGGGTCATGAACTTTGAAGACGGTGGCGTCGTTACTGTAACCCAGGATTCCAGTTTGCAGGATGGCTGTTTCATCTACAAGGTCAAGTTCATTGGCGTGAACTTTCCTTCCGATGGACCTGTTATGCAAAAGAAGACAATGGGCTGGGAAGCCAGCACTGAGCGTTTGTATCCTCGTGATGGCGTGTTGAAAGGAGAGATTCATAAGGCTCTGAAGCTGAAAGACGGTGGTCATTACCTAGTTGAATTCAAAAGTATTTACATGGCAAAGAAGCCTGTGCAGCTACCAGGGTACTACTATGTTGACTCCAAACTGGATATAACAAGCCACAACGAAGACTATACAATCGTTGAGCAGTATGAAAGAACCGAGGGACGCCACCATCTGTTCCTTTAGCGGCCAACGTCGACTAAAGCCAAATAGAAATTATTCAGTTCTGGCTTAAGTTTTTAAAAGTGATATTATTTATTTGGTTGTAACCAACCAAAAGAATGTAAATAACTAATACATAATTATGTTAGTTTTAAGTTAGCAACAAATTGATTTTAGCTATATTAGCTACTTGGTTAATAAATAGAATATATTTATTTAAAGATAATTCGTTTTTATTGTCAGGGAGTGAGTTTGCTTAAAAACTCGTTTAAGCTTATCGATACGCGTACGGCGCGCCAAAGCTTTGTTCATAACTTCGTATAGCATACATTATACGAAGTTATgTCCGGATGGCCACGTAATAAGTGTGCGTTGAATTTATTCGCAAAAACATTGCATATTTTCGGCAAAGTAAAATTTTGTTGCATACCTTATCAAAAAATAAGTGCTGCATACTTTTTAGAGAAACCAAATAATTTTTTATTGCATACCCGTTTTTAATAAAATACATTGCATACCCTCTTTTAATAAAAAATATTGCATACTTTGACGAAACAAATTTTCGTTGCATACCCAATAAAAGATTATTATATTGCATACCCGTTTTTAATAAAATACATTGCATACCCTCTTTTAATAAAAAATATTGCATACGTTGACGAAACAAATTTTCGTTGCATACCCAATAAAAGATTATTATATTGCATACCTTTTCTTGCCATACCATTTAGCCGATCAATTGTGCTCGGCAACAGTATATTTGTGGTGTGCCAACCAACAACatgcccAACACGAAGAGCAGCAGTGTGGTGGATTTCTCAGTTATTGATTTTGCTTCTGCAAAGACAGCAAACGCAAAAGGGCAACATTTGCTGGTCGGCATGCTTTTCGGGGGAGGAGGACTGGGCGCGAGCAGGGCGAGGCATGCATTCAAAAAGCCGGCTCCAACGGAGAGCGAGAGAGCGCAAGAGCCAAGTGTACAGCAAGGCAAACAACAAACAACGAAACACGAAGGATGAGTACTTAAAACCAAACACGTGATAGTACACGGTATTAGTTGGGATCAAAGTGCACGTTTCTGTTAAGTTTTGCAACCGCTCGTTATTAAAAGCAGTTCACAAAATACTTAAAAGAAATCGAATTAGAGCCAGAACATTTTTTCAAATTATATTTTGTTATACATTTCTTTGTAAAAGGAAATTAAAATAGAAAATTCGAATGGCTTGCATATCCTAAATATGCATTTTATTATTATATGCGTTATAATACATATAATTAATATGTTATTAATATCTAACTTATTTAACTAATTACATCAATTACTGCATTACATTTAATTATTTGAAATCTATGTCCATACATTgagctcaggcctagattaCTTTTTCTTTTTTGCCTGGCCGGCCTTTTTCGTGGCCGCCGGCCTTTTGTCGCCTCCCAGCTGAGACAGGTCGATCCGTGTCTCGTACAGGCCGGTGATGCTCTGGTGGATCAGGGTGGCGTCCAGCACCTCTTTGGTGCTGGTGTACCTCTTCCGGTCGATGGTGGTGTCAAAGTACTTGAAGGCGGCAGGGGCTCCCAGATTGGTCAGGGTAAACAGGTGGATGATATTCTCGGCCTGCTCTCTGATGGGCTTATCCCGGTGCTTGTTGTAGGCGGACAGCACTTTGTCCAGATTAGCGTCGGCCAGGATCACTCTCTTGGAGAACTCGCTGATCTGCTCGATGATCTCGTCCAGGTAGTGCTTGTGCTGTTCCACAAACAGCTGTTTCTGCTCATTATCCTCGGGGGAGCCCTTCAGCTTCTCATAGTGGCTGGCCAGGTACAGGAAGTTCACATATTTGGAGGGCAGGGCCAGTTCGTTTCCCTTCTGCAGTTCGCCGGCAGAGGCCAGCATTCTCTTCCGGCCGTTTTCCAGCTCGAACAGGGAGTACTTAGGCAGCTTGATGATCAGGTCCTTTTTCACTTCTTTGTAGCCCTTGGCTTCCAGAAAGTCGATGGGATTCTTCTCGAAGCTGCTTCTTTCCATGATGGTGATCCCCAGCAGCTCTTTCACACTCTTCAGTTTCTTGGACTTGCCCTTTTCCACTTTGGCCACCACCAGCACAGAATAGGCCACGGTGGGGCTGTCGAAGCCGCCGTACTTCTTAGGGTCCCAGTCCTTCTTTCTGGCGATCAGCTTATCGCTGTTCCTCTTGGGCAGGATAGACTCTTTGCTGAAGCCGCCTGTCTGCACCTCGGTCTTTTTCACGATATTCACTTGGGGCATGCTCAGCACTTTCCGCACGGTGGCAAAATCCCGGCCCTTATCCCACACGATCTCCCCGGTTTCGCCGTTTGTCTCGATCAGAGGCCGCTTCCGGATCTCGCCGTTGGCCAGGGTAATCTCGGTCTTGAAAAAGTTCATGATGTTGCTGTAGAAGAAGTACTTGGCGGTAGCCTTGCCGATTTCCTGCTCGCTCTTGGCGATCATCTTCCGCACGTCGTACACCTTGTAGTCGCCGTACACGAACTCGCTTTCCAGCTTAGGGTACTTTTTGATCAGGGCGGTTCCCACGACGGCGTTCAGGTAGGCGTCGTGGGCGTGGTGGTAGTTGTTGATCTCGCGCACTTTGTAAAACTGGAAATCCTTCCGGAAATCGGACACCAGCTTGGACTTCAGGGTGATCACTTTCACTTCCCGGATCAGCTTGTCATTCTCGTCGTACTTAGTGTTCATCCGGGAGTCCAGGATCTGTGCCACGTGCTTTGTGATCTGCCGGGTTTCCACCAGCTGTCTCTTGATGAAGCCGGCCTTATCCAGTTCGCTCAGGCCGCCTCTCTCGGCCTTGGTCAGATTGTCGAACTTTCTCTGGGTAATCAGCTTGGCGTTCAGCAGCTGCCGCCAGTAGTTCTTCATCTTCTTCACGACCTCTTCGGAGGGCACGTTGTCGCTCTTGCCCCGGTTCTTGTCGCTTCTGGTCAGCACCTTGTTGTCGATGGAGTCGTCCTTCAGAAAGCTCTGAGGCACGATATGGTCCACATCGTAGTCGGACAGCCGGTTGATGTCCAGTTCCTGGTCCACGTACATATCCCGCCCATTCTGCAGGTAGTACAGGTACAGCTTCTCGTTCTGCAGCTGGGTGTTTTCCACGGGGTGTTCTTTCAGGATCTGGCTGCCCAGCTCTTTGATGCCCTCTTCGATCCGCTTCATTCTCTCGCGGCTGTTCTTCTGTCCCTTCTGGGTGGTCTGGTTCTCTCTGGCCATTTCGATCACGATGTTCTCGGGCTTGTGCCGGCCCATCACTTTCACGAGCTCGTCCACCACCTTCACTGTCTGCAGGATGCCCTTCTTAATGGCGGGGCTGCCGGCCAGATTGGCAATGTGCTCGTGCAGGCTATCGCCCTGGCCGGACACCTGGGCTTTCTGGATGTCCTCTTTAAAGGTCAGGCTGTCGTCGTGGATCAGCTGCATGAAGTTTCTGTTGGCGAAGCCGTCGGACTTCAGGAAATCCAGGATTGTCTTGCCGGACTGCTTGTCCCGGATGCCGTTGATCAGCTTCCGGCTCAGCCTGCCCCAGCCGGTGTATCTCCGCCGCTTCAGCTGCTTCATCACTTTGTCGTCGAACAGGTGGGCATAGGTTTTCAGCCGTTCCTCGATCATCTCTCTGTCCTCAAACAGTGTCAGGGTCAGCACGATATCTTCCAGAATGTCCTCGTTTTCCTCATTGTCCAGGAAGTCCTTGTCCTTGATAATTTTCAGCAGATCGTGGTATGTGCCCAGGGAGGCGTTGAACCGATCTTCCACGCCGGAGATTTCCACGGAGTCGAAGCACTCGATTTTCTTGAAGTAGTCCTCTTTCAGCTGCTTCACGGTCACTTTCCGGTTGGTCTTGAACAGCAGGTCCACGATGGCCTTTTTCTGCTCGCCGCTCAGGAAGGCGGGCTTTCTCATTCCCTCGGTCACGTATTTCACTTTGGTCAGCTCGTTATACACGGTGAAGTACTCGTACAGCAGGCTGTGCTTGGGCAGCACCTTCTCGTTGGGCAGGTTCTTATCGAAGTTGGTCATCCGCTCGATGAAGCTCTGGGCGGAAGCGCCCTTGTCCACCACTTCCTCGAAGTTCCAGGGGGTGATGGTTTCCTCGCTCTTTCTGGTCATCCAGGCGAATCTGCTGTTTCCCCTGGCCAGAGGGCCCACGTAGTAGGGGATGCGGAAGGTCAGGATCTTCTCGATCTTTTCCCGGTTGTCCTTCAGGAATGGGTAAAAATCTTCCTGCCGCCGCAGAATGGCGTGCAGCTCTCCCAGGTGGATCTGGTGGGGGATGCTGCCGTTGTCGAAGGTCCGCTGCTTCCGCAGCAGGTCCTCTCTGTTCAGCTTCACGAGCAGTTCCTCGGTGCCGTCCATCTTTTCCAGGATGGGCTTGATGAACTTGTAGAACTCTTCCTGGCTGGCTCCGCCGTCAATGTAGCCGGCGTAGCCGTTCTTGCTCTGGTCGAAGAAAATCTCTTTGTACTTCTCAGGCAGCTGCTGCCGCACGAGAGCTTTCAGCAGGGTCAGGTCCTGGTGGTGCTCGTCGTATCTCTTGATCATAGAGGCGCTCAGGGGGGCCTTGGTGATCTCGGTGTTCACTCTCAGGATGTCGCTCAGCAGGATGGCGTCGGACAGGTTCTTGGCGGCCAGAAACAGGTCGGCGTACTGGTCGCCGATCTGGGCCAGCAGGTTGTCCAGGTCGTCGTCGTAGGTGTCCTTGCTCAGCTGCAGTTTGGCATCCTCGGCCAGGTCGAAGTTGCTCTTGAAGTTGGGGGTCAGGCCCAGGCTCAGGGCAATCAGGTTGCCGAACAGGCCATTCTTCTTCTCGCCGGGCAGCTGGGCGATCAGATTTTCCAGCCGTCTGCTCTTGCTCAGTCTGGCAGACAGGATGGCCTTGGCGTCCACGCCGCTGGCGTTGATGGGGTTTTCCTCGAACAGCTGGTTGTAGGTCTGCACCAGCTGGATGAACAGCTTGTCCACGTCGCTGTTGTCGGGGTTCAGGTCGCCCTCGATCAGGAAGTGGCCCCGGAACTTGATCATGTGGGCCAGGGCCAGATAGATCAGCCGCAGGTCGGCCTTGTCGGTGCTGTCCACCAGTTTCTTTCTCAGGTGGTAGATGGTGGGGTACTTCTCGTGGTAGGCCACCTCGTCCACGATGTTGCCGAAGATGGGGTGCCGCTCGTGCTTCTTATCCTCTTCCACCAGGAAGGACTCTTCCAGTCTGTGGAAGAAGCTGTCGTCCACCTTGGCCATCTCGTTGCTGAAGATCTCTTGCAGATAGCAGATCCGGTTCTTCCGTCTGGTGTATCTTCTTCTGGCGGTTCTCTTCAGCCGGGTGGCCTCGGCTGTTTCGCCGCTGTCGAACAGCAGGGCTCCGATCAGGTTCTTCTTGATGCTGTGCCGGTCGGTGTTGCCCAGCACCTTGAATTTCTTGCTGGGCACCTTGTACTCGTCGGTGATCACGGCCCAGCCCACAGAGTTGGTGCCGATGTCCAGGCCGATGCTGTACTTCTTGTCGGCTGCTGGGACTCCGTGGATACCGACCTTCCGCTTCTTCTTTGGGGCCATCTTATCGTCATCGTCTTTGTAATCAATATCATGATCCTTGTAGTCTCCGTCGTGGTCCTTATAGTCCATtctaggcgcgccagatctggtaccgATTGATATTTTTTTTTTAATTTGGCCTGCCTTTCAAGAGCAAATTAATTCTTTTTCACTTTATGTATTACCTTACAAACTTTGATAAGTTCAGATCACTTCGCGCAACGCAAAACCGAAAAGGATCTGTTAAAAGTACCAAAAATCTAGTGGTGGAATTTCCCATTGTGCTATCGCAATTCGATATTCACAATTTCGATAATTTATATATATAGCCGTTTTCCACTAACACATCAAATGTGATTTAGCTATTAATGAAATTGTAATATTCAAGCAACCTAAGTGATTCATTCATATGTACATACTTATATGTAAAGATGGTGGTGGCATTTTTAACATGGGGAAGTTCTTATTTATTTACTTTTTCGTTTGTTATAGGTGAATTCCTCTTTATTTTTATACAATATAATTTTATTTTACGTTAAGCAACCAATTTAAGGGCTATGAAACGTAGTAACTAACAGGAGCAACGAAGATAACATGGAAACATACAAAATTTGTATAAAAATATTGATTAGATAATAGTTTGGCTTGTTTATGCCACGTGTCCAAAGATATCTTTGATGACGCCTCCTCAGTGTTCTCCTGGTTGTCTGGTATGTGCCTGATTTAATTTGTTCTCAAACTGAAGGTGGATGCTGAGGTCATGTGCGATATCAACATTTCTCGCGTACCAGGGCGCGTTGGTGATTGTTCGCAACGCTTGGTTTTGGAGCCGTTGCCCTCTCACTATGTCCCACCGCCGATTTGACCCGCAGACGTATCCCGCTTGTCCCCAACAACCGCACTTCATCATAATACATCATCTCTAGACCACACTCAAACGATTTATTTATTAAAACTAAGTGATATATAGGGAATTGTTATTGAAGGTAATTAGTATAATTCAAAAACGCCCAACACGCTGTTCTTTCATTGAAATACAGTGGAGCCCTGCTTGCAAGCAGTGCAAGTTTAAAAAACATCGCAATGCTATGTGTGTGTACTCGCTTAAAAGCCAGATGTACATTAAAATACGGTAACACTGTTGACTTCTGAAACCCAAACTACTTCCAGTTGGGAATTGTATTAGCCATGGGAAGGCATTTCTTGACAAGCCACCGCCCGGTAACACTAAAACGCGTTAGGGTGTTCTGTCAACTCGTTCTGCGAATACAGAAACGAAAAAAATAGTAAACAACATTTATTGGAGGGAAAAAAAGAAATAGCCCGGCTTCCGCATAAACAACTGTCGCAGACCCGCAGCACCGATTAAAGCCGACAGCCGACGCCAGTTAACCTAACCTCTGATCAAATTAAGTGGGCCAAAATGAGCGTGGAGGAGGAAGTGTTTCGAATCCAAAAGAAGATGAGCAAGATGGCCAGCGACGGCACAGTAAGTATCGGGATTAGCTAACGTGCCGCGCATCATGTCACAAATATACATATATCCACCGTACAGGGACAGGATCAGGCTCTGGACCTGCTGAAGGCCCTGCAAACGCTTAACATCAATCTCGACATTCTGACCAAAACGCGCATCGGCATGACCGTAAACGAGCTGCGCAAGAGTAGCAAGGACGACGAGGTGATCGCTCTGGCCAAGACGCTGATCAAGAACTGGAAACGATTCCTCGCCAGCCCGGCGCCAACCACACCGAACAACAGCTCCGCTAAGGAGGGCTCCTCCAACAACAGCAGTGCCTCGAAGTCCACCAGTGCCGCCAAGTCGTCGTCGTCAATCTCCGGCAAGGATAAGTCCAGTTCATCCAGCTCATCCAAGGATAAAGAGAAGAAAGGCTCGACATCCTCGTCACAGACCTCGTTTCCCTCCGGCGGCATGACAGATGCGGTCCGCATCAAATGCCGCGAAATGCTGGCCACCGCACTGAAGATTGGTGAAGTGCCCGAAGGATGCGGTGAGCCGGAGGAAATGGCCGCCGAACTGGAGGATGCCATTTACTCCGAGTTCAATAACACGGATATGAAGTACAAGAATCGCATTAGGTCACGCGTGGCCAATCTGAAGGACCCCAAGAATCCTGGATTGCGCGGCAACTTTATGTGCGGCGCCGTCACTGCCAAGCAGCTGGCCAAAATGACGCCGGAGGAGATGGCCAGCGACGAGATGAAGAAGCTGCGCGAGAAGTTCGTCAAGGAGGCCATCAACGATGCCCAGCTGGCCACCGTGCAGGGCACCAAGACCGATCTCCTCAAGTGCGCCAAGTGCAAGAAGCGCAACTGCACCTACAACCAGCTGCAGGGATCCTTTTTTGCTCACCTGTGATTGCTCCTACTCAAATACAAAAACATCAAATTTTCTGTCAATAAAGCATATTTATTTATATTTATTTTACAGGAAAGAATTCCTTTTAAAGTGTATTTTAACCTATAATGAAAAACGATTAAAAAAAATACATAAAATAATTCGAAAATTTTTGAATAGCCCAGGTTGATAAAAATTCATTTCATACGTTTTATAACTTATGCCCCTAAGTATTTTTTGACCATAGTGTTTCAATTCTACATTAATTTTACAGAGTAGAATGAAACGCCACCTACTCAGCCAAGAGGCGAAAAGGTTAGCTCGCCAAGCAGAGAGGGCGCCAGTGCTCACTACTTTTTATAATTCTCAACTTCTTTTTCCAGACTCAGTTCGTATATATAGACCTATTTTCAATTTAACGTCGGTTTTGGACACTGGAACCGGTTTTAGAGCTAGAAATAGCAAGTTAAAATAAGGCTAGTCCGTTATCAACTTGAAAAAGTGGCACCGAGTCGGTGCgggcatgcgTGGCCACGTAATAAGTGTGCGTTGAATTTATTCGCAAAAACATTGCATATTTTCGGCAAAGTAAAATTTTGTTGCATACCTTATCAAAAAATAAGTGCTGCATACTTTTTAGAGAAACCAAATAATTTTTTATTGCATACCCGTTTTTAATAAAATACATTGCATACCCTCTTTTAATAAAAAATATTGCATACTTTGACGAAACAAATTTTCGTTGCATACCCAATAAAAGATTATTATATTGCATACCCGTTTTTAATAAAATACATTGCATACCCTCTTTTAATAAAAAATATTGCATACGTTGACGAAACAAATTTTCGTTGCATACCCAATAAAAGATTATTATATTGCATACCTTTTCTTGCCATACCATTTAGCCGATCAATTGTGCTCGGCAACAGTATATTTGTGGTGTGCCAACCAACAACCCTAGGatctgCGGAGTACTGTCCTCCGAGCGGAGTACTGTCCTCCGAGCGGAGTACTGTCCTCCGAGCGGAGTACTGTCCTCCGAGCGGAGTACTGTCCTCCGAGCGGAGACTCTAGCGAGCGAGCCGTAGCTTACCGAAGTATACACTTAAATTCAGTGCACGTTTGCTTGTTGAGAGGAAAGGTTGTGTGCGGACGAATTTTTTTTTGAAAACCGGTGATAGAGCCTGAACCAGAAAAGATAAAAGAAGGCTATACCAGTGGGAGTACACAAACAGAGTAAGTTTGAATAGTAAAAAAAATCATTTATGTAAACAATAACGTGACTGTGCGTTAGGTCCTGTTCATTGGTACCCGCCCGGGGATCATCTGTTCTAGAATGAAAAACATCAAAAAAAACCAGGTAATGAACCTGGGTCCGAACTCTAAACTGCTGAAAGAATACAAATCCCAGCTGATCGAACTGAACATCGAACAGTTCGAAGCAGGTATCGGTCTGATCCTGGGTGATGCTTACATCCGTTCTCGTGATGAAGGTAAAACCTACTGTATGCAGTTCGAGTGGAAAAACAAAGCATACATGGACCACGTATGTCTGCTGTACGATCAGTGGGTACTGTCCCCGCCGCACAAAAAAGAACGTGTTAACCACCTGGGTAACCTGGTAATCACCTGGGGCGCCCAGACTTTCAAACACCAAGCTTTCAACAAACTGGCTAACCTGTTCATCGTTAACAACAAAAAAACCATCCCGAACAACCTGGTTGAAAACTACCTGACCCCGATGTCTCTGGCATACTGGTTCATGGATGATGGTGGTAAATGGGATTACAACAAAAACTCTACCAACAAATCGATCGTACTGAACACCCAGTCTTTCACTTTCGAAGAAGTAGAATACCTGGTTAAGGGTCTGCGTAACAAATTCCAACTGAACTGTTACGTAAAAATCAACAAAAACAAACCGATCATCTACATCGATTCTATGTCTTACCTGATCTTCTACAACCTGATCAAACCGTACCTGATCCCGCAGATGATGTACAAACTGCCGAACACTATCTCCTCCGAAACTTTCCTGAAACCAAAAAAGAAGAGAAAGGTATAAGAAGACCCCAAGGACTTTCCTTCAGAATTGCTAAGTTTTTTGAGTCATGCTGTGTTTAGTAATAGAACTCTTGCTTGCTTTGCTATTTACACCACAAAGGAAAAAGCTGCACTGCTATACAAGAAAATTATGGAAAAATATTTGATGTATAGTGCCTTGACTAGAGATCATAATCAGCCATACCACATTTGTAGAGGTTTTACTTGCTTTAAAAAACCTCCCACACCTCCCCCTGAACCTGAAACATAAAATGAATGCAATTGTTGTTGTTAACTTGTTTATTGCAGCTTATAATGGTTACAAATAAAGCAATAGCATCACAAATTTCACAAATAAAGCATTTTTTTCACTGCATTCTAGTTGTGGTTTGTCCAAACTCATCAATGTATCTTATCATGTCTGGATCgcgcttattgattggcTGGTCCATCATGATGAACGGGTCGAGGTGGCGGTAGTTGATCCCGGCGAACGCGCGGCGCACCGGGAAGCCCTCGCCCTCGAAACCGCTGGGCGCGGTGGTCACGGTGAGCACGGGACGTGCGACGGCGTCGGCTGGTGCGGATACGCGGGGCAGCGTCAGCGGGTTCTCGACGGTCACGGCGGGCATGTCGACAAGCCGAATTGATCCACTAGAAGGCCTAATTCGtCTAGCAGGATCTctaggGCTTCGACCGTTTTAACCTCGAAATATGCACATGTAAGGACGGATGTGAGCGAACGCCAGTGATGACCGGGATCAGAGGTAACCTACCATGGTGGGGATTAGGTGACCGTTCGCAGGTAGTTTGATCGGAGCGAATGTTCGGGGGGTCTGGCGTCAGAGGCTCTAAACTTTATGTAATTCCTGCCGCGAAACACGCACGTATCAAGCAGTCAGCTGTTCTCTTCGTTCAGCGCGCGCCGGTGTTGCAAAACGAGCGCTCTTCGCCGGCGGTGGCTCGTGCGATAGTTCGTTTTGTCGGTAATCCGATGTTGCCGCGCCGATATCATGTGATGTTGTCACAGTGCGCGAAATTCGAATGGTGGTGTGCAGTGATTGTGTTGTGACGGCGAGTGGCGCGTGTGGGTGCTTAGTTTTGGGAGATGTTTTCGTATTTTTTTGTTGATAACTCAGGCTTTGTTGCTGTGTTGTAGTACTATTTTCCATTGCGCGGTGTCCAGCTTTTAATTAGTGGCACATATTCTTAGCAAGTAAAAATTATTTTGCATACTATTAAATTTCTTATAAATTATTTTCTAAAATTAAGTTTACCTTTTCAATTTTACTAAAAATATCGATATATTTATTATCGCTGGAAAACTACATTATTCCACCTCTAAGCAAGAACCGTTAGTTGGCGCGTAGCTTTACCACAAAATTCCTGGAATTGCCGTACGCTTCGCAGTTGTTTCAAGTTGTCTAAGGGACATACGATTTTTTTTGCCTCTGCGTCACGATTTTAACCCAAAAGCGAGTTTAGTTACATGTACATTATTATTAGATAAAGAAGTATCGCGAATACTTCAGTTGAATAAACTGTGCTTGGTTTTTGGGTGAGGATTTGTGGAAAGTAGAGTGCGCGATAACCGTAACTTTCGACCCGGATTTTCGCCtgAATTgagatctcTCTAGAggtacCGCCACCATGGCCCCAAAGAAGAAGCGGAAGGTCGGTATCCACGGTGTCCCAGCAGCCcaaATGGACTCCCAGCAGCCAGATCTGAAGCTACTGTCTTCTATCGAACAAGCATGCGATATTTGCCGACTTAAAAAGCTCAAGTGCTCCAAAGAAAAACCGAAGTGCGCCAAGTGTCTGAAGAACAACTGGGAGTGTCGCTACTCTCCCAAAACCAAAAGGTCTCCGCTGACTAGGGCACATCTGACAGAAGTGGAATCAAGGCTAGAAAGACTGGAACAGCTATTTCTACTGATTTTTCCTCGAGAAGACCTTGACATGATTTTGAAAATGGATTCTTTACAGGATATAAAAGCATTGTTAGAATTCCCGGGTGTCGACCAGAAAAAGTTCAATAAAGTCAGAGTTGTGAGAGCACTGGATGCTGTTGCTCTCCCACAGCCAGTGGGCGTTCCAAATGAAAGCCAAGCCCTAAGCCAGAGATTCACTTTTTCACCAGGTCAAGACATACAGTTGATTCCACCACTGATCAACCTGTTAATGAGCATTGAACCAGATGTGATCTATGCAGGACATGACAACACAAAACCTGACACCTCCAGTTCTTTGCTGACAAGTCTTAATCAACTAGGCGAGAGGCAACTTCTTTCAGTAGTCAAGTGGTCTAAATCATTGCCAGGTTTTCGAAACTTACATATTGATGACCAGATAACTCTCATTCAGTATTCTTGGATGAGCTTAATGGTGTTTGGTCTAGGATGGAGATCCTACAAACACGTCAGTGGGCAGATGCTGTATTTTGCACCTGATCTAATACTAAATGAACAGCGGATGAAAGAATCATCATTCTATTCATTATGCCTTACCATGTGGCAGATCCCACAGGAGTTTGTCAAGCTTCAAGTTAGCCAAGAAGAGTTCCTCTGTATGAAAGTATTGTTACTTCTTAATACAATTCCTTTGGAAGGGCTACGAAGTCAAACCCAGTTTGAGGAGATGAGGTCAAGCTACATTAGAGAGCTCATCAAGGCAATTGGTTTGAGGCAAAAAGGAGTTGTGTCGAGCTCACAGCGTTTCTATCAACTTACAAAACTTCTTGATAACTTGCATGATCTTGTCAAACAACTTCATCTGTACTGCTTGAATACATTTATCCAGTCCCGGGCACTGAGTGTTGAATTTCCAGAAATGATGTCTGAAGTTATTGCTGGGTCGACGAGATATCAAGCAGAATTCCAGTACCTGCCAGATACAGACGATCGTCACCGGATTGAGGAGAAACGTAAAAGGACATATGAGACCTTCAAGAGCATCATGAAGAAGAGTCCTTTCAGCGGACCCACCGACCCCCGGCCTCCACCTCGACGCATTGCTGTGCCTTCCCGCAGCTCAGCTTCTGTCCCCAAGCCAGCACCCCAGCCCTATCCCTTTACGTCATCCCTGAGCACCATCAACTATGATGAGTTTCCCACCATGGTGTTTCCTTCTGGGCAGATCAGCCAGGCCTCGGCCTTGGCCCCGGCCCCTCCCCAAGTCCTGCCCCAGGCTCCAGCCCCTGCCCCTGCTCCAGCCATGGTATCAGCTCTGGCCCAGGCCCCAGCCCCTGTCCCAGTCCTAGCCCCAGGCCCTCCTCAGGCTGTGGCCCCACCTGCCCCCAAGCCCACCCAGGCTGGGGAAGGAACGCTGTCAGAGGCCCTGCTGCAGCTGCAGTTTGATGATGAAGACCTGGGGGCCTTGCTTGGCAACAGCACAGACCCAGCTGTGTTCACAGACCTGGCATCCGTCGACAACTCCGAGTTTCAGCAGCTGCTGAACCAGGGCATACCTGTGGCCCCCCACACAACTGAGCCCATGCTGATGGAGTACCCTGAGGCTATAACTCGCCTAGTGACAGGGGCCCAGAGGCCCCCCGACCCAGCTCCTGCTCCACTGGGGGCCCCGGGGCTCCCCAATGGCCTCCTTTCAGGAGATGAAGACTTCTCCTCCATTGCGGACATGGACTTCTCAGCCCTGCTGAGTCAGATCAGCTCCTAATctagcacctaggaggatccactcgAGGTTTAGAGAGGGCGAATCCAGCTCTGGAGCAGAGGCTCTGGCAGCTTTTGCAGCGTTTATATAACATGAAATATATATACGCATTCCGATCAAAGCTGGGTTAACCAGATAGATAGATAGTAACGTTTAAATAGCGCCTGGCGCGTTCGATTTTAAAGAGATTTAGAGCGTTATCCCGTGCCTATAGATCaTATAGTATAGACAACGAACGATCACTCAAATCCAAGTCAATAATTCAAGAATTTATGTCTGTTTCTGTGAAAGGGAAACTAATTTTGTTAAAGAAGACTTACAATATCGTAATACTTGTTCAATCGTCGTGGCCGATAGAAATATCTTACAATCCGAAAGTTGATGAATGGAATTGGTCTGCAACTGGTCGCCTTCATTTCGTAAAATGTTCGCTTGCGGCCGAAAAATTTCGATATATCTACAATTcATCTACAATCTTTACTAAATTTTGAAAAAGGAACACTTTGAATTTCGAACTGTCAATCGTATCATTAGAATTTAATCTAAATTTAAATCTTGCTAAAGGAAATAGCAAGGAACACTTTCGTCGTCGGCTACGCATTCATTGTAAAATTTTAAATTTTGACATTCCGCACTTTTTGATAGATAAGCGAAGAGTATTTTTATTACATGTATCGCAAGTATTCATTTCAACACACATATCTATATATATATATATATATATATATATATATATATATATATATGTTATATATTTATTCAATTTTGTTTACCATTGATCAATTTTTCACACATGAAACAACCGCCAGCATTATATAATTTTTTTATTTTTTTAAAAAATGTGTACACATATTCTGAAAATGAAAAATTCAATGGCTCGAcTGCCAAATAAAGAAATGGTTACAATTTAAGGAAACAAATGTCCTTCTTGCACTAGGGGGGTGACATCCCTAGGTCCGGAGAATgtttaaacaGAAGTTCCTATTCCGAAGTTCCTATTCTCTAGAAAGTATAGGAACTTCtcgtagtgccccaactggggtaacctttgagttctctcagttgggggcgtagCGGTCTCGAAGCCGCGGTGCGGGTGCCAGGGCGTGCCCTTGGGCTCCCCGGGCGCGTACTCCACCTCACCCATCTGGTCCAcctgcaggGACGTCAGGTGGCACTTTTCGGGGAAATGTGCGCGGAACCCCTATTTGTTTATTTTTCTAAATACATTCAAATATGTATCCGCTCATGAGACAATAACCCTGATAAATGCTTCAATAATATTGAAAAAGGAAGAGTATGAGTATTCAACATTTCCGTGTCGCCCTTATTCCCTTTTTTGCGGCATTTTGCCTTCCTGTTTTTGCTCACCCAGAAACGCTGGTGAAAGTAAAAGATGCTGAAGATCAGTTGGGTGCACGAGTGGGTTACATCGAACTGGATCTCAACAGCGGTAAGATCCTTGAGAGTTTTCGCCCCGAAGAACGTTTTCCAATGATGAGCACTTTTAAAGTTCTGCTATGTGGCGCGGTATTATCCCGTATTGACGCCGGGCAAGAGCAACTCGGTCGCCGCATACACTATTCTCAGAATGACTTGGTTGAGTACTCACCAGTCACAGAAAAGCATCTTACGGATGGCATGACAGTAAGAGAATTATGCAGTGCTGCCATAACCATGAGTGATAACACTGCGGCCAACTTACTTCTGACAACGATCGGAGGACCGAAGGAGCTAACCGCTTTTTTGCACAACATGGGGGATCATGTAACTCGCCTTGATCGTTGGGAACCGGAGCTGAATGAAGCCATACCAAACGACGAGCGTGACACCACGATGCCTGTAGCAATGGCAACAACGTTGCGCAAACTATTAACTGGCGAACTACTTACTCTAGCTTCCCGGCAACAATTAATAGACTGGATGGAGGCGGATAAAGTTGCAGGACCACTTCTGCGCTCGGCCCTTCCGGCTGGCTGGTTTATTGCTGATAAATCTGGAGCCGGTGAGCGTGGGTCTCGCGGTATCATTGCAGCACTGGGGCCAGATGGTAAGCCCTCCCGTATCGTAGTTATCTACACGACGGGGAGTCAGGCAACTATGGATGAACGAAATAGACAGATCGCTGAGATAGGTGCCTCACTGATTAAGCATTGGTAACTGTCAGACCAAGTTTACTCATATATACTTTAGATTGATTTAAAACTTCATTTTTAATTTAAAAGGATCTAGGTGAAGATCCTTTTTGATAATCTCATGACCAAAATCCCTTAACGTGAGTTTTCGTTCCACTGAGCGTCAGACCCCGTAGAAAAGATCAAAGGATCTTCTTGAGATCCTTTTTTTCTGCGCGTAATCTGCTGCTTGCAAACAAAAAAACCACCGCTACCAGCGGTGGTTTGTTTGCCGGATCAAGAGCTACCAACTCTTTTTCCGAAGGTAACTGGCTTCAGCAGAGCGCAGATACCAAATACTGTTCTTCTAGTGTAGCCGTAGTTAGGCCACCACTTCAAGAACTCTGTAGCACCGCCTACATACCTCGCTCTGCTAATCCTGTTACCAGTGGCTGCTGCCAGTGGCGATAAGTCGTGTCTTACCGGGTTGGACTCAAGACGATAGTTACCGGATAAGGCGCAGCGGTCGGGCTGAACGGGGGGTTCGTGCACACAGCCCAGCTTGGAGCGAACGACCTACACCGAACTGAGATACCTACAGCGTGAGCTATGAGAAAGCGCCACGCTTCCCGAAGGGAGAAAGGCGGACAGGTATCCGGTAAGCGGCAGGGTCGGAACAGGAGAGCGCACGAGGGAGCTTCCAGGGGGAAACGCCTGGTATCTTTATAGTCCTGTCGGGTTTCGCCACCTCTGACTTGAGCGTCGATTTTTGTGATGCTCGTCAGGGGGGCGGAGCCTATGGAAAAACGCCAGCAACGCGGCCTTTTTACGGTTCCTGGCCTTTTGCTGGCCTTTTGCTCACATGTTCTTTCCGATATCATTGG
