# Supplementary material for: Repeat mediated excision of gene drive elements for restoring wild-type populations
Source: PLoS Genet. 2024 Nov 7;20(11):e1011450. doi: 10.1371/journal.pgen.1011450 (PMC11584131; doi:10.1371/journal.pgen.1011450)
Supplement: S1 Text — (DOCX) [file pgen.1011450.s010.docx]

## **S1 Text**

### **Courtship Assays**

A construct containing EGFP under the control of a 3xP3 promoter was inserted into exon 2 of the *D. melanogaster yellow* gene at the drive element (yMCR or yReMEDE) target site, generating a null mutation. The courtship behavior of yellow flies was then assessed in both noncompetitive and competitive mating crosses with wild-type (*w^1118^*) flies conducted in specially designed fly arenas, as described previously [1]. Briefly, female and male flies were gently aspirated into the circular arenas of the apparatus. Mating behavior was then captured by video over a five-hour duration. Any copulation between a male and female fly observed during the video analysis was subsequently scored as a successful mating. Courtship studies were conducted at room temperature over a similar time range during the day (12 hour light/dark cycle) with a standard corn-meal/oatmeal based medium.

### **Population Studies**

All multi-generational cage trials were conducted at 25 C (12 hour light/dark cycle) in standard 250 mL rearing bottles containing a standard corn-meal/oatmeal based medium. For each genotype, bottles were seeded with equal numbers of unmated male and female flies. After 5 days, this initial generation (n) of flies was removed from the bottles. Subsequent generations (n+1) were then collected and randomly divided into two equal pools. One pool was seeded into a fresh cage (n+2), while the other was scored and further analyzed.

### **Modeling**

Mathematical modeling was performed with methods that have been described previously [2], but with the following modifications. In this study, the continuous addition of offspring into the adult mating pool was replaced with distinct generations. A modified version of the widely used lumped age-class model of mosquito life-history [3–5] was incorporated to more fully account for the fly’s four different states: egg, larvae, pupae, and adult (male and female). In order to adapt the model to *Drosophila,* the following parameters were assigned to the fly’s life-history stages. Egg production per day (day[-1]) = 100; duration of egg stage (days) =1; duration of larval stage (days) = 4; duration of pupa stage (days) = 5 [6–8]; daily mortality risk of egg stage (day[-1]) = 0.15; daily mortality risk of pupa stage (day[-1]) = 0.15 [9–11]; daily mortality risk of adult stage (day[-1]) = 0.263; and daily population growth rate (day[-1]) = 1.196 [12]. We also developed a stochastic version of the previously described methods where genotype-specific daily egg production follows a multivariate Poisson distribution informed by parental genotypes and the “Equation Generation” module. Survival and death events follow binomial distributions at the population level.

Equation Generation

Equations for determining the number of offspring with each genotype were derived as described previously [2], but with the following modifications. An additional factor of epsilon (ε) was incorporated into the equations to capture the rate of ‘v’ persistence. Where ‘v’ represents a wild-type allele resulting from the excision of a previously inserted drive element, but engineered to contain a Cas9 target site that is resistant to re-cutting. The formula 1-ε was then used to represent a ‘v’ allele that has been converted into the original wild-type allele (w) sequence by mismatch repair. In order to simulate the X-linked inheritance of the ReMEDE drive construct, the probability equations for the heterogametic males were simplified to include a single allele. All possible combinations of the two female alleles and one male allele were diagramed and probabilities computed for each.

Model Accessibility

Details regarding the execution of modeling methods and interpretation of results have been provided previously [2]. All files pertaining to the generation of equations, model execution, and plotting of charts can be found at the following link: <https://github.com/mln27/Python-CRISPR1-1-SEM-gene-drive>

## **Reference:**

1. Boutros CL, Miner LE, Mazor O, Zhang SX. Measuring and Altering Mating Drive in Male Drosophila melanogaster. JoVE J Vis Exp. 2017; e55291. doi:10.3791/55291

2. Chae K, Dawson C, Valentin C, Contreras B, Zapletal J, Myles KM, et al. Engineering a self-eliminating transgene in the yellow fever mosquito, Aedes aegypti. PNAS Nexus. 2022;1: pgac037. doi:10.1093/pnasnexus/pgac037

3. Sánchez C. HM, Wu SL, Bennett JB, Marshall JM. MGDrivE: A modular simulation framework for the spread of gene drives through spatially explicit mosquito populations. Methods Ecol Evol. 2020;11: 229–239. doi:10.1111/2041-210X.13318

4. Hancock PA, Godfray HCJ. Application of the lumped age-class technique to studying the dynamics of malaria-mosquito-human interactions. Malar J. 2007;6: 98. doi:10.1186/1475-2875-6-98

5. Deredec A, Godfray HCJ, Burt A. Requirements for effective malaria control with homing endonuclease genes. Proc Natl Acad Sci U S A. 2011;108: E874-880. doi:10.1073/pnas.1110717108

6. Fernández-Moreno MA, Farr CL, Kaguni LS, Garesse R. Drosophila melanogaster as a model system to study mitochondrial biology. Methods Mol Biol Clifton NJ. 2007;372: 33–49. doi:10.1007/978-1-59745-365-3_3

7. Ong C, Yung L-YL, Cai Y, Bay B-H, Baeg G-H. Drosophila melanogaster as a model organism to study nanotoxicity. Nanotoxicology. 2015;9: 396–403. doi:10.3109/17435390.2014.940405

8. Mołoń M, Dampc J, Kula-Maximenko M, Zebrowski J, Mołoń A, Dobler R, et al. Effects of Temperature on Lifespan of Drosophila melanogaster from Different Genetic Backgrounds: Links between Metabolic Rate and Longevity. Insects. 2020;11: 470. doi:10.3390/insects11080470

9. Durdevic Z, Pillai RS, Ephrussi A. Transposon silencing in the Drosophila female germline is essential for genome stability in progeny embryos. Life Sci Alliance. 2018;1. doi:10.26508/lsa.201800179

10. Shropshire JD, On J, Layton EM, Zhou H, Bordenstein SR. One prophage WO gene rescues cytoplasmic incompatibility in Drosophila melanogaster. Proc Natl Acad Sci U S A. 2018;115: 4987–4991. doi:10.1073/pnas.1800650115

11. Layton EM, On J, Perlmutter JI, Bordenstein SR, Shropshire JD. Paternal Grandmother Age Affects the Strength of Wolbachia-Induced Cytoplasmic Incompatibility in Drosophila melanogaster. mBio. 2019;10: e01879. doi:10.1128/mBio.01879-19

12. Horváth B, Betancourt AJ, Kalinka AT. A novel method for quantifying the rate of embryogenesis uncovers considerable genetic variation for the duration of embryonic development in Drosophila melanogaster. BMC Evol Biol. 2016;16: 200. doi:10.1186/s12862-016-0776-z
