# Supplementary material for: Repeat mediated excision of gene drive elements for restoring wild-type populations
Source: PLoS Genet. 2024 Nov 7;20(11):e1011450. doi: 10.1371/journal.pgen.1011450 (PMC11584131; doi:10.1371/journal.pgen.1011450)
Supplement: Attachment — Submitted filename: PGENETICS-D-23-01355_REV.pdf [file pgen.1011450.s012.pdf]

PLOS Genetics

Repeat mediated excision of gene drive elements for restoring wild-type populations
--Manuscript Draft--

**Manuscript Number:**	PGENETICS-D-23-01355
**Full Title:**	Repeat mediated excision of gene drive elements for restoring wild-type populations
**Short Title:**	Repeat mediated excision of gene drives
**Article Type:**	Research Article
**Section/Category:**	General
**Keywords:**	Gene drive, Single Strand Annealing, SSA, DNA Repair, Drosophila, CRISPR, Cas9, Homologous Recombination, HR, Direct Repeats, autonomous, homing, double-strand break
**Abstract:**	We demonstrate here that single strand annealing (SSA) repair can be co-opted for the precise autocatalytic excision of a drive element. Although SSA is not the predominant form of DNA repair in eukaryotic organisms, we increased the likelihood of its use by engineering direct repeats at sites flanking the drive allele, and then introducing a double-strand DNA break (DSB) at a second endonuclease target site encoded within the drive allele. We have termed this technology Repeat Mediated Excision of a Drive Element (ReMEDE). Incorporation of ReMEDE into the previously described mutagenic chain reaction (MCR) gene drive, targeting the yellow gene of Drosophila melanogaster, replaced drive alleles with wild-type alleles demonstrating proof-of-principle. Although the ReMEDE system requires further research and development, the technology has a number of attractive features as a gene drive mitigation strategy, chief among these the potential to restore a wild-type population without releasing additional transgenic organisms or large-scale environmental engineering efforts.
**Additional Information:**	
**Question**	**Response**
**Government Employee**<br><br>Are you or any of the contributing authors an employee of the United States government?<br><br>Manuscripts authored by one or more US Government employees are not copyrighted, but are licensed under a CC0 Public Domain Dedication, which allows unlimited distribution and reuse of the article for any lawful purpose. This is a legal requirement for US Government employees.<br><br>This will be typeset if the manuscript is accepted for publication.	No - No authors are employees of the U.S. government.
**Financial Disclosure**<br><br>Enter a financial disclosure statement that describes the sources of funding for the	Yes

work included in this submission. Review the submission guidelines for detailed requirements. View published research articles from *PLOS Genetics* for specific examples.<br><br>This statement is required for submission and **will appear in the published article** if the submission is accepted. Please make sure it is accurate.<br><br>**Funded studies**<br>Enter a statement with the following details:<br>• Initials of the authors who received each award<br>• Grant numbers awarded to each author<br>• The full name of each funder<br>• URL of each funder website<br>• Did the sponsors or funders play any role in the study design, data collection and analysis, decision to publish, or preparation of the manuscript?<br><br>Did you receive funding for this work?	
Please add funding details.<br>as follow-up to "**Financial Disclosure**<br><br>Enter a financial disclosure statement that describes the sources of funding for the work included in this submission. Review the submission guidelines for detailed requirements. View published research articles from *PLOS Genetics* for specific examples.<br><br>This statement is required for submission and **will appear in the published article** if the submission is accepted. Please make sure it is accurate.	KMM, ZNA; AI119081; National Institute for Allergy and Infectious Diseases, NIH (https://www.niaid.nih.gov)<br>KMM, ZNA AI148787; National Institute for Allergy and Infectious Diseases, NIH (https://www.niaid.nih.gov)<br>KMM, ZNA; HR0011-16-2-0036; Defense Advanced Research Projects Agency, DOD (https://www.darpa.mil)<br>The funders had no role in study design, data collection and analysis, decision to publish, or preparation of the manuscript.

**Funded studies**<br>Enter a statement with the following details:<br>• Initials of the authors who received each award<br>• Grant numbers awarded to each author<br>• The full name of each funder<br>• URL of each funder website<br>• Did the sponsors or funders play any role in the study design, data collection and analysis, decision to publish, or preparation of the manuscript?<br><br>Did you receive funding for this work?"	
Please select the country of your main research funder (please select carefully as in some cases this is used in fee calculation).<br>as follow-up to "**Financial Disclosure**<br><br>Enter a financial disclosure statement that describes the sources of funding for the work included in this submission. Review the submission guidelines for detailed requirements. View published research articles from *PLOS Genetics* for specific examples.<br><br>This statement is required for submission and **will appear in the published article** if the submission is accepted. Please make sure it is accurate.<br><br>**Funded studies**<br>Enter a statement with the following details:<br>• Initials of the authors who received each award<br>• Grant numbers awarded to each author<br>• The full name of each funder<br>• URL of each funder website<br>• Did the sponsors or funders play any role in the study design, data collection and analysis, decision to publish, or preparation of the manuscript?<br><br>Did you receive funding for this work?"	UNITED STATES - US
**Competing Interests**	KMM and ZNA are inventors on US provisional patent application

On behalf of all authors, disclose any competing interests that could be perceived to bias this work.<br><br>This statement will be typeset if the manuscript is accepted for publication.<br><br>Review the instructions link below and PLOS Genetics' competing interests policy to determine what information must be disclosed at submission.	PCT/US2021/041951, submitted by Texas A&M University, which covers vector constructs that are pre-programmed to self-eliminate or self- remove at a predetermined time, and methods of making the same. PRC, JZ, RDM, MLNM declare no competing interests.
**Data Availability**<br><br>Provide a **Data Availability Statement** in the box below. This statement should detail where the data used in this submission can be accessed. This statement will be typeset if the manuscript is accepted for publication.<br><br>Before publication, authors are required to make all data underlying their findings fully available, without restriction. Review our PLOS Data Policy page for detailed information on this policy. Instructions for writing your Data Availability statement can be accessed via the Instructions link below.	All data generated or analyzed during this study are included with this published article. There are no additional datasets associated with this manuscript.

Powered by Editorial Manager® and ProduXion Manager® from Aries Systems Corporation

Manuscript Click here to access/download;Manuscript;pgenetics-568397.pdf

Repeat mediated excision of gene drive elements for restoring wild-type populations

Pratima R Chennuri[1], Josef Zapletal[2], Raquel D Monfardini[1], Martial Loth Ndeffo-Mbah[3,4], Zach N Adelman[1], Kevin M Myles[1]

[1]Department of Entomology and AgriLife Research, Texas A&M University, College Station, TX 77843, USA

[2]Department of Industrial and Systems Engineering, Texas A&M University, College Station, TX 77843, USA

[3]Department of Veterinary Integrative Biosciences, Texas A&M University, College Station, TX 77843, USA

[4]Department of Epidemiology and Biostatistics, Texas A&M University, College Station, TX 77843, USA

Correspondence: *mylesk@tamu.edu

Abstract

We demonstrate here that single strand annealing (SSA) repair can be co-opted for the precise autocatalytic excision of a drive element. Although SSA is not the predominant form of DNA repair in eukaryotic organisms, we increased the likelihood of its use by engineering direct repeats at sites flanking the drive allele, and then introducing a double-strand DNA break (DSB) at a second endonuclease target site encoded within the drive allele. We have termed this technology Repeat Mediated Excision of a Drive Element (ReMEDE). Incorporation of ReMEDE into the previously described mutagenic chain reaction (MCR) gene drive, targeting the *yellow* gene of *Drosophila melanogaster*, replaced drive alleles with wild-type alleles demonstrating proof-of-principle. Although the ReMEDE system requires further research and development, the technology has a number of attractive features as a gene drive mitigation strategy, chief among these the potential to restore a wild-type population without releasing additional transgenic organisms or large-scale environmental engineering efforts.

Key words

Gene drive, Single Strand Annealing, SSA, DNA Repair, Drosophila, CRISPR, Cas9, Homologous Recombination, HR, Direct Repeats, autonomous, homing, double-strand break

Introduction

Gene drives are currently being developed as tools for the control of sexually reproducing populations of organisms that negatively impact public health, agriculture, and conservation efforts (Burt, 2003; Esvelt et al., 2014). The core technology underlying various gene drive-based control methods currently under development are transgenes that will be inherited at super-Mendelian rates, after the individuals that have been engineered to carry them mate with wild populations. Examples of organisms that are currently being considered for control with this technology include insects that adversely affect public health, e.g., mosquito vector species (Gantz and Bier, 2015; Hammond et al., 2016; Kyrou et al., 2018), agriculture, e.g., pest species (Courtier-Orgogozo et al., 2017; Scott et al., 2018); and invasive rodents threatening the natural fauna and flora of island ecosystems, e.g., rat and mouse species (Grunwald et al., 2019; Leitschuh et al., 2018). Although the concept of large-scale genetic engineering projects to control or modify entire populations has existed for some time (Curtis, 1968, p. 19; Deredec et al., 2008), the description and development of the type II CRISPR/Cas9 system for introducing targeted double-strand DNA breaks (DSBs) (Jinek et al., 2012), with subsequent demonstration of the system's potential for inducing mutagenic chain reactions (Gantz and Bier, 2015), has spurred a period of rapid technological development in this area. In theory, the CRISPR/Cas9 system is capable of cutting the DNA of any organism. Thus, many different gene drive configurations have been developed or theorized that are based on this programmable nuclease system originally from bacteria and archaea (Esvelt et al., 2014; Raban et al., 2020).

While none of these configurations have yet been tested in the field, the development of CRISPR/Cas9-based homing gene drives are proceeding rapidly (Raban et al., 2020). In this configuration, the transgene encodes the Cas9 endonuclease and a guide RNA (gRNA). The synthetic gRNA will direct the Cas9 to a predetermined target site present in the organism's genome, resulting in a DSB at the target location. As eukaryotic organisms routinely repair DSBs, engineering the transgene with the sequences flanking the DSB (i.e., homology arms) enables a form of homology directed repair, termed homologous recombination (HR), that is capable of restoring the integrity of the double helix structure at the break site using a nearby homologous donor sequence, usually a sister chromatid during the S and G2 phases of the cell cycle. While HR is the most common of the various homology directed repair processes, it also requires the longest lengths of sequence homology between the donor and acceptor DNA. Homology directed repair of the DSB site, with the transgene serving as the donor, results in "gene conversion". If gene conversion occurs in the germline, the transgene will be heritable, and in theory the process repeated indefinitely in subsequent generations, converting heterozygous progeny to homozygous progeny in the germline. This process ensures that the transgenic drive allele is inherited at rates far exceeding normal ratios of Mendelian inheritance. For this reason, homing gene drives have been referred to as being "threshold-independent", i.e., capable of being transmitted through a population following the introduction of an undefined but low number of individuals carrying the transgenic drive allele (Marshall and Akbari, 2018). However, multiple studies that have mathematically modeled the spread of homing drives predict that in reality this type of drive may only be able to spread through a population when introduced above a particular frequency, which is likely determined by a number of different factors (Alphey and Bonsall, 2014; Deredec et al., 2008; Tanaka et al., 2017; Unckless et al., 2015).

I think it is more accurate to invert this - HDR is a form of homologous recombination, not the other way round

mention some of them concisely - why be stingy here, and ruin such a thorough introduction?

Nevertheless, the specter of introducing, at previously unimaginable scales, potentially irreversible alterations to complex ecosystems raises a number of complicated ethical, legal, and social concerns (Montenegro de Wit, 2019; NASEM, 2016). Therefore, if the potential medical, agricultural and environmental benefits of gene drive technologies are to be fully realized, these concerns will need to be mitigated. This can be achieved through the development of technologies capable of limiting, both spatially and temporally, the spread of gene drives through a population (Marshall and Akbari, 2018; Raban et al., 2020). While a number of mitigation strategies for minimizing the possibility of unanticipated harmful effects on humans, animals and the environment have been proposed (Basgall et al., 2018; DiCarlo et al., 2015; Esvelt et al., 2014; Gantz and Bier, 2016; Marshall and Akbari, 2018; Wu et al., 2016; Xu et al., 2020), current state-of-the-art technologies for limiting CRISPR/Cas9-based homing gene drives are primarily based on various designs of transgenic genetic elements (Gantz and Bier, 2016) or split drive configurations (Kandul et al., 2021; Li et al., 2020; López Del Amo et al., 2020; Oberhofer et al., 2021). A number of other systems have also been proposed, and in some cases demonstrated in laboratory studies, to limit the spread of CRISPR/Cas9-based homing gene drives (Basgall et al., 2018; Chae et al., 2020; DiCarlo et al., 2015; Esvelt et al., 2014; Taxiarchi et al., 2021). Although demonstrating the efficacy of these systems is a significant advance, it is not yet clear that any of the current technologies will be able to adequately address concerns surrounding the use of gene drives in nature. While other types of drive mechanisms also exist (Bier, 2022), and may ultimately prove to raise fewer concerns than homing drives, no drive technology has yet been deemed entirely safe and tested in nature.

the primary citations, using CATCHA, anti-Cas elements etc need to be mentioned here

not explained

OK, so they are mentioned here but this opening is incongruous with the previous statement that said 'primarily based'

Previously, we proposed a novel approach to leverage a less common form of homology directed repair, single-strand annealing (SSA), for the precise removal of gene drive alleles (Zapletal et al., 2021). In theory, this technology has the potential to permit an autonomous low-threshold drive to spread through a population over a specified period of time, after which the drive allele would be remediated from the environment, fully restoring a wild-type population. Here, we demonstrate proof-of-concept for this approach through the precise autocatalytic excision of an autonomous CRISPR/Cas9-based homing drive. This was accomplished by co-opting the SSA repair mechanism, following induction of a DSB by a second endonuclease at a target site encoded within the drive allele. Although SSA is not the predominant form of DNA repair in eukaryotic organisms, we increased the likelihood of its use by engineering direct repeats at sites flanking the drive allele. We have termed this technology Repeat Mediated Excision of a Drive Element (ReMEDE).

This absolutletly NEEDS a cartoon schematic to show overall approach

this is a statement that I'm not sure is validated, in the specific context of deliberate endonuclease-mediated homing setup, is it? i.e. if it has equivalent intrachromosomal and inter-chromosomal homology, what are the relative rates of repair across the two?

Methods

Plasmid Construction

Standard recombinant DNA techniques were used to clone all constructs. Supplementary figures S1A (yDR and yReMET), S2A (yMCR) and S2B (yReMEDE) outline plasmid construction. Complete maps and sequences are shown in Fig. S6.

Microinjections

Plasmids were purified using the Macherey-Nagel NucleoBond Xtra Midi endotoxin-free kit (#740420) and sent to BestGene Inc. or GenetiVision for embryo microinjections. The plasmids p-yMCR.EGFP and p-yDR.EGFP were co-injected with transient sources of a single guide RNA targeting exon 2 of the *yellow* gene (pCFD3-dU6:3-y1-sgRNA) and Cas9 (pBS-Hsp70-Cas9, a gift from Melissa Harrison & Kate O'Connor-Giles & Jill Wildonger - Addgene plasmid # 46294) into a w^{1118} stock (BDSC #3605). The plasmids p-ReMET.RFP and p-ReMEDE.RFP were co-injected with a transient source of ϕC31 into stocks carrying the construct p-yDR.EGFP. All transgenic stocks were confirmed through PCR and Sanger sequencing.

Genomic DNA Isolation and Sequence Analysis

Genomic DNA was prepared from individual flies according to protocols from Gloor et al., 1993 (Gloor et al., 1993). PCR reactions were assembled with GoTaq®G2 Green Master Mix (#7823), purified with Zymo Research DNA Clean & Concentrator-5 kit (#4014), sequenced and analyzed with SnapGene and PolyPeakParser.

Genetic Crosses and Phenotyping

Injected G0 transformants were crossed with w^{1118} flies and G1 progeny screened for either EGFP or RFP positive individuals. These individuals were then outcrossed with w^{1118} flies prior to establishing homozygous lines. The yMCR or yReMEDE males were crossed with wild-type (w^{1118}) females in order to generate F1 females. The F1 females were then mated with wild-type males in single pair crosses for scoring F2 gene drive phenotypes.

Population Cage Studies

Discrete small scale population studies were set up in standard fly rearing bottles (Genesee Scientific Cat. #32-130) with a design similar to those described previously (Bishop et al., 2022; Xu et al., 2020). Briefly, for each genotype an equal number of virgin females and males were seeded. All flies were removed following egg laying. The progeny from each subsequent generation were collected and then separated into two equal pools (n=150) of randomly selected flies. Flies from one pool were screened and phenotyped, while the other pool was seeded into a new cage.

Drosophila Husbandry

The I-Site reporter line, w^{1118}*; P{*$w^{+mW.hs}$*=w8,I-site}7* (BDSC# 6972), was sourced from the Bloomington Drosophila Stock Center. A nos-Gal4 line, *y* w*; P{*w^{+mC}*=GAL4-nos.NGT}40* (Kyoto DGRC# 107-748), was obtained from the Kyoto Drosophila Stock Center. Stocks were maintained on standard oatmeal/cornmeal medium at 18 °C, but all experiments were performed at 25 °C, unless otherwise noted. All gene drive lines were handled in an ACL-2 facility in accordance with standard containment protocols. All work with genetically modified organisms was performed under protocols approved by the Institutional Biosafety Committee at Texas A&M University.

Results

Repeat Mediated Excision of a Transgene (ReMET)

As an initial test of the hypothesis that the SSA repair pathway can be co-opted for the removal of gene drive alleles, we generated multiple transgenic lines containing ReMET reporter constructs flanked with SSA substrates (i.e., direct repeats) of various lengths. range? Briefly, the reporter constructs consist of an endonuclease, I-*Sce*I, under the control of a 5x Upstream Activation Sequence (*UASp.I-SceI*), the 18 bp I-*Sce*I target site (I-Site), and two fluorescent marker sequences, enhanced green fluorescent protein (EGFP) and red fluorescent protein (RFP), under the control of independent 3xP3 promoters. The yeast transcription activator protein, Gal4, was placed under the control of the germline-specific promoter *nanos* (*nos*) in a mifepristone (RU486)-inducible GeneSwitch expression system (“non-GS”") - it took me ages to understand what was meant by this!! (Fig. 1A and S1A). Constructs were inserted into the *yellow (y)* gene of *D. melanogaster*. Expression of I-*Sce*I generates a DSB at the I-Site sequence, which facilitates SSA-based repair mediated by the presence of the direct repeats, leading to transgene excision. Successful ReMET in F2 progeny is indicated by the loss of yellow body color and both fluorescent marker sequences (Fig. 1A). A silent mutation (TGG > TcG) was engineered into the right homology arm flanking the yReMET constructs so that a single nucleotide change would be left behind following SSA repair, providing evidence that the transgene had in fact been present prior to removal, which would otherwise be indistinguishable from wild-type sequences (Fig. 1A and S1A). To our knowledge, the range of direct repeat substrate lengths over which there is a linear relationship with SSA frequency has not been determined in *D. melanogaster.* Therefore, yReMET lines with either 30, 250, or 500 bp of homologous flanking repeat sequences were created. ok

is this PCR then sequencing? Do the primers bind either side of the (previous) insertion i.e. either side of the putatively repaired cut site i.e. as opposed to both being on the downstream side of the cut site yet incorporating the TcG SNP? In the latter case I'm wondering about template switching in the PCR between the TGG alleles and the TcG alleles which might confuse things a little

It is well known that the GeneSwitch system exhibits some leakiness (Scialo et al., 2016). Therefore, the stability of reporter lines containing direct repeats was initially investigated with ReMET constructs lacking the inducible *nos-GS/*GAL4 expression system. Importantly, we did not observe evidence of unintended recombination events after crossing these lines with wild-type flies (Fig. S1B). However, pair-mated reciprocal crosses of F1 flies with a *nos-Gal4* driver line resulted in successful ReMET events (i.e, excision of the transgene mediated by SSA). The frequency of these events was consistent with a linear relationship between SSA and the direct repeat lengths flanking the transgene, with lower rates of excision observed for the 250 bp length and higher rates observed with the 500 bp length (Fig. 1B). We did not observe SSA-mediated excision of the transgene with the 30 bp direct repeat length (Fig. 1B), suggesting that this length may be at or below a minimum effective processing segment (MEPS) length (Shen and Huang, 1986). Interestingly, SSA appeared to occur at a relatively higher mean rate in the male germline, with successful ReMET observed in ~15% of F2 progeny when the 250 bp direct repeats were present, and in ~35% of the F2 offspring when the 500 bp direct repeats were present. However, successful ReMET was observed in only ~6% of the F2 offspring of yReMET females for the 250 direct repeat length, and in ~15% of progeny when the 500 bp direct repeats were present (Fig. 1B).

As expected, crossing siblings with the complete ReMET construct, which included *nos-GS/*GAL4, in the absence of RU486 resulted in low levels of progeny (0.2 to 0.6%) that exhibited evidence of SSA (Fig. S1C). However, a more surprising result was that significant increases in the frequencies of successful ReMET were not observed in the presence of RU486 (Fig. S1C). ReMET was observed in ~1% of the F1 offspring with the 250 direct repeat length, and in ~0.5% of the progeny when the 500 bp direct repeats were

present (Fig. 1C). Similar to our earlier results, there was little evidence of SSA with the 30 bp direct repeat length (Fig. 1C). It is possible that the relatively lower levels of SSA observed in these experiments are somehow related to the continual leaky expression of the endonuclease, I-*Sce*I, from the *nos-GS*/GAL4 system throughout development, rather than introduction through a parental cross, as was the case with the previous *in trans* configuration. Nevertheless, the presence of the silent molecular marker, TcG, engineered into the right homology arm provided sequence confirmation of ReMET in all flies exhibiting evidence of SSA (i.e., the loss of yellow body color as well as both fluorescent marker sequences). In order to confirm activation of the *nos-GS* by mifepristone, males containing the full ReMET construct were crossed with a previously published transgenic line (Rong and Golic, 2000), containing a *white* (w^+) reporter gene with an adjacent I-Site sequence, in the presence of increasing concentrations of RU486. As expected, cutting at the site adjacent to the w^+ reporter by I-*Sce*I, expressed from the ReMET cassette, deleted all or part of the w^+ gene resulting in eye-color mosaicism (Fig. S1D). While I-*Sce*I was expressed at low levels from 5xUAS, even in the absence of treatment with mifepristone, increasing levels of mosaicism were observed in the eyes of F1 progeny depending on the concentration of RU486 present (Fig. S1D). This confirms that the *nos-GS* system present in the full ReMET constructs is functioning properly. Thus, leaky expression of I-*Sce*I from the *nos-GS* system appears to induce SSA at a maximum level, explaining why additional activation with RU486 does not increase ReMET frequencies in the F1 progeny (Fig. S1C). Nevertheless, these experiments demonstrate co-option of the SSA repair pathway for the precise removal of a transgene from *D. melanogaster*.

see my above comment on a priori expectations of rates of SSA

Super-Mendelian inheritance of a CRISPR/Cas9-based autonomous homing gene drive

In order to extend our findings demonstrating removal of a transgene to an active gene drive, we reconstructed the previously described CRISPR/Cas9-based yMCR gene drive (Gantz and Bier, 2015; Xu et al., 2020). This gene drive system is based on homology-dependent integration into the X-linked recessive *yellow* locus (Fig. S2A). As per Mendel's first law, a transgene segregates independently during gamete formation, so that each gamete carries only a single transgenic allele (Fig 2B). However, in genetic drive, allelic conversion transforms heterozygous recessive loss-of-function mutations into homozygous null mutations in the homogametic maternal germline (Fig. 2C), resulting in super-Mendelian inheritance of the drive transgene at frequencies > 50% (Champer et al., 2017; Gantz and Bier, 2015; Xu et al., 2020). This process, which is mediated through repair of the DSB by HR, has also been referred to as a mutagenic chain reaction (MCR). However, other genetic outcomes are also possible (Fig. 2C). Repair of the DSB generated by the Cas9 RNP by the more error prone non-homologous end joining (NHEJ) pathway may sometimes generate resistant alleles. Male flies inheriting resistant alleles may sometimes exhibit a wild-type phenotype, if the mutation does not shift the open reading frame, or a yellow body phenotype, if an out-of-frame mutation occurs (Fig. 2C). Female progeny inheriting these alleles generally do not appear yellow, even if the mutation is out-of-frame, as the paternal wild-type allele is dominant (Fig. 2C). However, female progeny inheriting a maternal allele containing either the transgene or an out-of-frame mutation may occasionally exhibit a yellow phenotype, or mosaic patches of wild-type pigmentation on a largely yellow body. This likely occurs through the persistence of maternally deposited Cas9 RNP in the zygote, which repeatedly cleaves the paternal wild-type allele until it is converted into a mutant allele by NHEJ, resulting in the observed zygotic allelic conversion, or somatic mosaicism (Fig. 2C). While in-frame mutations may sometimes result in a wild-type phenotype, out-of-frame alleles generally produce female

seems a bit excessive to repeat all this after havign done such a good job in Intro but just a difference in style

even more repetivie, especially as you already mentioned 'MCR' earlier. AS it goes, I don't like the term, especially as you also call it homing-based gene drive. Likely to cause confusion

all of this seems superfluous to the story at hand and can surely be explained more concisely, moving some of it to supp info??

progeny with the yellow body pigmentation (Fig. 2C). This non-Mendelian exception resulting from the perdurance of maternally deposited Cas9 RNP has been termed “dominant maternal effect” (Lin and Potter, 2016), and has been observed in gene drive experiments across species (Champer et al., 2018, 2017; Gantz and Bier, 2015; Grunwald et al., 2019; Guichard et al., 2019; Hammond et al., 2016; Xu et al., 2020).

Our MCR drive was modified to include *3xP3-EGFP* (MCR-EGFP) so that we could track the appearance of resistant alleles, both functional and non-functional (Fig. S2A). To validate the drive, we performed a series of independent single pair-mated outcrosses (n=58) with heterozygous F1 females and wild-type males. The F1 females were generated by crossing yMCR males to wild-type females. The mean transmission rate of the drive allele to the F2 progeny over all crosses was ~87% (Fig. 2C). The mean rate of offspring exhibiting a yellow mutant phenotype was ~11%, with the remaining ~2% of all F2 progeny displaying a wild-type phenotype (Fig. 2C). It should be noted that these percentages are consistent with previously reported yMCR inheritance patterns in *D. melanogaster* (Champer et al., 2018, 2017; Gantz and Bier, 2015; Xu et al., 2020).

all this could be summarised very briefly, since not novel nor pertinent to result other than “the gene drive we used showed transmission rate of 87%”

Repeat Mediated Excision of a Drive Element (ReMEDE)

flanked by repeats of 250bp

The yMCR construct was next recombined into the yReMET (250 bp) reporter line by standard methods to generate a yReMEDE line (Fig. 2A and Fig. S2B). Based on results regarding direct repeat substrate length and SSA frequency (see above), all subsequent experiments employed a direct repeat length of 250 bp. F1 females were generated by crossing yReMEDE males to wild-type females. The performance of the yReMEDE system was then assessed in a parallel series of outcrosses (n = 58), where heterozygous F1 yReMEDE females were pair-mated with wild-type males. The allelic inheritance patterns of the resulting progeny were then compared to those resulting from the outcrosses with the F1 yMCR females (Fig. 2C). Both the genotypes and phenotypes of progeny that are expected to result from crosses involving F1 yReMEDE females are shown in Figure S3. Successful ReMEDE was indicated by loss of fluorescent marker genes and reversion to wild-type body pigmentation in the F2 progeny (Fig. S3). As expected, the number of individuals with wild-type phenotypes increased significantly, but somewhat unexpectedly these comprised ~38% of the total yReMEDE progeny over all crosses (Fig. 2C). Further, this significant increase in wild-type progeny correlated with a significant decrease in the overall mean transmission rate of the drive allele (~54%; Fig. 2C and S2C). While we considered the hypothesis that incorporation of the ReMEDE components unintentionally disabled Cas9 resulting in uncut alleles, this was inconsistent with multiple observations. First, in single pair mated crosses (n = 30) of flies containing a transgene lacking drive elements, but inserted into the same location of the *yellow* gene, more than half of the F2 flies inherited the transgene at rates of less than 50%, with only a single replicate nominally exceeding the 60% threshold (Fig. 2B and Data S1). In contrast, transmission of the yReMEDE allele ranged from 18% to 75%, with rates over 60% being observed in 15 separate replicate crosses (Fig. 2C and Data S1). Further, the overall mean inheritance of the transgene was significantly lower than that of the ReMEDE construct (p value = 0.01; Fig. 2B and C). More importantly, yellow F1 females were generated by crossing yReMEDE males with wild-type females (Fig. 2C), which could not occur in absence of Cas9 mediated cutting. Additionally, the overall percentage of progeny exhibiting resistant alleles (i.e., yellow body color) in the yReMEDE F2 generations did not significantly differ from those observed in the yMCR F2 flies (Fig. 2C and Data S1). If the Cas9 were no longer active, the transgenic construct would then be subject to normal Mendelian

why is this unexpected? you said you expected more WTs. What is surpising, specifically, about the figure of 38%

isn't this also expected?

this statement confuses me - was the expectation not 50%?

this is very confusing and needs more granularity - you mean the 48.6% compared to 53.5%?

drive?

Text

….since it requires cutting at both alleles?

inheritance, with only GFP positive yellow males generated from the crosses (Fig. 2B). Thus, a more plausible hypothesis consistent with these results is that both ReMEDE and allelic conversion are occurring simultaneously.

it can be confusing what you mean by allelic conversion here and elsewhere - I believe you mean it to mean 'any' type of activity that changes the non-drive allele i.e. could be homing of transgene to convert wt allele, could be homing of mutation, could be de novo end joining mutation from maternally deposited nuclease?

Phenotypic scoring of the F2 yReMEDE flies serves as a proxy for the approximate rate of SSA. This is particularly true with regard to the observed increases in numbers of male wild-type progeny generated through outcrosses of F1 yReMEDE females. In males, X-linked inheritance is exclusively maternal, necessarily suggesting germline conversion mediated by SSA (Fig. S2C and S3). Nonetheless, sequencing over the Cas9 target site in all wild-type individuals resulting from multiple pair-mated outcrosses of the F1 yReMEDE females, for the silent mutation (TGG > TcG) engineered into the right homology arm of the construct, confirmed transgene (i.e., the drive element) excision by SSA. By design, this mutation also functions as an engineered resistant allele in the critical protospacer adjacent motif (PAM), essential for Cas9-mediated cleavage (Fig. 2A). The altered PAM sequence prevents self-targeting that might lead to the formation of undesirable NHEJ-mediated resistant alleles or allelic conversion within the ReMEDE allele. Although pair mated crosses with F1 yMCR females rarely produced wild-type phenotypes (Fig. 2C and Data S1), sequencing confirmed them to be indels, generated either by NHEJ acting on a maternally inherited allele, or a dominant maternal effect acting on a paternally inherited allele (Fig. 2D). Overall, these indel sequences occurred at a frequency of ~1-2% in the inherited alleles (Fig. 2D and Data S1). Alleles containing indels, generated either through NHEJ or dominant maternal effect, occurred with similar frequency (~3%) in the wild-type progeny of F1 yReMEDE females (Fig. 2E and Data S1). However, sequencing also confirmed SSA-mediated events in ~4% of the maternally inherited wild-type alleles in the F2 yReMEDE flies (Fig. 2E and Data S1), which were not present in F2 yMCR flies. Paternally inherited wild-type alleles also increased to ~14% in F2 yReMEDE flies (Fig. 2E and Data S1). However, in contrast with paternally inherited alleles in the wild-type F2 yMCR flies, which contained only indels (Fig. 2D and Data S1), paternal alleles in the wild-type offspring of yReMEDE flies remained intact or uncut, which is consistent with limited or no maternal deposition of Cas9 RNP (Fig. 2E and Data S1).

it makes it incredibly diffcult to read if we are 8 pages in and 2 figures down and only now readign about the intended design of the construct!

this is SO hard to read - I get the impression that the strains or constructs were not initially for the purpose of investigating this phenomenon but that the analysis shifted towards determining this. That is not necessarily a bad thing but, regardless of chronology or design, these results need more structure and correct emphasis

We were only able to confirm ~4% of the wild-type alleles resulted from ReMEDE. These alleles were necessarily contributed by the F1 maternal germline (i.e., present in F2 male progeny), and carried the TcG marker sequence (Fig. 2E, S3 and Data S1). However, wild-type alleles containing a wild-type TGG or gGG sequence (possibly produced through erroneous repair) were also present (Fig. 2E, S3 and Data S1). It should be noted that F2 female progeny also inherited alleles with the TcG marker, but ascertaining the precise origin of these alleles is more difficult (Fig. S3). Thus, to investigate the origin of alleles containing the TcG sequence, as well as the TGG and gGG sequences, further we sequenced all progeny generated from a single pair-mated cross with two sets of primers (Pair-mated cross #4; Data S1). The first primer set encompassed the PAM sequence, while the second set encompassed the entire transgene, with binding sites located outside of the construct's left and right homology arms (Fig. S2A and S2B). The use of both primer sets permitted us to ascertain the zygosity of both drive (ReMEDE) and non-drive alleles (wild-type or containing indels) present in the homogametic female progeny. The results confirmed that only ~3% of the wild-type alleles in heterozygous females carried the engineered TcG marker, with a larger ~17% exhibiting the original wild-type TGG sequence (Pair-mated cross #4, Data S1). In contrast, all ReMEDE drive alleles, present in either male or female progeny, were positive for the purposely engineered TcG variant of the PAM sequence (Pair-mated cross #4, Data S1).

Replicate *en masse* crosses involving multiple F1 yReMEDE females and wild-type males yielded an average transmission rate of ~53% for the drive allele, with ~42% of the progeny exhibiting a wild-type phenotype (Fig. S2D), numbers very similar to those observed in pair-mated crosses (Fig. 2C). Similar percentages were observed in a series of additional replicate crosses performed in the presence of RU486 (Fig. S2D). Sequencing over the Cas9 target site in wild-type individuals for the silent mutation (TGG > TcG), engineered into the right homology arm of the construct, again provided confirmation of drive element excision by SSA (Fig. 2E and Data S1). Taken together, these experiments demonstrate proof-of-principle for successful co-option of the highly conserved SSA repair pathway for the precise autocatalytic excision of an active gene drive.

apologies if I'm missing this but this is actually NOT what you have shown - you monitored the frequency of each element (alone) in a cage over time. In your proposed use case scenario of a remediation you propsoe that the ReMEDE can clear an active low threshold gene drive from a population. You have not done this, it would seem.

ReMEDE converts the MCR-EGFP gene drive to wild-type alleles in population cage studies

In order to determine if the ReMEDE system would convert the gene drive element into wild-type alleles over multiple generations, we conducted cage trials with either the yReMEDE or yMCR drive flies. The cage trials were performed in accordance with those described in previously published studies (Bishop et al., 2022; Xu et al., 2020). We evenly divided six hundred male and female virgins into three replicate cages (200/cage). Within each cage, 25% of the flies were wild-type and 75% homozygous for either yMCR or yReMEDE. At each generation (n), approximately 150 flies/cage were randomly selected for scoring (i.e., fluorescent markers and body color), and another randomly selected 150 flies/cage seeded into the subsequent generation (n + 1) of cages. The frequency of flies with unmodified yMCR alleles (EGFP+) decreased only modestly, still representing a significant portion of the population after 14 generations (Fig. 3A). In contrast, the frequency of flies exhibiting yReMEDE drive phenotypes (EGFP+, RFP+, and yellow body color) decreased gradually, and were completely eliminated in 10-14 generations (Fig. 3B). Reciprocally, the frequency of flies exhibiting a wild-type phenotype increased gradually, representing 100% of the population after 10-14 generations (Fig. 3B). These experiments suggest that all gene drive elements carrying ReMEDE will eventually be converted to wild-type alleles given enough generations.

OK, having read on I understand it now but I'll leave the above point to illustrate where the confusion comes from in the hope that it will help in reframing it to make it more reader friendly) - I believe the confusion comes from conflation of the 'process' of ReMEDE and the 'genetic element' REMEDE (or yReMEDE)

I think an outsider looking at this might strugkgle to concludet automatically that the trajectory of yREMEDE is better than that of the yMCR in this case

ReMEDE-mediated conversion of a threshold-independent gene drive to wild-type

The conversion of the yMCR gene drive element into wild-type alleles by ReMEDE over multiple generations suggests that the system will perform similarly in converting a low-threshold gene drive into wild-type alleles. The *yellow* gene target site of the yMCR gene drive renders this element threshold dependent. First, the target cut site is susceptible to erroneous repair by NHEJ or other error-prone DNA repair pathways, which may disrupt the PAM sequence, generating alleles that can no longer be targeted by the Cas9 ribonucleoprotein (RNP), i.e., resistant alleles. Second, mutations in the *yellow* gene adversely affect male mating success (Barker, 1962; Bastock, 1956; Drapeau et al., 2006; Massey et al., 2019; Sturtevant, 1915; Xu et al., 2020). In order to generate mathematical models to predict the dynamics of a low or no-threshold drive containing ReMEDE over multiple generations, we determined the mating cost associated with *yellow* mutant flies, containing an insertion at the same site targeted by the drive allele. *D. melanogaster* engages in a series of courtship rituals (e.g., tapping, chasing, genital licking, singing, etc.) prior to successful copulation. A previous study has shown that the lower mating success of *yellow* males in comparison to wild-type males does not result from any observable difference in the amount of time spent courting, but rather from delayed initiation of copulation (Massey et al., 2019). However, these experiments were conducted with Canton-S wild-type flies. In our population studies, it was necessary to

these may well both be true but this is an inherent feature of this particular gene drive element (yMCR) and has nothing to do with REMEDE or other 2nd party mitigation measures…. seems a red herring to me

use the w^{1118} strain as wild type, in order to score eye-specific fluorescence. The *white* (*w*) gene has also been associated with copulation success, and w^{1118} flies display reduced courtship activity (Xiao et al., 2017). Thus, we calculated courtship indices (the proportion of time that a male was engaged in courtship activities divided by the total duration of observation) for wild-type (w^{1118}) and mutant (w^{1118};*yellow*) flies. Although wild-type males displayed reduced courting activities with wild-type females, the courtship of mutant *yellow* females was even more markedly reduced (Fig. S5A). Male *yellow* mutants had no success mating with wild-type females in these trials; thus, a courtship index was not recorded for these crosses (Fig. S5A). However, the courtship index of mutant *yellow* males was approximately 0.5, when calculated from crosses with mutant *yellow* females. Given the results of the single pair courtship assays, mating costs associated with mutant *yellow* males were instead calculated from competitive assays, where a wild-type virgin female was provided with both a wild-type male and mutant *yellow* male. In the 30 crosses performed for these assays, a single *yellow* male, exhibiting a courtship index of ~0.5, successfully copulated (Fig. S5A). Based on the results of these experiments, we estimated the mating cost associated with mutant *yellow* males to be 29/30 or 0.97 (Fig. S5A).

We have previously generated mathematical models for SSA-based excision of transgenes (Chae et al., 2022; Zapletal et al., 2021). These initial parameters were used to simulate allele frequencies for both autosomal and X-linked inheritance patterns (Fig. S5C and Supplementary Methods), and then modified to include empirical values derived from crosses involving yMCR (δ = NHEJ and mating cost of 0.97 for yellow males) and yReMEDE (α = SSA, γ = SSA resistance, and ε = engineered allele persistence) flies. This model was then used to run simulations of various scenarios. In conjunction with these simulations, we conducted multi-generation population cage trials to assess the impact of mating costs and resistant alleles associated with the *yellow* gene target site of the yMCR gene drive. Replicate cages were seeded with flies, such that yMCR females or males comprised 10% of the population, with the other 90% being virgin wild-type males or females; a level that might be consistent with the introduction of a low-threshold drive element. The scoring of each generation (n) and seeding of subsequent generations (n + 1) was performed as described above. In each of the cages, individuals exhibiting fluorescence and yellow body phenotypes were rapidly lost from the population, regardless of sex (Fig. S5B). Simulations conducted with similar parameters matched the observed performance of the drive element (g) in population cage studies (Fig. S5D). In an attempt to remove the mating costs associated with the *yellow* gene target site, additional cages were seeded with flies in which yMCR females or males again comprised 10% of the population, but with the other 90% being males or females homozygous for the null *yellow* allele (i.e., a mutant population). The frequency of the EGFP+ cassette (i.e., the yMCR drive allele) increased in the population, ultimately achieving 15-60% penetrance when introduced by female flies, and 30-80% penetrance when introduced by the male flies (Fig. S5B). Simulations of male releases derived with similar parameters roughly approximated the trends observed in the population cage trials (Fig. 4A).

Additional mathematical modeling removing all mating costs associated the yReMEDE drive, and with SSA (female α = 0.1, male α = 0.01) occurring at ~10%, where all events are marked (TcG) by the engineered resistant allele (v; ε = 1), results in a maximum ~50% drive allele frequency after about 15 generations, which are then progressively replaced by drive-resistant TcG-marked wild-type alleles (v) (Fig. 4B). However, several studies have analyzed and overcome the challenge of resistant alleles, either by targeting essential genes, using multiple guide RNAs, recoding target genes, or by incorporating a

combination of these strategies to ensure that only homozygous drive or wild-type allele combinations remain viable (Champer et al., 2020, 2018; Kandul et al., 2021; Kyrou et al., 2018). In lieu of these empirical developments, we simulated yReMEDE population dynamics in the absence of both mating costs and resistant alleles (δ = 0), with three different SSA frequencies. If approximately 30% of maternally inherited alleles are formerly drive alleles that were converted to wild-type alleles by SSA, of which 25% retain the engineered TcG resistant allele (v; ε = 0.25), w alleles (native wild type) and v alleles (drive-resistant wild type) persist in the population, while the drive alleles (g) are progressively eliminated (Fig. 4C). Alternatively, when SSA occurs at ~10%, with all events retaining the TcG marker (ε = 1), allelic conversions of wild-type alleles (w) by drive alleles achieve a maximum frequency of ~60% in < 15 generations, but are then subsequently entirely replaced by drive-resistant alleles (v) via SSA (Fig. 4D). When SSA occurs at the minimal rate of ~5%, with only 25% of these retaining the TcG marker (ε = 0.25), the frequency of the drive allele (g) quickly reaches ~90% in < 15 generations, but is subsequently replaced by engineered drive-resistant alleles (v) after several more generations (Fig. 4E). Therefore, taken together, stochastic modeling informed by empirical data, indicates that a mere 5-10% of SSA events where only one-fourth of the events remain drive-resistant (v), allow for efficient propagation of a low-threshold drive with eventual replacement by engineered drive-resistant wild-type alleles.

Discussion

Early successes mitigating the negative effects of resistance alleles on drive efficiency (Carballar-Lejarazú et al., 2020; Hammond et al., 2021, 2017; Kyrou et al., 2018; Unckless et al., 2017) suggest that the development of technologies for limiting the spread of drives through a population may be necessary, if such tools are ever to be successfully tested and deployed in the field. Indeed, several technological solutions have recently been proposed for homing drives, with the design and demonstration of various braking systems and confinable gene drive configurations representing a particularly positive development in this regard (Basgall et al., 2018; Chae et al., 2020; DiCarlo et al., 2015; Esvelt et al., 2014; Gantz and Bier, 2016; Taxiarchi et al., 2021; Wu et al., 2016). Nonetheless, it remains unclear if any of the currently proposed technologies will ultimately prove to be an adequate solution. First, many of these strategies are premised on the introduction of additional independent transgenic genetic elements. These elements share a number of similarities with gene drives themselves, and as a result their efficacy may be influenced by the same parameters as those affecting the homing drives they are designed to stop (i.e, gene conversion efficiency, fitness costs, timing, etc.), and indeed this has been observed in several laboratory trials and simulations (Alphey and Bonsall, 2014; Deredec et al., 2008; Tanaka et al., 2017; Unckless et al., 2015; Xu et al., 2020). Second, the introduction of additional transgenic elements may complicate risk assessment, as it might not be possible to extrapolate with a high enough degree of confidence how an autonomous drive might function in a particular ecological environment based on field testing in a split configuration, or in the presence of an independent genetic braking element. Third, no strategy satisfactorily addresses the critical requirement for removal of transgenes from natural populations, should that become necessary. Rather, the removal of multiple independent transgenes at the conclusion of a field trial, in any sort of acceptable time frame, would likely require inundation through the sustained release of wild-type individuals. We, therefore, sought to develop a solution where the excision of the transgene would be autocatalytic over the period of genetic drive, eventually restoring the wild-type population. By design, drive activity is meant to be gradually reduced as drive alleles are converted to wild-type alleles. We have previously proposed co-opting the SSA repair mechanism for this purpose (Zapletal et al., 2021, Chae et al., 2022).

it would be nice to state this, right at the start. A cartoon of the conceived approach would help!

Here, we demonstrate proof-of-concept using a self-eliminating version of the yMCR drive element (Gantz and Bier, 2015) targeting the *yellow* gene of *D. melanogaster*. The system, which we termed ReMEDE, significantly increased wild-type progeny. Crossing F1 yMCR females with wild-type males rarely produced wild-type individuals (Fig. 2C and Data S1). Sequencing these individuals confirmed them to be the result of maternally inherited indels generated through NHEJ, or a dominant maternal effect, acting on the paternally inherited allele (Fig. 2D). Overall, these indel sequences were present in ~1-2% of the inherited alleles (Fig. 2D and Data S1). These results are consistent with previous studies with the yMCR drive where the occurrence of in-frame, functional, mutations were also found to be relatively rare (Champer et al., 2017; Gantz and Bier, 2015; Xu et al., 2020). Alleles with indels generated either through NHEJ or dominant maternal effect were found to occur with similar frequency (~3%) in the progeny of F1 yReMEDE females (Fig. 2E and Data S1). However, in addition to these, sequencing confirmed SSA-mediated events in ~4% of the maternally inherited alleles (Fig. 2E and Data S1), confirming a genetic basis for observed increases in wild-type progeny in the crosses involving yReMEDE flies. Paternally inherited wild-type alleles also increased to ~14% in the yReMEDE offspring (Fig. 2E and Data S1). However, in contrast with the wild-

This is not a Discussion, this is a recapitulation of the Results. I find it much easier to read and keep hold of than the current Results section as it is currently written

type progeny of the yMCR flies, where all paternal alleles had been converted into indels, all of the paternal alleles present in the wild-type progeny of the yReMEDE flies remained intact or uncut, suggesting that the maternal deposition of Cas9 RNP was somehow prevented (Fig. 2E and Data S1). One possible explanation for this is that ReMEDE-mediated removal of the gene drive, suppressed Cas9 RNP perdurance (Fig. 2D, S2C, 2E, and Data S1), which would have otherwise resulted in zygotic allelic conversion.

Sequencing wild-type individuals generated from the pair-mated crosses of yMCR females did not reveal any alleles with a TGG PAM sequence (Fig. 2D and Data S1), suggesting that non-drive alleles are repeatedly cut with the Cas9 nuclease, eventually resulting in either allelic conversion or the formation of a resistant allele. Indels were found with similar frequency in the wild-type offspring of both yReMEDE and yMCR females (Fig. 2D, 2E and Data S1), as were resistant alleles (i.e., yellow progeny) in the F2 generations (Fig. 2C and Data S1), suggesting that the Cas9 remains active in the yReMEDE drive allele. Rather, the primary difference, evident from the results of single pair mated crosses, was the presence of wild-type flies with alleles containing either a TGG or TcG sequence at the PAM site in the F2 yReMEDE generation, which were not found in the F2 yMCR generation (Fig. 2D, 2E and Data S1). Sequencing of intact ReMEDE drive alleles revealed only the purposely engineered TcG variant of the PAM sequence (Pair-mated cross #4, Data S1). Thus, overall, these results are consistent with both drive conversion and SSA happening simultaneously in the ReMEDE flies. While we can only speculate on the origin of the wild-type alleles containing the TGG sequence, one possibility is that these are uncut alleles occurring because of ReMEDE-mediated removal of the drive allele. However, another hypothesis is that ReMEDE occurs with subsequent conversion of TcG to TGG in the F1 heterozygous maternal germline, via synthesis-dependent strand annealing (SDSA) and mismatch repair (MMR; see Fig. S4 for model).

There are two generally accepted models for the repair of DSBs by HR, double strand break repair (DSBR) and synthesis-dependent strand annealing (SDSA). Both models begin with the resection of the 5' termini flanking the DSB. This generates long single-stranded 3' tails, one of which invades the homologous duplex template sequence resulting in the formation of a D-loop. After this step, the two models diverge wherein the DSBR model is characterized by formation of double Holliday junctions (dHJs), the resolution of which may result in either crossover or non-crossover products (Szostak et al., 1983). SDSA is mediated by new synthesis, disassociation of the recently extended strand, and annealing with the other 3' tail in the broken chromosome, exclusively generating non-crossover products (Fig. S4). While the DSBR model appears to be preferred in meiotic cells, evidence increasingly suggests that the SDSA model is the major pathway of DSB repair in mitotic cells (Andersen and Sekelsky, 2010). In some cases, MMR machinery appears to be capable of recognizing and repairing mismatches present in the transient heteroduplex DNA (hetDNA) intermediates formed during the repair process (Jiricny, 2006). However, the newly synthesized strands, produced in both the DSBR and SDSA models, may also form hetDNA that can be detected and repaired through MMR. In either case, loss of heterozygosity would be possible through nonreciprocal transfer of information from the template strand (Hum and Jinks-Robertson, 2017). In the ReMEDE system, DSBs generated by I-*Sce*I occur between engineered direct repeats increasing the likelihood that they will be repaired by SSA, whereas those generated by Cas9 do not occur between direct repeats making it more likely that HR will be chosen. Occurrences of wild-type PAM sequences (TGG) in the yReMEDE lines, but not in yReMET lines that were devoid of Cas9, might suggest that it is the cleavage mediated by Cas9 that

is necessary for SDSA, and subsequently MMR, resulting in reversion of the engineered marker TcG to TGG. Sequencing of progeny from single pair-mated crosses revealed that all of the ReMEDE drive alleles present retained the engineered silent mutation, suggesting that these reversions would have to happen following excision of the ReMEDE construct (Data S1).

Our previous work on this topic was entirely conceptual, as such it was impossible to know how this technology would perform in an actual biological organism. However, one concern that we considered a distinct possibility was that SSA might be too efficient at removing the transgene, to the point that gene drive might not occur. In order to address this possibility, we placed the second nuclease (I-SceI) under the control of an inducible gene switch system. However, this system is leaky, and we continue to see expression of I-SceI (Fig. S1D). To our knowledge, all inducible systems exhibit some level of leakiness. In any case, these concerns ultimately proved to be unfounded, as SSA occurred at relatively low levels, even when higher levels of the nuclease were produced (Fig. S1C). These results suggest that there is no need for an inducible system in future iterations of the technology, which in any case would have been difficult to implement in a field setting. In practice, SSA re-constitutes wild-type alleles at relatively low levels, which is desirable as the wild-type population is only gradually restored, permitting a period of genetic drive. In multi-generational population cage studies, the ReMEDE drive element was completely eliminated, and a wild-type population restored in 10 to 14 generations (Fig. 3). A number of factors related to the performance of the yMCR gene drive (i.e., mating defects, resistant allele formation, etc.) prevented us from conducting population-level studies under conditions that would mimic the release of a low-threshold gene drive (Fig. S5). However, simulations with experimental parameters obtained from this study suggest a similar dynamic would occur with low threshold drives. Simulations of yReMEDE population dynamics, in the absence of both mating costs and resistant allele formation, determined that only 5% of SSA-mediated recombination events, even with only a fraction of these retaining the engineered PAM site, would be sufficient to permit the initial propagation of a low threshold drive, followed by its eventual replacement with wild-type alleles (Fig. 4E). These frequencies already fall below those observed in single pair mating experiments, suggesting that the ReMEDE technology may be capable of halting even the most robust gene drive, eventually reverting the population back to a wild-type state. Given the limitations of the yMCR gene drive, future work will focus on employing the ReMEDE system in the context of a more robust low threshold gene drive. However, the present study constitutes an effective proof-of-principle for the ReMEDE concept as a viable technology for controlling autonomous homing gene drives. The ReMEDE system has a number of theoretically desirable features as a technological safeguard for gene drives, including the potential to restore the original wild-type population, and addressing the issue of transgene persistence in natural populations. The ReMEDE concept should be considered along with other strategies currently under investigation for controlling, neutralizing, halting, deleting, etc., gene drives. Particularly, as early modeling efforts suggest that threshold-independent drives, similar to those that have already been developed in the malaria vector *Anopheles gambiae,* may be difficult to control with braking systems (Rode et al., 2020). While other strategies have also been demonstrated in laboratory settings, it is too soon to know if any of these will be successful in field settings (Basgall et al., 2018; Chae et al., 2020; Esvelt et al., 2014; Gantz and Bier, 2016). Further, it is entirely plausible that no mitigation strategy will prove ideal for every possible

scenario involving gene drives. Thus, prudence would appear to dictate that multiple gene drive mitigation strategies are pursued further at this juncture.

References

Alphey N, Bonsall MB. 2014. Interplay of population genetics and dynamics in the genetic control of mosquitoes. *J R Soc Interface* **11**. doi:10.1098/rsif.2013.1071

Andersen SL, Sekelsky J. 2010. Meiotic versus mitotic recombination: Two different routes for double-strand break repair. *BioEssays* **32**:1058–1066. doi:10.1002/bies.201000087

Barker JSF. 1962. Studies of Selective Mating Using the Yellow Mutant of Drosophila Melanogaster. *Genetics* **47**:623–640.

Basgall EM, Goetting SC, Goeckel ME, Giersch RM, Roggenkamp E, Schrock MN, Halloran M, Finnigan GC. 2018. Gene drive inhibition by the anti-CRISPR proteins AcrIIA2 and AcrIIA4 in saccharomyces cerevisiae. *Microbiol U K* **164**:464–474. doi:10.1099/mic.0.000635

Bastock M. 1956. A Gene Mutation Which Changes a Behavior Pattern. *Evolution* **10**:421–439. doi:10.2307/2407002

Bier E. 2022. Gene drives gaining speed. *Nat Rev Genet* **23**:5–22. doi:10.1038/s41576-021-00386-0

Bishop AL, López Del Amo V, Okamoto EM, Bodai Z, Komor AC, Gantz VM. 2022. Double-tap gene drive uses iterative genome targeting to help overcome resistance alleles. *Nat Commun* **13**:2595. doi:10.1038/s41467-022-29868-3

Burt A. 2003. Site-specific selfish genes as tools for the control and genetic engineering of natural populations. *Proc R Soc Lond B Biol Sci* **270**:921–928. doi:10.1098/rspb.2002.2319

Carballar-Lejarazú R, Ogaugwu C, Tushar T, Kelsey A, Pham TB, Murphy J, Schmidt H, Lee Y, Lanzaro GC, James AA. 2020. Next-generation gene drive for population modification of the malaria vector mosquito, *Anopheles gambiae*. *Proc Natl Acad Sci* **117**:22805–22814. doi:10.1073/pnas.2010214117

Chae D, Lee J, Lee N, Park K, Moon SJ, Kim HH. 2020. Chemical Controllable Gene Drive in Drosophila. *ACS Synth Biol* **9**:2362–2377. doi:10.1021/acssynbio.0c00117

Chae K, Dawson C, Valentin C, Contreras B, Zapletal J, Myles KM, Adelman ZN. 2022. Engineering a self-eliminating transgene in the yellow fever mosquito, Aedes aegypti. *PNAS Nexus* **1**:pgac037. doi:10.1093/pnasnexus/pgac037

Champer J, Liu J, Oh SY, Reeves R, Luthra A, Oakes N, Clark AG, Messer PW. 2018. Reducing resistance allele formation in CRISPR gene drive. *Proc Natl Acad Sci* **115**:5522–5527. doi:10.1073/pnas.1720354115

Champer J, Reeves R, Oh SY, Liu C, Liu J, Clark AG, Messer PW. 2017. Novel CRISPR/Cas9 gene drive constructs reveal insights into mechanisms of resistance allele formation and drive efficiency in genetically diverse populations. *PLoS Genet* **13**:e1006796. doi:10.1371/journal.pgen.1006796

Champer J, Yang E, Lee E, Liu J, Clark AG, Messer PW. 2020. A CRISPR homing gene drive targeting a haplolethal gene removes resistance alleles and successfully spreads through a cage population. *Proc Natl Acad Sci U S A* **117**:24377–24383. doi:10.1073/pnas.2004373117

Courtier-Orgogozo V, Morizot B, Boëte C. 2017. Agricultural pest control with CRISPR-based gene drive: time for public debate. *EMBO Rep* **18**:878–880. doi:10.15252/embr.201744205

Curtis CF. 1968. Possible Use of Translocations to fix Desirable Genes in Insect Pest Populations. *Nature* **218**:368–369. doi:10.1038/218368a0

Deredec A, Burt A, Godfray HCJ. 2008. The Population Genetics of Using Homing Endonuclease Genes in Vector and Pest Management. *Genetics* **179**:2013–2026. doi:10.1534/genetics.108.089037

DiCarlo JE, Chavez A, Dietz SL, Esvelt KM, Church GM. 2015. Safeguarding CRISPR-Cas9 gene drives in yeast. *Nat Biotechnol* **33**:1250–1255. doi:10.1038/nbt.3412

Drapeau MD, Cyran SA, Viering MM, Geyer PK, Long AD. 2006. A cis-regulatory sequence within the yellow locus of Drosophila melanogaster required for normal male mating success. *Genetics* **172**:1009–1030. doi:10.1534/genetics.105.045666

Esvelt KM, Smidler AL, Catteruccia F, Church GM. 2014. Concerning RNA-guided gene drives for the alteration of wild populations. *eLife* **3**:1–21. doi:10.7554/eLife.03401
Gantz VM, Bier E. 2016. The dawn of active genetics. *BioEssays* **38**:50–63. doi:10.1002/bies.201500102
Gantz VM, Bier E. 2015. The mutagenic chain reaction: A method for converting heterozygous to homozygous mutations. *Science* **348**:442–444. doi:10.1126/science.aaa5945
Gloor GB, Preston CR, Johnson-Schlitz DM, Nassif NA, Phillis RW, Benz WK, Robertson HM, Engels WR. 1993. Type I Repressors of P Element Mobility. *Genetics* **135**:81.
Grunwald HA, Gantz VM, Poplawski G, Xu XRS, Bier E, Cooper KL. 2019. Super-Mendelian inheritance mediated by CRISPR–Cas9 in the female mouse germline. *Nature*. doi:10.1038/s41586-019-0875-2
Guichard A, Haque T, Bobik M, Xu X-RS, Klanseck C, Kushwah RBS, Berni M, Kaduskar B, Gantz VM, Bier E. 2019. Efficient allelic-drive in Drosophila. *Nat Commun* **10**:1640. doi:10.1038/s41467-019-09694-w
Hammond A, Galizi R, Kyrou K, Simoni A, Siniscalchi C, Katsanos D, Gribble M, Baker D, Marois E, Russell S, Burt A, Windbichler N, Crisanti A, Nolan T. 2016. A CRISPR-Cas9 gene drive system targeting female reproduction in the malaria mosquito vector Anopheles gambiae. *Nat Biotechnol* **34**:78–83. doi:10.1038/nbt.3439
Hammond A, Karlsson X, Morianou I, Kyrou K, Beaghton A, Gribble M, Kranjc N, Galizi R, Burt A, Crisanti A, Nolan T. 2021. Regulating the expression of gene drives is key to increasing their invasive potential and the mitigation of resistance. *PLOS Genet* **17**:e1009321. doi:10.1371/journal.pgen.1009321
Hammond AM, Kyrou K, Bruttini M, North A, Galizi R, Karlsson X, Kranjc N, Carpi FM, D'Aurizio R, Crisanti A, Nolan T. 2017. The creation and selection of mutations resistant to a gene drive over multiple generations in the malaria mosquito. *PLoS Genet* **13**:e1007039. doi:10.1371/journal.pgen.1007039
Hum YF, Jinks-Robertson S. 2017. Mitotic gene conversion tracts associated with repair of a defined double-strand break in saccharomyces cerevisiae. *Genetics* **207**:115–128. doi:10.1534/genetics.117.300057
Jinek M, Chylinski K, Fonfara I, Hauer M, Doudna JA, Charpentier E. 2012. A Programmable Dual-RNA-Guided DNA Endonuclease in Adaptive Bacterial Immunity. *Science* **337**:816–821. doi:10.1126/science.1225829
Jiricny J. 2006. The multifaceted mismatch-repair system. *Nat Rev Mol Cell Biol 2006 75* **7**:335–346. doi:10.1038/nrm1907
Kandul NP, Liu J, Bennett JB, Marshall JM, Akbari OS. 2021. A confinable home-and-rescue gene drive for population modification. *eLife* **10**:e65939. doi:10.7554/eLife.65939
Kyrou K, Hammond AM, Galizi R, Kranjc N, Burt A, Beaghton AK, Nolan T, Crisanti A. 2018. A CRISPR–Cas9 gene drive targeting doublesex causes complete population suppression in caged Anopheles gambiae mosquitoes. *Nat Biotechnol* **36**:1062–1066. doi:10.1038/nbt.4245
Leitschuh CM, Kanavy D, Backus GA, Valdez RX, Serr M, Pitts EA, Threadgill D, Godwin J. 2018. Developing gene drive technologies to eradicate invasive rodents from islands. *J Responsible Innov* **5**:S121–S138. doi:10.1080/23299460.2017.1365232
Li M, Yang T, Kandul NP, Bui M, Gamez S, Raban R, Bennett J, Sánchez C HM, Lanzaro GC, Schmidt H, Lee Y, Marshall JM, Akbari OS. 2020. Development of a confinable gene drive system in the human disease vector Aedes aegypti. *eLife* **9**. doi:10.7554/eLife.51701
Lin C-C, Potter CJ. 2016. Non-Mendelian Dominant Maternal Effects Caused by CRISPR/Cas9 Transgenic Components in Drosophila melanogaster. *G3 GenesGenomesGenetics* **6**:3685–3691. doi:10.1534/g3.116.034884
López Del Amo V, Bishop AL, Sánchez C HM, Bennett JB, Feng X, Marshall JM, Bier E, Gantz VM. 2020. A

transcomplementing gene drive provides a flexible platform for laboratory investigation and potential field deployment. *Nat Commun* **11**:1–12. doi:10.1038/s41467-019-13977-7

Markstein M, Pitsouli C, Villalta C, Celniker SE, Perrimon N. 2008. Exploiting position effects and the gypsy retrovirus insulator to engineer precisely expressed transgenes. *Nat Genet* **40**:476–483. doi:10.1038/ng.101

Marshall JM, Akbari OS. 2018. Can CRISPR-Based Gene Drive Be Confined in the Wild? A Question for Molecular and Population Biology. *ACS Chem Biol* **13**:424–430. doi:10.1021/acschembio.7b00923

Massey JH, Chung D, Siwanowicz I, Stern DL, Wittkopp PJ. 2019. The yellow gene influences Drosophila male mating success through sex comb melanization. *eLife* **8**:e49388. doi:10.7554/eLife.49388

Montenegro de Wit M. 2019. Gene driving the farm: who decides, who owns, and who benefits? *Agroecol Sustain Food Syst* **43**:1054–1074. doi:10.1080/21683565.2019.1591566

NASEM. 2016. Gene Drives on the Horizon: Advancing Science, Navigating Uncertainty, and Aligning Research with Public Values. National Academies Press. doi:10.17226/23405

Oberhofer G, Ivy T, Hay BA. 2021. Split versions of Cleave and Rescue selfish genetic elements for measured self limiting gene drive. *PLOS Genet* **17**:e1009385. doi:10.1371/JOURNAL.PGEN.1009385

Raban RR, Marshall JM, Akbari OS. 2020. Progress towards engineering gene drives for population control. *J Exp Biol* **223**:jeb208181. doi:10.1242/jeb.208181

Rode NO, Courtier-Orgogozo V, Débarre F. 2020. Can a population targeted by a CRISPR-based homing gene drive be rescued? *G3 Genes Genomes Genet* **10**:3403–3415. doi:10.1534/g3.120.401484

Rong YS, Golic KG. 2000. Gene targeting by homologous recombination in Drosophila. *Science* **288**:2013–8. doi:10.1126/science.288.5473.2013

Scialo F, Sriram A, Stefanatos R, Sanz A. 2016. Practical Recommendations for the Use of the GeneSwitch Gal4 System to Knock-Down Genes in Drosophila melanogaster. *PLOS ONE* **11**:e0161817. doi:10.1371/journal.pone.0161817

Scott MJ, Gould F, Lorenzen M, Grubbs N, Edwards O, O'Brochta D. 2018. Agricultural production: assessment of the potential use of Cas9-mediated gene drive systems for agricultural pest control. *J Responsible Innov* **5**:S98–S120. doi:10.1080/23299460.2017.1410343

Shen P, Huang HV. 1986. Homologous recombination in Escherichia coli: dependence on substrate length and homology. *Genetics* **112**:441–57.

Sturtevant AH. 1915. Experiments on sex recognition and the problem of sexual selection in Drosophilia. *J Anim Behav* **5**:351–366. doi:10.1037/h0074109

Szostak J, Orr-Weaver T, Rothstein R, Stahl F. 1983. The double-strand-break repair model for recombination. *Cell* **33**:25–35. doi:10.1016/0092-8674(83)90331-8

Tanaka H, Stone HA, Nelson DR. 2017. Spatial gene drives and pushed genetic waves. *Proc Natl Acad Sci* **114**:8452–8457. doi:10.1073/pnas.1705868114

Taxiarchi C, Beaghton A, Don NI, Kyrou K, Gribble M, Shittu D, Collins SP, Beisel CL, Galizi R, Crisanti A. 2021. A genetically encoded anti-CRISPR protein constrains gene drive spread and prevents population suppression. *Nat Commun* **12**:3977. doi:10.1038/s41467-021-24214-5

Unckless RL, Clark AG, Messer PW. 2017. Evolution of resistance against CRISPR/Cas9 gene drive. *Genetics* **205**:827–841. doi:10.1534/genetics.116.197285

Unckless RL, Messer PW, Connallon T, Clark AG. 2015. Modeling the Manipulation of Natural Populations by the Mutagenic Chain Reaction. *Genetics* **201**:425–431. doi:10.1534/genetics.115.177592

Wu B, Luo L, Gao XJ. 2016. Cas9-triggered chain ablation of cas9 as a gene drive brake. *Nat Biotechnol* **34**:137–138. doi:10.1038/nbt.3444

Xiao C, Qiu S, Robertson RM. 2017. The white gene controls copulation success in Drosophila

melanogaster. *Sci Rep* **7**:7712. doi:10.1038/s41598-017-08155-y
Xu XRS, Bulger EA, Gantz VM, Klanseck C, Heimler SR, Auradkar A, Bennett JB, Miller LA, Leahy S, Juste SS, Buchman A, Akbari OS, Marshall JM, Bier E. 2020. Active Genetic Neutralizing Elements for Halting or Deleting Gene Drives. *Mol Cell* **80**:246-262.e4. doi:10.1016/j.molcel.2020.09.003
Zapletal J, Najmitabrizi N, Erraguntla M, Lawley MA, Myles KM, Adelman ZN. 2021. Making gene drive biodegradable. *Philos Trans R Soc B Biol Sci* **376**. doi:10.1098/rstb.2019.0804

Acknowledgements

We thank Dr. Omar Akbari at the University of California San Diego for reviewing the manuscript and providing helpful comments. The graphic cartoon art of the *Drosophila* head used in the figures was downloaded as a vector file from Zenodo (Somers, 2020) and modified.

Funding

The National Institute for Allergy and Infectious Diseases and Defense Advanced Research Projects Agency supported this work through grants AI119081, AI148787 and HR0011-16-2-0036. The funders had no role in study design, data collection and analysis, decision to publish, or preparation of the manuscript.

Author Contributions

KM, ZA, and PC conceptualized the study; PC and KM designed the research; PC, RM, and KM performed the experiments and analyzed the data; JZ, MN, and PC developed and ran the model simulations; PC and KM wrote the manuscript; KM, PC, and ZA revised the manuscript. All authors approved the final manuscript.

Data Availability

The data for this study are provided in the supplementary information/source data file provided with this paper.

Code Availability

All files pertaining to the generation of equations, model execution, and plotting of charts can be found at the following link: https://github.com/mln27/Python-CRISPR1-1-SEM-gene-drive

Competing Interests

KMM and ZNA are inventors on US provisional patent application PCT/US2021/041951, submitted by Texas A&M University, which covers vector constructs that are pre-programmed to self-eliminate or self-remove at a predetermined time, and methods of making the same. PRC, JZ, RDM, MLNM declare no competing interests.

Figure Legends

Figure 1. Repeat Mediated Excision of a Transgene (ReMET). (**A**) Schematic depicting ReMET constructs (*in trans* and *in cis*) and their removal by SSA. The yReMET construct (shaded in purple) contains EGFP and RFP under the control of independent 3xP3 promoters, and an endonuclease, I-*Sce*I, under the control of UASp. The construct is flanked by a pair of direct repeats (DR) that are 30, 250, or 500 bp in length. Insertion of the ReMET construct into exon 2 of the *yellow* gene creates a null allele that produces yellow body pigmentation with both green and red eye fluorescence. I-*Sce*I may be expressed by crossing with a nos-Gal4 driver line (*in trans*), or by expression from a RU486-inducible nos-GS (*in cis*). Expression of I-*Sce*I generates a DSB at the recognition site (I-Site) located between the DRs, excising the transgene and one of the direct repeats through SSA. SSA-mediated removal of the transgene restores wild type body pigmentation (brown body) with simultaneous loss of eye-specific fluorescence. The engineered synonymous mutation TGG->TcG provides molecular confirmation of ReMET. (**B**) Schematic depicting *in trans* mating scheme and phenotypic identification of germline SSA events. Percentage of F2 progeny exhibiting SSA-mediated transgene excision by DR length, confirmed through wild type body color and the presence of the engineered silent mutation (TcG). Each dot represents a separate pair-mated cross (mean and ± s.e.m. are shown in red), with the sex of the F1 yReMET fly shown above. (**C**) Schematic depicting *in cis* mating scheme and phenotypic identification of germline SSA events. Percentage of F1 progeny exhibiting SSA-mediated transgene excision by DR length, confirmed through wild type body color and the presence of the engineered silent mutation (TcG). Each dot represents a separate pair-mated cross (mean and ± s.e.m. are shown in red). UTR = Untranslated Region; DSB = Double Strand Break; UASp = Upstream Activation Sequence with pTransposase promoter; EGFP = Enhanced Green Fluorescent Protein; RFP = Red Fluorescent Protein; SSA = Single Strand Annealing; p values = * < 0.5, ** < 0.01, *** < 0.001, and **** < 0.0001; ns = not significant.

Figure 2. Repeat Mediated Excision of a Drive Element (ReMEDE). (**A**) Schematic depicting ReMEDE construct and its removal by SSA. The construct contains components of both the ReMET (*in cis*) and yMCR constructs, including those essential for genetic drive, i.e., Cas9 (under the control of a vasa promoter) and an sgRNA targeting exon 2 of the wild type *yellow* gene (under the control of a U6:3 promoter). The core gene drive components are flanked by a pair of gypsy insulator sequences (gyp). Expression of I-*Sce*I from nos-GS generates a DSB at the I-Site located between the DR, resulting in excision of the transgene by SSA, which is confirmed by the presence of an engineered silent mutation, TcG. (**B**) Schematic depicting maternal inheritance of an X-linked transgene. Mendelian inheritance of the recessive X-linked *yellow* mutant allele (generated through transgene insertion) from a heterozygous mother produces wild type body pigmentation in heterozygous female progeny, but a yellow phenotype in the hemizygous male progeny. Maternal inheritance of a transgene expressing GFP inserted into the yMCR and yReMEDE sgRNA target site. The percentage of progeny exhibiting GFP+ or GFP- phenotypes in replicate pair-mated crosses of heterozygous F1 females with wild type males are shown. Each dot represents a separate pair-mated cross. (**C**) When the transgene is a gene drive, multiple Mendelian outcomes, as well as non-Mendelian exceptions, are possible. When the heterozygous drive allele present in the F1 female creates a DSB in its wild type counterpart, HR-mediated repair results in allelic conversion increasing transgene frequency and generating super-Mendelian patterns of inheritance (green eyes). However, DSBs repaired by the more erroneous NHEJ may generate either recessive out-of-frame null alleles, or dominant in-frame alleles. Non-Mendelian exceptions may also occur due to the zygotic perdurance of maternally deposited

Cas9 RNPs generating DSBs in the paternal wild type alleles, resulting in either in-frame or out-of-frame repair, which generates the additional possibilities illustrated. Maternal inheritance of either the yMCR or yReMEDE alleles. The percentage of progeny exhibiting drive, wild type, or yellow phenotypes in replicate pair-mated crosses of heterozygous F1 females with wild type males are shown. Each dot represents a separate pair-mated cross (**D**) Sequencing results for the *yellow* gene in wild type progeny of pair-mated crosses involving F1 yMCR females and wild type males, sorted by parental lineage. (**E**) Sequencing results for the *yellow* gene in wild type progeny of pair-mated or *en masse* crosses involving F1 yReMEDE females and wild type males, sorted by parental lineage. The sequence present at the PAM site is shown, as well as the DNA repair pathway presumed to be responsible for its generation. NHEJ = Non-Homologous End Joining; SSA = Single Strand Annealing; p values = ** < 0.01, *** < 0.001, and **** < 0.0001; ns = not significant.

Figure 3. Population cage trials with either yMCR or yReMEDE gene drives. Graphs show the prevalence of drive and wild type phenotypes (exhibited by both male and female flies) over time in cage trials involving either (**A)** yMCR (green lines) or (**B**) yReMEDE (red lines). Solid lines represent single replicate cages, while dotted lines represent mean values.

Figure 4. Stochastic modeling of ReMEDE technology performance in a population. All models were run with the X-linked inheritance module that outputs allele frequencies in two plots. One plot shows five simulations randomly selected from 100 independently run simulations (Left), while the other plot shows the mean of all simulations (Right). All simulations were run in a low threshold release scenario (10% of flies containing a drive allele) and in the absence of mating costs. (**A**) Simulation of yMCR gene drive, generating resistant alleles at frequencies comparable to those observed experimentally, but in the absence of reduced courtship activity and mating success. (**B**) Simulation of yReMEDE where SSA converts approximately 10% of the drive alleles to wild type. The generation of resistant alleles and other parameters occur at frequencies comparable to those shown in A. (**C**) Simulation of a gene drive containing ReMEDE that generates no resistance alleles, but with SSA converting approximately 30% of the drive alleles to wild type; a quarter of which retain the engineered resistant allele in the PAM site (ε = 0.25). (**D**) Simulation of a gene drive containing ReMEDE that generates no resistance alleles, but with SSA converting approximately 10% of the drive alleles to wild type, all of which retain the engineered resistant allele in the PAM site (ε = 1) (**E**) Simulation of a gene drive containing ReMEDE that generates no resistance alleles, but with SSA converting a modest ~5% of the drive alleles to wild type, a quarter of which retain the engineered resistant allele in the PAM site (ε = 0.25).

Figure 1

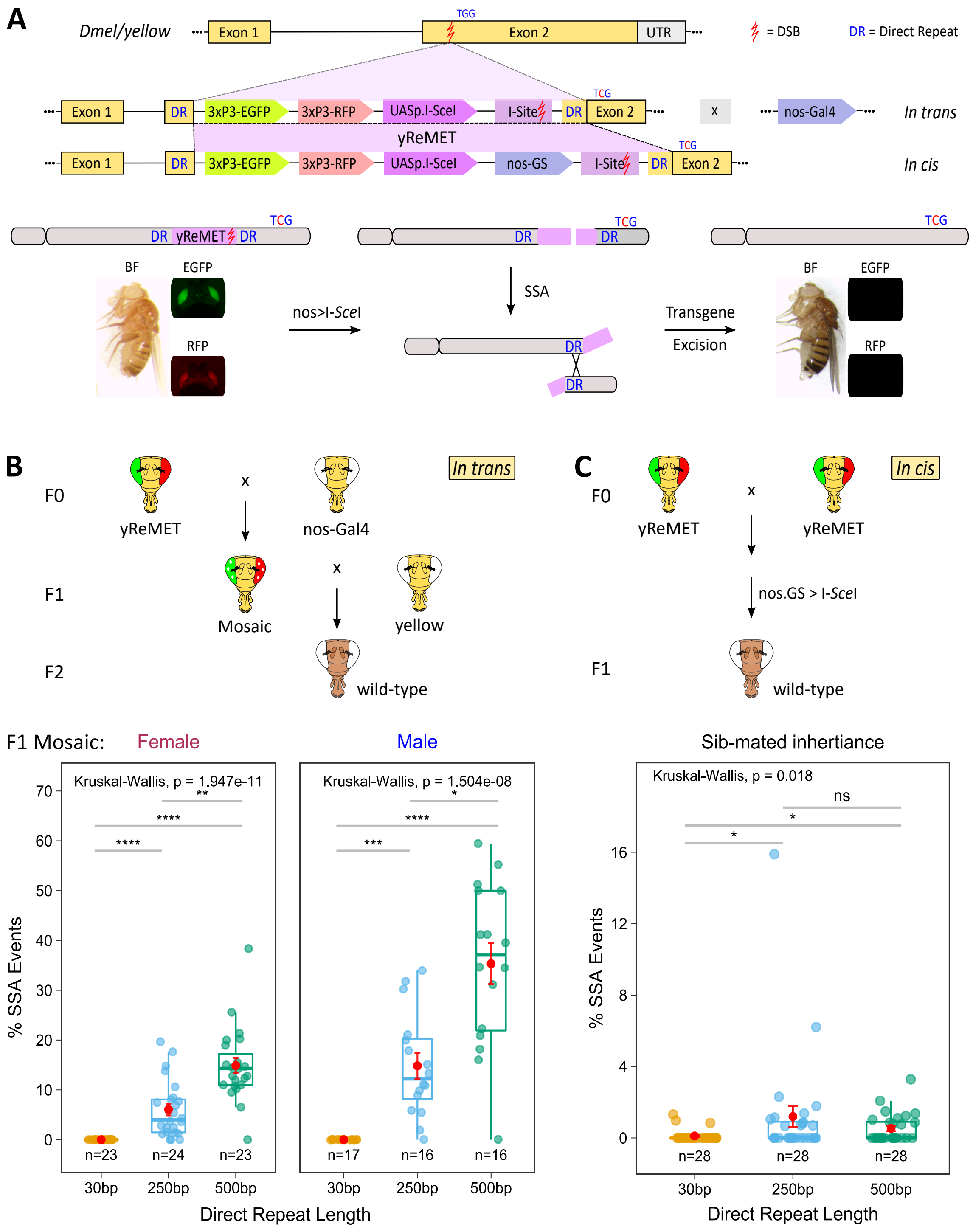
A
Dmel/yellow
Exon 1
TGG
Exon 2
UTR
= DSB
DR = Direct Repeat
TCG
In trans
In cis
3xP3-EGFP
3xP3-RFP
UASp.I-SceI
I-Site
nos-Gal4
nos-GS
yReMET
DR
BF
EGFP
RFP
nos>I-SceI
SSA
Transgene
Excision
B
In trans
F0
F1
F2
yReMET
x
nos-Gal4
Mosaic
yellow
wild-type
C
In cis
nos.GS > I-SceI
F1 Mosaic:
Female
Male
Kruskal-Wallis, p = 1.947e-11
Kruskal-Wallis, p = 1.504e-08
% SSA Events
n=23
n=24
n=17
n=16
30bp
250bp
500bp
Direct Repeat Length
Sib-mated inheritance
Kruskal-Wallis, p = 0.018
ns
n=28

Figure 2

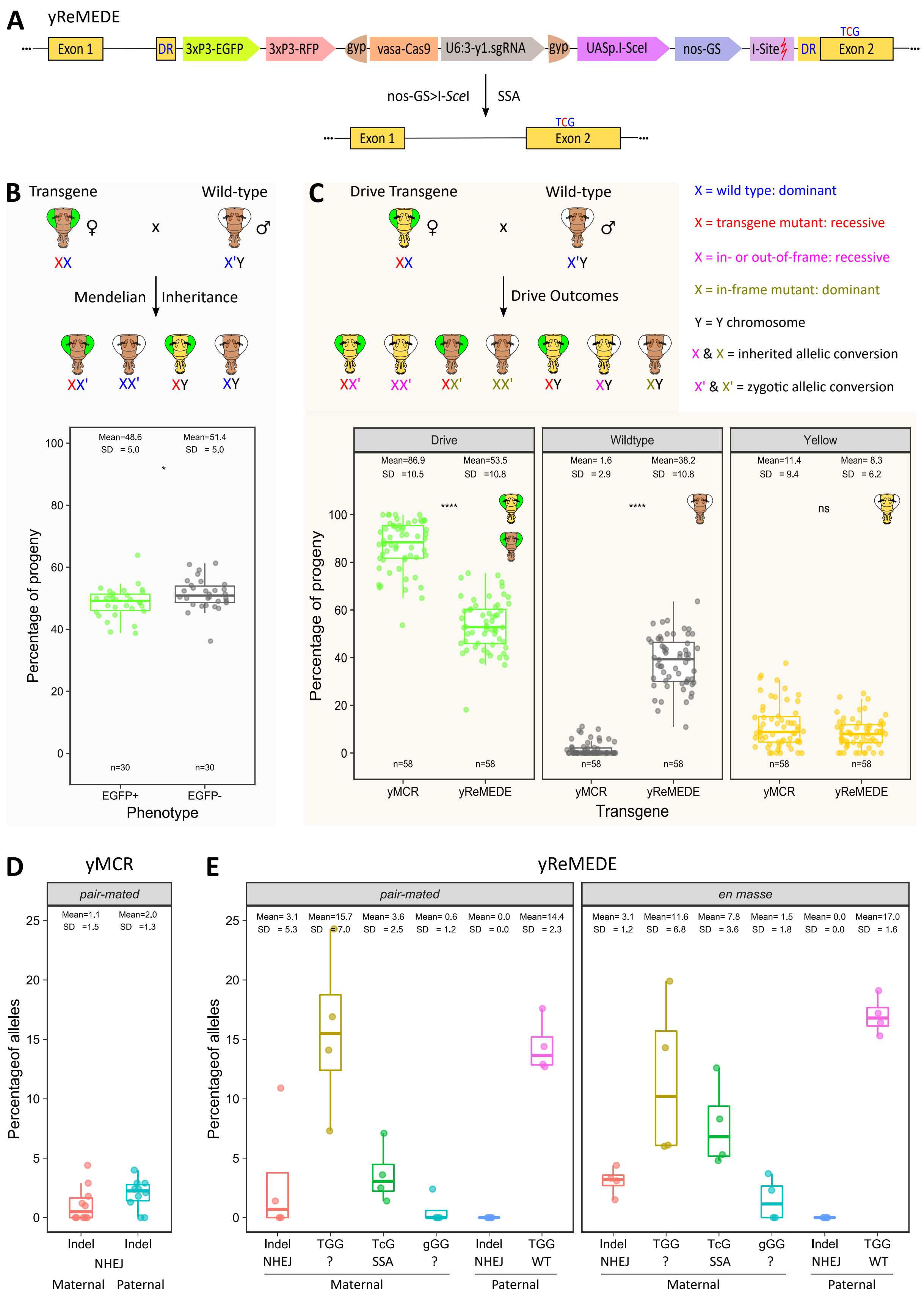

A
yReMEDE
Exon 1
DR
3xP3-EGFP
3xP3-RFP
gyp
vasa-Cas9
U6:3-y1.sgRNA
gyp
UASp.I-SceI
nos-GS
I-Site
DR
TCG
Exon 2
nos-GS>I-SceI
SSA
Exon 1
TCG
Exon 2
B
Transgene
Wild-type
XX
X'Y
Mendelian
Inheritance
XX'
XX'
XY
XY
Mean=48.6 SD = 5.0
Mean=51.4 SD = 5.0
*
Percentage of progeny
n=30
n=30
EGFP+
EGFP-
Phenotype
C
Drive Transgene
Wild-type
XX
X'Y
Drive Outcomes
XX'
XX'
XX'
XX'
XY
XY
XY
X = wild type: dominant
X = transgene mutant: recessive
X = in- or out-of-frame: recessive
X = in-frame mutant: dominant
Y = Y chromosome
X & X = inherited allelic conversion
X' & X' = zygotic allelic conversion
Drive
Wildtype
Yellow
Mean=86.9 SD =10.5
Mean=53.5 SD =10.8
Mean= 1.6 SD = 2.9
Mean=38.2 SD =10.8
Mean=11.4 SD = 9.4
Mean= 8.3 SD = 6.2
****
****
ns
Percentage of progeny
n=58
yMCR
yReMEDE
Transgene
D
yMCR
pair-mated
Mean=1.1 SD =1.5
Mean=2.0 SD =1.3
Percentageof alleles
Indel
Indel
NHEJ
Maternal
Paternal
E
yReMEDE
pair-mated
en masse
Mean= 3.1 SD = 5.3
Mean=15.7 SD =7.0
Mean= 3.6 SD = 2.5
Mean= 0.6 SD = 1.2
Mean= 0.0 SD = 0.0
Mean=14.4 SD = 2.3
Mean= 3.1 SD = 1.2
Mean=11.6 SD = 6.8
Mean= 7.8 SD = 3.6
Mean= 1.5 SD = 1.8
Mean= 0.0 SD = 0.0
Mean=17.0 SD = 1.6
Indel NHEJ
TGG ?
TcG SSA
gGG ?
Indel NHEJ
TGG WT
Maternal
Paternal

Figure 3

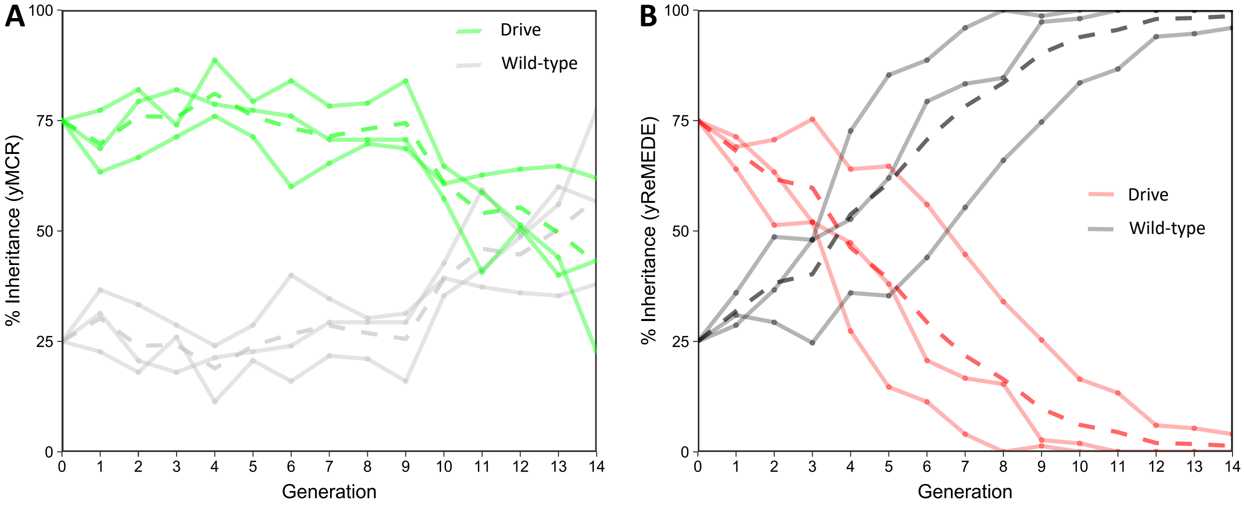
A
B
Drive
Wild-type
% Inheritance (yMCR)
% Inheritance (yReMEDE)
Generation
0
25
50
75
100
0
1
2
3
4
5
6
7
8
9
10
11
12
13
14

Figure 4

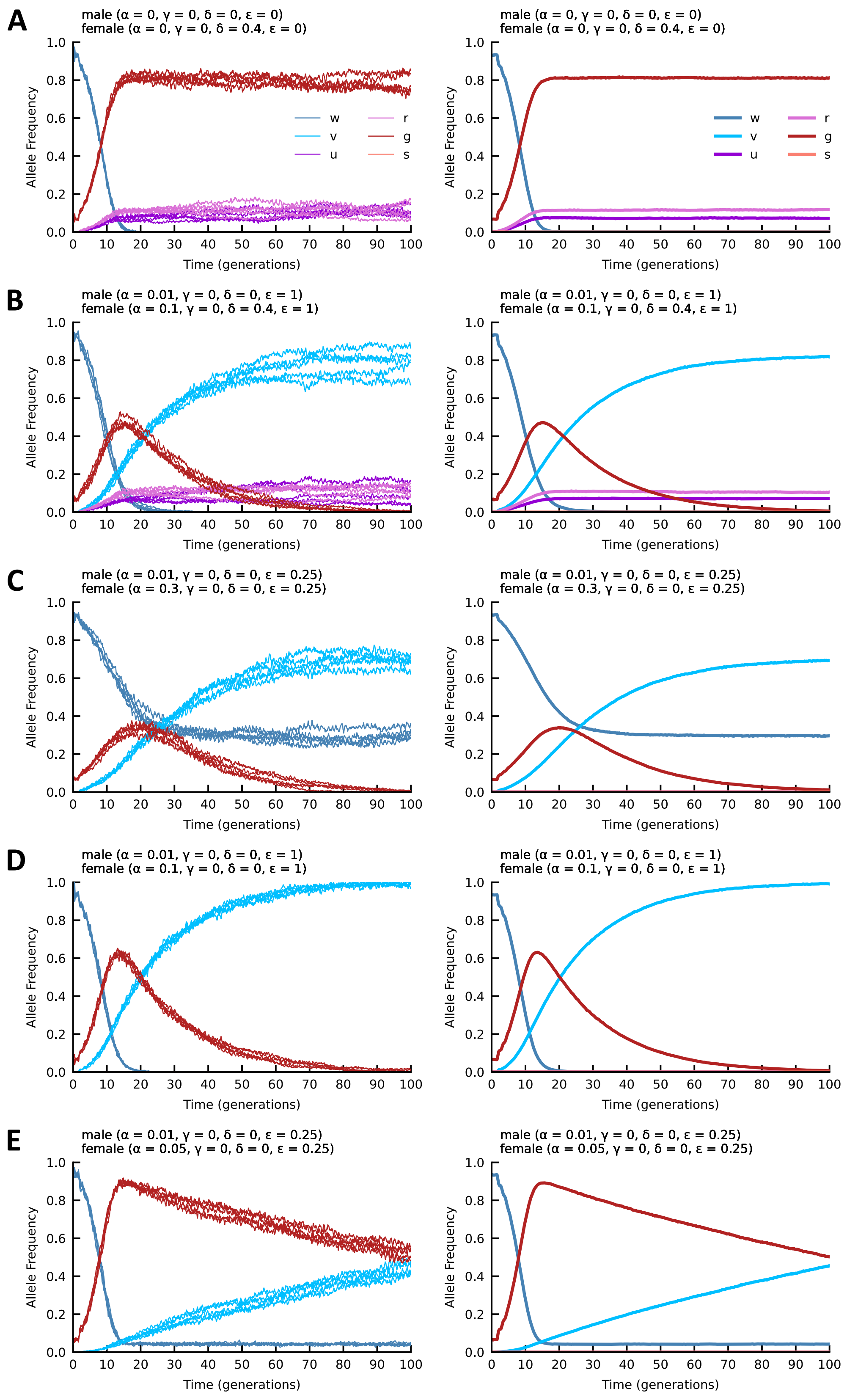

w = wild-type | u = in-frame mutant | v = transgene excised, wild-type | α = rate of SSA | δ = rate of 'u' formation

g = gene drive | r = out-of-frame mutant | s = excision resistant gene drive | γ = rate of SSA-resistance | ε = rate of 'v' persistence

Supplementary Methods

Courtship Assays

A construct containing EGFP under the control of a 3xP3 promoter was inserted into exon 2 of the *D. melanogaster yellow* gene at the drive element (yMCR or yReMEDE) target site, generating a null mutation. The courtship behavior of yellow flies was then assessed in both noncompetitive and competitive mating crosses with wild-type (w^{1118}) flies conducted in specially designed fly arenas, as described previously (Boutros et al., 2017). Briefly, female and male flies were gently aspirated into the circular arenas of the apparatus. Mating behavior was then captured by video over a five-hour duration. Any copulation between a male and female fly observed during the video analysis was subsequently scored as a successful mating. Courtship studies were conducted at room temperature over a similar time range during the day (12 hour light/dark cycle) with a standard corn-meal/oatmeal based medium.

Population Studies

All multi-generational cage trials were conducted at 25 C (12 hour light/dark cycle) in standard 250 mL rearing bottles containing a standard corn-meal/oatmeal based medium. For each genotype, bottles were seeded with equal numbers of unmated male and female flies. After 5 days, this initial generation (n) of flies was removed from the bottles. Subsequent generations (n+1) were then collected and randomly divided into two equal pools. One pool was seeded into a fresh cage (n+2), while the other was scored and further analyzed.

Modeling

Mathematical modeling was performed with methods that have been described previously (Chae et al., 2022), but with the following modifications. In this study, the continuous addition of offspring into the adult mating pool was replaced with distinct generations. A modified version of the widely used lumped age-class model of mosquito life-history (Hancock and Godfray, 2007; Deredec et al., 2011; Sánchez C. et al., 2020) was incorporated to more fully account for the fly's four different states: egg, larvae, pupae, and adult (male and female). In order to adapt the model to *Drosophila,* the following parameters were assigned to the fly's life-history stages. Egg production per day (day[-1]) = 100; duration of egg stage (days) =1; duration of larval stage (days) = 4; duration of pupa stage (days) = 5 (Fernández-Moreno et al., 2007; Ong et al., 2015; Mołoń et al., 2020); daily mortality risk of egg stage (day[-1]) = 0.15; daily mortality risk of pupa stage (day[-1]) = 0.15 (Durdevic et al., 2018; Dylan Shropshire et al., 2018; Layton et al., 2019); daily mortality risk of adult stage (day[-1]) = 0.263; and daily population growth rate (day[-1]) = 1.196 (Horváth et al., 2016). We also developed a stochastic version of the previously described methods where genotype-specific daily egg production follows a multivariate Poisson distribution informed by parental genotypes and the "Equation Generation" module. Survival and death events follow binomial distributions at the population level.

<u>Equation Generation</u>

Equations for determining the number of offspring with each genotype were derived as described previously (Chae et al., 2022), but with the following modifications. An additional factor of epsilon (ε) was incorporated into the equations to capture the rate of 'v' persistence. Where 'v' represents a wild-type allele resulting from the excision of a previously inserted drive element, but engineered to contain a Cas9 target site that is resistant to re-cutting. The formula 1-ε was then used to represent a 'v' allele that has been converted into the original wild-type allele (w) sequence by mismatch repair. In order to simulate

the X-linked inheritance of the ReMEDE drive construct, the probability equations for the heterogametic males were simplified to include a single allele. All possible combinations of the two female alleles and one male allele were diagramed and probabilities computed for each.

Model Accessibility

Details regarding the execution of modeling methods and interpretation of results have been provided previously (Chae et al., 2022). All files pertaining to the generation of equations, model execution, and plotting of charts can be found at the following link: https://github.com/mln27/Python-CRISPR1-1-SEM-gene-drive

Supplementary References

Boutros, C. L., Miner, L. E., Mazor, O., and Zhang, S. X. (2017). Measuring and Altering Mating Drive in Male Drosophila melanogaster. *J. Vis. Exp.*, e55291. doi:10.3791/55291.

Chae, K., Dawson, C., Valentin, C., Contreras, B., Zapletal, J., Myles, K. M., et al. (2022). Engineering a self-eliminating transgene in the yellow fever mosquito, Aedes aegypti. *PNAS Nexus* 1. doi:10.1093/PNASNEXUS/PGAC037.

Deredec, A., Godfray, H. C. J., and Burt, A. (2011). Requirements for effective malaria control with homing endonuclease genes. *Proc. Natl. Acad. Sci. U. S. A.* 108. doi:10.1073/PNAS.1110717108/SUPPL_FILE/APPENDIX.PDF.

Durdevic, Z., Pillai, R. S., and Ephrussi, A. (2018). Transposon silencing in the Drosophila female germline is essential for genome stability in progeny embryos. *Life Sci. Alliance* 1. doi:10.26508/LSA.201800179.

Dylan Shropshire, J., On, J., Layton, E. M., Zhou, H., and Bordenstein, S. R. (2018). One prophage WO gene rescues cytoplasmic incompatibility in Drosophila melanogaster. *Proc. Natl. Acad. Sci. U. S. A.* 115, 4987–4991. doi:10.1073/PNAS.1800650115/SUPPL_FILE/PNAS.1800650115.SD01.XLSX.

Fernández-Moreno, M. A., Farr, C. L., Kaguni, L. S., and Garesse, R. (2007). Drosophila melanogaster as a Model System to Study Mitochondrial Biology. *Methods Mol. Biol.* 372, 33. doi:10.1007/978-1-59745-365-3_3.

Hancock, P. A., and Godfray, H. C. J. (2007). Application of the lumped age-class technique to studying the dynamics of malaria-mosquito-human interactions. *Malar. J.* 6, 1–13. doi:10.1186/1475-2875-6-98/FIGURES/5.

Horváth, B., Betancourt, A. J., and Kalinka, A. T. (2016). A novel method for quantifying the rate of embryogenesis uncovers considerable genetic variation for the duration of embryonic development in Drosophila melanogaster. *BMC Evol. Biol.* 16, 1–14. doi:10.1186/S12862-016-0776-Z.

Layton, E. M., On, J., Perlmutter, J. I., Bordenstein, S. R., and Shropshire, J. D. (2019). Paternal grandmother age affects the strength of Wolbachia-induced cytoplasmic incompatibility in Drosophila melanogaster. *MBio* 10. doi:10.1128/MBIO.01879-19/SUPPL_FILE/MBIO.01879-19-ST002.DOCX.

Mołoń, M., Dampc, J., Kula-Maximenko, M., Zebrowski, J., Mołoń, A., Dobler, R., et al. (2020). Effects of Temperature on Lifespan of Drosophila melanogaster from Different Genetic Backgrounds: Links between Metabolic Rate and Longevity. *Insects 2020, Vol. 11, Page 470* 11, 470.

doi:10.3390/INSECTS11080470.

Ong, C., Yung, L. Y. L., Cai, Y., Bay, B. H., and Baeg, G. H. (2015). Drosophila melanogaster as a model organism to study nanotoxicity. *http://dx.doi.org/10.3109/17435390.2014.940405* 9, 396–403. doi:10.3109/17435390.2014.940405.

Sánchez C., H. M., Wu, S. L., Bennett, J. B., and Marshall, J. M. (2020). MGDrivE: A modular simulation framework for the spread of gene drives through spatially explicit mosquito populations. *Methods Ecol. Evol.* 11, 229–239. doi:10.1111/2041-210X.13318.

Supplementary Figure Legends

Figure S1. Generation of yReMET lines, transgene excision, and characterization of RU486 inducible promoter. (**A**) Schematic depicting the two-step process for the generation of yReMET (*in cis*) transgenic lines. In the first step, a donor plasmid, p-yDR.EGFP, with DRs of varying lengths (30, 250 or 500 bp), identical in sequence to the 3’ end of the 5’ homology arm, were inserted into the *Drosophila melanogaster yellow* gene at a Cas9 target site. The donor construct contained 3xP3-EGFP, which served as a marker of transgenesis, a recognition site for the I-*Sce*I endonuclease (I-Site), a loxP site, and an attP landing site. The resulting yDR transgenic fly lines were also engineered to contain the silent PAM mutation TGG->TcG. In the second step, a donor plasmid, p-ReMET.RFP, containing an attB site was inserted into the yDR lines through ϕC31-mediated recombination. The p-ReMET.RFP donor contained 3xP3-RFP, a loxP site, as well as the nos-GS and UASp.I-*Sce*I components. In another configuration, used for the *in trans* experiments, only the UASp.I-*Sce*I is included and not the nos-GS. Integration of the p-ReMET.RFP plasmid into the yDR lines generated the various yReMET lines containing DRs of different lengths. (**B**) Percentage of F2 progeny exhibiting SSA-mediated transgene excision by DR length and sex, confirmed through the presence of wild type body pigmentation and the engineered silent mutation (TcG). Gold dots represent individual pair-mated crosses of F1 yReMET (*in trans* configuration) flies with wild type flies. Blue dots represent individual pair-mated crosses of F1 yReMET (*in trans* configuration) flies with transgenic nos-Gal4 flies. P-value **** < 0.0001; ns = not significant. (**C**) Percentage of F1 progeny exhibiting SSA-mediated transgene excision by DR length and RU486 concentration, confirmed through the presence of wild type body pigmentation and the engineered silent mutation (TcG). Each dot/triangle represents a separate pair-mated cross (mean and ± s.e.m. are shown in red). (**D**) Images of adult eye pigmentation in F1 progeny resulting from pair-mated crosses of yReMET (*in cis* configuration) flies with flies containing a *white* (w^+) reporter gene with an adjacent I-Site sequence. Images are representative of the average level of mosaicism observed for the given concentration of RU486.

Figure S2. Generation and characterization of yReMEDE line. (**A**) Schematic depicting insertion of p-yMCR.EGFP into the *yellow* gene of *Drosophila melanogaster*. The plasmid p-yMCR.EGFP contains Cas9 (under the control of a vasa promoter), an sgRNA targeting exon 2 of the wild type *yellow* gene (under the control of a U6:3 promoter), and EGFP (under the control of the eye-specific 3xP3 promoter). The primer annealing sites 43 and 44 are located outside the homology arms in order to permit genotyping of target alleles in flies exhibiting a wild type phenotype. (**B**) Schematic depicting the generation of the yReMEDE line through ϕC31-mediated recombination of p-ReMEDE.RFP into the yDR line. The plasmid p-ReMEDE.RFP contains the yMCR drive element flanked by a pair of gypsy insulators (gyp), nos-GS > UASp-I-*Sce*I, a loxP site, and 3xP3-RFP. Primer annealing sites 208 and 16 permit genotyping of the PAM site in the drive allele. (**C**) Maternal and paternal inheritance of drive elements. Percentage of progeny exhibiting scored phenotypes (i.e., body pigmentation and fluorescent eye color) as indicated by cartoon fly heads. Each dot represents a separate pair-mated cross. (**D**) Percentage of progeny exhibiting wild type phenotypes in replicate *en masse* crosses of yReMEDE flies. Each dot represents a separate *en masse* cross (mean and ± s.e.m. are shown in red). (**E**) Percentage of progeny exhibiting wild type phenotypes in replicate *en masse* crosses of yReMEDE flies in the presence of RU486 at the concentrations specified. LHA = Left Homology Arm; RHA = Right Homology Arm; p values = ** < 0.01, *** < 0.001, and **** < 0.0001; ns = not significant.

Figure S3. Matrilineal inheritance of wild type alleles in progeny of females carrying yReMEDE. Mating scheme for generation of F1 yReMEDE females and their F2 progeny. Images of fly heads indicate scored phenotypes of eye fluorescence (white = no marker, green = EGFP, red = RFP) and body color (brown = + or +Δ, yellow = Drive or -Δ). Possible allelic combinations are shown below head images (ReMEDE = Drive, wild type = +, in-frame indel = +Δ, out-of-frame indel = -Δ). Male flies are indicated by the presence of the Y chromosome. Blue boxes enclose genotypes and phenotypes that confirm SSA-mediated transgene excision, regardless of the PAM sequence.

Figure S4. Model for reversion of the engineered TcG PAM sequence to wild-type. The SDSA model appears to be the primary DSB repair mechanism in mitotic cells and is shown for simplicity. Production of Cas9 RNP from the gene drive (donor) allele (shown in green) generates a DSB in the wild type (recipient) allele (shown in red). Resection of the 5' DNA ends produces 3' single-stranded tails. Strand invasion of the donor drive allele by one of the recipient allele's 3' tail results in base pairing of complementary strands generating a tract of heteroduplex DNA (hetDNA). This process displaces the originally duplexed strand forming a displacement (D)-loop. Elongation of the invading 3' end increases the size of the D-loop, eventually producing a sequence that extends beyond the DSB. Dismantling of the D-loop frees the newly synthesized sequence to anneal with the other 3' tail of the recipient allele, generating a new tract of hetDNA with the opposite strand, i.e., SDSA. Mismatches present in the hetDNA are repaired by MMR. Excision followed by resynthesis and ligation repairs the mis-paired bases, restoring the original wild type sequence of the recipient and eliminating heterozygosity at the locus. SDSA = Synthesis Mediated Strand Annealing, DSB = Double Strand Break, RNP = RiboNucleoProtein.

Figure S5. Performance of the yMCR gene drive in a low threshold release scenario. (**A**) Courtship indices calculated for *yellow* mutant (mt) and w^{1118} (wt) flies in the crossing schemes shown. P-values = ** < 0.01, and **** < 0.0001. (**B**) Cage trials with the yMCR gene drive in either a w^{1118} (wt) or *yellow* mutant (mt) population. F1 females or males carrying the yMCR allele initially made up 10% of the total population. Graphs show the prevalence of the drive phenotype (green line) in both male and female progeny over time. Each line represents a separate replicate cage trial. (**C**) Theoretical model of allelic conversion dynamics occurring within the F1 female germline during oogenesis. In gene drive, a DSB occurs independently at each susceptible target allele (w) with a probability of *q*, after which repair occurs by HR with a probability of *p,* or by NHEJ with a probability of *1-p*. HR results in allelic conversion of w to g, while NHEJ produces in-frame alleles (u) with a probability of δ, or out-of-frame alleles (r) with a probability of 1-δ. In the presence of ReMEDE, a second DSB occurs and is repaired by SSA with a probability of α, or by NHEJ with a probability of γ. SSA retains the engineered silent PAM mutation (TcG) with a probability of ε, or TcG reverts back to TGG through MMR with a probability of 1-ε. (**D**) Stochastic modeling of the yMCR gene drive in a wild type population. All models were run with the X-linked inheritance module that outputs allele frequencies in two plots. One plot shows five simulations randomly selected from 100 independently run simulations (Left), while the other plot shows the mean of all simulations (Right). Simulations of the yMCR gene drive were run in a low threshold release scenario (10% of flies containing a drive allele), and generating resistant alleles at frequencies (δ = 0.4) comparable to those observed experimentally. Similarly, mating cost parameters were set at levels consistent with empirically determined values for yellow male (0.97) and female (0.31) flies in a wild type population.

S1

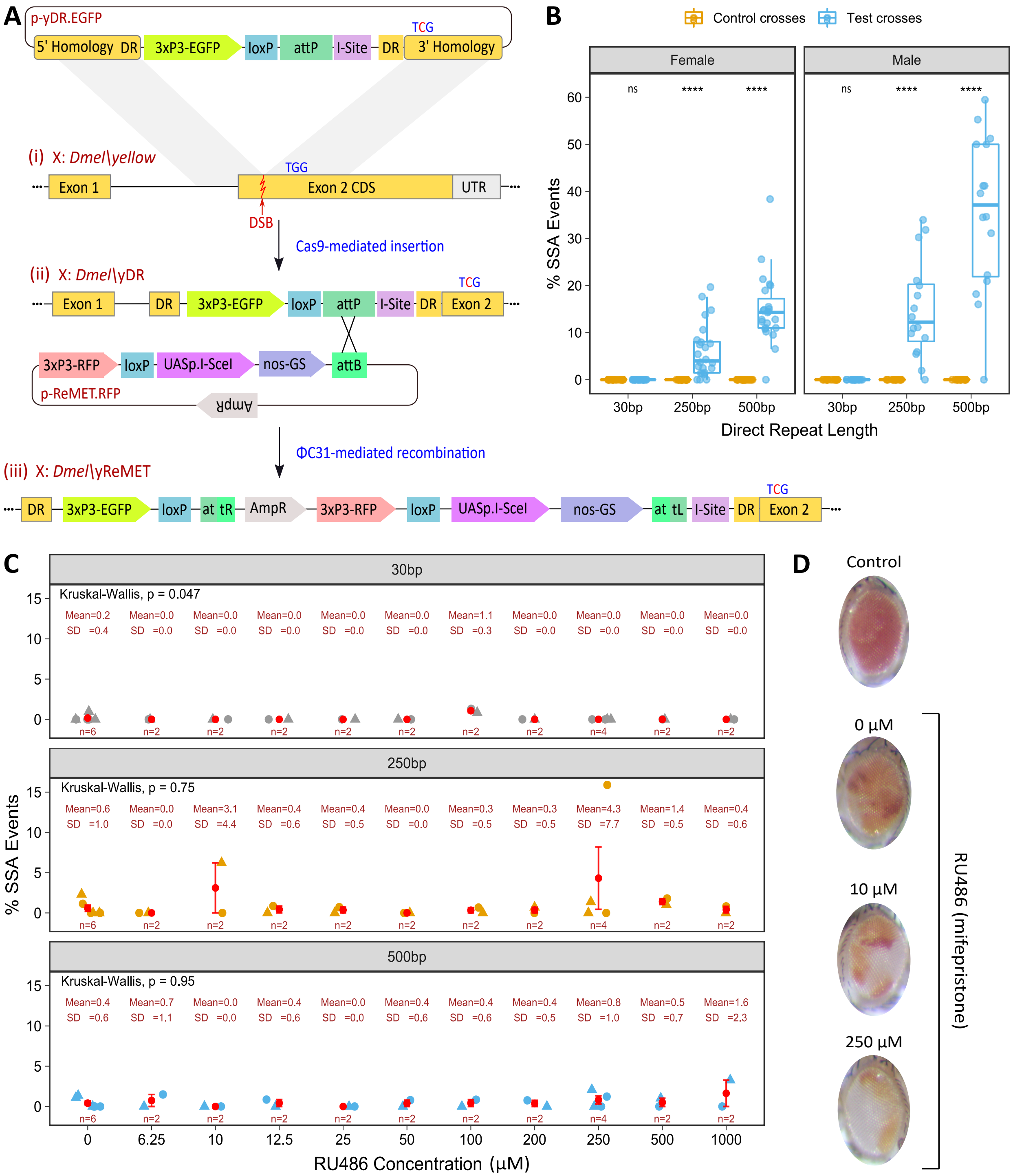
A
p-yDR.EGFP
5' Homology DR
3xP3-EGFP
loxP
attP
I-Site
DR
TCG
3' Homology
(i) X: Dmel\yellow
Exon 1
TGG
Exon 2 CDS
UTR
DSB
Cas9-mediated insertion
(ii) X: Dmel\yDR
Exon 1
DR
3xP3-EGFP
loxP
attP
I-Site
DR
TCG
Exon 2
3xP3-RFP
loxP
UASp.I-SceI
nos-GS
attB
p-ReMET.RFP
AmpR
ΦC31-mediated recombination
(iii) X: Dmel\yReMET
DR
3xP3-EGFP
loxP
at tR
AmpR
3xP3-RFP
loxP
UASp.I-SceI
nos-GS
at tL
I-Site
DR
TCG
Exon 2
B
Control crosses
Test crosses
Female
Male
ns
****
****
ns
****
****
% SSA Events
0
10
20
30
40
50
60
30bp
250bp
500bp
30bp
250bp
500bp
Direct Repeat Length
C
30bp
Kruskal-Wallis, p = 0.047
Mean=0.2 SD =0.4
Mean=0.0 SD =0.0
Mean=0.0 SD =0.0
Mean=0.0 SD =0.0
Mean=0.0 SD =0.0
Mean=0.0 SD =0.0
Mean=1.1 SD =0.3
Mean=0.0 SD =0.0
Mean=0.0 SD =0.0
Mean=0.0 SD =0.0
Mean=0.0 SD =0.0
n=6 n=2 n=2 n=2 n=2 n=2 n=2 n=2 n=4 n=2 n=2
250bp
Kruskal-Wallis, p = 0.75
Mean=0.6 SD =1.0
Mean=0.0 SD =0.0
Mean=3.1 SD =4.4
Mean=0.4 SD =0.6
Mean=0.4 SD =0.5
Mean=0.0 SD =0.0
Mean=0.3 SD =0.5
Mean=0.3 SD =0.5
Mean=4.3 SD =7.7
Mean=1.4 SD =0.5
Mean=0.4 SD =0.6
n=6 n=2 n=2 n=2 n=2 n=2 n=2 n=2 n=4 n=2 n=2
500bp
Kruskal-Wallis, p = 0.95
Mean=0.4 SD =0.6
Mean=0.7 SD =1.1
Mean=0.0 SD =0.0
Mean=0.4 SD =0.6
Mean=0.0 SD =0.0
Mean=0.4 SD =0.6
Mean=0.4 SD =0.6
Mean=0.4 SD =0.5
Mean=0.8 SD =1.0
Mean=0.5 SD =0.7
Mean=1.6 SD =2.3
n=6 n=2 n=2 n=2 n=2 n=2 n=2 n=2 n=4 n=2 n=2
% SSA Events
0
5
10
15
0 6.25 10 12.5 25 50 100 200 250 500 1000
RU486 Concentration (µM)
D
Control
0 µM
10 µM
250 µM
RU486 (mifepristone)

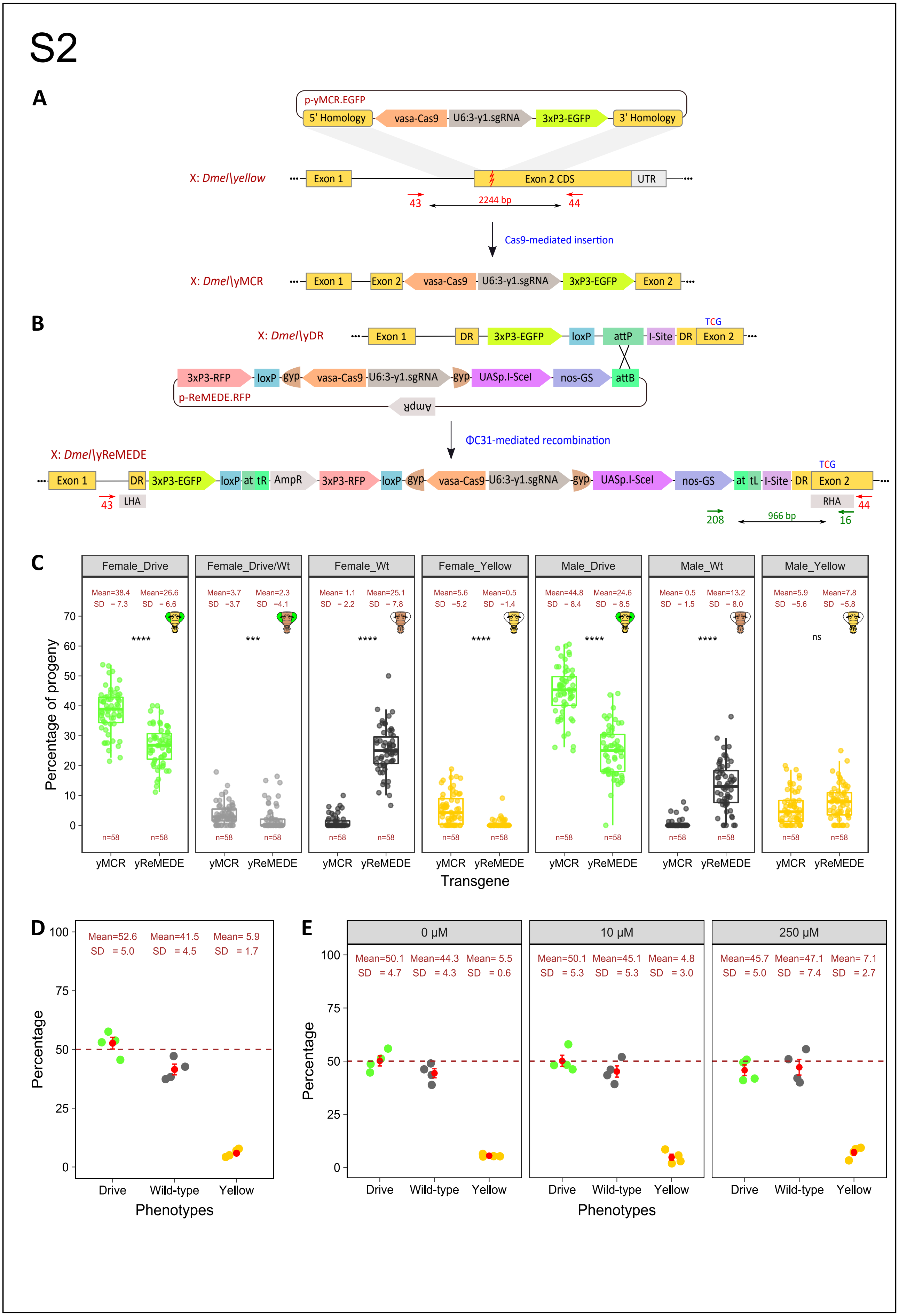

S2
A
p-yMCR.EGFP
5' Homology
vasa-Cas9
U6:3-y1.sgRNA
3xP3-EGFP
3' Homology
X: Dmel\yellow
Exon 1
Exon 2 CDS
UTR
43
2244 bp
44
Cas9-mediated insertion
X: Dmel\yMCR
Exon 1
Exon 2
vasa-Cas9
U6:3-y1.sgRNA
3xP3-EGFP
Exon 2
B
X: Dmel\yDR
Exon 1
DR
3xP3-EGFP
loxP
attP
I-Site
DR
TCG
Exon 2
3xP3-RFP
loxP
gyp
vasa-Cas9
U6:3-y1.sgRNA
gyp
UASp.I-SceI
nos-GS
attB
p-ReMEDE.RFP
AmpR
ΦC31-mediated recombination
X: Dmel\yReMEDE
Exon 1
DR
3xP3-EGFP
loxP
at tR
AmpR
3xP3-RFP
loxP
gyp
vasa-Cas9
U6:3-y1.sgRNA
gyp
UASp.I-SceI
nos-GS
at tL
I-Site
DR
TCG
Exon 2
43
LHA
RHA
44
208
966 bp
16
C
Female_Drive
Female_Drive/Wt
Female_Wt
Female_Yellow
Male_Drive
Male_Wt
Male_Yellow
Mean=38.4 SD = 7.3
Mean=26.6 SD = 6.6
Mean=3.7 SD =3.7
Mean=2.3 SD =4.1
Mean= 1.1 SD = 2.2
Mean=25.1 SD = 7.8
Mean=5.6 SD =5.2
Mean=0.5 SD =1.4
Mean=44.8 SD = 8.4
Mean=24.6 SD = 8.5
Mean= 0.5 SD = 1.5
Mean=13.2 SD = 8.0
Mean=5.9 SD =5.6
Mean=7.8 SD =5.8
****
***
****
****
****
****
ns
Percentage of progeny
n=58
yMCR
yReMEDE
Transgene
D
Mean=52.6 SD = 5.0
Mean=41.5 SD = 4.5
Mean= 5.9 SD = 1.7
Percentage
Drive
Wild-type
Yellow
Phenotypes
E
0 μM
10 μM
250 μM
Mean=50.1 SD = 4.7
Mean=44.3 SD = 4.3
Mean= 5.5 SD = 0.6
Mean=50.1 SD = 5.3
Mean=45.1 SD = 5.3
Mean= 4.8 SD = 3.0
Mean=45.7 SD = 5.0
Mean=47.1 SD = 7.4
Mean= 7.1 SD = 2.7
Percentage
Drive
Wild-type
Yellow
Phenotypes

S3

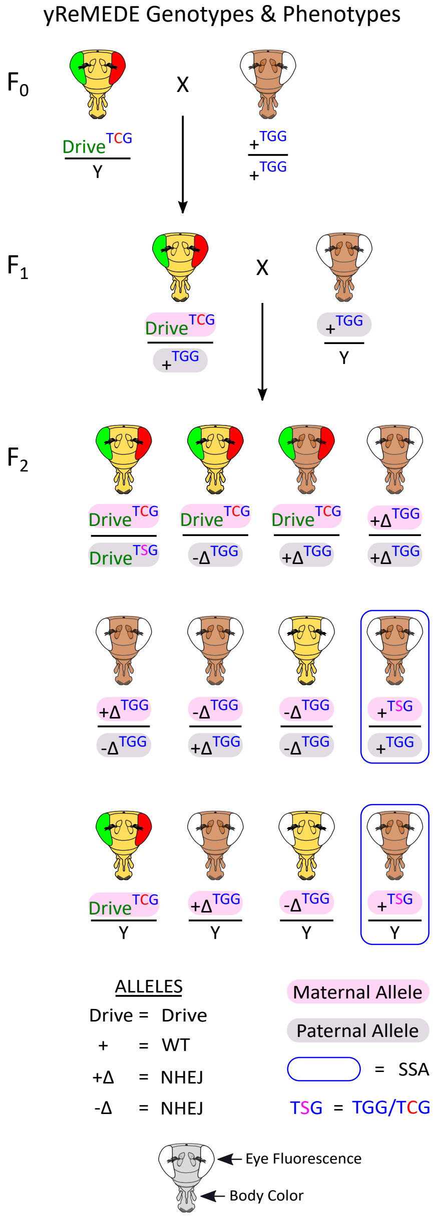
yReMEDE Genotypes & Phenotypes
F0
X
Drive TCG / Y
+TGG / +TGG
F1
X
Drive TCG / +TGG
+TGG / Y
F2
Drive TCG / Drive TSG
Drive TCG / -Δ TGG
Drive TCG / +Δ TGG
+Δ TGG / +Δ TGG
+Δ TGG / -Δ TGG
-Δ TGG / +Δ TGG
-Δ TGG / -Δ TGG
+TSG / +TGG
Drive TCG / Y
+Δ TGG / Y
-Δ TGG / Y
+TSG / Y
ALLELES
Drive = Drive
+ = WT
+Δ = NHEJ
-Δ = NHEJ
Maternal Allele
Paternal Allele
= SSA
TSG = TGG/TCG
Eye Fluorescence
Body Color

S4

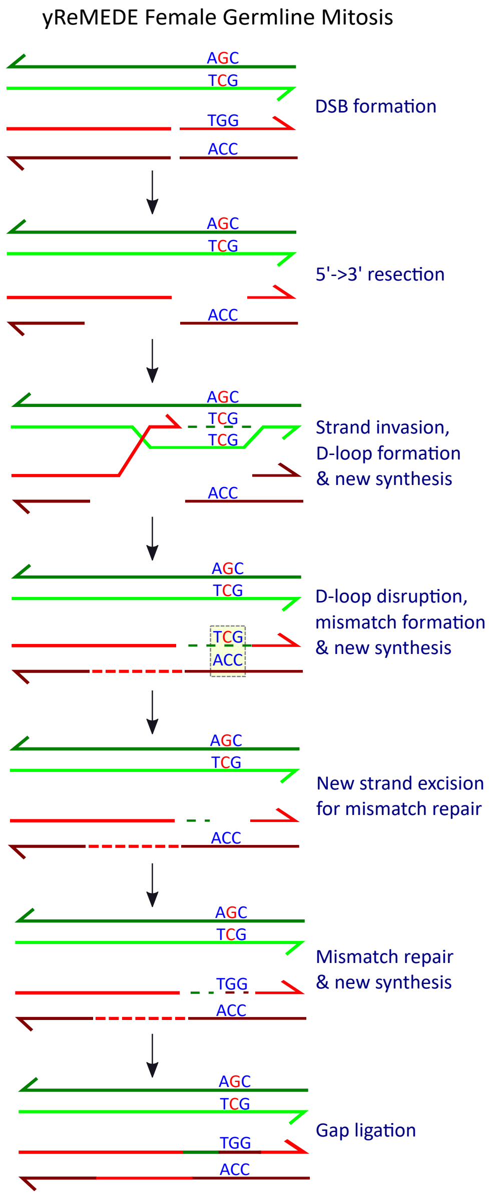
yReMEDE Female Germline Mitosis
AGC
TCG
DSB formation
TGG
ACC
AGC
TCG
5'->3' resection
ACC
AGC
TCG
TCG
Strand invasion,
D-loop formation
& new synthesis
ACC
AGC
TCG
D-loop disruption,
mismatch formation
& new synthesis
TCG
ACC
AGC
TCG
New strand excision
for mismatch repair
ACC
AGC
TCG
Mismatch repair
& new synthesis
TGG
ACC
AGC
TCG
Gap ligation
TGG
ACC

S5

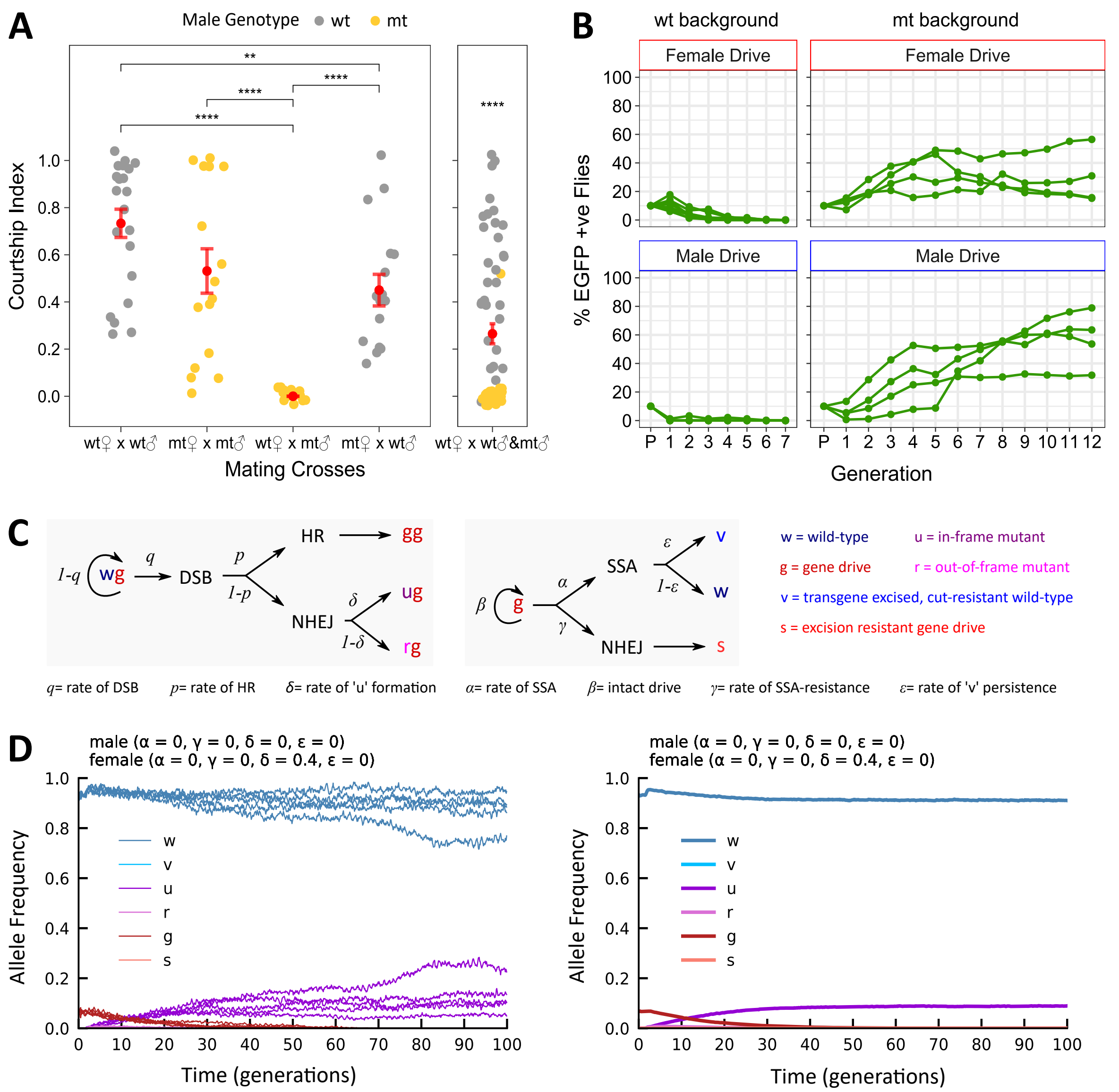
A
Male Genotype wt mt
Courtship Index
Mating Crosses
wt♀ x wt♂
mt♀ x mt♂
wt♀ x mt♂
mt♀ x wt♂
wt♀ x wt♂&mt♂
B
wt background
mt background
Female Drive
Male Drive
% EGFP +ve Flies
Generation
C
HR
gg
DSB
NHEJ
ug
rg
SSA
v
w
s
w = wild-type
u = in-frame mutant
g = gene drive
r = out-of-frame mutant
v = transgene excised, cut-resistant wild-type
s = excision resistant gene drive
q= rate of DSB
p= rate of HR
δ= rate of 'u' formation
α= rate of SSA
β= intact drive
γ= rate of SSA-resistance
ε= rate of 'v' persistence
D
male (α = 0, γ = 0, δ = 0, ε = 0)
female (α = 0, γ = 0, δ = 0.4, ε = 0)
Allele Frequency
Time (generations)

S6. Plasmid Maps and Sequences

p-yDR(30bp).EGFP

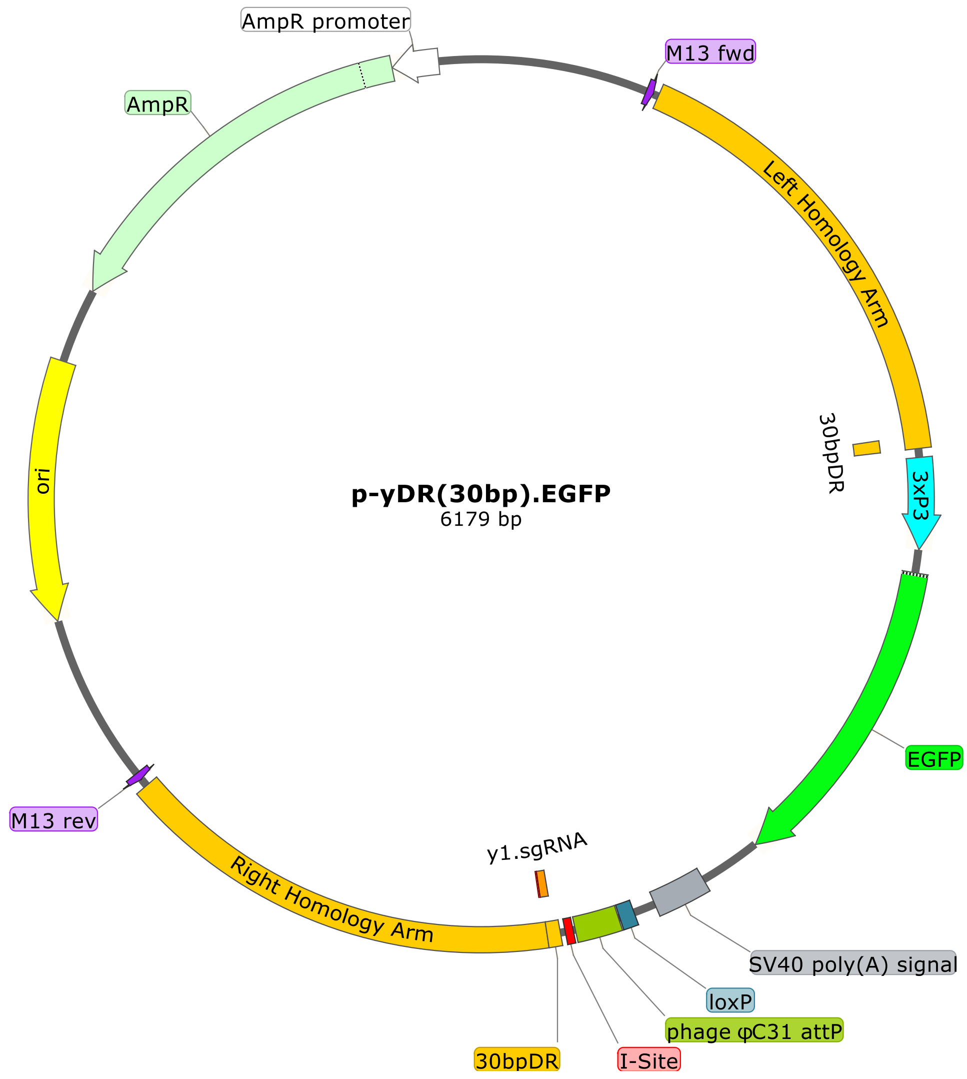

30 bp DR = GTGGTCGGCTGTGGGTTTTGGACACTGGAA

TGG = PAM

tcgcgcgtttcggtgatgacggtgaaaacctctgacacatgcagctcccggagacggtcacagcttgtctgtaagcggatgccgggagcagacaagc
ccgtcagggcgcgtcagcgggtgttggcgggtgtcggggctggcttaactatgcggcatcagagcagattgtactgagagtgcaccatatgcggtgtg
aaataccgcacagatgcgtaaggagaaaataccgcatcaggcgccattcgccattcaggctgcgcaactgttgggaagggcgatcggtgcgggcctc
ttcgctattacgccagctggcgaaagggggatgtgctgcaaggcgattaagttgggtaacgccagggttttcccagtcacgacgttgtaaaacgacgg

ccagtCTCGAGgacgtcGTTGCGAGGTTTTAGGACTGAAAGAGCACATGTCAAAATATAAATTTGTTCAAATACTTTAT
ATTTGACTGAATTAGATTGTTATTTTAAAAGTTATGAATTAAATAAAGATTGAAAGGTGCATTATGCTCAAATGTAT
ATTTATCGCAACCCCCGGTTACTTTGTAAAGCAAAAACGCCTGGTTTGATTTTTAAGAAGATGGGTCGGTAAATCG
ATAAAAGCTATATTTTCTGGTCGTTGCAGTCTCACTCGCCTGCTATAAAAACATTAAAAGTTCCCAGAAACAATAAA
TGTCTTTAAATTCAATTAACGAAGAAATAAAGAAGGAAAAGAACTGGAGCGGAAATCGGTCGAAATACTGCCAAT
GGCCACATATACATTTAACAGCGATATATGGTATACATATTGATAATGATGTCAGACGCAATTGCTTCAGACGGCT
AATGACATCGCAAATTGCACGCAACTTGCAATAGTGCCAATTATGACTGAAGTACATATAGCCGGGGATCTTTTAA
CATAAACTTCCAGTAGATGTACAAGCAGAAAAAAGAGCCATTAGCACGGCAGTTACCATTGCTTATGATTCCTTGT
GTCCAAAATAATGACAAATAGGTATATAAATAATTAAATGCCAAACATAAGCGATTCTAATTTACCTTTACATCTGT
ATGCATTTACATATTATCCAGAAAACAGACAGCGATAACTTGCAACATTGCTTAGTATAATAATCCAAAGAAGGAA
TTTAGGCAGAAATTCCAGTTAATTAAATATTCAAAACAAACTTTATTTAGTGCCTCAATAATAGTTTGGCCCTGCTA
ATTCTCCTATTTTATTTTTTAGGGATTCCGGCCACTCTGACCTATATAAACATGGACCGCAGTTTGACGGGTTCACC
GGAGCTAATTCCGTATCCAGATTGGCGCTCAAATACAGCTGGAGATTGCGCCAACAGTATTACCACTGCCTACCGC
ATTAAAGTGGATGAGT**GTGGTCGGCTGTGGGTTTTGGACACTGGAA**GCGGCCGCCCTAGGCGTACGGGATCTAA
TTCAATTAGAGACTAATTCAATTAGAGCTAATTCAATTAGGATCCAAGCTTATCGATTTCGAACCCTCGACCGCCGG
AGTATAAATAGAGGCGCTTCGTCTACGGAGCGACAATTCAATTCAAACAAGCAAAGTGAACACGTCGCTAAGCGA
AAGCTAAGCAAATAAACAAGCGCAGCTGAACAAGCTAAACAATCGGGGTACCGCTAGAGTCGACGGTACCGCGG
GCCCGGGATCCACCGGTCGCCACCATGGTGAGCAAGGGCGAGGAGCTGTTCACCGGGGTGGTGCCCATCCTGGT
CGAGCTGGACGGCGACGTAAACGGCCACAAGTTCAGCGTGTCCGGCGAGGGCGAGGGCGATGCCACCTACGGC
AAGCTGACCCTGAAGTTCATCTGCACCACCGGCAAGCTGCCCGTGCCCTGGCCCACCCTCGTGACCACCCTGACCT
ACGGCGTGCAGTGCTTCAGCCGCTACCCCGACCACATGAAGCAGCACGACTTCTTCAAGTCCGCCATGCCCGAAG
GCTACGTCCAGGAGCGCACCATCTTCTTCAAGGACGACGGCAACTACAAGACCCGCGCCGAGGTGAAGTTCGAGG
GCGACACCCTGGTGAACCGCATCGAGCTGAAGGGCATCGACTTCAAGGAGGACGGCAACATCCTGGGGCACAAG
CTGGAGTACAACTACAACAGCCACAACGTCTATATCATGGCCGACAAGCAGAAGAACGGCATCAAGGTGAACTTC
AAGATCCGCCACAACATCGAGGACGGCAGCGTGCAGCTCGCCGACCACTACCAGCAGAACACCCCCATCGGCGAC
GGCCCCGTGCTGCTGCCCGACAACCACTACCTGAGCACCCAGTCCGCCCTGAGCAAAGACCCCAACGAGAAGCGC
GATCACATGGTCCTGCTGGAGTTCGTGACCGCCGCCGGGATCACTCTCGGCATGGACGAGCTGTACAAGTAAAGC
GGCCTAAGCGATCGCTCAGGCCGCGACTCTAGATCATAATCAGCCATACCACATTTGTAGAGGTTTTACTTGCTTTA
AAAAACCTCCCACACCTCCCCCTGAACCTGAAACATAAAATGAATGCAATTGTTGTTGTTAACTTGTTTATTGCAGC
TTATAATGGTTACAAATAAAGCAATAGCATCACAAATTTCACAAATAAAGCATTTTTTTCACTGCATTCTAGTTGTG
GTTTGTCCAAACTCATCAATGTATCTTAAAGCTTATCGATACGCGTACGGCGCGCCAAAGCTTTGTCTCGAGTCCGG
AATAACTTCGTATAGCATACATTATACGAAGTTATGGTCCCAGGTCAGAAGCGGTTTTCGGGAGTAGTGCCCCAAC
TGGGGTAACCTTTGAGTTCTCTCAGTTGGGGGCGTAGGGTCGCCGACATGACACAAGGGGTTTGGTCTAGGGATA
ACAGGGTAATAGCGGCCGGCC**GTGGTCGGCTGTGGGTTTTGGACACTGGAA**CCGTGGGCATCGGCAATACCACC
ACTAATCCGTGCCCCTATGCGGTAAATGTCTTTGACTTGACCACGGATACGCGAATTCGGAGATACGAGCTACCTG
GCGTGGACACAAATCCAAATACTTTCATAGCTAACATTGCCGTGGATATAGGCAAAAATTGCGATGATGCATATGC
CTATTTTGCCGATGAATTGGGATACGGCTTGATTGCTTACTCCTGGGAACTGAACAAGTCCTGGAGATTCTCGGCA
CATTCGTATTTTTTCCCCGATCCATTGAGGGGCGATTTCAATGTCGCTGGTATTAACTTCCAATGGGGCGAGGAGG
GTATATTTGGTATGTCCCTTTCGCCCATTCGATCGGATGGTTATCGTACCCTGTACTTTAGTCCGTTAGCAAGTCATC
GACAATTTGCCGTATCCACGAGGATTTTGAGGGATGAAACCAGGACGGAAGATAGCTATCATGACTTTGTTGCCTT
AGATGAACGGGGTCCAAACTCCCATACCACTTCACGTGTGATGAGCGATGATGGAATTGAGCTGTTCAATTTAATA
GATCAAAATGCAGTGGGTTGCTGGCACTCATCAATGCCGTACTCACCGCAATTTCATGGCATTGTGGATCGCGATG
ACGTTGGCTTAGTTTTTCCGGCCGATGTGAAAATTGATGAGAACAAAAACGTTTGGGTTCTATCCGATAGGATGCC
CGTTTTCTTGCTGTCTGACTTGGATTATTCAGATACTAATTTCCGAATTTACACGGCTCCCTTGGCCACTTTAATTGA

GAATACTGTGTGTGATTTGAGGAATAACGCCTATGGGCCGCCAAATACCGTTTCAATACCAAAACAAGCCGTTTTG
CCAATGGGTCCACCGTTATATACGAAACAATATCGTCCTGTCTTGCCACAGAAACCTCAGACCAGCTGGGCTTCCTC
GCCGCCTCCTCCAAGTCGCACTTATTTGCCCGCCAATTCAGGCAATGTAGTCTCCAGgctagcggcgtaatcatggtcatagc
tgtttcctgtgtgaaattgttatccgctcacaattccacacaacatacgagccggaagcataaagtgtaaagcctggggtgcctaatgagtgagctaac
tcacattaattgcgttgcgctcactgcccgctttccagtcgggaaacctgtcgtgccagctgcattaatgaatcggccaacgcgcggggagaggcggtt
tgcgtattgggcgctcttccgcttcctcgctcactgactcgctgcgctcggtcgttcggctgcggcgagcggtatcagctcactcaaaggcggtaatacg
gttatccacagaatcaggggataacgcaggaaagaacatgtgagcaaaaggccagcaaaaggccaggaaccgtaaaaaggccgcgttgctggcgt
ttttccataggctccgcccccctgacgagcatcacaaaaatcgacgctcaagtcagaggtggcgaaacccgacaggactataaagataccaggcgttt
ccccctggaagctccctcgtgcgctctcctgttccgaccctgccgcttaccggatacctgtccgcctttctcccttcgggaagcgtggcgctttctcatagc
tcacgctgtaggtatctcagttcggtgtaggtcgttcgctccaagctgggctgtgtgcacgaaccccccgttcagcccgaccgctgcgccttatccggta
actatcgtcttgagtccaacccggtaagacacgacttatcgccactggcagcagccactggtaacaggattagcagagcgaggtatgtaggcggtgct
acagagttcttgaagtggtggcctaactacggctacactagaagaacagtatttggtatctgcgctctgctgaagccagttaccttcggaaaaagagtt
ggtagctcttgatccggcaaacaaaccaccgctggtagcggtggtttttttgtttgcaagcagcagattacgcgcagaaaaaaaggatctcaagaaga
tcctttgatcttttctacggggtctgacgctcagtggaacgaaaactcacgttaagggattttggtcatgagattatcaaaaaggatcttcacctagatc
cttttaaattaaaaatgaagttttaaatcaatctaaagtatatatgagtaaacttggtctgacagttaccaatgcttaatcagtgaggcacctatctcag
cgatctgtctatttcgttcatccatagttgcctgactccccgtcgtgtagataactacgatacgggagggcttaccatctggccccagtgctgcaatgata
ccgcgagacccacgctcaccggctccagatttatcagcaataaaccagccagccggaagggccgagcgcagaagtggtcctgcaactttatccgcct
ccatccagtctattaattgttgccgggaagctagagtaagtagttcgccagttaatagtttgcgcaacgttgttgccattgctacaggcatcgtggtgtca
cgctcgtcgtttggtatggcttcattcagctccggttcccaacgatcaaggcgagttacatgatcccccatgttgtgcaaaaaagcggttagctccttcg
gtcctccgatcgttgtcagaagtaagttggccgcagtgttatcactcatggttatggcagcactgcataattctcttactgtcatgccatccgtaagatgc
ttttctgtgactggtgagtactcaaccaagtcattctgagaatagtgtatgcggcgaccgagttgctcttgcccggcgtcaatacgggataataccgcgc
cacatagcagaactttaaaagtgctcatcattggaaaacgttcttcggggcgaaaactctcaaggatcttaccgctgttgagatccagttcgatgtaac
ccactcgtgcacccaactgatcttcagcatcttttactttcaccagcgtttctgggtgagcaaaaacaggaaggcaaaatgccgcaaaaaagggaata
agggcgacacggaaatgttgaatactcatactcttcctttttcaatattattgaagcatttatcagggttattgtctcatgagcggatacatatttgaatgt
atttagaaaaataaacaaataggggttccgcgcacatttccccgaaaagtgccacctgacgtctaagaaaccattattatcatgacattaacctataa
aaataggcgtatcacgaggccctttcgtc

p-yDR(250bp).EGFP

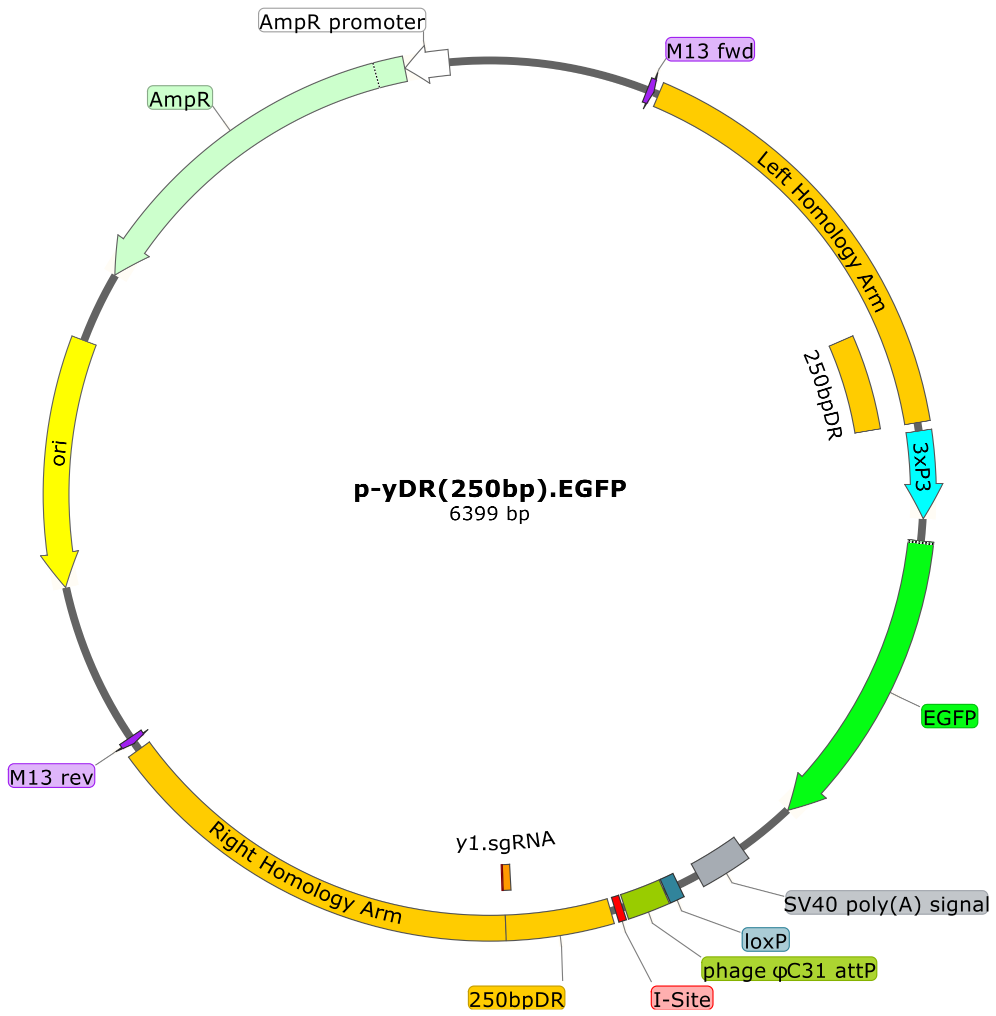

250 bp DR =
ATATTCAAAACAAACTTTATTTAGTGCCTCAATAATAGTTTGGCCCTGCTAATTCTCCTATTTTATTTTTTAGGGATTCCGGCCACTCTGACCTATATAAACATGGACCGCAGTTTGACGGGTTCACCGGAGCTAATTCCGTATCCAGATTGGCGCTCAAATACAGCTGGAGATTGCGCCAACAGTATTACCACTGCCTACCGCATTAAAGTGGATGAGTGTGGTCGGCTGTGGGTTTTGGACACTGGAA

p-yDR(500bp).EGFP

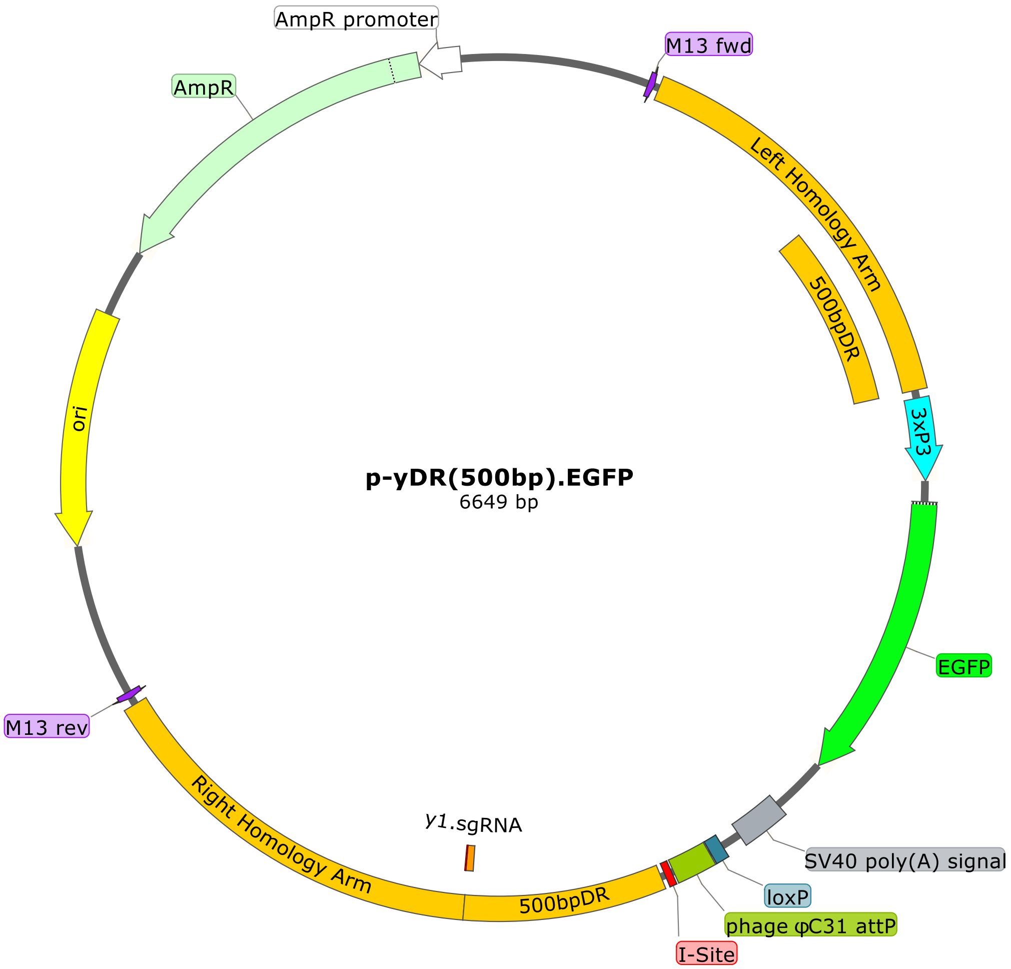

500 bp DR =
ACTTCCAGTAGATGTACAAGCAGAAAAAAGAGCCATTAGCACGGCAGTTACCATTGCTTATGATTCCTTGTGTCC
AAAATAATGACAAATAGGTATATAAATAATTAAATGCCAAACATAAGCGATTCTAATTTACCTTTACATCTGTAT
GCATTTACATATTATCCAGAAAACAGACAGCGATAACTTGCAACATTGCTTAGTATAATAATCCAAAGAAGGAA
TTTAGGCAGAAATTCCAGTTAATTAAATATTCAAAACAAACTTTATTTAGTGCCTCAATAATAGTTTGGCCCTGCT
AATTCTCCTATTTTATTTTTTAGGGATTCCGGCCACTCTGACCTATATAAACATGGACCGCAGTTTGACGGGTTCA
CCGGAGCTAATTCCGTATCCAGATTGGCGCTCAAATACAGCTGGAGATTGCGCCAACAGTATTACCACTGCCTAC
CGCATTAAAGTGGATGAGTGTGGTCGGCTGTGGGTTTTGGACACTGGAA

p-ReMET.RFP_*in trans*

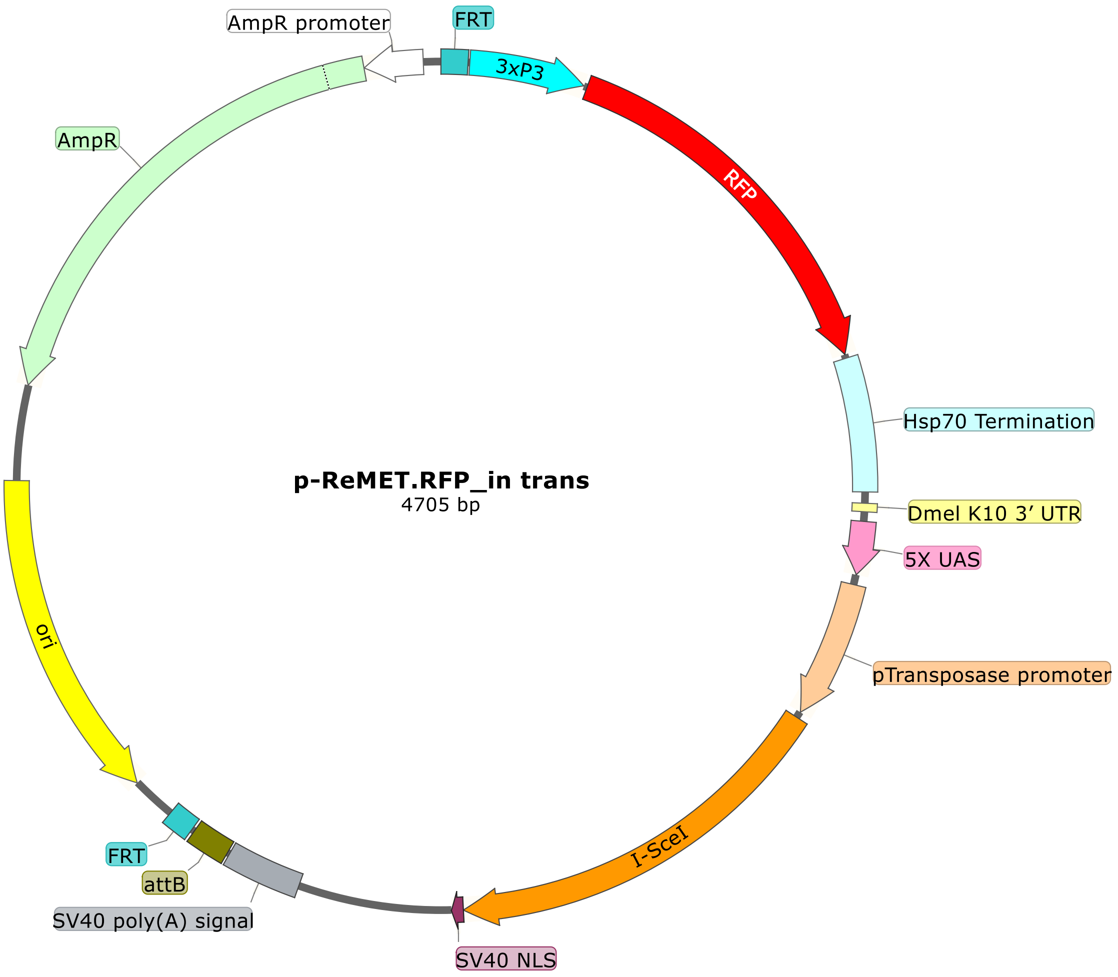

GAAGTTCCTATTCCGAAGTTCCTATTCTCTAGAAAGTATAGGAACTTCgtaGGATCTAATTCAATTAGAGACTAATTC
AATTAGAGCTAATTCAATTAGGATCCAAGCTTATCGATTTCGAACCCTCGACCGCCGGAGTATAAATAGAGGCGCT
TCGTCTACGGAGCGACAATTCAATTCAAACAAGCAAAGTGAACACGTCGCTAAGCGAAAGCTAAGCAAATAAACA
AGCGCAGCTGAACAAGCTAAACAATCGGGCCACCATGAGGTCTTCCAAGAATGTTATCAAGGAGTTCATGAGGTT
TAAGGTTCGCATGGAAGGAACGGTCAATGGGCACGAGTTTGAAATAGAAGGCGAAGGAGAGGGGAGGCCATAC
GAAGGCCACAATACCGTAAAGCTTAAGGTAACCAAGGGGGGACCTTTGCCATTTGCTTGGGATATTTTGTCACCAC
AATTTCAGTATGGAAGCAAGGTATATGTCAAGCACCCTGCCGACATACCAGACTATAAAAAGCTGTCATTTCCTGA
AGGATTTAAATGGGAAAGGGTCATGAACTTTGAAGACGGTGGCGTCGTTACTGTAACCCAGGATTCCAGTTTGCA
GGATGGCTGTTTCATCTACAAGGTCAAGTTCATTGGCGTGAACTTTCCTTCCGATGGACCTGTTATGCAAAAGAAG
ACAATGGGCTGGGAAGCCAGCACTGAGCGTTTGTATCCTCGTGATGGCGTGTTGAAAGGAGAGATTCATAAGGCT
CTGAAGCTGAAAGACGGTGGTCATTACCTAGTTGAATTCAAAAGTATTTACATGGCAAAGAAGCCTGTGCAGCTAC
CAGGGTACTACTATGTTGACTCCAAACTGGATATAACAAGCCACAACGAAGACTATACAATCGTTGAGCAGTATGA
AAGAACCGAGGGACGCCACCATCTGTTCCTTTAGCGGCCAACGTCGACTAAAGCCAAATAGAAATTATTCAGTTCT

GGCTTAAGTTTTTAAAAGTGATATTATTTATTTGGTTGTAACCAACCAAAAGAATGTAAATAACTAATACATAATTA
TGTTAGTTTTAAGTTAGCAACAAATTGATTTTAGCTATATTAGCTACTTGGTTAATAAATAGAATATATTTATTTAAA
GATAATTCGTTTTTATTGTCAGGGAGTGAGTTTGCTTAAAAACTCGTTTAAGCTTATCGATACGCGTACGGCGCGCC
AAAGCTTTGTTCgCCTAGGatctgCGGAGTACTGTCCTCCGAGCGGAGTACTGTCCTCCGAGCGGAGTACTGTCCTC
CGAGCGGAGTACTGTCCTCCGAGCGGAGTACTGTCCTCCGAGCGGAGACTCTAGCGAGCGAGCCGTAGCTTACCG
AAGTATACACTTAAATTCAGTGCACGTTTGCTTGTTGAGAGGAAAGGTTGTGTGCGGACGAATTTTTTTTTGAAAA
CCGGTGATAGAGCCTGAACCAGAAAAGATAAAAGAAGGCTATACCAGTGGGAGTACACAAACAGAGTAAGTTTG
AATAGTAAAAAAAATCATTTATGTAAACAATAACGTGACTGTGCGTTAGGTCCTGTTCATTGGTACCCGCCCGGGG
ATCATCTGTTCTAGAATGAAAAACATCAAAAAAAACCAGGTAATGAACCTGGGTCCGAACTCTAAACTGCTGAAAG
AATACAAATCCCAGCTGATCGAACTGAACATCGAACAGTTCGAAGCAGGTATCGGTCTGATCCTGGGTGATGCTTA
CATCCGTTCTCGTGATGAAGGTAAAACCTACTGTATGCAGTTCGAGTGGAAAAACAAAGCATACATGGACCACGT
ATGTCTGCTGTACGATCAGTGGGTACTGTCCCCGCCGCACAAAAAAGAACGTGTTAACCACCTGGGTAACCTGGTA
ATCACCTGGGGCGCCCAGACTTTCAAACACCAAGCTTTCAACAAACTGGCTAACCTGTTCATCGTTAACAACAAAA
AAACCATCCCGAACAACCTGGTTGAAAACTACCTGACCCCGATGTCTCTGGCATACTGGTTCATGGATGATGGTGG
TAAATGGGATTACAACAAAAACTCTACCAACAAATCGATCGTACTGAACACCCAGTCTTTCACTTTCGAAGAAGTA
GAATACCTGGTTAAGGGTCTGCGTAACAAATTCCAACTGAACTGTTACGTAAAAATCAACAAAAACAAACCGATCA
TCTACATCGATTCTATGTCTTACCTGATCTTCTACAACCTGATCAAACCGTACCTGATCCCGCAGATGATGTACAAAC
TGCCGAACACTATCTCCTCCGAAACTTTCCTGAAACCAAAAAAGAAGAGAAAGGTATAAGAAGACCCCAAGGACT
TTCCTTCAGAATTGCTAAGTTTTTTGAGTCATGCTGTGTTTAGTAATAGAACTCTTGCTTGCTTTGCTATTTACACCA
CAAAGGAAAAAGCTGCACTGCTATACAAGAAAATTATGGAAAAATATTTGATGTATAGTGCCTTGACTAGAGATC
ATAATCAGCCATACCACATTTGTAGAGGTTTTACTTGCTTTAAAAAACCTCCCACACCTCCCCCTGAACCTGAAACAT
AAAATGAATGCAATTGTTGTTGTTAACTTGTTTATTGCAGCTTATAATGGTTACAAATAAAGCAATAGCATCACAAA
TTTCACAAATAAAGCATTTTTTTCACTGCATTCTAGTTGTGGTTTGTCCAAACTCATCAATGTATCTTATCATGTCTG
GATCCGGTCTCGAAGCCGCGGTGCGGGTGCCAGGGCGTGCCCTTGGGCTCCCCGGGCGCGTACTCCACCTCACCC
ATCTGGTCGAAGTTCCTATTCCGAAGTTCCTATTCTCTAGAAAGTATAGGAACTTCCCAATGATATCGGAAAGAAC
ATGTGAGCAAAAGGCCAGCAAAAGGCCAGGAACCGTAAAAAGGCCGCGTTGCTGGCGTTTTTCCATAGGCTCCGC
CCCCCTGACGAGCATCACAAAAATCGACGCTCAAGTCAGAGGTGGCGAAACCCGACAGGACTATAAAGATACCAG
GCGTTTCCCCCTGGAAGCTCCCTCGTGCGCTCTCCTGTTCCGACCCTGCCGCTTACCGGATACCTGTCCGCCTTTCTC
CCTTCGGGAAGCGTGGCGCTTTCTCATAGCTCACGCTGTAGGTATCTCAGTTCGGTGTAGGTCGTTCGCTCCAAGC
TGGGCTGTGTGCACGAACCCCCCGTTCAGCCCGACCGCTGCGCCTTATCCGGTAACTATCGTCTTGAGTCCAACCC
GGTAAGACACGACTTATCGCCACTGGCAGCAGCCACTGGTAACAGGATTAGCAGAGCGAGGTATGTAGGCGGTG
CTACAGAGTTCTTGAAGTGGTGGCCTAACTACGGCTACACTAGAAGAACAGTATTTGGTATCTGCGCTCTGCTGAA
GCCAGTTACCTTCGGAAAAAGAGTTGGTAGCTCTTGATCCGGCAAACAAACCACCGCTGGTAGCGGTGGTTTTTTT
GTTTGCAAGCAGCAGATTACGCGCAGAAAAAAAGGATCTCAAGAAGATCCTTTGATCTTTTCTACGGGGTCTGAC
GCTCAGTGGAACGAAAACTCACGTTAAGGGATTTTGGTCATGAGATTATCAAAAAGGATCTTCACCTAGATCCTTT
TAAATTAAAAATGAAGTTTTAAATCAATCTAAAGTATATATGAGTAAACTTGGTCTGACAGTTACCAATGCTTAATC
AGTGAGGCACCTATCTCAGCGATCTGTCTATTTCGTTCATCCATAGTTGCCTGACTCCCCGTCGTGTAGATAACTAC
GATACGGGAGGGCTTACCATCTGGCCCCAGTGCTGCAATGATACCGCGAGACCCACGCTCACCGGCTCCAGATTT
ATCAGCAATAAACCAGCCAGCCGGAAGGGCCGAGCGCAGAAGTGGTCCTGCAACTTTATCCGCCTCCATCCAGTC
TATTAATTGTTGCCGGGAAGCTAGAGTAAGTAGTTCGCCAGTTAATAGTTTGCGCAACGTTGTTGCCATTGCTACA
GGCATCGTGGTGTCACGCTCGTCGTTTGGTATGGCTTCATTCAGCTCCGGTTCCCAACGATCAAGGCGAGTTACAT
GATCCCCCATGTTGTGCAAAAAAGCGGTTAGCTCCTTCGGTCCTCCGATCGTTGTCAGAAGTAAGTTGGCCGCAGT
GTTATCACTCATGGTTATGGCAGCACTGCATAATTCTCTTACTGTCATGCCATCCGTAAGATGCTTTTCTGTGACTG
GTGAGTACTCAACCAAGTCATTCTGAGAATAGTGTATGCGGCGACCGAGTTGCTCTTGCCCGGCGTCAATACGGG

ATAATACCGCGCCACATAGCAGAACTTTAAAAGTGCTCATCATTGGAAAACGTTCTTCGGGGCGAAAACTCTCAAG
GATCTTACCGCTGTTGAGATCCAGTTCGATGTAACCCACTCGTGCACCCAACTGATCTTCAGCATCTTTTACTTTCAC
CAGCGTTTCTGGGTGAGCAAAAACAGGAAGGCAAAATGCCGCAAAAAAGGGAATAAGGGCGACACGGAAATGT
TGAATACTCATACTCTTCCTTTTTCAATATTATTGAAGCATTTATCAGGGTTATTGTCTCATGAGCGGATACATATTT
GAATGTATTTAGAAAAATAAACAAATAGGGGTTCCGCGCACATTTCCCCGAAAAGTGCCACCTGACGTC

p-ReMET.RFP_*in cis*

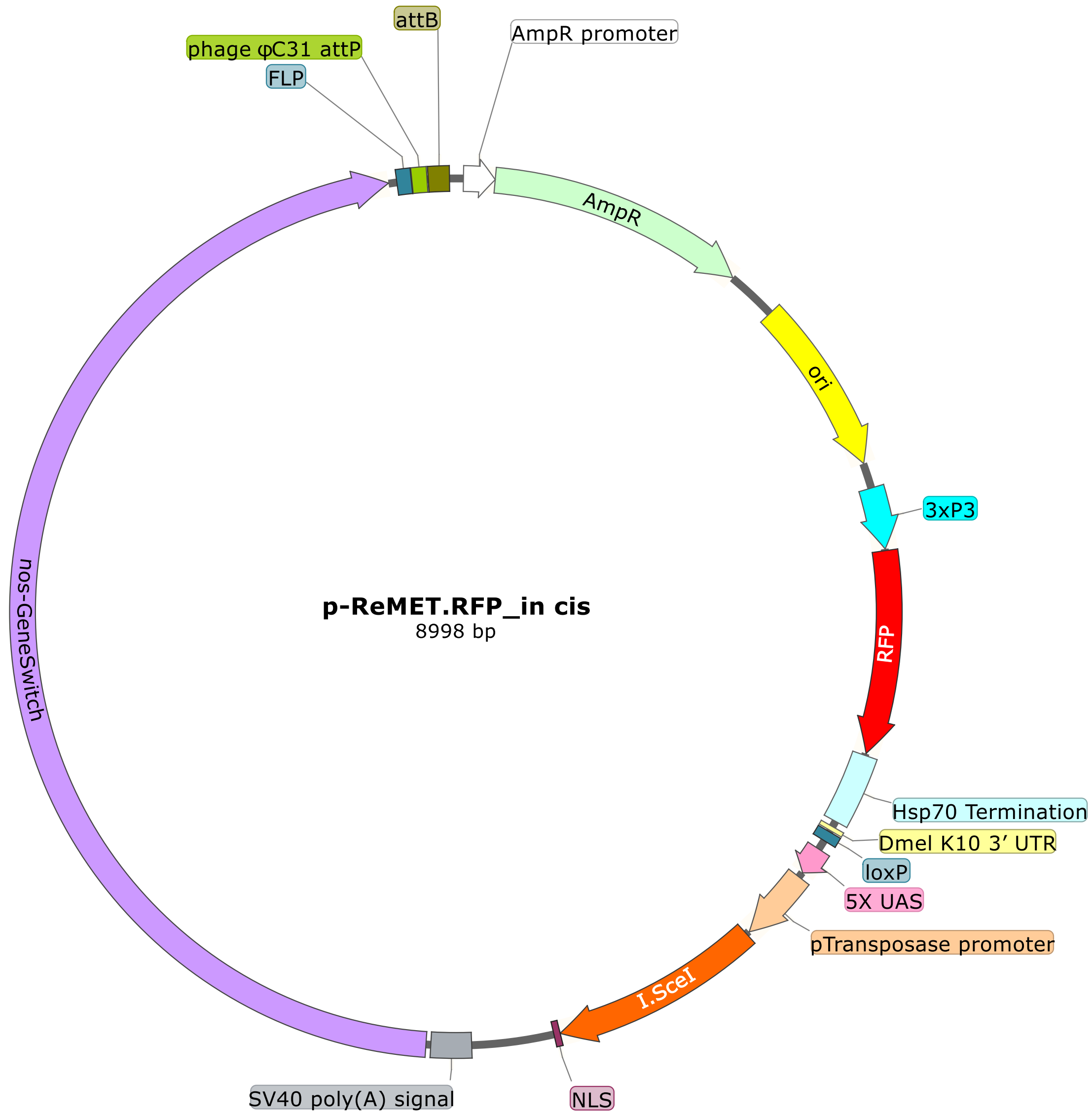

aggtggcacttttcggggaaatgtgcgcggaacccctatttgtttatttttctaaatacattcaaatatgtatccgctcatgagacaataaccctgataaa
tgcttcaataatattgaaaaaggaagagtatgagtattcaacatttccgtgtcgcccttattcccttttttgcggcattttgccttcctgtttttgctcaccc
agaaacgctggtgaaagtaaaagatgctgaagatcagttgggtgcacgagtgggttacatcgaactggatctcaacagcggtaagatccttgagagt
tttcgccccgaagaacgttttccaatgatgagcacttttaaagttctgctatgtggcgcggtattatcccgtattgacgccgggcaagagcaactcggtc
gccgcatacactattctcagaatgacttggttgagtactcaccagtcacagaaaagcatcttacggatggcatgacagtaagagaattatgcagtgct
gccataaccatgagtgataacactgcggccaacttacttctgacaacgatcggaggaccgaaggagctaaccgcttttttgcacaacatgggggatca
tgtaactcgccttgatcgttgggaaccggagctgaatgaagccataccaaacgacgagcgtgacaccacgatgcctgtagcaatggcaacaacgttg
cgcaaactattaactggcgaactacttactctagcttcccggcaacaattaatagactggatggaggcggataaagttgcaggaccacttctgcgctcg
gcccttccggctggctggtttattgctgataaatctggagccggtgagcgtgggtctcgcggtatcattgcagcactggggccagatggtaagccctccc

gtatcgtagttatctacacgacggggagtcaggcaactatggatgaacgaaatagacagatcgctgagataggtgcctcactgattaagcattggtaa
ctgtcagaccaagtttactcatatatactttagattgatttaaaacttcatttttaatttaaaaggatctaggtgaagatcctttttgataatctcatgacc
aaaatcccttaacgtgagttttcgttccactgagcgtcagaccccgtagaaaagatcaaaggatcttcttgagatcctttttttctgcgcgtaatctgctg
cttgcaaacaaaaaaaccaccgctaccagcggtggtttgtttgccggatcaagagctaccaactctttttccgaaggtaactggcttcagcagagcgc
agataccaaatactgttcttctagtgtagccgtagttaggccaccacttcaagaactctgtagcaccgcctacatacctcgctctgctaatcctgttacca
gtggctgctgccagtggcgataagtcgtgtcttaccgggttggactcaagacgatagttaccggataaggcgcagcggtcgggctgaacggggggttc
gtgcacacagcccagcttggagcgaacgacctacaccgaactgagatacctacagcgtgagctatgagaaagcgccacgcttcccgaagggagaaa
ggcggacaggtatccggtaagcggcagggtcggaacaggagagcgcacgagggagcttccagggggaaacgcctggtatctttatagtcctgtcgg
gtttcgccacctctgacttgagcgtcgatttttgtgatgctcgtcaggggggcggagcctatggaaaaacgccagcaacgcggcctttttacggttcctg
gccttttgctggccttttgctcacatgttctttccgatatcattgggacgtcggatctaattcaattagagactaattcaattagagctaattcaattagga
tccaagcttatcgatttcgaaccctcgaccgccggagtataaatagaggcgcttcgtctacggagcgacaattcaattcaaacaagcaaagtgaacac
gtcgctaagcgaaagctaagcaaataaacaagcgcagctgaacaagctaaacaatcgggccaccatgaggtcttccaagaatgttatcaaggagtt
catgaggtttaaggttcgcatggaaggaacggtcaatgggcacgagtttgaaatagaaggcgaaggagaggggaggccatacgaaggccacaata
ccgtaaagcttaaggtaaccaagggggacctttgccatttgcttgggatattttgtcaccacaatttcagtatggaagcaaggtatatgtcaagcacc
ctgccgacataccagactataaaaagctgtcatttcctgaaggatttaaatgggaaagggtcatgaactttgaagacggtggcgtcgttactgtaaccc
aggattccagtttgcaggatggctgtttcatctacaaggtcaagttcattggcgtgaactttccttccgatggacctgttatgcaaaagaagacaatggg
ctgggaagccagcactgagcgtttgtatcctcgtgatggcgtgttgaaaggagagattcataaggctctgaagctgaaagacggtggtcattacctag
ttgaattcaaaagtatttacatggcaaagaagcctgtgcagctaccagggtactactatgttgactccaaactggatataacaagccacaacgaagac
tatacaatcgttgagcagtatgaaagaaccgagggacgccaccatctgttcctttagcggccaacgtcgactaaagccaaatagaaattattcagttct
ggcttaagtttttaaaagtgatattatttatttggttgtaaccaaccaaaagaatgtaaataactaatacataattatgttagttttaagttagcaacaaa
ttgattttagctatattagctacttggttaataaatagaatatatttatttaaagataattcgtttttattgtcagggagtgagtttgcttaaaaactcgttt
aagcttatcgatacgcgtacggcgcgccaaagctttgttcataacttcgtatagcatacattatacgaagttatgtccggaatgcccgggcatgcgcct
aggatctgcggagtactgtcctccgagcggagtactgtcctccgagcggagtactgtcctccgagcggagtactgtcctccgagcggagtactgtcctc
cgagcggagactctagcgagcgagccgtagcttaccgaagtatacacttaaattcagtgcacgtttgcttgttgagaggaaaggttgtgtgcggacga
atttttttttgaaaaccggtgatagagcctgaaccagaaaagataaaagaaggctataccagtgggagtacacaaacagagtaagtttgaatagtaa
aaaaaatcatttatgtaaacaataacgtgactgtgcgttaggtcctgttcattggtacccgcccggggatcatctgttctagaatgaaaaacatcaaaa
aaaaccaggtaatgaacctgggtccgaactctaaactgctgaaagaatacaaatcccagctgatcgaactgaacatcgaacagttcgaagcaggtat
cggtctgatcctgggtgatgcttacatccgttctcgtgatgaaggtaaaacctactgtatgcagttcgagtggaaaaacaaagcatacatggaccacgt
atgtctgctgtacgatcagtgggtactgtccccgccgcacaaaaaagaacgtgttaaccacctgggtaacctggtaatcacctggggcgcccagacttt
caaacaccaagctttcaacaaactggctaacctgttcatcgttaacaacaaaaaaaccatcccgaacaacctggttgaaaactacctgaccccgatgt
ctctggcatactggttcatggatgatggtggtaaatgggattacaacaaaaactctaccaacaaatcgatcgtactgaacacccagtctttcactttcg
aagaagtagaatacctggttaagggtctgcgtaacaaattccaactgaactgttacgtaaaaatcaacaaaaacaaaccgatcatctacatcgattct
atgtcttacctgatcttctacaacctgatcaaaccgtacctgatcccgcagatgatgtacaaactgccgaacactatctcctccgaaactttcctgaaac
caaaaaagaagagaaaggtataagaagaccccaaggactttccttcagaattgctaagtttttttgagtcatgctgtgtttagtaatagaactcttgctt
gctttgctatttacaccacaaaggaaaaagctgcactgctatacaagaaaattatggaaaaatatttgatgtatagtgccttgactagagatcataatc
agccataccacatttgtagaggttttacttgctttaaaaaacctcccacacctccccctgaacctgaaacataaaatgaatgcaattgttgttgttaactt
gtttattgcagcttataatggttacaaataaagcaatagcatcacaaatttcacaaataaagcatttttttcactgcattctagttgtggtttgtccaaact
catcaatgtatcttatcatgtctggatcgcgcttattgattggctggtccatcatgatgaacgggtcgaggtggcggtagttgatcccggcgaacgcgcg
gcgcaccgggaagccctcgccctcgaaaccgctgggcgcggtggtcacggtgagcacgggacgtgcgacggcgtcggctggtgcggatacgcggggg
cagcgtcagcgggttctcgacggtcacggcgggcatgtcgacaagccgaattgatccactagaaggcctaattcgtctagcaggatctctagggcttc
gaccgttttaacctcgaaatatgcacatgtaaggacggatgtgagcgaacgccagtgatgaccgggatcagaggtaacctaccatggtggggattag
gtgaccgttcgcaggtagtttgatcggagcgaatgttcggggggtctggcgtcagaggctctaaactttatgtaattcctgccgcgaaacacgcacgta
tcaagcagtcagctgttctcttcgttcagcgcgcgccggtgttgcaaaacgagcgctcttcgccggcggtggctcgtgcgatagttcgttttgtcggtaat
ccgatgttgccgcgccgatatcatgtgatgttgtcacagtgcgcgaaattcgaatggtggtgtgcagtgattgtgttgtgacggcgagtggcgcgtgtgg

gtgcttagttttgggagatgttttcgtatttttttgttgataactcaggctttgttgctgtgttgtagtactattttccattgcgcggtgtccagcttttaatta
gtggcacatattcttagcaagtaaaaattattttgcatactattaaatttcttataaattattttctaaaattaagtttaccttttcaattttactaaaaatat
cgatatatttattatcgctggaaaactacattattccacctctaagcaagaaccgttagttggcgcgtagctttaccacaaaattcctggaattgccgta
cgcttcgcagttgtttcaagttgtctaagggacatacgattttttttgcctctgcgtcacgattttaacccaaaagcgagtttagttacatgtacattattat
tagataaagaagtatcgcgaatacttcagttgaataaactgtgcttggtttttgggtgaggatttgtggaaagtagagtgcgcgataaccgtaactttcg
acccggattttcgcctgaattgagatctctctagaggtaccgccaccatggccccaaagaagaagcggaaggtcggtatccacggtgtcccagcagcc
caaatggactcccagcagccagatctgaagctactgtcttctatcgaacaagcatgcgatatttgccgacttaaaaagctcaagtgctccaaagaaaa
accgaagtgcgccaagtgtctgaagaacaactgggagtgtcgctactctcccaaaaccaaaaggtctccgctgactagggcacatctgacagaagtg
gaatcaaggctagaaagactggaacagctatttctactgatttttcctcgagaagaccttgacatgattttgaaaatggattctttacaggatataaaa
gcattgttagaattcccgggtgtcgaccagaaaaagttcaataaagtcagagttgtgagagcactggatgctgttgctctcccacagccagtgggcgtt
ccaaatgaaagccaagccctaagccagagattcactttttcaccaggtcaagacatacagttgattccaccactgatcaacctgttaatgagcattgaa
ccagatgtgatctatgcaggacatgacaacacaaaacctgacacctccagttctttgctgacaagtcttaatcaactaggcgagaggcaacttctttca
gtagtcaagtggtctaaatcattgccaggttttcgaaacttacatattgatgaccagataactctcattcagtattcttggatgagcttaatggtgtttggt
ctaggatggagatcctacaaacacgtcagtgggcagatgctgtattttgcacctgatctaatactaaatgaacagcggatgaaagaatcatcattctat
tcattatgccttaccatgtggcagatcccacaggagtttgtcaagcttcaagttagccaagaagagttcctctgtatgaaagtattgttacttcttaatac
aattcctttggaagggctacgaagtcaaacccagtttgaggagatgaggtcaagctacattagagagctcatcaaggcaattggtttgaggcaaaaa
ggagttgtgtcgagctcacagcgtttctatcaacttacaaaacttcttgataacttgcatgatcttgtcaaacaacttcatctgtactgcttgaatacattt
atccagtcccgggcactgagtgttgaatttccagaaatgatgtctgaagttattgctgggtcgacgagatatcaagcagaattccagtacctgccagat
acagacgatcgtcaccggattgaggagaaacgtaaaaggacatatgagaccttcaagagcatcatgaagaagagtcctttcagcggacccaccgac
ccccggcctccacctcgacgcattgctgtgccttcccgcagctcagcttctgtccccaagccagcaccccagccctatccctttacgtcatccctgagcac
catcaactatgatgagtttcccaccatggtgtttccttctgggcagatcagccaggcctcggccttggccccggcccctccccaagtcctgccccaggct
ccagcccctgcccctgctccagccatggtatcagctctggcccaggccccagcccctgtcccagtcctagccccaggccctcctcaggctgtggcccca
cctgcccccaagcccacccaggctggggaaggaacgctgtcagaggccctgctgcagctgcagtttgatgatgaagacctgggggccttgcttggcaa
cagcacagacccagctgtgttcacagacctggcatccgtcgacaactccgagtttcagcagctgctgaaccagggcatacctgtggcccccacacaa
ctgagcccatgctgatggagtaccctgaggctataactcgcctagtgacaggggcccagaggcccccgacccagctcctgctccactgggggccccg
gggctccccaatggcctcctttcaggagatgaagacttctcctccattgcggacatggacttctcagccctgctgagtcagatcagctcctaatctagca
cctaggaggatccactcgaggtttagagagggcgaatccagctctggagcagaggctctggcagcttttgcagcgtttatataacatgaaatatatata
cgcattccgatcaaagctgggttaaccagatagatagatagtaacgtttaaatagcgcctggcgcgttcgattttaaagagatttagagcgttatcccg
tgcctatagatcatatagtatagacaacgaacgatcactcaaatccaagtcaataattcaagaatttatgtctgtttctgtgaaagggaaactaattttg
ttaaagaagacttacaatatcgtaatacttgttcaatcgtcgtggccgatagaaatatcttacaatccgaaagttgatgaatggaattggtctgcaactg
gtcgccttcatttcgtaaaatgttcgcttgcggccgaaaaatttcgatatatctacaattcatctacaatctttactaaattttgaaaaaggaacactttg
aatttcgaactgtcaatcgtatcattagaatttaatctaaatttaaatcttgctaaaggaaatagcaaggaacactttcgtcgtcggctacgcattcattg
taaaattttaaattttgacattccgcactttttgatagataagcgaagagtatttttattacatgtatcgcaagtattcatttcaacacacatatctatatat
atatatatatatatatatatatatatatatatatgttatatatttattcaattttgtttaccattgatcaatttttcacacatgaaacaaccgccagcatt
atataattttttttattttttttaaaaaatgtgtacacatattctgaaaatgaaaaattcaatggctcgactgccaaataaagaaatggttacaatttaagga
aacaaatgtccttcttgcactagggggggtgacatccctaggtccggagaatgtttaaacagaagttcctattccgaagttcctattctctagaaagtata
ggaacttctcgtagtgccccaactggggtaacctttgagttctctcagttgggggcgtagcggtctcgaagccgcggtgcgggtgccagggcgtgccct
tgggctccccgggcgcgtactccacctcacccatctggtccacctgcagggacgtc

p-yMCR.EGFP

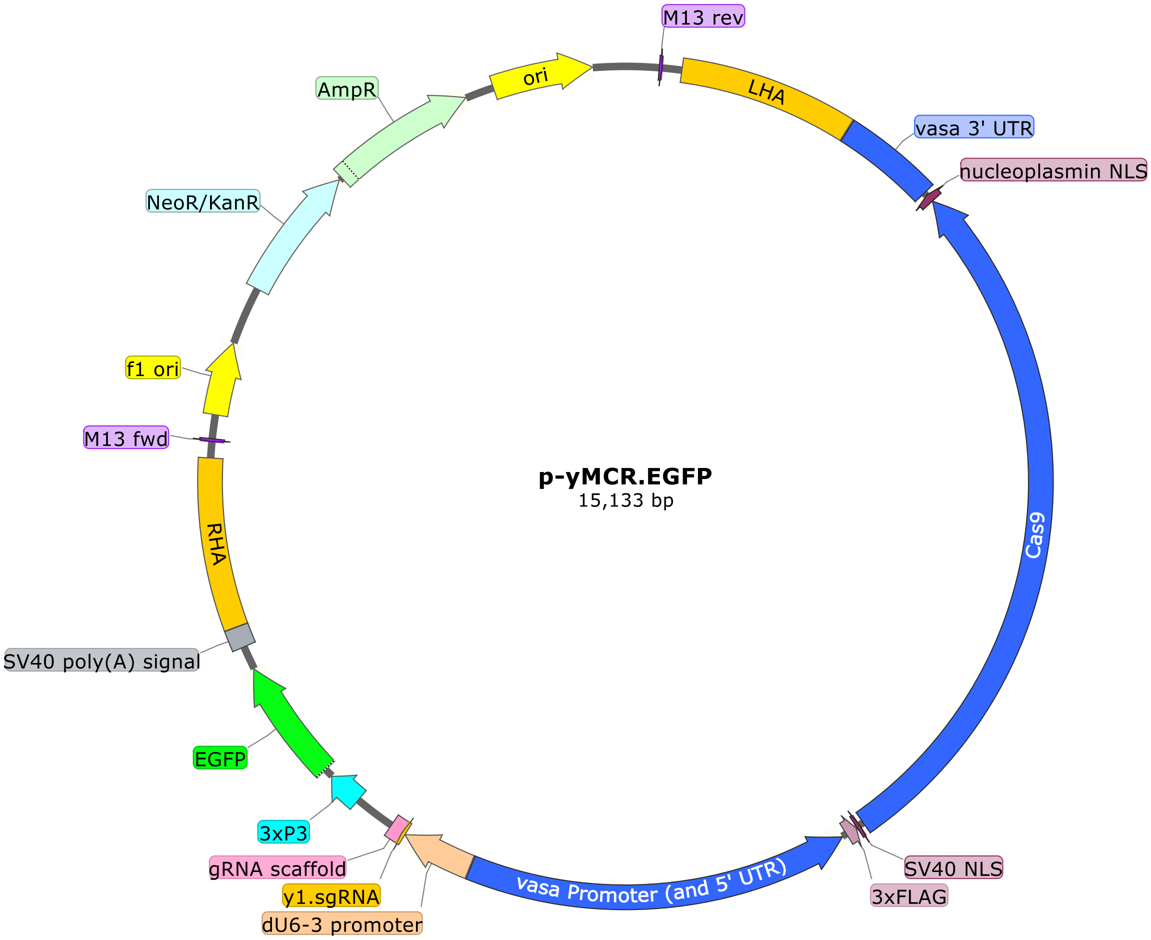

ATACGCAAACCGCCTCTCCCCGCGCGTTGGCCGATTCATTAATGCAGCTGGCACGACAGGTTTCCCGACTGGAAAG
CGGGCAGTGAGCGCAACGCAATTAATGTGAGTTAGCTCACTCATTAGGCACCCCAGGCTTTACACTTTATGCTTCC
GGCTCGTATGTTGTGTGGAATTGTGAGCGGATAACAATTTCACACAGGAAACAGCTATGACCATGATTACGCCAA
GCTATTTAGGTGACACTATAGAATACTCAAGCTATGCATCAAGCTTGGTACCGAGCTCGGATCCACTAGTAACGGC
CGCCAGTGTGCTGGAATTCGCCCTTGTTGCGAGGTTTTAGGACTGAAAGAGCACATGTCAAAATATAAATTTGTTC
AAATACTTTATATTTGACTGAATTAGATTGTTATTTTAAAAGTTATGAATTAAATAAAGATTGAAAGGTGCATTATG
CTCAAATGTATATTTATCGCAACCCCCGGTTACTTTGTAAAGCAAAAACGCCTGGTTTGATTTTTAAGAAGATGGGT
CGGTAAATCGATAAAAGCTATATTTTCTGGTCGTTGCAGTCTCACTCGCCTGCTATAAAAACATTAAAAGTTCCCAG
AAACAATAAATGTCTTTAAATTCAATTAACGAAGAAATAAAGAAGGAAAAGAACTGGAGCGGAAATCGGTCGAAA
TACTGCCAATGGCCACATATACATTTAACAGCGATATATGGTATACATATTGATAATGATGTCAGACGCAATTGCTT
CAGACGGCTAATGACATCGCAAATTGCACGCAACTTGCAATAGTGCCAATTATGACTGAAGTACATATAGCCGGG
GATCTTTTAACATAAACTTCCAGTAGATGTACAAGCAGAAAAAAGAGCCATTAGCACGGCAGTTACCATTGCTTAT
GATTCCTTGTGTCCAAAATAATGACAAATAGGTATATAAATAATTAAATGCCAAACATAAGCGATTCTAATTTACCT
TTACATCTGTATGCATTTACATATTATCCAGAAAACAGACAGCGATAACTTGCAACATTGCTTAGTATAATAATCCA

AAGAAGGAATTTAGGCAGAAATTCCAGTTAATTAAATATTCAAAACAAACTTTATTTAGTGCCTCAATAATAGTTTG
GCCCTGCTAATTCTCCTATTTTATTTTTTAGGGATTCCGGCCACTCTGACCTATATAAACATGGACCGCAGTTTGACG
GGTTCACCGGAGCTAATTCCGTATCCAGATTGGCGCTCAAATACAGCTGGAGATTGCGCCAACAGTATTACCACTG
CCTACCGCATTAAAGTGGATGAGTGTGGTCGGCTGTGGGTTTTGGACACTGGAAGCTAGCAACACGAAGAGCAG
CAGTGTGGTGGATTTCTCAGTTATTGATTTTGCTTCTGCAAAGACAGCAAACGCAAAAGGGCAACATTTGCTGGTC
GGCATGCTTTTCGGGGGAGGAGGACTGGGCGCGAGCAGGGCGAGGCATGCATTCAAAAAGCCGGCTCCAACGG
AGAGCGAGAGAGCGCAAGAGCCAAGTGTACAGCAAGGCAAACAACAAACAACGAAACACGAAGGATGAGTACT
TAAAACCAAACACGTGATAGTACACGGTATTAGTTGGGATCAAAGTGCACGTTTCTGTTAAGTTTTGCAACCGCTC
GTTATTAAAAGCAGTTCACAAAATACTTAAAAGAAATCGAATTAGAGCCAGAACATTTTTTCAAATTATATTTTGTT
ATACATTTCTTTGTAAAAGGAAATTAAAATAGAAAATTCGAATGGCTTGCATATCCTAAATATGCATTTTATTATTAT
ATGCGTTATAATACATATAATTAATATGTTATTAATATCTAACTTATTTAACTAATTACATCAATTACTGCATTACATT
TAATTATTTGAAATCTATGTCCATACATTgagctcaggcctagattaCTTTTTCTTTTTTGCCTGGCCGGCCTTTTTCGTGGC
CGCCGGCCTTTTGTCGCCTCCCAGCTGAGACAGGTCGATCCGTGTCTCGTACAGGCCGGTGATGCTCTGGTGGATC
AGGGTGGCGTCCAGCACCTCTTTGGTGCTGGTGTACCTCTTCCGGTCGATGGTGGTGTCAAAGTACTTGAAGGCG
GCAGGGGCTCCCAGATTGGTCAGGGTAAACAGGTGGATGATATTCTCGGCCTGCTCTCTGATGGGCTTATCCCGG
TGCTTGTTGTAGGCGGACAGCACTTTGTCCAGATTAGCGTCGGCCAGGATCACTCTCTTGGAGAACTCGCTGATCT
GCTCGATGATCTCGTCCAGGTAGTGCTTGTGCTGTTCCACAAACAGCTGTTTCTGCTCATTATCCTCGGGGGAGCCC
TTCAGCTTCTCATAGTGGCTGGCCAGGTACAGGAAGTTCACATATTTGGAGGGCAGGGCCAGTTCGTTTCCCTTCT
GCAGTTCGCCGGCAGAGGCCAGCATTCTCTTCCGGCCGTTTTCCAGCTCGAACAGGGAGTACTTAGGCAGCTTGAT
GATCAGGTCCTTTTTCACTTCTTTGTAGCCCTTGGCTTCCAGAAAGTCGATGGGATTCTTCTCGAAGCTGCTTCTTTC
CATGATGGTGATCCCCAGCAGCTCTTTCACACTCTTCAGTTTCTTGGACTTGCCCTTTTCCACTTTGGCCACCACCAG
CACAGAATAGGCCACGGTGGGGCTGTCGAAGCCGCCGTACTTCTTAGGGTCCCAGTCCTTCTTTCTGGCGATCAGC
TTATCGCTGTTCCTCTTGGGCAGGATAGACTCTTTGCTGAAGCCGCCTGTCTGCACCTCGGTCTTTTTCACGATATTC
ACTTGGGGCATGCTCAGCACTTTCCGCACGGTGGCAAAATCCCGGCCCTTATCCCACACGATCTCCCCGGTTTCGCC
GTTTGTCTCGATCAGAGGCCGCTTCCGGATCTCGCCGTTGGCCAGGGTAATCTCGGTCTTGAAAAAGTTCATGATG
TTGCTGTAGAAGAAGTACTTGGCGGTAGCCTTGCCGATTTCCTGCTCGCTCTTGGCGATCATCTTCCGCACGTCGTA
CACCTTGTAGTCGCCGTACACGAACTCGCTTTCCAGCTTAGGGTACTTTTTGATCAGGGCGGTTCCCACGACGGCG
TTCAGGTAGGCGTCGTGGGCGTGGTGGTAGTTGTTGATCTCGCGCACTTTGTAAAACTGGAAATCCTTCCGGAAAT
CGGACACCAGCTTGGACTTCAGGGTGATCACTTTCACTTCCCGGATCAGCTTGTCATTCTCGTCGTACTTAGTGTTC
ATCCGGGAGTCCAGGATCTGTGCCACGTGCTTTGTGATCTGCCGGGTTTCCACCAGCTGTCTCTTGATGAAGCCGG
CCTTATCCAGTTCGCTCAGGCCGCCTCTCTCGGCCTTGGTCAGATTGTCGAACTTTCTCTGGGTAATCAGCTTGGCG
TTCAGCAGCTGCCGCCAGTAGTTCTTCATCTTCTTCACGACCTCTTCGGAGGGCACGTTGTCGCTCTTGCCCCGGTT
CTTGTCGCTTCTGGTCAGCACCTTGTTGTCGATGGAGTCGTCCTTCAGAAAGCTCTGAGGCACGATATGGTCCACA
TCGTAGTCGGACAGCCGGTTGATGTCCAGTTCCTGGTCCACGTACATATCCCGCCCATTCTGCAGGTAGTACAGGT
ACAGCTTCTCGTTCTGCAGCTGGGTGTTTTCCACGGGGTGTTCTTTCAGGATCTGGCTGCCCAGCTCTTTGATGCCC
TCTTCGATCCGCTTCATTCTCTCGCGGCTGTTCTTCTGTCCCTTCTGGGTGGTCTGGTTCTCTCTGGCCATTTCGATCA
CGATGTTCTCGGGCTTGTGCCGGCCCATCACTTTCACGAGCTCGTCCACCACCTTCACTGTCTGCAGGATGCCCTTC
TTAATGGCGGGGCTGCCGGCCAGATTGGCAATGTGCTCGTGCAGGCTATCGCCCTGGCCGGACACCTGGGCTTTC
TGGATGTCCTCTTTAAAGGTCAGGCTGTCGTCGTGGATCAGCTGCATGAAGTTTCTGTTGGCGAAGCCGTCGGACT
TCAGGAAATCCAGGATTGTCTTGCCGGACTGCTTGTCCCGGATGCCGTTGATCAGCTTCCGGCTCAGCCTGCCCCA
GCCGGTGTATCTCCGCCGCTTCAGCTGCTTCATCACTTTGTCGTCGAACAGGTGGGCATAGGTTTTCAGCCGTTCCT
CGATCATCTCTCTGTCCTCAAACAGTGTCAGGGTCAGCACGATATCTTCCAGAATGTCCTCGTTTTCCTCATTGTCCA
GGAAGTCCTTGTCCTTGATAATTTTCAGCAGATCGTGGTATGTGCCCAGGGAGGCGTTGAACCGATCTTCCACGCC
GGAGATTTCCACGGAGTCGAAGCACTCGATTTTCTTGAAGTAGTCCTCTTTCAGCTGCTTCACGGTCACTTTCCGGT

TGGTCTTGAACAGCAGGTCCACGATGGCCTTTTTCTGCTCGCCGCTCAGGAAGGCGGGCTTTCTCATTCCCTCGGT
CACGTATTTCACTTTGGTCAGCTCGTTATACACGGTGAAGTACTCGTACAGCAGGCTGTGCTTGGGCAGCACCTTCT
CGTTGGGCAGGTTCTTATCGAAGTTGGTCATCCGCTCGATGAAGCTCTGGGCGGAAGCGCCCTTGTCCACCACTTC
CTCGAAGTTCCAGGGGGTGATGGTTTCCTCGCTCTTTCTGGTCATCCAGGCGAATCTGCTGTTTCCCCTGGCCAGA
GGGCCCACGTAGTAGGGGATGCGGAAGGTCAGGATCTTCTCGATCTTTTCCCGGTTGTCCTTCAGGAATGGGTAA
AAATCTTCCTGCCGCCGCAGAATGGCGTGCAGCTCTCCCAGGTGGATCTGGTGGGGGATGCTGCCGTTGTCGAAG
GTCCGCTGCTTCCGCAGCAGGTCCTCTCTGTTCAGCTTCACGAGCAGTTCCTCGGTGCCGTCCATCTTTTCCAGGAT
GGGCTTGATGAACTTGTAGAACTCTTCCTGGCTGGCTCCGCCGTCAATGTAGCCGGCGTAGCCGTTCTTGCTCTGG
TCGAAGAAAATCTCTTTGTACTTCTCAGGCAGCTGCTGCCGCACGAGAGCTTTCAGCAGGGTCAGGTCCTGGTGGT
GCTCGTCGTATCTCTTGATCATAGAGGCGCTCAGGGGGGCCTTGGTGATCTCGGTGTTCACTCTCAGGATGTCGCT
CAGCAGGATGGCGTCGGACAGGTTCTTGGCGGCCAGAAACAGGTCGGCGTACTGGTCGCCGATCTGGGCCAGCA
GGTTGTCCAGGTCGTCGTCGTAGGTGTCCTTGCTCAGCTGCAGTTTGGCATCCTCGGCCAGGTCGAAGTTGCTCTT
GAAGTTGGGGGTCAGGCCCAGGCTCAGGGCAATCAGGTTGCCGAACAGGCCATTCTTCTTCTCGCCGGGCAGCTG
GGCGATCAGATTTTCCAGCCGTCTGCTCTTGCTCAGTCTGGCAGACAGGATGGCCTTGGCGTCCACGCCGCTGGCG
TTGATGGGGTTTTCCTCGAACAGCTGGTTGTAGGTCTGCACCAGCTGGATGAACAGCTTGTCCACGTCGCTGTTGT
CGGGGTTCAGGTCGCCCTCGATCAGGAAGTGGCCCCGGAACTTGATCATGTGGGCCAGGGCCAGATAGATCAGC
CGCAGGTCGGCCTTGTCGGTGCTGTCCACCAGTTTCTTTCTCAGGTGGTAGATGGTGGGGTACTTCTCGTGGTAGG
CCACCTCGTCCACGATGTTGCCGAAGATGGGGTGCCGCTCGTGCTTCTTATCCTCTTCCACCAGGAAGGACTCTTCC
AGTCTGTGGAAGAAGCTGTCGTCCACCTTGGCCATCTCGTTGCTGAAGATCTCTTGCAGATAGCAGATCCGGTTCT
TCCGTCTGGTGTATCTTCTTCTGGCGGTTCTCTTCAGCCGGGTGGCCTCGGCTGTTTCGCCGCTGTCGAACAGCAG
GGCTCCGATCAGGTTCTTCTTGATGCTGTGCCGGTCGGTGTTGCCCAGCACCTTGAATTTCTTGCTGGGCACCTTGT
ACTCGTCGGTGATCACGGCCCAGCCCACAGAGTTGGTGCCGATGTCCAGGCCGATGCTGTACTTCTTGTCGGCTGC
TGGGACTCCGTGGATACCGACCTTCCGCTTCTTCTTTGGGGCCATCTTATCGTCATCGTCTTTGTAATCAATATCATG
ATCCTTGTAGTCTCCGTCGTGGTCCTTATAGTCCATtctaggcgcgccagatctggtaccgATTGATATTTTTTTTTAATTTG
GCCTGCCTTTCAAGAGCAAATTAATTCTTTTTCACTTTATGTATTACCTTACAAACTTTGATAAGTTCAGATCACTTC
GCGCAACGCAAAACCGAAAAGGATCTGTTAAAAGTACCAAAAATCTAGTGGTGGAATTTCCCATTGTGCTATCGCA
ATTCGATATTCACAATTTCGATAATTTATATATATAGCCGTTTTCCACTAACACATCAAATGTGATTTAGCTATTAAT
GAAATTGTAATATTCAAGCAACCTAAGTGATTCATTCATATGTACATACTTATATGTAAAGATGGTGGTGGCATTTT
TAACATGGGGAAGTTCTTATTTATTTACTTTTTCGTTTGTTATAGGTGAATTCCTCTTTATTTTTATACAATATAATTT
TATTTTACGTTAAGCAACCAATTTAAGGGCTATGAAACGTAGTAACTAACAGGAGCAACGAAGATAACATGGAAA
CATACAAAATTTGTATAAAAATATTGATTAGATAATAGTTTGGCTTGTTTATGCCACGTGTCCAAAGATATCTTTGA
TGACGCCTCCTCAGTGTTCTCCTGGTTGTCTGGTATGTGCCTGATTTAATTTGTTCTCAAACTGAAGGTGGATGCTG
AGGTCATGTGCGATATCAACATTTCTCGCGTACCAGGGCGCGTTGGTGATTGTTCGCAACGCTTGGTTTTGGAGCC
GTTGCCCTCTCACTATGTCCCACCGCCGATTTGACCCGCAGACGTATCCCGCTTGTCCCCAACAACCGCACTTCATC
ATAATACATCATCTCTAGACCACACTCAAACGATTTATTTATTAAAACTAAGTGATATATAGGGAATTGTTATTGAA
GGTAATTAGTATAATTCAAAAACGCCCAACACGCTGTTCTTTCATTGAAATACAGTGGAGCCCTGCTTGCAAGCAG
TGCAAGTTTAAAAAACATCGCAATGCTATGTGTGTGTACTCGCTTAAAAGCCAGATGTACATTAAAATACGGTAAC
ACTGTTGACTTCTGAAACCCAAACTACTTCCAGTTGGGAATTGTATTAGCCATGGGAAGGCATTTCTTGACAAGCC
ACCGCCCGGTAACACTAAAACGCGTTAGGGTGTTCTGTCAACTCGTTCTGCGAATACAGAAACGAAAAAAATAGT
AAACAACATTTATTGGAGGGAAAAAAAGAAATAGCCCGGCTTCCGCATAAACAACTGTCGCAGACCCGCAGCACC
GATTAAAGCCGACAGCCGACGCCAGTTAACCTAACCTCTGATCAAATTAAGTGGGCCAAAATGAGCGTGGAGGAG
GAAGTGTTTCGAATCCAAAAGAAGATGAGCAAGATGGCCAGCGACGGCACAGTAAGTATCGGGATTAGCTAACG
TGCCGCGCATCATGTCACAAATATACATATATCCACCGTACAGGGACAGGATCAGGCTCTGGACCTGCTGAAGGC
CCTGCAAACGCTTAACATCAATCTCGACATTCTGACCAAAACGCGCATCGGCATGACCGTAAACGAGCTGCGCAAG

AGTAGCAAGGACGACGAGGTGATCGCTCTGGCCAAGACGCTGATCAAGAACTGGAAACGATTCCTCGCCAGCCC
GGCGCCAACCACACCGAACAACAGCTCCGCTAAGGAGGGCTCCTCCAACAACAGCAGTGCCTCGAAGTCCACCAG
TGCCGCCAAGTCGTCGTCGTCAATCTCCGGCAAGGATAAGTCCAGTTCATCCAGCTCATCCAAGGATAAAGAGAA
GAAAGGCTCGACATCCTCGTCACAGACCTCGTTTCCCTCCGGCGGCATGACAGATGCGGTCCGCATCAAATGCCGC
GAAATGCTGGCCACCGCACTGAAGATTGGTGAAGTGCCCGAAGGATGCGGTGAGCCGGAGGAAATGGCCGCCG
AACTGGAGGATGCCATTTACTCCGAGTTCAATAACACGGATATGAAGTACAAGAATCGCATTAGGTCACGCGTGG
CCAATCTGAAGGACCCCAAGAATCCTGGATTGCGCGGCAACTTTATGTGCGGCGCCGTCACTGCCAAGCAGCTGG
CCAAAATGACGCCGGAGGAGATGGCCAGCGACGAGATGAAGAAGCTGCGCGAGAAGTTCGTCAAGGAGGCCAT
CAACGATGCCCAGCTGGCCACCGTGCAGGGCACCAAGACCGATCTCCTCAAGTGCGCCAAGTGCAAGAAGCGCAA
CTGCACCTACAACCAGCTGCAGGGATCCTTTTTTGCTCACCTGTGATTGCTCCTACTCAAATACAAAAACATCAAAT
TTTCTGTCAATAAAGCATATTTATTTATATTTATTTTACAGGAAAGAATTCCTTTTAAAGTGTATTTTAACCTATAATG
AAAAACGATTAAAAAAAATACATAAAATAATTCGAAAATTTTTGAATAGCCCAGGTTGATAAAAATTCATTTCATAC
GTTTTATAACTTATGCCCCTAAGTATTTTTTGACCATAGTGTTTCAATTCTACATTAATTTTACAGAGTAGAATGAAA
CGCCACCTACTCAGCCAAGAGGCGAAAAGGTTAGCTCGCCAAGCAGAGAGGGCGCCAGTGCTCACTACTTTTTAT
AATTCTCAACTTCTTTTTCCAGACTCAGTTCGTATATATAGACCTATTTTCAATTTAACGTCGGTTTTGGACACTGGA
ACCGGTTTTAGAGCTAGAAATAGCAAGTTAAAATAAGGCTAGTCCGTTATCAACTTGAAAAAGTGGCACCGAGTC
GGTGCTTTTTTGCCTACCTGGAGCCTGAGAGTTGTTCAATAAAATAAAAATGTTTCGTTTTTTTGCTTTCGCCAGTAT
TTATTATTTTTCATCAATATGTATTCAATTTGGTATGTATTTAGTAATTGTAATATATAGACAATGGTTTTCCGTTGAC
GTACATACATCTGACGTGTGTTTATTTAGACATAATAGTTATGTTTTCACATCTTTTTAATGTTCGCTTAATGCGTAT
GCATTTGACGTGGATCTAATTCAATTAGAGACTAATTCAATTAGAGCTAATTCAATTAGGATCCAAGCTTATCGATT
TCGAACCCTCGACCGCCGGAGTATAAATAGAGGCGCTTCGTCTACGGAGCGACAATTCAATTCAAACAAGCAAAG
TGAACACGTCGCTAAGCGAAAGCTAAGCAAATAAACAAGCGCAGCTGAACAAGCTAAACAATCGGGGTACCGCT
AGAGTCGACGGTACCGCGGGCCCGGGATCCACCGGTCGCCACCATGGTGAGCAAGGGCGAGGAGCTGTTCACCG
GGGTGGTGCCCATCCTGGTCGAGCTGGACGGCGACGTAAACGGCCACAAGTTCAGCGTGTCCGGCGAGGGCGAG
GGCGATGCCACCTACGGCAAGCTGACCCTGAAGTTCATCTGCACCACCGGCAAGCTGCCCGTGCCCTGGCCCACCC
TCGTGACCACCCTGACCTACGGCGTGCAGTGCTTCAGCCGCTACCCCGACCACATGAAGCAGCACGACTTCTTCAA
GTCCGCCATGCCCGAAGGCTACGTCCAGGAGCGCACCATCTTCTTCAAGGACGACGGCAACTACAAGACCCGCGC
CGAGGTGAAGTTCGAGGGCGACACCCTGGTGAACCGCATCGAGCTGAAGGGCATCGACTTCAAGGAGGACGGCA
ACATCCTGGGGCACAAGCTGGAGTACAACTACAACAGCCACAACGTCTATATCATGGCCGACAAGCAGAAGAACG
GCATCAAGGTGAACTTCAAGATCCGCCACAACATCGAGGACGGCAGCGTGCAGCTCGCCGACCACTACCAGCAGA
ACACCCCCATCGGCGACGGCCCCGTGCTGCTGCCCGACAACCACTACCTGAGCACCCAGTCCGCCCTGAGCAAAG
ACCCCAACGAGAAGCGCGATCACATGGTCCTGCTGGAGTTCGTGACCGCCGCCGGGATCACTCTCGGCATGGACG
AGCTGTACAAGTAAAGCGGCCTAAGCGATCGCTCAGGCCGCGACTCTAGATCATAATCAGCCATACCACATTTGTA
GAGGTTTTACTTGCTTTAAAAAACCTCCCACACCTCCCCCTGAACCTGAAACATAAAATGAATGCAATTGTTGTTGT
TAACTTGTTTATTGCAGCTTATAATGGTTACAAATAAAGCAATAGCATCACAAATTTCACAAATAAAGCATTTTTTTC
ACTGCATTCTAGTTGTGGTTTGTCCAAACTCATCAATGTATCTTACTCCGTGGGCATCGGCAATACCACCACTAATC
CGTGCCCCTATGCGGTAAATGTCTTTGACTTGACCACGGATACGCGAATTCGGAGATACGAGCTACCTGGCGTGG
ACACAAATCCAAATACTTTCATAGCTAACATTGCCGTGGATATAGGCAAAAATTGCGATGATGCATATGCCTATTTT
GCCGATGAATTGGGATACGGCTTGATTGCTTACTCCTGGGAACTGAACAAGTCCTGGAGATTCTCGGCACATTCGT
ATTTTTTCCCCGATCCATTGAGGGGCGATTTCAATGTCGCTGGTATTAACTTCCAATGGGGCGAGGAGGGTATATT
TGGTATGTCCCTTTCGCCCATTCGATCGGATGGTTATCGTACCCTGTACTTTAGTCCGTTAGCAAGTCATCGACAAT
TTGCCGTATCCACGAGGATTTTGAGGGATGAAACCAGGACGGAAGATAGCTATCATGACTTTGTTGCCTTAGATG
AACGGGGTCCAAACTCCCATACCACTTCACGTGTGATGAGCGATGATGGAATTGAGCTGTTCAATTTAATAGATCA
AAATGCAGTGGGTTGCTGGCACTCATCAATGCCGTACTCACCGCAATTTCATGGCATTGTGGATCGCGATGACGTT

GGCTTAGTTTTTCCGGCCGATGTGAAAATTGATGAGAACAAAAACGTTTGGGTTCTATCCGATAGGATGCCCGTTT
TCTTGCTGTCTGACTTGGATTATTCAGATACTAATTTCCGAATTTACACGGCTCCCTTGGCCACTTTAATTGAGAATA
CTGTGTGTGATTTGAGGAATAACGCCTATGGGCCGCCAAATACCGTTTCAATACCAAAACAAGCCGTTTTGCCAAT
GGGTCCACCGTTATATACGAAACAATATCGTCCTGTCTTGCCACAGAAACCTCAGACCAGCTGGGCTTCCTCGCCG
CCTCCTCCAAGTCGCACTTATTTGCCCGCCAATTCAGGCAATGTAGTCTCCAGAAGGGCGAATTCTGCAGATATCCA
TCACACTGGCGGCCGCTCGAGCATGCATCTAGAGGGCCCAATTCGCCCTATAGTGAGTCGTATTACAATTCACTGG
CCGTCGTTTTACAACGTCGTGACTGGGAAAACCCTGGCGTTACCCAACTTAATCGCCTTGCAGCACATCCCCCTTTC
GCCAGCTGGCGTAATAGCGAAGAGGCCCGCACCGATCGCCCTTCCCAACAGTTGCGCAGCCTGAATGGCGAATGG
ACGCGCCCTGTAGCGGCGCATTAAGCGCGGCGGGTGTGGTGGTTACGCGCAGCGTGACCGCTACACTTGCCAGC
GCCCTAGCGCCCGCTCCTTTCGCTTTCTTCCCTTCCTTTCTCGCCACGTTCGCCGGCTTTCCCCGTCAAGCTCTAAATC
GGGGGCTCCCTTTAGGGTTCCGATTTAGTGCTTTACGGCACCTCGACCCCAAAAAACTTGATTAGGGTGATGGTTC
ACGTAGTGGGCCATCGCCCTGATAGACGGTTTTTCGCCCTTTGACGTTGGAGTCCACGTTCTTTAATAGTGGACTCT
TGTTCCAAACTGGAACAACACTCAACCCTATCTCGGTCTATTCTTTTGATTTATAAGGGATTTTGCCGATTTCGGCCT
ATTGGTTAAAAAATGAGCTGATTTAACAAAAATTTAACGCGAATTTTAACAAAATTCAGGGCGCAAGGGCTGCTAA
AGGAAGCGGAACACGTAGAAAGCCAGTCCGCAGAAACGGTGCTGACCCCGGATGAATGTCAGCTACTGGGCTAT
CTGGACAAGGGAAAACGCAAGCGCAAAGAGAAAGCAGGTAGCTTGCAGTGGGCTTACATGGCGATAGCTAGACT
GGGCGGTTTTATGGACAGCAAGCGAACCGGAATTGCCAGCTGGGGCGCCCTCTGGTAAGGTTGGGAAGCCCTGC
AAAGTAAACTGGATGGCTTTCTTGCCGCCAAGGATCTGATGGCGCAGGGGATCAAGATCTGATCAAGAGACAGG
ATGAGGATCGTTTCGCATGATTGAACAAGATGGATTGCACGCAGGTTCTCCGGCCGCTTGGGTGGAGAGGCTATT
CGGCTATGACTGGGCACAACAGACAATCGGCTGCTCTGATGCCGCCGTGTTCCGGCTGTCAGCGCAGGGGCGCCC
GGTTCTTTTTGTCAAGACCGACCTGTCCGGTGCCCTGAATGAACTGCAGGACGAGGCAGCGCGGCTATCGTGGCT
GGCCACGACGGGCGTTCCTTGCGCAGCTGTGCTCGACGTTGTCACTGAAGCGGGAAGGGACTGGCTGCTATTGG
GCGAAGTGCCGGGGCAGGATCTCCTGTCATCCCACCTTGCTCCTGCCGAGAAAGTATCCATCATGGCTGATGCAAT
GCGGCGGCTGCATACGCTTGATCCGGCTACCTGCCCATTCGACCACCAAGCGAAACATCGCATCGAGCGAGCACG
TACTCGGATGGAAGCCGGTCTTGTCGATCAGGATGATCTGGACGAAGAGCATCAGGGGCTCGCGCCAGCCGAAC
TGTTCGCCAGGCTCAAGGCGCGCATGCCCGACGGCGAGGATCTCGTCGTGACCCATGGCGATGCCTGCTTGCCGA
ATATCATGGTGGAAAATGGCCGCTTTTCTGGATTCATCGACTGTGGCCGGCTGGGTGTGGCGGACCGCTATCAGG
ACATAGCGTTGGCTACCCGTGATATTGCTGAAGAGCTTGGCGGCGAATGGGCTGACCGCTTCCTCGTGCTTTACGG
TATCGCCGCTCCCGATTCGCAGCGCATCGCCTTCTATCGCCTTCTTGACGAGTTCTTCTGAATTGAAAAAGGAAGAG
TATGAGTATTCAACATTTCCGTGTCGCCCTTATTCCCTTTTTTGCGGCATTTTGCCTTCCTGTTTTTGCTCACCCAGAA
ACGCTGGTGAAAGTAAAAGATGCTGAAGATCAGTTGGGTGCACGAGTGGGTTACATCGAACTGGATCTCAACAG
CGGTAAGATCCTTGAGAGTTTTCGCCCCGAAGAACGTTTTCCAATGATGAGCACTTTTAAAGTTCTGCTATGTGGC
GCGGTATTATCCCGTATTGACGCCGGGCAAGAGCAACTCGGTCGCCGCATACACTATTCTCAGAATGACTTGGTTG
AGTACTCACCAGTCACAGAAAAGCATCTTACGGATGGCATGACAGTAAGAGAATTATGCAGTGCTGCCATAACCA
TGAGTGATAACACTGCGGCCAACTTACTTCTGACAACGATCGGAGGACCGAAGGAGCTAACCGCTTTTTTGCACAA
CATGGGGGATCATGTAACTCGCCTTGATCGTTGGGAACCGGAGCTGAATGAAGCCATACCAAACGACGAGCGTG
ACACCACGATGCCTGTAGCAATGGCAACAACGTTGCGCAAACTATTAACTGGCGAACTACTTACTCTAGCTTCCCG
GCAACAATTAATAGACTGGATGGAGGCGGATAAAGTTGCAGGACCACTTCTGCGCTCGGCCCTTCCGGCTGGCTG
GTTTATTGCTGATAAATCTGGAGCCGGTGAGCGTGGGTCTCGCGGTATCATTGCAGCACTGGGGCCAGATGGTAA
GCCCTCCCGTATCGTAGTTATCTACACGACGGGGAGTCAGGCAACTATGGATGAACGAAATAGACAGATCGCTGA
GATAGGTGCCTCACTGATTAAGCATTGGTAACTGTCAGACCAAGTTTACTCATATATACTTTAGATTGATTTAAAAC
TTCATTTTTAATTTAAAAGGATCTAGGTGAAGATCCTTTTTGATAATCTCATGACCAAAATCCCTTAACGTGAGTTTT
CGTTCCACTGAGCGTCAGACCCCGTAGAAAAGATCAAAGGATCTTCTTGAGATCCTTTTTTTCTGCGCGTAATCTGC
TGCTTGCAAACAAAAAAACCACCGCTACCAGCGGTGGTTTGTTTGCCGGATCAAGAGCTACCAACTCTTTTTCCGA

AGGTAACTGGCTTCAGCAGAGCGCAGATACCAAATACTGTTCTTCTAGTGTAGCCGTAGTTAGGCCACCACTTCAA
GAACTCTGTAGCACCGCCTACATACCTCGCTCTGCTAATCCTGTTACCAGTGGCTGCTGCCAGTGGCGATAAGTCG
TGTCTTACCGGGTTGGACTCAAGACGATAGTTACCGGATAAGGCGCAGCGGTCGGGCTGAACGGGGGGTTCGTG
CACACAGCCCAGCTTGGAGCGAACGACCTACACCGAACTGAGATACCTACAGCGTGAGCTATGAGAAAGCGCCAC
GCTTCCCGAAGGGAGAAAGGCGGACAGGTATCCGGTAAGCGGCAGGGTCGGAACAGGAGAGCGCACGAGGGA
GCTTCCAGGGGGAAACGCCTGGTATCTTTATAGTCCTGTCGGGTTTCGCCACCTCTGACTTGAGCGTCGATTTTTGT
GATGCTCGTCAGGGGGGCGGAGCCTATGGAAAAACGCCAGCAACGCGGCCTTTTTACGGTTCCTGGCCTTTTGCT
GGCCTTTTGCTCACATGTTCTTTCCTGCGTTATCCCCTGATTCTGTGGATAACCGTATTACCGCCTTTGAGTGAGCTG
ATACCGCTCGCCGCAGCCGAACGACCGAGCGCAGCGAGTCAGTGAGCGAGGAAGCGGAAGAGCGCCCA

p-ReMEDE.RFP

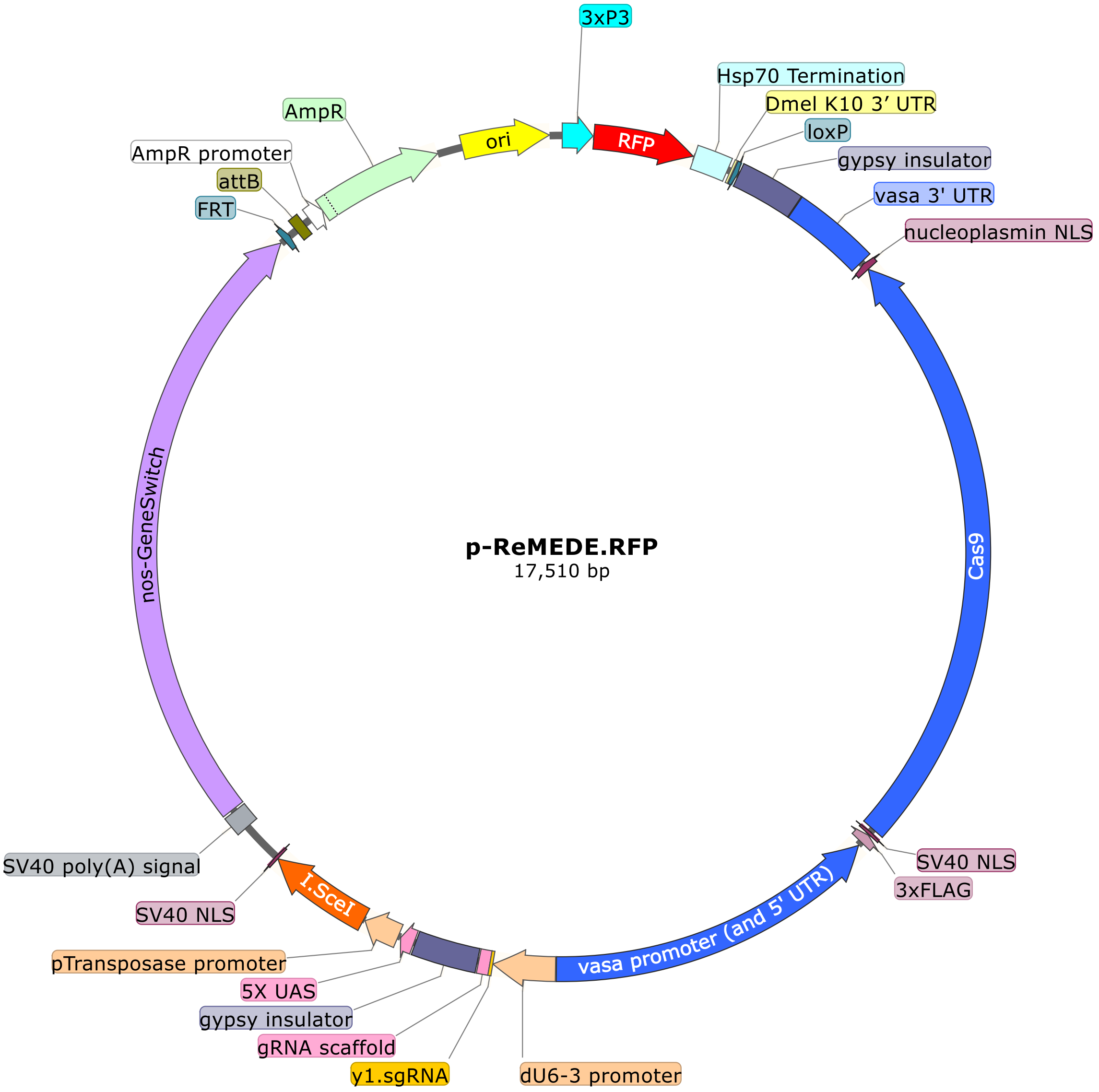

GACGTCGGATCTAATTCAATTAGAGACTAATTCAATTAGAGCTAATTCAATTAGGATCCAAGCTTATCGATTTCGAA
CCCTCGACCGCCGGAGTATAAATAGAGGCGCTTCGTCTACGGAGCGACAATTCAATTCAAACAAGCAAAGTGAAC
ACGTCGCTAAGCGAAAGCTAAGCAAATAAACAAGCGCAGCTGAACAAGCTAAACAATCGGGCCACCATGAGGTCT
TCCAAGAATGTTATCAAGGAGTTCATGAGGTTTAAGGTTCGCATGGAAGGAACGGTCAATGGGCACGAGTTTGAA
ATAGAAGGCGAAGGAGAGGGGAGGCCATACGAAGGCCACAATACCGTAAAGCTTAAGGTAACCAAGGGGGGAC
CTTTGCCATTTGCTTGGGATATTTTGTCACCACAATTTCAGTATGGAAGCAAGGTATATGTCAAGCACCCTGCCGAC
ATACCAGACTATAAAAAGCTGTCATTTCCTGAAGGATTTAAATGGGAAAGGGTCATGAACTTTGAAGACGGTGGC
GTCGTTACTGTAACCCAGGATTCCAGTTTGCAGGATGGCTGTTTCATCTACAAGGTCAAGTTCATTGGCGTGAACTT

TCCTTCCGATGGACCTGTTATGCAAAAGAAGACAATGGGCTGGGAAGCCAGCACTGAGCGTTTGTATCCTCGTGA
TGGCGTGTTGAAAGGAGAGATTCATAAGGCTCTGAAGCTGAAAGACGGTGGTCATTACCTAGTTGAATTCAAAAG
TATTTACATGGCAAAGAAGCCTGTGCAGCTACCAGGGTACTACTATGTTGACTCCAAACTGGATATAACAAGCCAC
AACGAAGACTATACAATCGTTGAGCAGTATGAAAGAACCGAGGGACGCCACCATCTGTTCCTTTAGCGGCCAACG
TCGACTAAAGCCAAATAGAAATTATTCAGTTCTGGCTTAAGTTTTTAAAAGTGATATTATTTATTTGGTTGTAACCA
ACCAAAAGAATGTAAATAACTAATACATAATTATGTTAGTTTTAAGTTAGCAACAAATTGATTTTAGCTATATTAGC
TACTTGGTTAATAAATAGAATATATTTATTTAAAGATAATTCGTTTTTATTGTCAGGGAGTGAGTTTGCTTAAAAAC
TCGTTTAAGCTTATCGATACGCGTACGGCGCGCCAAAGCTTTGTTCATAACTTCGTATAGCATACATTATACGAAGT
TATgTCCGGATGGCCACGTAATAAGTGTGCGTTGAATTTATTCGCAAAAACATTGCATATTTTCGGCAAAGTAAAAT
TTTGTTGCATACCTTATCAAAAAATAAGTGCTGCATACTTTTTAGAGAAACCAAATAATTTTTTATTGCATACCCGTT
TTTAATAAAATACATTGCATACCCTCTTTTAATAAAAAATATTGCATACTTTGACGAAACAAATTTTCGTTGCATACC
CAATAAAAGATTATTATATTGCATACCCGTTTTTAATAAAATACATTGCATACCCTCTTTTAATAAAAAATATTGCAT
ACGTTGACGAAACAAATTTTCGTTGCATACCCAATAAAAGATTATTATATTGCATACCTTTTCTTGCCATACCATTTA
GCCGATCAATTGTGCTCGGCAACAGTATATTTGTGGTGTGCCAACCAACAACatgcccAACACGAAGAGCAGCAGTG
TGGTGGATTTCTCAGTTATTGATTTTGCTTCTGCAAAGACAGCAAACGCAAAAGGGCAACATTTGCTGGTCGGCAT
GCTTTTCGGGGGAGGAGGACTGGGCGCGAGCAGGGCGAGGCATGCATTCAAAAAGCCGGCTCCAACGGAGAGC
GAGAGAGCGCAAGAGCCAAGTGTACAGCAAGGCAAACAACAAACAACGAAACACGAAGGATGAGTACTTAAAAC
CAAACACGTGATAGTACACGGTATTAGTTGGGATCAAAGTGCACGTTTCTGTTAAGTTTTGCAACCGCTCGTTATTA
AAAGCAGTTCACAAAATACTTAAAAGAAATCGAATTAGAGCCAGAACATTTTTTCAAATTATATTTTGTTATACATT
TCTTTGTAAAAGGAAATTAAAATAGAAAATTCGAATGGCTTGCATATCCTAAATATGCATTTTATTATTATATGCGT
TATAATACATATAATTAATATGTTATTAATATCTAACTTATTTAACTAATTACATCAATTACTGCATTACATTTAATTA
TTTGAAATCTATGTCCATACATTgagctcaggcctagattaCTTTTTCTTTTTTGCCTGGCCGGCCTTTTTCGTGGCCGCCG
GCCTTTTGTCGCCTCCCAGCTGAGACAGGTCGATCCGTGTCTCGTACAGGCCGGTGATGCTCTGGTGGATCAGGGT
GGCGTCCAGCACCTCTTTGGTGCTGGTGTACCTCTTCCGGTCGATGGTGGTGTCAAAGTACTTGAAGGCGGCAGG
GGCTCCCAGATTGGTCAGGGTAAACAGGTGGATGATATTCTCGGCCTGCTCTCTGATGGGCTTATCCCGGTGCTTG
TTGTAGGCGGACAGCACTTTGTCCAGATTAGCGTCGGCCAGGATCACTCTCTTGGAGAACTCGCTGATCTGCTCGA
TGATCTCGTCCAGGTAGTGCTTGTGCTGTTCCACAAACAGCTGTTTCTGCTCATTATCCTCGGGGGAGCCCTTCAGC
TTCTCATAGTGGCTGGCCAGGTACAGGAAGTTCACATATTTGGAGGGCAGGGCCAGTTCGTTTCCCTTCTGCAGTT
CGCCGGCAGAGGCCAGCATTCTCTTCCGGCCGTTTTCCAGCTCGAACAGGGAGTACTTAGGCAGCTTGATGATCA
GGTCCTTTTTCACTTCTTTGTAGCCCTTGGCTTCCAGAAAGTCGATGGGATTCTTCTCGAAGCTGCTTCTTTCCATGA
TGGTGATCCCCAGCAGCTCTTTCACACTCTTCAGTTTCTTGGACTTGCCCTTTTCCACTTTGGCCACCACCAGCACAG
AATAGGCCACGGTGGGGCTGTCGAAGCCGCCGTACTTCTTAGGGTCCCAGTCCTTCTTTCTGGCGATCAGCTTATC
GCTGTTCCTCTTGGGCAGGATAGACTCTTTGCTGAAGCCGCCTGTCTGCACCTCGGTCTTTTTCACGATATTCACTT
GGGGCATGCTCAGCACTTTCCGCACGGTGGCAAAATCCCGGCCCTTATCCCACACGATCTCCCCGGTTTCGCCGTTT
GTCTCGATCAGAGGCCGCTTCCGGATCTCGCCGTTGGCCAGGGTAATCTCGGTCTTGAAAAAGTTCATGATGTTGC
TGTAGAAGAAGTACTTGGCGGTAGCCTTGCCGATTTCCTGCTCGCTCTTGGCGATCATCTTCCGCACGTCGTACACC
TTGTAGTCGCCGTACACGAACTCGCTTTCCAGCTTAGGGTACTTTTTGATCAGGGCGGTTCCCACGACGGCGTTCA
GGTAGGCGTCGTGGGCGTGGTGGTAGTTGTTGATCTCGCGCACTTTGTAAAACTGGAAATCCTTCCGGAAATCGG
ACACCAGCTTGGACTTCAGGGTGATCACTTTCACTTCCCGGATCAGCTTGTCATTCTCGTCGTACTTAGTGTTCATCC
GGGAGTCCAGGATCTGTGCCACGTGCTTTGTGATCTGCCGGGTTTCCACCAGCTGTCTCTTGATGAAGCCGGCCTT
ATCCAGTTCGCTCAGGCCGCCTCTCTCGGCCTTGGTCAGATTGTCGAACTTTCTCTGGGTAATCAGCTTGGCGTTCA
GCAGCTGCCGCCAGTAGTTCTTCATCTTCTTCACGACCTCTTCGGAGGGCACGTTGTCGCTCTTGCCCCGGTTCTTG
TCGCTTCTGGTCAGCACCTTGTTGTCGATGGAGTCGTCCTTCAGAAAGCTCTGAGGCACGATATGGTCCACATCGT
AGTCGGACAGCCGGTTGATGTCCAGTTCCTGGTCCACGTACATATCCCGCCCATTCTGCAGGTAGTACAGGTACAG

CTTCTCGTTCTGCAGCTGGGTGTTTTCCACGGGGTGTTCTTTCAGGATCTGGCTGCCCAGCTCTTTGATGCCCTCTTC
GATCCGCTTCATTCTCTCGCGGCTGTTCTTCTGTCCCTTCTGGGTGGTCTGGTTCTCTCTGGCCATTTCGATCACGAT
GTTCTCGGGCTTGTGCCGGCCCATCACTTTCACGAGCTCGTCCACCACCTTCACTGTCTGCAGGATGCCCTTCTTAA
TGGCGGGGCTGCCGGCCAGATTGGCAATGTGCTCGTGCAGGCTATCGCCCTGGCCGGACACCTGGGCTTTCTGGA
TGTCCTCTTTAAAGGTCAGGCTGTCGTCGTGGATCAGCTGCATGAAGTTTCTGTTGGCGAAGCCGTCGGACTTCAG
GAAATCCAGGATTGTCTTGCCGGACTGCTTGTCCCGGATGCCGTTGATCAGCTTCCGGCTCAGCCTGCCCCAGCCG
GTGTATCTCCGCCGCTTCAGCTGCTTCATCACTTTGTCGTCGAACAGGTGGGCATAGGTTTTCAGCCGTTCCTCGAT
CATCTCTCTGTCCTCAAACAGTGTCAGGGTCAGCACGATATCTTCCAGAATGTCCTCGTTTTCCTCATTGTCCAGGA
AGTCCTTGTCCTTGATAATTTTCAGCAGATCGTGGTATGTGCCCAGGGAGGCGTTGAACCGATCTTCCACGCCGGA
GATTTCCACGGAGTCGAAGCACTCGATTTTCTTGAAGTAGTCCTCTTTCAGCTGCTTCACGGTCACTTTCCGGTTGG
TCTTGAACAGCAGGTCCACGATGGCCTTTTTCTGCTCGCCGCTCAGGAAGGCGGGCTTTCTCATTCCCTCGGTCACG
TATTTCACTTTGGTCAGCTCGTTATACACGGTGAAGTACTCGTACAGCAGGCTGTGCTTGGGCAGCACCTTCTCGTT
GGGCAGGTTCTTATCGAAGTTGGTCATCCGCTCGATGAAGCTCTGGGCGGAAGCGCCCTTGTCCACCACTTCCTCG
AAGTTCCAGGGGGTGATGGTTTCCTCGCTCTTTCTGGTCATCCAGGCGAATCTGCTGTTTCCCCTGGCCAGAGGGC
CCACGTAGTAGGGGATGCGGAAGGTCAGGATCTTCTCGATCTTTTCCCGGTTGTCCTTCAGGAATGGGTAAAAATC
TTCCTGCCGCCGCAGAATGGCGTGCAGCTCTCCCAGGTGGATCTGGTGGGGGATGCTGCCGTTGTCGAAGGTCCG
CTGCTTCCGCAGCAGGTCCTCTCTGTTCAGCTTCACGAGCAGTTCCTCGGTGCCGTCCATCTTTTCCAGGATGGGCT
TGATGAACTTGTAGAACTCTTCCTGGCTGGCTCCGCCGTCAATGTAGCCGGCGTAGCCGTTCTTGCTCTGGTCGAA
GAAAATCTCTTTGTACTTCTCAGGCAGCTGCTGCCGCACGAGAGCTTTCAGCAGGGTCAGGTCCTGGTGGTGCTCG
TCGTATCTCTTGATCATAGAGGCGCTCAGGGGGGCCTTGGTGATCTCGGTGTTCACTCTCAGGATGTCGCTCAGCA
GGATGGCGTCGGACAGGTTCTTGGCGGCCAGAAACAGGTCGGCGTACTGGTCGCCGATCTGGGCCAGCAGGTTG
TCCAGGTCGTCGTCGTAGGTGTCCTTGCTCAGCTGCAGTTTGGCATCCTCGGCCAGGTCGAAGTTGCTCTTGAAGT
TGGGGGTCAGGCCCAGGCTCAGGGCAATCAGGTTGCCGAACAGGCCATTCTTCTTCTCGCCGGGCAGCTGGGCGA
TCAGATTTTCCAGCCGTCTGCTCTTGCTCAGTCTGGCAGACAGGATGGCCTTGGCGTCCACGCCGCTGGCGTTGAT
GGGGTTTTCCTCGAACAGCTGGTTGTAGGTCTGCACCAGCTGGATGAACAGCTTGTCCACGTCGCTGTTGTCGGG
GTTCAGGTCGCCCTCGATCAGGAAGTGGCCCCGGAACTTGATCATGTGGGCCAGGGCCAGATAGATCAGCCGCA
GGTCGGCCTTGTCGGTGCTGTCCACCAGTTTCTTTCTCAGGTGGTAGATGGTGGGGTACTTCTCGTGGTAGGCCAC
CTCGTCCACGATGTTGCCGAAGATGGGGTGCCGCTCGTGCTTCTTATCCTCTTCCACCAGGAAGGACTCTTCCAGTC
TGTGGAAGAAGCTGTCGTCCACCTTGGCCATCTCGTTGCTGAAGATCTCTTGCAGATAGCAGATCCGGTTCTTCCG
TCTGGTGTATCTTCTTCTGGCGGTTCTCTTCAGCCGGGTGGCCTCGGCTGTTTCGCCGCTGTCGAACAGCAGGGCT
CCGATCAGGTTCTTCTTGATGCTGTGCCGGTCGGTGTTGCCCAGCACCTTGAATTTCTTGCTGGGCACCTTGTACTC
GTCGGTGATCACGGCCCAGCCCACAGAGTTGGTGCCGATGTCCAGGCCGATGCTGTACTTCTTGTCGGCTGCTGG
GACTCCGTGGATACCGACCTTCCGCTTCTTCTTTGGGGCCATCTTATCGTCATCGTCTTTGTAATCAATATCATGATC
CTTGTAGTCTCCGTCGTGGTCCTTATAGTCCATtctaggcgcgccagatctggtaccgATTGATATTTTTTTTTAATTTGGCC
TGCCTTTCAAGAGCAAATTAATTCTTTTTCACTTTATGTATTACCTTACAAACTTTGATAAGTTCAGATCACTTCGCG
CAACGCAAAACCGAAAAGGATCTGTTAAAAGTACCAAAAATCTAGTGGTGGAATTTCCCATTGTGCTATCGCAATT
CGATATTCACAATTTCGATAATTTATATATATAGCCGTTTTCCACTAACACATCAAATGTGATTTAGCTATTAATGAA
ATTGTAATATTCAAGCAACCTAAGTGATTCATTCATATGTACATACTTATATGTAAAGATGGTGGTGGCATTTTTAA
CATGGGGAAGTTCTTATTTATTTACTTTTTCGTTTGTTATAGGTGAATTCCTCTTTATTTTTATACAATATAATTTTATT
TTACGTTAAGCAACCAATTTAAGGGCTATGAAACGTAGTAACTAACAGGAGCAACGAAGATAACATGGAAACATA
CAAAATTTGTATAAAAATATTGATTAGATAATAGTTTGGCTTGTTTATGCCACGTGTCCAAAGATATCTTTGATGAC
GCCTCCTCAGTGTTCTCCTGGTTGTCTGGTATGTGCCTGATTTAATTTGTTCTCAAACTGAAGGTGGATGCTGAGGT
CATGTGCGATATCAACATTTCTCGCGTACCAGGGCGCGTTGGTGATTGTTCGCAACGCTTGGTTTTGGAGCCGTTG
CCCTCTCACTATGTCCCACCGCCGATTTGACCCGCAGACGTATCCCGCTTGTCCCCAACAACCGCACTTCATCATAAT

ACATCATCTCTAGACCACACTCAAACGATTTATTTATTAAAACTAAGTGATATATAGGGAATTGTTATTGAAGGTAA
TTAGTATAATTCAAAAACGCCCAACACGCTGTTCTTTCATTGAAATACAGTGGAGCCCTGCTTGCAAGCAGTGCAA
GTTTAAAAAACATCGCAATGCTATGTGTGTGTACTCGCTTAAAAGCCAGATGTACATTAAAATACGGTAACACTGT
TGACTTCTGAAACCCAAACTACTTCCAGTTGGGAATTGTATTAGCCATGGGAAGGCATTTCTTGACAAGCCACCGC
CCGGTAACACTAAAACGCGTTAGGGTGTTCTGTCAACTCGTTCTGCGAATACAGAAACGAAAAAAATAGTAAACA
ACATTTATTGGAGGGAAAAAAAGAAATAGCCCGGCTTCCGCATAAACAACTGTCGCAGACCCGCAGCACCGATTA
AAGCCGACAGCCGACGCCAGTTAACCTAACCTCTGATCAAATTAAGTGGGCCAAAATGAGCGTGGAGGAGGAAG
TGTTTCGAATCCAAAAGAAGATGAGCAAGATGGCCAGCGACGGCACAGTAAGTATCGGGATTAGCTAACGTGCC
GCGCATCATGTCACAAATATACATATATCCACCGTACAGGGACAGGATCAGGCTCTGGACCTGCTGAAGGCCCTG
CAAACGCTTAACATCAATCTCGACATTCTGACCAAAACGCGCATCGGCATGACCGTAAACGAGCTGCGCAAGAGT
AGCAAGGACGACGAGGTGATCGCTCTGGCCAAGACGCTGATCAAGAACTGGAAACGATTCCTCGCCAGCCCGGC
GCCAACCACACCGAACAACAGCTCCGCTAAGGAGGGCTCCTCCAACAACAGCAGTGCCTCGAAGTCCACCAGTGC
CGCCAAGTCGTCGTCGTCAATCTCCGGCAAGGATAAGTCCAGTTCATCCAGCTCATCCAAGGATAAAGAGAAGAA
AGGCTCGACATCCTCGTCACAGACCTCGTTTCCCTCCGGCGGCATGACAGATGCGGTCCGCATCAAATGCCGCGAA
ATGCTGGCCACCGCACTGAAGATTGGTGAAGTGCCCGAAGGATGCGGTGAGCCGGAGGAAATGGCCGCCGAACT
GGAGGATGCCATTTACTCCGAGTTCAATAACACGGATATGAAGTACAAGAATCGCATTAGGTCACGCGTGGCCAA
TCTGAAGGACCCCAAGAATCCTGGATTGCGCGGCAACTTTATGTGCGGCGCCGTCACTGCCAAGCAGCTGGCCAA
AATGACGCCGGAGGAGATGGCCAGCGACGAGATGAAGAAGCTGCGCGAGAAGTTCGTCAAGGAGGCCATCAAC
GATGCCCAGCTGGCCACCGTGCAGGGCACCAAGACCGATCTCCTCAAGTGCGCCAAGTGCAAGAAGCGCAACTGC
ACCTACAACCAGCTGCAGGGATCCTTTTTTGCTCACCTGTGATTGCTCCTACTCAAATACAAAAACATCAAATTTTCT
GTCAATAAAGCATATTTATTTATATTTATTTTACAGGAAAGAATTCCTTTTAAAGTGTATTTTAACCTATAATGAAAA
ACGATTAAAAAAAATACATAAAATAATTCGAAAATTTTTGAATAGCCCAGGTTGATAAAAATTCATTTCATACGTTT
TATAACTTATGCCCCTAAGTATTTTTTGACCATAGTGTTTCAATTCTACATTAATTTTACAGAGTAGAATGAAACGCC
ACCTACTCAGCCAAGAGGCGAAAAGGTTAGCTCGCCAAGCAGAGAGGGCGCCAGTGCTCACTACTTTTTATAATT
CTCAACTTCTTTTTCCAGACTCAGTTCGTATATATAGACCTATTTTCAATTTAACGTCGGTTTTGGACACTGGAACCG
GTTTTAGAGCTAGAAATAGCAAGTTAAAATAAGGCTAGTCCGTTATCAACTTGAAAAAGTGGCACCGAGTCGGTG
CgggcatgcgTGGCCACGTAATAAGTGTGCGTTGAATTTATTCGCAAAAACATTGCATATTTTCGGCAAAGTAAAATT
TTGTTGCATACCTTATCAAAAAATAAGTGCTGCATACTTTTTAGAGAAACCAAATAATTTTTTATTGCATACCCGTTT
TTAATAAAATACATTGCATACCCTCTTTTAATAAAAAATATTGCATACTTTGACGAAACAAATTTTCGTTGCATACCC
AATAAAAGATTATTATATTGCATACCCGTTTTTAATAAAATACATTGCATACCCTCTTTTAATAAAAAATATTGCATA
CGTTGACGAAACAAATTTTCGTTGCATACCCAATAAAAGATTATTATATTGCATACCTTTTCTTGCCATACCATTTAG
CCGATCAATTGTGCTCGGCAACAGTATATTTGTGGTGTGCCAACCAACAACCCTAGGatctgCGGAGTACTGTCCTC
CGAGCGGAGTACTGTCCTCCGAGCGGAGTACTGTCCTCCGAGCGGAGTACTGTCCTCCGAGCGGAGTACTGTCCT
CCGAGCGGAGACTCTAGCGAGCGAGCCGTAGCTTACCGAAGTATACACTTAAATTCAGTGCACGTTTGCTTGTTGA
GAGGAAAGGTTGTGTGCGGACGAATTTTTTTTTGAAAACCGGTGATAGAGCCTGAACCAGAAAAGATAAAAGAA
GGCTATACCAGTGGGAGTACACAAACAGAGTAAGTTTGAATAGTAAAAAAAATCATTTATGTAAACAATAACGTG
ACTGTGCGTTAGGTCCTGTTCATTGGTACCCGCCCGGGGATCATCTGTTCTAGAATGAAAAACATCAAAAAAAACC
AGGTAATGAACCTGGGTCCGAACTCTAAACTGCTGAAAGAATACAAATCCCAGCTGATCGAACTGAACATCGAAC
AGTTCGAAGCAGGTATCGGTCTGATCCTGGGTGATGCTTACATCCGTTCTCGTGATGAAGGTAAAACCTACTGTAT
GCAGTTCGAGTGGAAAAACAAAGCATACATGGACCACGTATGTCTGCTGTACGATCAGTGGGTACTGTCCCCGCC
GCACAAAAAAGAACGTGTTAACCACCTGGGTAACCTGGTAATCACCTGGGGCGCCCAGACTTTCAAACACCAAGC
TTTCAACAAACTGGCTAACCTGTTCATCGTTAACAACAAAAAAACCATCCCGAACAACCTGGTTGAAAACTACCTGA
CCCCGATGTCTCTGGCATACTGGTTCATGGATGATGGTGGTAAATGGGATTACAACAAAAACTCTACCAACAAATC
GATCGTACTGAACACCCAGTCTTTCACTTTCGAAGAAGTAGAATACCTGGTTAAGGGTCTGCGTAACAAATTCCAA

CTGAACTGTTACGTAAAAATCAACAAAAACAAACCGATCATCTACATCGATTCTATGTCTTACCTGATCTTCTACAA
CCTGATCAAACCGTACCTGATCCCGCAGATGATGTACAAACTGCCGAACACTATCTCCTCCGAAACTTTCCTGAAAC
CAAAAAAGAAGAGAAAGGTATAAGAAGACCCCAAGGACTTTCCTTCAGAATTGCTAAGTTTTTTGAGTCATGCTGT
GTTTAGTAATAGAACTCTTGCTTGCTTTGCTATTTACACCACAAAGGAAAAAGCTGCACTGCTATACAAGAAAATTA
TGGAAAAATATTTGATGTATAGTGCCTTGACTAGAGATCATAATCAGCCATACCACATTTGTAGAGGTTTTACTTGC
TTTAAAAAACCTCCCACACCTCCCCCTGAACCTGAAACATAAAATGAATGCAATTGTTGTTGTTAACTTGTTTATTGC
AGCTTATAATGGTTACAAATAAAGCAATAGCATCACAAATTTCACAAATAAAGCATTTTTTTCACTGCATTCTAGTT
GTGGTTTGTCCAAACTCATCAATGTATCTTATCATGTCTGGATCgcgcttattgattggcTGGTCCATCATGATGAACGGG
TCGAGGTGGCGGTAGTTGATCCCGGCGAACGCGCGGCGCACCGGGAAGCCCTCGCCCTCGAAACCGCTGGGCGC
GGTGGTCACGGTGAGCACGGGACGTGCGACGGCGTCGGCTGGTGCGGATACGCGGGGCAGCGTCAGCGGGTTC
TCGACGGTCACGGCGGGCATGTCGACAAGCCGAATTGATCCACTAGAAGGCCTAATTCGtCTAGCAGGATCTctagg
GCTTCGACCGTTTTAACCTCGAAATATGCACATGTAAGGACGGATGTGAGCGAACGCCAGTGATGACCGGGATCA
GAGGTAACCTACCATGGTGGGGATTAGGTGACCGTTCGCAGGTAGTTTGATCGGAGCGAATGTTCGGGGGGTCT
GGCGTCAGAGGCTCTAAACTTTATGTAATTCCTGCCGCGAAACACGCACGTATCAAGCAGTCAGCTGTTCTCTTCG
TTCAGCGCGCGCCGGTGTTGCAAAACGAGCGCTCTTCGCCGGCGGTGGCTCGTGCGATAGTTCGTTTTGTCGGTA
ATCCGATGTTGCCGCGCCGATATCATGTGATGTTGTCACAGTGCGCGAAATTCGAATGGTGGTGTGCAGTGATTGT
GTTGTGACGGCGAGTGGCGCGTGTGGGTGCTTAGTTTTGGGAGATGTTTTCGTATTTTTTTGTTGATAACTCAGGC
TTTGTTGCTGTGTTGTAGTACTATTTTCCATTGCGCGGTGTCCAGCTTTTAATTAGTGGCACATATTCTTAGCAAGTA
AAAATTATTTTGCATACTATTAAATTTCTTATAAATTATTTTCTAAAATTAAGTTTACCTTTTCAATTTTACTAAAAAT
ATCGATATATTTATTATCGCTGGAAAACTACATTATTCCACCTCTAAGCAAGAACCGTTAGTTGGCGCGTAGCTTTA
CCACAAAATTCCTGGAATTGCCGTACGCTTCGCAGTTGTTTCAAGTTGTCTAAGGGACATACGATTTTTTTTGCCTCT
GCGTCACGATTTTAACCCAAAAGCGAGTTTAGTTACATGTACATTATTATTAGATAAAGAAGTATCGCGAATACTTC
AGTTGAATAAACTGTGCTTGGTTTTTGGGTGAGGATTTGTGGAAAGTAGAGTGCGCGATAACCGTAACTTTCGACC
CGGATTTTCGCCtgAATTgagatctcTCTAGAggtacCGCCACCATGGCCCCAAAGAAGAAGCGGAAGGTCGGTATCCA
CGGTGTCCCAGCAGCCcaaATGGACTCCCAGCAGCCAGATCTGAAGCTACTGTCTTCTATCGAACAAGCATGCGAT
ATTTGCCGACTTAAAAAGCTCAAGTGCTCCAAAGAAAAACCGAAGTGCGCCAAGTGTCTGAAGAACAACTGGGAG
TGTCGCTACTCTCCCAAAACCAAAAGGTCTCCGCTGACTAGGGCACATCTGACAGAAGTGGAATCAAGGCTAGAA
AGACTGGAACAGCTATTTCTACTGATTTTTCCTCGAGAAGACCTTGACATGATTTTGAAAATGGATTCTTTACAGGA
TATAAAAGCATTGTTAGAATTCCCGGGTGTCGACCAGAAAAAGTTCAATAAAGTCAGAGTTGTGAGAGCACTGGA
TGCTGTTGCTCTCCCACAGCCAGTGGGCGTTCCAAATGAAAGCCAAGCCCTAAGCCAGAGATTCACTTTTTCACCA
GGTCAAGACATACAGTTGATTCCACCACTGATCAACCTGTTAATGAGCATTGAACCAGATGTGATCTATGCAGGAC
ATGACAACACAAAACCTGACACCTCCAGTTCTTTGCTGACAAGTCTTAATCAACTAGGCGAGAGGCAACTTCTTTCA
GTAGTCAAGTGGTCTAAATCATTGCCAGGTTTTCGAAACTTACATATTGATGACCAGATAACTCTCATTCAGTATTC
TTGGATGAGCTTAATGGTGTTTGGTCTAGGATGGAGATCCTACAAACACGTCAGTGGGCAGATGCTGTATTTTGCA
CCTGATCTAATACTAAATGAACAGCGGATGAAAGAATCATCATTCTATTCATTATGCCTTACCATGTGGCAGATCCC
ACAGGAGTTTGTCAAGCTTCAAGTTAGCCAAGAAGAGTTCCTCTGTATGAAAGTATTGTTACTTCTTAATACAATTC
CTTTGGAAGGGCTACGAAGTCAAACCCAGTTTGAGGAGATGAGGTCAAGCTACATTAGAGAGCTCATCAAGGCAA
TTGGTTTGAGGCAAAAAGGAGTTGTGTCGAGCTCACAGCGTTTCTATCAACTTACAAAACTTCTTGATAACTTGCAT
GATCTTGTCAAACAACTTCATCTGTACTGCTTGAATACATTTATCCAGTCCCGGGCACTGAGTGTTGAATTTCCAGA
AATGATGTCTGAAGTTATTGCTGGGTCGACGAGATATCAAGCAGAATTCCAGTACCTGCCAGATACAGACGATCG
TCACCGGATTGAGGAGAAACGTAAAAGGACATATGAGACCTTCAAGAGCATCATGAAGAAGAGTCCTTTCAGCGG
ACCCACCGACCCCCGGCCTCCACCTCGACGCATTGCTGTGCCTTCCCGCAGCTCAGCTTCTGTCCCCAAGCCAGCAC
CCCAGCCCTATCCCTTTACGTCATCCCTGAGCACCATCAACTATGATGAGTTTCCCACCATGGTGTTTCCTTCTGGGC
AGATCAGCCAGGCCTCGGCCTTGGCCCCGGCCCCTCCCCAAGTCCTGCCCCAGGCTCCAGCCCCTGCCCCTGCTCC

AGCCATGGTATCAGCTCTGGCCCAGGCCCCAGCCCCTGTCCCAGTCCTAGCCCCAGGCCCTCCTCAGGCTGTGGCC
CCACCTGCCCCCAAGCCCACCCAGGCTGGGGAAGGAACGCTGTCAGAGGCCCTGCTGCAGCTGCAGTTTGATGAT
GAAGACCTGGGGGCCTTGCTTGGCAACAGCACAGACCCAGCTGTGTTCACAGACCTGGCATCCGTCGACAACTCC
GAGTTTCAGCAGCTGCTGAACCAGGGCATACCTGTGGCCCCCCACACAACTGAGCCCATGCTGATGGAGTACCCT
GAGGCTATAACTCGCCTAGTGACAGGGGCCCAGAGGCCCCCCGACCCAGCTCCTGCTCCACTGGGGGCCCCGGG
GCTCCCCAATGGCCTCCTTTCAGGAGATGAAGACTTCTCCTCCATTGCGGACATGGACTTCTCAGCCCTGCTGAGTC
AGATCAGCTCCTAATctagcacctaggaggatccactcgAGGTTTAGAGAGGGCGAATCCAGCTCTGGAGCAGAGGCTCT
GGCAGCTTTTGCAGCGTTTATATAACATGAAATATATATACGCATTCCGATCAAAGCTGGGTTAACCAGATAGATA
GATAGTAACGTTTAAATAGCGCCTGGCGCGTTCGATTTTAAAGAGATTTAGAGCGTTATCCCGTGCCTATAGATCa
TATAGTATAGACAACGAACGATCACTCAAATCCAAGTCAATAATTCAAGAATTTATGTCTGTTTCTGTGAAAGGGA
AACTAATTTTGTTAAAGAAGACTTACAATATCGTAATACTTGTTCAATCGTCGTGGCCGATAGAAATATCTTACAAT
CCGAAAGTTGATGAATGGAATTGGTCTGCAACTGGTCGCCTTCATTTCGTAAAATGTTCGCTTGCGGCCGAAAAAT
TTCGATATATCTACAATTcATCTACAATCTTTACTAAATTTTGAAAAAGGAACACTTTGAATTTCGAACTGTCAATCG
TATCATTAGAATTTAATCTAAATTTAAATCTTGCTAAAGGAAATAGCAAGGAACACTTTCGTCGTCGGCTACGCATT
CATTGTAAAATTTTAAATTTTGACATTCCGCACTTTTTGATAGATAAGCGAAGAGTATTTTTATTACATGTATCGCAA
GTATTCATTTCAACACACATATCTATATATATATATATATATATATATATATATATATATATATATGTTATATATTTAT
TCAATTTTGTTTACCATTGATCAATTTTTCACACATGAAACAACCGCCAGCATTATATAATTTTTTTATTTTTTTAAAA
AATGTGTACACATATTCTGAAAATGAAAAATTCAATGGCTCGAcTGCCAAATAAAGAAATGGTTACAATTTAAGGA
AACAAATGTCCTTCTTGCACTAGGGGGGTGACATCCCTAGGTCCGGAGAATgtttaaacaGAAGTTCCTATTCCGAAG
TTCCTATTCTCTAGAAAGTATAGGAACTTCtcgtagtgccccaactggggtaacctttgagttctctcagttgggggcgtagCGGTCTC
GAAGCCGCGGTGCGGGTGCCAGGGCGTGCCCTTGGGCTCCCCGGGCGCGTACTCCACCTCACCCATCTGGTCCAc
ctgcaggGACGTCAGGTGGCACTTTTCGGGGAAATGTGCGCGGAACCCCTATTTGTTTATTTTTCTAAATACATTCAA
ATATGTATCCGCTCATGAGACAATAACCCTGATAAATGCTTCAATAATATTGAAAAAGGAAGAGTATGAGTATTCA
ACATTTCCGTGTCGCCCTTATTCCCTTTTTTGCGGCATTTTGCCTTCCTGTTTTTGCTCACCCAGAAACGCTGGTGAA
AGTAAAAGATGCTGAAGATCAGTTGGGTGCACGAGTGGGTTACATCGAACTGGATCTCAACAGCGGTAAGATCCT
TGAGAGTTTTCGCCCCGAAGAACGTTTTCCAATGATGAGCACTTTTAAAGTTCTGCTATGTGGCGCGGTATTATCCC
GTATTGACGCCGGGCAAGAGCAACTCGGTCGCCGCATACACTATTCTCAGAATGACTTGGTTGAGTACTCACCAGT
CACAGAAAAGCATCTTACGGATGGCATGACAGTAAGAGAATTATGCAGTGCTGCCATAACCATGAGTGATAACAC
TGCGGCCAACTTACTTCTGACAACGATCGGAGGACCGAAGGAGCTAACCGCTTTTTTGCACAACATGGGGGATCA
TGTAACTCGCCTTGATCGTTGGGAACCGGAGCTGAATGAAGCCATACCAAACGACGAGCGTGACACCACGATGCC
TGTAGCAATGGCAACAACGTTGCGCAAACTATTAACTGGCGAACTACTTACTCTAGCTTCCCGGCAACAATTAATA
GACTGGATGGAGGCGGATAAAGTTGCAGGACCACTTCTGCGCTCGGCCCTTCCGGCTGGCTGGTTTATTGCTGAT
AAATCTGGAGCCGGTGAGCGTGGGTCTCGCGGTATCATTGCAGCACTGGGGCCAGATGGTAAGCCCTCCCGTATC
GTAGTTATCTACACGACGGGGAGTCAGGCAACTATGGATGAACGAAATAGACAGATCGCTGAGATAGGTGCCTCA
CTGATTAAGCATTGGTAACTGTCAGACCAAGTTTACTCATATATACTTTAGATTGATTTAAAACTTCATTTTTAATTT
AAAAGGATCTAGGTGAAGATCCTTTTTGATAATCTCATGACCAAAATCCCTTAACGTGAGTTTTCGTTCCACTGAGC
GTCAGACCCCGTAGAAAAGATCAAAGGATCTTCTTGAGATCCTTTTTTTCTGCGCGTAATCTGCTGCTTGCAAACAA
AAAAACCACCGCTACCAGCGGTGGTTTGTTTGCCGGATCAAGAGCTACCAACTCTTTTTCCGAAGGTAACTGGCTT
CAGCAGAGCGCAGATACCAAATACTGTTCTTCTAGTGTAGCCGTAGTTAGGCCACCACTTCAAGAACTCTGTAGCA
CCGCCTACATACCTCGCTCTGCTAATCCTGTTACCAGTGGCTGCTGCCAGTGGCGATAAGTCGTGTCTTACCGGGTT
GGACTCAAGACGATAGTTACCGGATAAGGCGCAGCGGTCGGGCTGAACGGGGGGTTCGTGCACACAGCCCAGCT
TGGAGCGAACGACCTACACCGAACTGAGATACCTACAGCGTGAGCTATGAGAAAGCGCCACGCTTCCCGAAGGG
AGAAAGGCGGACAGGTATCCGGTAAGCGGCAGGGTCGGAACAGGAGAGCGCACGAGGGAGCTTCCAGGGGGA
AACGCCTGGTATCTTTATAGTCCTGTCGGGTTTCGCCACCTCTGACTTGAGCGTCGATTTTTGTGATGCTCGTCAGG

GGGGCGGAGCCTATGGAAAAACGCCAGCAACGCGGCCTTTTTACGGTTCCTGGCCTTTTGCTGGCCTTTTGCTCAC
ATGTTCTTTCCGATATCATTGG

Click here to access/download
Supporting Information
msfile-1.pdf

Data_S1

Click here to access/download
Supporting Information
supp-1.xlsx
