# Supplementary material for: Repeat mediated excision of gene drive elements for restoring wild-type populations
Source: PLoS Genet. 2024 Nov 7;20(11):e1011450. doi: 10.1371/journal.pgen.1011450 (PMC11584131; doi:10.1371/journal.pgen.1011450)
Supplement: Attachment — Submitted filename: Response_to_Reviewers.pdf [file pgen.1011450.s015.pdf]

Dear Dr Myles,

Thank you very much for submitting your Research Article entitled 'Repeat mediated excision of gene drive elements for restoring wild-type populations' to PLOS Genetics.

The manuscript was fully evaluated at the editorial level and by independent peer reviewers. The reviewers appreciated the attention to an important topic but identified some concerns that we ask you address in a revised manuscript.

We therefore ask you to modify the manuscript according to the review recommendations. Your revisions should address the specific points made by each reviewer.

We wish to express our sincere gratitude to the editors and reviewers for their thoughtful and constructive feedback. We greatly appreciate the time and effort they have invested in reviewing our manuscript. In response, we have made substantial revisions and addressed the reviewers' suggestions wherever possible. For comments we were unable to fully incorporate or address, we have provided explanations. Importantly, where we could not provide clear experimental answers, we modified the text to acknowledge alternative possibilities and interpretations of the data. Below, you will find a point-by-point response with our replies presented in italicized text. We have also included a marked up version of the manuscript with all changes from the previous version tracked.

In addition we ask that you:
1) Provide a detailed list of your responses to the review comments and a description of the changes you have made in the manuscript.
2) Upload a Striking Image with a corresponding caption to accompany your manuscript if one is available (either a new image or an existing one from within your manuscript). If this image is judged to be suitable, it may be featured on our website. Images should ideally be high resolution, eye-catching, single panel square images. For examples, please browse our archive. If your image is from someone other than yourself, please ensure that the artist has read and agreed to the terms and conditions of the Creative Commons Attribution License. Note: we cannot publish copyrighted images.

We hope to receive your revised manuscript within the next 30 days. If you anticipate any delay in its return, we would ask you to let us know the expected resubmission date by email to plosgenetics@plos.org.

If present, accompanying reviewer attachments should be included with this email; please notify the journal office if any appear to be missing. They will also be available for download from the link below. You can use this link to log into the system when you are ready to submit a revised version, having first consulted our Submission Checklist.

While revising your submission, please upload your figure files to the Preflight Analysis and Conversion Engine (PACE) digital diagnostic tool. PACE helps ensure that figures meet PLOS requirements. To use PACE, you must first register as a user. Then, login and navigate to the UPLOAD tab, where you will find detailed instructions on how to use the tool. If you encounter any issues or have any questions when using PACE, please email us at figures@plos.org.

Please be aware that our data availability policy requires that all numerical data underlying graphs or summary statistics are included with the submission, and you will need to provide this upon resubmission if not already present. In addition, we do not permit the inclusion of phrases such as "data not shown" or "unpublished results" in manuscripts. All points should be backed up by data provided with the submission.
To enhance the reproducibility of your results, we recommend that you deposit your laboratory protocols in protocols.io, where a protocol can be assigned its own identifier (DOI) such that it can be cited independently in the future. Additionally, PLOS ONE offers an option to publish peer-reviewed clinical study protocols. Read more information on sharing protocols at https://plos.org/protocols?utm_medium=editorial-email&utm_source=authorletters&utm_campaign=protocols

Please review your reference list to ensure that it is complete and correct. If you have cited papers that have been retracted, please include the rationale for doing so in the manuscript text, or remove these references and replace them with relevant current references. Any changes to the reference list should be mentioned in the rebuttal letter that accompanies your revised manuscript. If you need to cite a retracted article, indicate the article's retracted status in the References list and also include a citation and full reference for the retraction notice.

PLOS has incorporated Similarity Check, powered by iThenticate, into its journal-wide submission system in order to screen submitted content for originality before publication. Each PLOS journal undertakes screening on a proportion of submitted articles. You will be contacted if needed following the screening process.

To resubmit, you will need to go to the link below and 'Revise Submission' in the 'Submissions Needing Revision' folder.

Please let us know if you have any questions while making these revisions.

Yours sincerely,

Jackson Champer
Academic Editor
PLOS Genetics

Gregory Copenhaver
Section Editor
PLOS Genetics

The authors have fixed most of the issues, and the manuscript is not likely to go out for review again. However, some issues remain from reviewer 3, which are hopefully clearer in the new review. This reviewer presents some options that the authors could use to really thoroughly investigate their constructs. They should seriously consider these, but perhaps the authors could use their existing constructs to address at least some of this, which will likely be enough for the publication.

This could help determine if the wild-type alleles (which should be labeled more clearly as such in the figures, and perhaps be added as an outcome to Figure 1C because they are much more common) actually come from the ReMEDE element or if the ReMEDE just has lower cut rates compare to the original yMCR. I'd lean toward the latter as the more likely explanation, with the gypsy elements perhaps reducing Cas9 expression, or the ReMEDE removing the drive before it has the chance to express enough Cas9 to cut all the wild-type alleles. These possibilities will need to be clearly laid out if there is not a clear experimental answer.

As noted in reviewer 3's point #1, the experiment would involve using males, ideally separate experiments with both constructs. Based on the promoters, it looks like everything would express quite well in males (both vasa and nanos have high male expression, nanos might even be higher in males than females in many Cas9 constructs). Thus, you could just cross ReMEDE males to wild-type or yellow (from resistance alleles) females and check the outcomes. With no drive-induced cleavage or wild-type template to track, you could be more certain of SSA outcomes, which could address some of what reviewer 3 is concerned about. Using ReMEDE homozygous females could also work well for this purpose, especially if there was a problem with this system in males for unclear reasons.

We have incorporated the results of experiments involving gene drive male crosses into the revised manuscript (Fig. S4A). However, we did not observe any SSA outcomes in these crosses. We hypothesize that this may be due to transcriptional silencing of the X chromosome in the germline of heterogametic males, where transgene expression on the X chromosome is suppressed relative to autosomes. We have added a discussion of this phenomenon, supported by appropriate references.

In these crosses, all female offspring inherited the gene drive transgene and expressed fluorescent markers (Fig. S4A). However, yellow body pigmentation, an X-linked recessive trait, could only appear in these females if Cas9 from the paternal transgene was active. Since there was no maternal Cas9 source, any loss of function in the maternally inherited yellow allele must have occurred through Cas9 expressed from the paternally inherited gene drive. If the yReMEDE drive were producing lower levels of Cas9 to an extent that would leave some alleles uncut, we would expect to observe this, but it was not seen. Instead, as anticipated, 100% of female progeny from both yMCR and yReMEDE crosses exhibited yellow pigmentation, suggesting approximately equivalent Cas9 activity from both elements (Fig. S4A). Furthermore, this is consistent with the results of crosses involving gene drive females. Crosses of F1 yMCR females with wild-type males produced indels in 1–2% of inherited alleles (Fig. 2D), and similar frequencies (~3%) were found in crosses with F1 yReMEDE females (Fig. 2E). Again, if the yReMEDE drive were producing significantly lower levels of Cas9, we would not expect to see similar rates of indel formation.

To assess SSA in the absence of drive-induced cleavage or leaky in-cis expression of I-SceI, we performed crosses with a yReMEDE transgene containing mutations in the vasa promoter that render Cas9 non-functional (Fig. S4B). Similarly, the nos-GS element driving I-SceI expression was inactivated by replacing the UAS sequence upstream of Hsp70 with UASt. Unlike UASp in the active yReMEDE gene drive element, UASt is repressed in the female germline of Drosophila melanogaster. Homozygous females or hemizygous males for the transgene were outcrossed to either wild-type flies or a transgenic line expressing I-SceI from the Hsp70 promoter. I-SceI expression was induced by subjecting embryos to heat shock (38 °C for 1 hour). Fluorescent markers in yReMEDE were used to assess transgene inheritance. Mosaic F1 individuals were outcrossed to wild-type flies, and germline events were identified in F2 progeny by fluorescence. Crossing schemes for transgenic males and females are shown in Fig. S4C and D. As expected, control crosses with wild-type flies resulted in Mendelian inheritance of the transgene in F2 progeny (Fig. S4C). However, outcrosses with the I-SceI line (experimental test) showed a significant reduction in F2 progeny with fluorescent markers, indicating SSA-mediated loss of the transgene (Fig. S4C and Data S1). The percentage of SSA events, categorized by sex, is shown in Fig. S4E and Data S1. Sequencing of randomly selected families from female experimental outcrosses confirmed that some alleles contained the engineered TcG sequence but were otherwise wild type, suggesting SSA events (Fig. S4F and Data S1). Similarly, sequencing from a male experimental outcross showed that all alleles in non-fluorescent progeny contained the TcG sequence, further suggesting SSA-mediated inheritance (Fig. S4F and Data S1).

We believe the comment above may have intended to refer to figure 2C rather than figure 1C. The original figure 2C showed expected outcomes. As the wild-type alleles were not an expected outcome they were not depicted in the figure. However, we have modified the figure and hopefully it is more clear now.

For the comments of reviewer 2 and the other comments of reviewer 3, changes to the text for clarity and emphasis should be sufficient. For aspects of reviewer 3's comments on experiments and uncertainty, it will be important to address it as much as possible, probably including the above suggested experiment.

We have addressed the other comments of reviewer 2 and 3 below.

Finally, (for authors and reviewers) in the manuscript pdf, the figure resolutions were indeed very low. However, this was because the authors submitted separate figure files that were then converted by the online system to be part of the pdf, reducing the resolution in the process. To rectify this, you need to click on the "tif" file download in the upper right corner of the figure pages. These will then download the higher resolution figures, which are legible. However, I should note that while they are legible, it may still not meet the journal standards, so consider uploading higher resolution versions as part of the revisions, or else you might be asked to do it again anyway later in the publication process.

Sincerely,
Jackson Champer

Reviewer's Responses to Questions
Comments to the Authors:
Please note here if the review is uploaded as an attachment.
Reviewer #1: The authors have addressed all my comments comprehensively, and the manuscript has been improved in current version with necessary revisions and extra sequencing data, which enhanced the accuracy and clarity of their work. Therefore, I recommend to accept this manuscript for publication in PLOS Genetics.

Reviewer #2: Thank you for your responses. The idea of using SSA to construct a self-extinguishing gene drive is interesting and this paper serves as a good proof of principle. I only have one minor comments now: regarding the sequencing result in Fig.3A, it's good to see evidence of resistance forming over time but only at later stage, however I found it hard to understand the actual frequency of this 17bp deletion. The caption said it's the representative sequences and on figure 3A the 'samples' stat for this sequence is 3. Does that mean you found three individuals bearing the same 17bp deletion? If yes, how many samples did you sequenced to find these 3? Would be good to clarify this.

Thank you for pointing this out. The most recent samples represent 3 different mosquito pools, each consisting of 15 males and 15 females, that were sequenced by nanopore. The older samples represent individuals sequenced with sanger methods. We have now clarified this in the figure legend.

Reviewer #3: Review uploaded as an attachment

Reviewer #4: The authors have revised the manuscript sufficiently. I appreciate that the authors took the time to carefully address my all comments. This revised version can be accepted for publication. however, the authors should provide a better quality images in the final version of the manuscript. Images/pictures in the present version are not of great quality.

Reviewer #5: The authors had an extensive set of reviews, a common thrust of which was to improve the clarity of the exposition of the results, to do the work justice. Looking at the responses to reviewers I am happy with the clarifications. I must admit, I have not had the time to re-read the whole article and compare the before and after, so I am taking the authors on their word that they have incorporated the necessary changes.
The low resolution of the figures in the resubmitted PDF precluded me from looking at them.

Have all data underlying the figures and results presented in the manuscript been provided?
Large-scale datasets should be made available via a public repository as described in the *PLOS Genetics* data availability policy, and numerical data that underlies graphs or summary statistics should be provided in spreadsheet form as supporting information.
Reviewer #1: Yes
Reviewer #2: Yes
Reviewer #3: None
Reviewer #4: Yes
Reviewer #5: Yes

PLOS authors have the option to publish the peer review history of their article (what does this mean?). If published, this will include your full peer review and any attached files.

If you choose "no", your identity will remain anonymous but your review may still be made public.

Do you want your identity to be public for this peer review? For information about this choice, including consent withdrawal, please see our Privacy Policy.
Reviewer #1: No
Reviewer #2: No
Reviewer #3: No
Reviewer #4: No
Reviewer #5: No

The revised manuscript by Chennuri et al. is more clearly written and discusses several issues regarding data interpretation that are improvements in the presentation of their results. Nonetheless, I still have questions regarding some important aspects of the experimental results which weaken the impact of the manuscript in its current form.

In reviewing the authors responses to my prior comments, it seems that our various intended points may have crossed in certain respects, which could in part be due to my not having been sufficiently clear. I will try to restate my key concerns here again, hopefully, more effectively.

We thank the reviewer for the time and effort they clearly expended in reviewing and thinking about our manuscript. Clearly, they were very interested in the topic of our manuscript, and the many questions that the results have raised about DNA repair pathways in Drosophila and other organisms.

1) I am still quite puzzled by the low levels of SSA-mediated drive deletion reported do not increase upon induction of the I-SceI gene either in the yReMet-cis configuration. The authors suggest that this might be due to basal levels of nuclease activity already being saturating such that further induction yields no higher rates of SSA. This is odd though given how low those rates are (also those rates are even lower for the 500 bp repeat than for the 250 bp version, which also does not make sense) and is very different than the rates they observe for their transacting system for inducing I-SceI using the GAL4/UAS system, which does steadily increase in efficiency as the repeat length gets longer (Fig. 1B). Similarly, efficient SSA-mediated deletion rates were reported in the pre-print by Aguilar and colleagues (reference included in revised list of recommended SSA papers to cite below). The very marginal activity of the yReMet-cis element makes it hard to understand the results for the yReMEDE, which as I understand it relies on the same genetic cassette for I-SceI expression and yet according to their analysis in Fig. 2C seems to generate a substantially higher rate of SSA events? It is also noteworthy that in the paper by Chae et al, 2022 by the same authors examining SSA in mosquitoes using a similar scheme, SSA rates using the nos:I-SceI system were also quite low. If one supposed that in the current

study, I-SceI expression was no different in yReMet-cis and yReMEDE, then could Cas9 expression by the primary mechanism behind the generation of the excess of y+ in yReMEDE/+ heterozygotes?

We are similarly puzzled by the differences in apparent SSA rates between the gene drive and the yReMET-cis transgene. We believe this variation is somehow linked to the gene drive process itself. Interestingly, in some crosses involving flies with the yReMET-cis transgene, SSA rates were actually much higher than those observed with the gene drive, based on measurements using the engineered marker allele (Fig. 1C). Thus, rates of SSA varied considerably among these crosses. Therefore, it also seems possible that in the presence of gene drive, SSA simply becomes more consistent resulting in the higher average rate? If so, then determining the actual mechanism behind this effect could prove to be very difficult.

One experiment that should shed light on this question would be to measure the rate of generating y+ progeny when the yReMEDE drive element is homozygous as compared to heterozygous in females since Cas9 would not be doing any cutting when homozygous. Similarly, analyzing the basal level of generating y+ progeny from yReMEDE males mated to an attached-X stock should be informative? Would they see comparable levels of SSA as in Fig. 2C (yReMet/WT heterozygotes)? Comparing yReMEDE males (with no paternal Cas9 deposition) to homozyous yReMEDE females (where maternal Cas9 deposition does take place) would provide an indication for whether Cas9 in the absence of target cleavage could somehow increase SSA (unlikely, but worth testing to be sure). Perhaps the authors have already done these experiments?

The most direct approach would be to generate a version of yReMEDE either without I-SceI (to unambiguously test for its requirement in SSA) or place I-SceI expression under GAL4 control as in the yReMet trans element. Then the authors could cross this element to the nos-GAL4 driver and see whether it undergoes the same efficient SSA deletion as seen with yReMet. Another informative experiment would be to place the yReMEDE-UAS element in trans to the yReMet-cis element in the presence of nos-GAL4. This experiment should be possible to do technically given the very low level of SSA observed with the yReMet-cis element by first combining yReMet-cis with nos-GAL4 and then cross their progeny to yReMEDE-UAS. By differentially marking the yReMet-cis and yReMEDE chromosomes (e.g., with either a viable achaete-scute allele or white-) the authors should be able to compare the relative frequency of SSA deletion in these two elements, which would resolve whether the low frequency for the yReMet-cis element has to do with its internal structure or its ability to induce I-SceI expression.

It also should be revealing to simply redo the experiment in Fig. 2B using differentially marked chromosomes carrying the yReMEDE versus WT alleles (again either the very closely linked achaete-scute or slightly more distant white- locus could serve as such markers). This type of analysis would additionally shed light on the question raised in point 3 below regarding the possibility that the yReMEDE system combined with Cas9 drive could induce chromosome damage or loss. Furthermore, this analysis would allow the authors to determine whether the dual action of Cas9 and I-SceI can increase recombination between the two chromosome homologs, which could offer important mechanistic clues to resolving the issue in point 2.

We have included in the revision the results of the experiments involving gene drive male crosses (Fig. S4A). However, we did not see any SSA outcomes in these crosses. We postulate that this may be due to transcriptional silencing of the X chromosome in the germline of heterogametic males, where transgene expression on the X chromosome is suppressed relative to autosomes. We have included in the discussion, a description of this phenomenon with the appropriate references for support.

In order to assess SSA in the absence of drive-induced cleavage or leaky in-cis expression of I-SceI, we performed crosses with a yReMEDE transgene (Fig. S4B) containing mutations in the vasa promoter that render Cas9 non-functional (Fig. S4B). Similarly, the nos-GS element driving I-SceI expression was inactivated by replacing the UAS sequence upstream of Hsp70 with UASt. Unlike UASp (p = P transposase promoter) in the active yReMEDE gene drive element, UASt is repressed in the female germline of Drosophila melanogaster. Homozygous females or hemizygous males for the transgene were outcrossed to either wild-type flies or a transgenic line expressing I-SceI from the Hsp70 promoter. I-SceI expression was induced by subjecting embryos to heat shock (38 °C for 1 hour). Fluorescent markers in yReMEDE were used to assess transgene inheritance. Mosaic F1 individuals were outcrossed to wild-type flies, and germline events were identified in F2 progeny by fluorescence. Crossing schemes for transgenic males and females are shown in Fig. S4C and D. As expected, control crosses with wild-type flies resulted in Mendelian inheritance of the transgene in F2 progeny (Fig. S4C). However, outcrosses with the I-SceI line (experimental test) showed a significant reduction in F2 progeny with fluorescent markers, indicating SSA-mediated loss of the transgene (Fig. S4C and Data S1). The percentage of SSA events, categorized by sex, is shown in figure S4E and data S1. Sequencing of randomly selected families from female experimental outcrosses confirmed that some alleles contained the engineered TcG

sequence but were otherwise wild type, suggesting SSA events (Fig. S4F and Data S1). Similarly, sequencing from a male experimental outcross showed that all alleles in non-fluorescent progeny contained the TcG sequence, further suggesting SSA-mediated inheritance (Fig. S4F and Data S1).

2) Curiously, the reported SSA-mediated deletion events often do not carry the expected cis-marked allelic variant (this contrasts with what they observe for the yReMet element either in cis- or trans configurations). The basis for this finding is not resolved? The authors discuss a variety of possible such events might be occurring (including a need for Cas9 cleavage), but they do not perform experiments to analyze the basis for this phenomenon, which most likely holds a key to how such dual nuclease systems work.
Including additional polymorphisms at different distances and both sides of the repeats (up to 10 kb away) also would be an informative way to help figure this process out. Similarly, as mentioned above, they could use a tightly linked visible marker (e.g., viable alleles of the achaete-scute complex, which is the gene immediately next to yellow) to help distinguish local gene-conversion from cross-over events. These types of studies should provide the type of data to conclusively determine the origin and requirements for various aspects of the drive process

Alternatively, since trans-acting GAL4/UAS system generate decent levels of SSA-mediated deletion, the authors might consider constructing a similar split version the yReMEDE system (with I-SceI expression under GAL4 control as suggested above) to see if they obtain different results than with the cis-acting system. In particular, what would be the fraction of yellow+ alleles carrying the TCG allele when the yReMEDE allele was homozygous versus in trans to a wt allele or a cleavage resistant allele (different from TCG)? In these experiments, it would be important to used well marked chromosomes distinguishing the one carrying the drive cassette and the target allele. Another possible approach would be to generate a females with a trans configuration of yReMet ac+ w-/yReMEDE ac- w+; nos-GAL4 to determine the relative efficiency of SSA in these two vector contexts. Such a dual marked system would also permit an assessment of whether induction of SSA events lead to greater rates of recombination between achaete and white.

We are confused by the reviewer's assertion that "the reported SSA-mediated deletion events often do not carry the expected cis-marked allelic variant (this contrasts with what they observe for the yReMet element either in cis- or trans configurations)". In the experiments with yReMET we observed either the transgene, engineered TcG marker, or wild-type sequences depending of course on the cross. Observation of the gGG sequence in the yReMEDE experiments was exceedingly rare. Thus, we primarily observed either the transgene, engineered TcG marker or wild-type sequences in the yReMEDE experiments as well, which closely aligns with our observations in the yReMET experiments.

Please also see above.

3) The question of whether the dual nuclease system in yReMEDE results in genome damage while the drive casette is copying to a wt target, which could result in a potential reduction in the fraction of chromosomes carrying the drive construct, remains unresolved (the authors seem to address my previous question by referring to transmission through males, which is not the question I had in mind). The phenomenon, if it occurs, would be evident in progeny of females carrying one drive allele and a differentially marked WT allele (carrying an adjacent visible achaete-scute allele). The question would be whether the fraction of male progeny carrying the drive chromosome equaled that carrying the WT allele? If not, then some of the alleles present in females could be homozygous lethals (or hemizygous lethals in males). Such alleles could be recovered over balancers if this result was observed and further analyzed to assess their origin and nature. This type of analysis might also benefit from using a split yReMEDE system as suggested above since there would be no prior I-SceI cleavage prior to making the cross.

Thank you for the clarification, we agree that this is something that we have not ruled out. As such, we have included this possibility in our discussion of possible reasons that we see the unexpected wild type alleles.

4) The authors argue that they cannot use the current system to adequately test their system in multigenerational cage trials primarily because of the strong competitive advantage that yellow+ males have relative to less fit yellow- males. There are several relatively simple solutions to this issue, which after all, is crucially central to testing their concept. One simple solution is to associate both test alleles with a second well separated yellow mutant allele (e.g., near 5' end of the gene). This could easily be accomplished by crossing the drive element to chromosome carrying the 5' y-allele and recovering progeny in which the element had copied to that chromosome. Then all competing individuals would be competitively equal. Alternatively, they could add recoded 3' yellow sequences to drive element to endow it with a yellow+ phenotype.

Based on modeling results, the authors suggest that their system even with its relatively low level of basal SSA-mediated cassette deletion could lead to eventual population replacement with a cassette-deleted allele. While this may true, there are several issues that still need to be considered including how would they maintain such an unstable strain in the lab. It obviously could not be kept long-term as a pure homozygous stock. The authors should also compare such scenarios to much simpler drive systems such as those designed to accomplish population suppression. Many of these systems generate low levels of functional NHEJ alleles. Such functional cleavage resistant target alleles also can take over populations as has been demonstrate experimentally by several groups. One could even use a very efficient suppression drive such as the Doublesex drive from the Crisanti group and then introduce a low fraction of individuals carrying a recoded functional target site as many commentators have suggested as an alternative which perhaps might generate less stochastic outcomes. What are the advantages that the authors see would SSA-mediated cassette deletion over just letting the drive cassette get eliminated by competition with a wild-type allele.

We acknowledge the reviewer's valid concern, which is one of the reasons we explored the inducible system. However, due to the leakiness of this system and others we have tested, this remains a significant challenge. We believe that one of the valuable contributions of our work is identifying the issues that need to be resolved or overcome to make this configuration effective. While there may not be advantages in the scenario outlined above, to the best of our knowledge, no one had previously considered an alternative based on the SSA mechanism prior to our study. The SSA mechanism could offer advantages if methods are developed to allow more precise control over the timing of gene drive removal. If this is not feasible, the technology may be better suited for other applications, such as an in trans braking system.

For all the reasons summarized above, hopefully better articulated this round, I suggest that the authors consider doing a few additional incisive experiments to resolve what I believe are key unresolved questions. This additional analysis, much of which could be done with existing stocks, would strengthen the study significantly and provide a better data set for evaluating the potential virtues and limitations of their very worthy conceptual idea.

We would like to once again thank the reviewer for their invaluable suggestions. While the yellow gene drive was beneficial for initially demonstrating the concept, it is only one example of gene drive technology and has numerous limitations (e.g., resistance allele formation, mating cost, sex linkage). Therefore, our preference is to continue work on this system in the context of other drives, which we are currently pursuing. The reviewer's insights have already proven extremely helpful in guiding this research. We are eager to see how many of the results observed with the yellow drive will be similar in other drives, as this will prove informative in determining which of these are simply specific to the ReMEDE yellow drive, or are actually useful insights into the fundamental biology of DNA repair pathways and gene drive systems, which would then merit more detailed studies.

5) Discussion and citations: There are several points deserving more consideration in the discussion section. Most notably, the authors should include a thorough comparison of the results from the current study in Drosophila with the extensive experimental findings already published by the same group in *Aedes aegypti* mosquitoes (Chae et al., 2022). While the results in both systems are similar in some respects (low levels of SSA), they also are different in others (for example in mosquitoes NHEJ alleles at the I-SceI site cause loss of DsRed function but not adjacent GFP expression thus distinguishing NHEJ from SSA alleles, which is not considered in the current study). The way the authors currently cite that prior work makes it seem as though the Chae study is only a computational paper. It would be helpful to synthesize all these results to provide readers with a sense of how ReMEDE technology might progress in the future and what issues need to be considered for developing the technology to a field applicable state.

To the best of our knowledge, there is no prior work examining the removal of a gene drive other than the purely computational paper. The other prior studies focus on SSA in the context of removing a transgene, or as a method for creating knock-ins. Additionally, some of the SSA work had not yet been published at the time of our initial submission. Nevertheless, we have revised our statements regarding the prior computational work to more clearly articulate what we meant to say, and in accordance with your suggestions have cited the recommended papers.

Another odd result in this study mentioned by the authors is the apparent instability of the I-SceI target site. Since that sequence contains a 4 bp directly repeated sequence including 3Gs is this event potentially being mediated by a low frequency of SSA or perhaps MMEJ. If this this a problem intrinsic to I-SceI, which would render cassettes carrying such target site deletions refractory to I-SceI mediated SSA, might some other orthogonal nuclease be used instead?

Thank you, this is another excellent suggestion. We believe there may very well be differences depending on the nuclease used to make the DSB, and MMEJ may have much to do with this. However, given the short sequences required for MMEJ it may be difficult to identify any target site that doesn't have some micro homology.

Finally, regarding my prior list of suggested reference the authors should cite to ensure that adequate credit is assigned to prior work on exploiting SSA in Drosophila, I note that the authors are indeed correct in pointing out that I had selected the wrong citations for them to consider in this regard. My apologies on that mistake. Perhaps the authors could cite the three references indicated below, all of which have SSA in their titles. These references could all be cited in the introduction as antecedent work, and in the case of the latter two papers, also could be cited with regard to testing the dependence on the length of the flanking repeats for efficiency of SSA-mediated deletion.

We have cited, thank you.
