# Supplementary material for: Repeat mediated excision of gene drive elements for restoring wild-type populations
Source: PLoS Genet. 2024 Nov 7;20(11):e1011450. doi: 10.1371/journal.pgen.1011450 (PMC11584131; doi:10.1371/journal.pgen.1011450)
Supplement: Attachment — Submitted filename: Review Comments_122823.docx [file pgen.1011450.s011.docx]

In this study, the authors demonstrated a precise self-elimination of engineered gene drive components to restore the wild population in *D. melanogaster*. A number of modern gene drives have been developed to curb human-disease vectors and agricultural pests, and have been found efficient at lab scale, but none of them tested in field yet due to a variety of concerns including safeguards or unintended impacts upon release. A few self-limiting homing drives have been designed and developed for a controlled spread of the drive elements. However, development of a seamless self-elimination enabled homing drives as developed by the authors here, would offer more nature safe drive options. In a separate study (Zapletal et al., 2021) using mathematical models, the authors have shown the potential of such self-eliminating autonomous gene-drives in restoring the wild population over the time, at a rate of 10% drive elimination events.

Here, firstly the authors tested a Repeat Mediated Excision of a Transgene (ReMET), which relies on DSB repair by SSA repair pathway. The transgene cassette, consist of direct repeats, removal was assessed in cis (all in one) and trans (split) configurations, using UAS/GAL4 approach. The authors noted a significant SSA repair events depending on the ‘direct repeat’ sizes. Similarly, the authors designed a full CRISPR/Cas9-based gene drive cassette, consists of flanking direct repeats (250 bp) and inducible nos-GeneSwitch (RU486 induced GAL4), placed in the exon-2 of the yellow gene and successfully restored a wild type of population. The authors named this technology ‘Repeat Mediated Excision of a Drive’ (ReMEDE). The authors smartly mutated the PAM site in the right homology arm/sequence of the drive construct to score the SSA generated wild type allele upon the drive cassette removal. Moreover, the cage experiment was performed to evaluate ReMEDE potential, and authors noted absolute drive removal within 10-14 generations.

I have a couple of major and minor comments that would need to be addressed before the paper would be acceptable for publication.

Major Comments:

1. It is interesting to note that the SSA repair was relatively high for ReMET (6%-35%) in *trans* where the I-Site site was not exposed to the endonuclease prior to the F_0_ crosses (Fig 1B) whereas it was just 0.5%-1% in *cis* configuration. Importantly, with *cis* ReMET the leaky expression of I-SceI can result in an I-Site resistance allele formation. I’d suggest assessing the I-Site sequence intactness in the strains/stock (*cis* F_0_). The I-Site DSB if repaired by error prone NHEJ can reduce the SSA repair in the *cis* self-crosses shown. The authors should also cross the *cis* ReMET with *yellow* mutants to assess the SSA for male and female germlines respectively. Do the authors find mosaic phenotype in yReMET (cis) like trans (Fig 1)?
2. It is not clear why did the authors decide to develop a self-elimination full drive (yReMEDE) using the inducible *nos*-GS system which showed such a little SSA and a leaky expression too. Surprisingly, here the authors noted a relatively high rate of SSA and large number of wild type flies’ generation with the yReMEDE crosses compared to yMCR drive, that’s great! were these crosses performed on RU486 or without it? should mention in the results or M&M section. Also, did the authors get the yellow F1 females for yMCR as well or it was only with the yReMEDE (Fig.2C)? do the authors think that the use of gypsy elements in ReMEDE construct (unlike yMCR) modulated Cas9/gRNA expression (more precise), and likely reduced the Cas9/RNP deposition so that paternal alleles remained un-cut for yReMEDE’s wild type offspring (F2)?
3. Importantly, in the final yReMEDE construct a number of repetitive sequences/elements are present, I fear that these repetitive elements are prone to breakdown/rearrangements during homing, and can be lost during the repair processes (Simoni A et al, 2014 Nucleic Acids Research, Yang E et al, 2022 G3, Oberhofer et al, 2018), did the authors perform any assessment on the construct stability? or noted partial repair events?

**Minor comments:**

1. M&M: The authors should provide more details on the plasmid’s design/construction and cloning procedures followed.
2. M&M: The total numbers of genetic crosses performed (pair or *en masse*) for ReMET and others should be mentioned. The progeny counts can be added to the supplementary info or results.
3. M&M: the authors should provide more details on the cage set up. Should include total cages set (control/test), insect release ratio, days allowed for the egg laying, cage dimensions, RU486 (conc.) used or not etc. The screening/phenotype, progeny counts can be added to the supplementary info.
4. Cage studies: Fig 3, even with the yMCR’s high release ratio, why it never surged above the initial releases and the population remained almost steady (WT:yMCR) until gen-8-9, later the wild population gained? Did the authors note resistant alleles’ accumulation and/or drive mating/fitness costs? The later population has all the wild type of body color flies or yellow/mutants? The authors should provide more details on the observations/results etc.
5. Also, with the yReMEDE, it seems like the SSA dominate over the homing and the drive alleles could not sustain in the population even at initial generations. did the authors assess the SSA (TcG) in the later population or the rapid elimination is due to the associated fitness costs (if any) of the yReMEDE strains and overall reduced homing? the authors should provide more discussion, since the SSA events noted with the pair crosses were low. Did the authors note I-Site resistance alleles accumulation too in the population?
6. A direct repeat sequence (DR 250 bp) is placed close to the right homology site to achieve SSA repair. Do the authors consider that it could impede the entire cassette’s homing being a potential left homology for the Cas9/DSB repair as well? If yes, can be discussed.
7. Also, the I-Site is positioned asymmetrically in the construct, won't be good to keep it a bit symmetrical or use I-Sites on both the ends? Probably, it’d shorten the DRs exposure time for SSA.
8. The authors should also add a comment on the ReMEDE stock/line maintenance in the lab.
9. Term ‘genetic drive’ can be replaced with ‘gene drive’ to be consistent throughout the main text.
