# Supplementary material for: Repeat mediated excision of gene drive elements for restoring wild-type populations
Source: PLoS Genet. 2024 Nov 7;20(11):e1011450. doi: 10.1371/journal.pgen.1011450 (PMC11584131; doi:10.1371/journal.pgen.1011450)
Supplement: Attachment — Submitted filename: Responses_to_Reviewers.docx [file pgen.1011450.s013.docx]

Dear Dr Myles,

Thank you very much for submitting your Research Article entitled 'Repeat mediated excision of gene drive elements for restoring wild-type populations' to PLOS Genetics.

The manuscript was fully evaluated at the editorial level and by independent peer reviewers. The reviewers appreciated the attention to an important problem, but raised some substantial concerns about the current manuscript. Based on the reviews, we will not be able to accept this version of the manuscript, but we would be willing to review a much-revised version. We cannot, of course, promise publication at that time.

Should you decide to revise the manuscript for further consideration here, your revisions should address the specific points made by each reviewer. We will also require a detailed list of your responses to the review comments and a description of the changes you have made in the manuscript.

If you decide to revise the manuscript for further consideration at PLOS Genetics, please aim to resubmit within the next 60 days, unless it will take extra time to address the concerns of the reviewers, in which case we would appreciate an expected resubmission date by email to [plosgenetics@plos.org](mailto:plosgenetics@plos.org).

If present, accompanying reviewer attachments are included with this email; please notify the journal office if any appear to be missing. They will also be available for download from the link below. You can use this link to log into the system when you are ready to submit a revised version, having first consulted our [Submission Checklist](https://urldefense.com/v3/__http:/journals.plos.org/plosgenetics/s/submit-now*loc-submission-checklist__;Iw!!KwNVnqRv!F5-yyXLXfGG9KYzEJpFvQMzkBsnjqfUZsAS2AHRsg34ybQqFJ4CddJcdYee_GFT5z3Pvkv_WB3uicbs$).

To enhance the reproducibility of your results, we recommend that you deposit your laboratory protocols in [protocols.io](http://protocols.io/), where a protocol can be assigned its own identifier (DOI) such that it can be cited independently in the future. Additionally, PLOS ONE offers an option to publish peer-reviewed clinical study protocols. Read more information on sharing protocols at [https://plos.org/protocols?utm_medium=editorial-email&utm_source=authorletters&utm_campaign=protocols](https://urldefense.com/v3/__https:/plos.org/protocols?utm_medium=editorial-email&utm_source=authorletters&utm_campaign=protocols__;!!KwNVnqRv!F5-yyXLXfGG9KYzEJpFvQMzkBsnjqfUZsAS2AHRsg34ybQqFJ4CddJcdYee_GFT5z3Pvkv_Wrbzo0Zs$)

Please be aware that our [data availability policy](https://urldefense.com/v3/__http:/journals.plos.org/plosgenetics/s/data-availability__;!!KwNVnqRv!F5-yyXLXfGG9KYzEJpFvQMzkBsnjqfUZsAS2AHRsg34ybQqFJ4CddJcdYee_GFT5z3Pvkv_W8HBmwHg$) requires that all numerical data underlying graphs or summary statistics are included with the submission, and you will need to provide this upon resubmission if not already present. In addition, we do not permit the inclusion of phrases such as "data not shown" or "unpublished results" in manuscripts. All points should be backed up by data provided with the submission.

While revising your submission, please upload your figure files to the [Preflight Analysis and Conversion Engine](https://urldefense.com/v3/__http:/pace.apexcovantage.com/__;!!KwNVnqRv!F5-yyXLXfGG9KYzEJpFvQMzkBsnjqfUZsAS2AHRsg34ybQqFJ4CddJcdYee_GFT5z3Pvkv_WCG-WEMU$) (PACE) digital diagnostic tool.  PACE helps ensure that figures meet PLOS requirements. To use PACE, you must first register as a user. Then, login and navigate to the UPLOAD tab, where you will find detailed instructions on how to use the tool. If you encounter any issues or have any questions when using PACE, please email us at [figures@plos.org](mailto:figures@plos.org).

PLOS has incorporated [Similarity Check](https://urldefense.com/v3/__http:/www.crossref.org/crosscheck.html__;!!KwNVnqRv!F5-yyXLXfGG9KYzEJpFvQMzkBsnjqfUZsAS2AHRsg34ybQqFJ4CddJcdYee_GFT5z3Pvkv_WBgAOW9U$), powered by iThenticate, into its journal-wide submission system in order to screen submitted content for originality before publication. Each PLOS journal undertakes screening on a proportion of submitted articles. You will be contacted if needed following the screening process.

To resubmit, use the link below and 'Revise Submission' in the 'Submissions Needing Revision' folder.


We are sorry that we cannot be more positive about your manuscript at this stage. Please do not hesitate to contact us if you have any concerns or questions.

Yours sincerely,

Jackson Champer
Academic Editor
PLOS Genetics

Gregory Copenhaver
Editor-in-Chief
PLOS Genetics

We have surprisingly received a large number of reviews for this manuscript despite the proximity of the winter holiday to the submission. I’d like to thank the reviewers for their rapid response during this time of year.

The topic and method of this study was interesting to myself and the reviewers. However, there are a large number of essential points detailed by the reviewers that I agree with and should be taken into account in a major revision before the paper can be published. Acceptance of the paper will ultimately be dependent on fully addressing all review comments, which in most cases can be by substantial changes and additions to the text.

While several points will require toning down efficiency claims in the manuscript, lower than ideal efficiency isn’t necessarily an obstacle to publication here. The fastest path to publication probably involves following most/all suggestions from the reviewers in these cases (you can always work to improve the efficiency in future studies).

There is some repetition between the reviewers (though each one also raises unique points), so in these cases, later responses in your “response to reviewers” document can be abbreviated, referring to previous responses.

Sincerely,
Jackson Champer

Reviewer's Responses to Questions

*We would like to thank the editors and reviewers for their comments, which were both substantive and constructive. We recognize that the reviewers’ time and effort are valuable, and thank them for their service in reviewing this manuscript. We have made significant revisions to the manuscript, whenever possible incorporating the suggestions of the reviewers. In cases where the intent behind comments was not entirely clear to us, we have responded or made revisions based on our best interpretation of the feedback. We apologize if any concerns have not been fully addressed, and are open to providing more information or making further revisions as necessary. Please find below a point-by-point response in italicized text.*

**Comments to the Authors:
Please note here if the review is uploaded as an attachment.**

Reviewer #1: In this study, the authors proposed an innovative design (ReMEDE) to restrict the spread of CRISPR/Cas9-based homing gene drives by employing the SSA repair pathway to remove the drive element. The manuscript is well constructed, the experiments are reasonably designed, and the results are clearly described. Overall, this is an interesting and well-done study. It fits the publication scope of PLOS Genetics; thus, I recommend it to be published if the authors can address the below concerns.

#1: The authors claimed that the drive allele could be replaced by wildtype allele through SSA. In fact, there was a silent mutation TcG, instead of the real wildtype TGG, left in the genome after SSA. Though leading to a wildtype phenotype, the replaced allele could not be called a wildtype allele. The authors should emphasize this difference and redefine the allele in the manuscript.

*We struggled with this, but in the interest of clarity ultimately made the decision to describe the TcG allele as wildtype. Of course, the reviewer is correct, and has helped us to see that it would in fact be better to more accurately describe the consequences of SSA in the ReMEDE system. Thank you, we have attempted to change all the relevant references to this allele in the manuscript to include more accurate language. If we missed any, we would be happy to revise further*

#2: the end of page 2, the statement may not be quite accurate. “threshold-independent” gene drive should be able to spread in the population even with low initial release frequency. If they need a specific introduction frequency to spread, they are “threshold-dependent”, which could be achieved by adjusting modeling parameters as mentioned in the cited references. But in most cases or parameter range, homing drives are threshold-independent.

*With apologies, we are not quite grasping the reviewer’s argument here. We certainly agree that theoretically homing drives should be threshold independent, but in practice this is not always the case, as evidenced by the yellow drive used in this study. However, if the reviewer would like to make a specific suggestion about how we could improve the accuracy of this statement, we would be happy to incorporate the language.*

#3: Though SSA is a preferred repair pathway in these ReMEDE lines due to the flanking repeats, NHEJ is also a possibility in the repair of the I-SceI induced DNA break. This will generate resistance at the I-SceI target site, which may further spread in the population at a high frequency and impair ReMEDE. I would like to see sequencing evidence of any NHEJ present or absent in this system.

*The reviewer is absolutely correct, NHEJ is a possible mechanism of repair at the double stranded break that occurs at the I-SceI target site. Further, this would indeed ultimately undermine the ReMEDE strategy. Please find the requested sequencing data in Fig. 3A. The raw data is also present in a pdf (produced from a sam file) with the I-Site highlighted in Supplemental Fig. 7. We also included a new simulation, which has a parameter to account for disruption of the I-Site in Supplemental Fig. 5E. From very early on in this work, we ourselves were surprised by the relative stability of the I-Site sequence in the ReMEDE flies. However, the sequencing data indicates that I-SceI resistant alleles will eventually arise. Our modelling suggests that the strategy can still succeed if the rate of resistant allele formation is not too high. However, the genomic context of the site (and drive element) may be important to the stability of the cut site. Thus, a drive in another location may exhibit higher rates of I-SceI resistant allele formation. Therefore, it will be important in future work to explore strategies for mitigating the formation of I-SceI resistant alleles (e.g., including multiple I-Site sequences, etc.). Many such strategies have already been proposed and investigated as Cas9 target sites are also subject to the same concern.*

*The yReMEDE construct has proven to be relatively stable. Sequencing of homozygous females and hemizygous males from the stock line has not revealed any instability in the construct other than at the I-Site sequence (discussed above), or partial repair events.*

*However, the genomic target site location (i.e., the yellow gene) may play a role in the relative stability of the construct. The yellow gene is present on the X-chromosome and males are hemizygous for X. Also, male Drosophila do not undergo meiotic recombination and also exhibit transcriptional suppression of the X chromosome. Additionally, the F1 females generated from male parents are generally heterozygous. These might be mitigating factors influencing the stability of the construct, but testing the ReMEDE system in the context of other gene drives will provide more information.*

#4: As shown in the manuscript, a single I-SceI cut was not enough to always remove the drive allele, even with decent length flanking repeats. Hence, incorporating two I-SceI sites near both ends may help with removing such a large fragment, if they can be cut simultaneously. The other solutions could be using rescue element to eliminate resistant alleles with high fitness cost, or applying CRISPR coupled with multiplexed gRNAs instead of I-SceI.

*Thank you for these suggestions, we are currently investigating these solutions. However, given the limitations of the drive targeting the yellow gene (i.e., resistance allele formation, mating cost, etc.), we are doing this work in the context of other more robust drives. As discussed above, such strategies would also help mitigate the formation of I-SceI resistant alleles. Thank you again.*

#5: page 7, “Further, the overall mean inheritance of the transgene was significantly lower than that of the ReMEDE construct (p value = 0.01; Fig. 2B and C)”. ReMEDE should be corrected as yMCR.

*The comparison is between yEGFP and yReMEDE. The data scoring inheritance of the transgene had a relatively small standard deviation. The yReMEDE data had a much larger standard deviation. Thus, even though the mean values were similar, inheritance of transgene was significantly lower than that of yReMEDE. However, we apologize for the lack of clarity in the description, which led to the confusion. We have edited the text, references to figures, and the Data S1 file in an attempt to make this clearer. Thank you for noting the confusing text.*

#6: near the end of page 7, a low level of maternal deposition was detected by seeing GFP- yellow flies, but it still could not rule out the possibility that Cas9 was much less active (but not inactive at all) in the ReMEDE germline compared to yMCR, which caused low germline cut rate in the first place. It is possible that gypsy elements decreased Cas9 expression.

*Flanking transgenes with insulators has been shown to block the effects of neighbouring enhancers, silencers, as well as heterochromatin (i.e., position effects), in order to ensure that expression of the transgenes is sufficient to produce a detectable phenotype. The gypsy retrovirus insulator in particular has previously been found to boost gene expression to levels severalfold greater than un-insulated loci (Markstein M, Pitsouli C, Villalta C, Celniker SE, Perrimon N. Exploiting position effects and the gypsy retrovirus insulator to engineer precisely expressed transgenes. Nat Genet. 2008 Apr;40(4):476-83. doi: 10.1038/ng.101. Epub 2008 Mar 2. PMID: 18311141; PMCID: PMC2330261.) Thus, it would be surprising if the gypsy elements were actually decreasing Cas9 expression in this context. However, we certainly cannot rule this possibility out, and have revised the discussion to acknowledge this possibility.*

#7: in Figure 3, presenting allele frequencies (or wild-type versus ReMEDE SSA alleles if possible) at the end of the cage experiment would enhance the clarity of the results.

*In our cage experiments, we focused solely on scoring phenotypes, without conducting sequencing. This decision was made due to the very large numbers of wild-type flies involved, among which only a very small percentage would likely carry the TcG allele. Therefore, we regret that we are unable to provide this specific dataset.*

#8: some references may be cited imprecisely in the discussion session. Hammond et al 2017 focused on characterizing resistant alleles, while Unckless et al 2017 presented a modeling paper without demonstrating successful mitigation of resistance alleles.

*Thank you pointing this out. We have removed both references and included additional relevant citations. We sincerely apologize for the oversight.*

#9: based on Figure 2, the germline cut rate in yReMEDE seemed quite low, though about 15% maternal deposition was detected. In Figure 2E, TcG was only detectable in maternal lineage rather than paternal lineage, which indicates that TcG might be derived from the early embryonic cut induced by Cas9/deposition from grandmother. Thus, it is possible that, in the drive mother, double strand break used TcG in the drive as a template and copied it to the original wildtype allele after Cas9/gRNA cut.

*The F1 crosses in these experiments used what has been termed by some in the literature as “master females”. The so-called “master females” are generated by crossing a drive male with wild-type female. Although I don’t think we explicitly used the term “master females” in the originally submitted version of the manuscript, the generation of the female flies used in these crosses is described. In any case, there shouldn’t be any deposition of Cas9 from the grandmother (i.e., grandmaternal effect in the heterogametic sex chromosomes). While we cannot entirely rule out the possibility that the TcG present in the drive allele could have been used as a template to repair the double stranded break in the wild type allele, it seems more likely that these would have been generated by I-SceI, the flanking direct repeats, and SSA. However, we have amended the discussion to acknowledge this possibility.*

#10: the inheritance rate of yReMEDE was ~50% in crosses. With such low inheritance rate, the drive will not be able to spread in the population, just as shown in Figure 3 cage experiments. But in the modeling (Figure 4), why did all the drive increase in the first few generations? This result looks a bit confusing to me.

*A major drawback of the yMCR drive target gene “yellow” is that loss-of-function adversely affects male mating success (Sturtevant, 1915; Merrell, 1949; Bastock, 1956; Barker, 1962; Drapeau et al., 2006; Massey et al., 2019; Xu et al., 2020). Alleles containing indels, generated either through NHEJ or dominant maternal effect, occurred with similar frequency (~3%) with both the yMCR and yReMEDE drives. Any wild-type flies that arise, through the formation of resistant alleles, have an enormous courtship advantage, which results in elimination of the drive allele in population cage studies. This process plays out even more rapidly in the presence of ReMEDE, which is of course designed to generate wild-type resistant alleles. Therefore, the yMCR drive is not an ideal drive for demonstrating the ReMEDE technology in the context of population studies; although, it does have some other advantages. While we are currently transferring the technology to other drives, when we conceived of this work no other drives were available. Thus, we modelled ReMEDE under various parameters in the absence of any mating costs, which is why the drive increases in the first few generations. We also modelled the actual yMCR drive, and this is shown in Fig. S5D. The model approximates fairly well the population cage studies conducted in a wt background (Fig. S5B). The yMCR drive performed better at a lower threshold of introduction when cage studies were conducted in a yellow mutant background, which mitigated some of the mating costs (Fig. S5B).*

Reviewer #2: This paper is a proof of principle study for removing transgene drive alleles and restoring a phenotypical wildtype population using an inducible SSA repair pathway. Although this study proves that drive conversion and excursion of drive elements can happen simultaneously and produce more wildtype progeny, its medium drive efficiency and low SSA rate might limit its application. The modelling simulation suggests that drive alleles can be replaced by the engineered resistant allele over generations even with a low SSA rate when ignoring fitness cost and any other form of resistant allele.

Some specific comments on the manuscript:
1. In Figure S3, WT progeny is indicated as the result of the SSA event. However, as the authors discussed on page 13, these can also be uncut alleles, especially considering all ReMEDE drive alleles contain that TcG maker, and how WT alleles are repeatedly cut by Cas9 to form indel resistant alleles in yMCR crosses. Crossing the drive with an external source of Cas9 might provide some insight into whether or not the Cas9 is adequately expressed. This issue also applies to using the increase in WT progeny in yReMEDE crosses as a proxy for the SSA rate as stated on page 8 and later in the discussion on page 12. The percentage of WT progeny increases from ~2% to ~38% in Figure 2C, but nearly 29% of them are WT without the engineered TCG maker. It’s important to give a clear statement of the rate of SSA events under various scenarios and include it in the abstract so that readers can have a general impression at just a glance.

*Alleles with indels generated either through NHEJ or “dominant maternal effect” were found to occur at a frequency ~3% in the progeny of the F1 yReMEDE females. These results are consistent with previous studies with the yMCR drive where indels were found to occur with similar frequency (Gantz and Bier, 2015; Champer et al., 2017; Xu et al., 2020). This being the case, we are honestly at a loss to explain the origin of the wild type alleles with TGG or gGG. However, the reviewer is correct that we should be careful to not to mislead anyone about the rate of SSA events, as the engineered silent TcG marker is the only evidence of bonafide SSA. Thus, we have revised the abstract in accordance with the reviewer’s suggestion. We would be happy to revise further, should the reviewer deem it necessary. Thank you for this suggestion.*

2. Figure 4: In figure 4B, authors included functional, non-functional and engineered drive resistant alleles. However resistant alleles can also form from NHEJ repair at the I-SceI cutting site. This type of resistant allele will prevent DSB thus SSA, allowing the drive allele to maintain or even spread, if the drive conversion rate allows, in the population. Do authors see any evidence of I-SceI resistance and at what frequency?

*The reviewer is absolutely correct, NHEJ is a possible mechanism of repair at the double stranded break that occurs at the I-SceI target site. Further, this would indeed ultimately undermine the ReMEDE strategy. Please find the requested sequencing data in Fig. 3A. The raw data is also present in a pdf (produced from a sam file) with the I-Site highlighted in Supplemental Fig. 7. We also included a new simulation, which has a parameter to account for disruption of the I-Site in Supplemental Fig. 5E. From very early on in this work, we ourselves were surprised by the relative stability of the I-Site sequence in the ReMEDE flies. However, the sequencing data indicates that I-SceI resistant alleles will eventually arise. Our modelling suggests that the strategy can still succeed if the rate of resistant allele formation is not too high. However, the genomic context of the site (and drive element) may be important to the stability of the cut site. Thus, a drive in another location may exhibit higher rates of I-SceI resistant allele formation. Therefore, it will be important in future work to explore strategies for mitigating the formation of I-SceI resistant alleles (e.g., including multiple I-Site sequences, etc.). Many such strategies have already been proposed and investigated as Cas9 target sites are also subject to the same concern.*

*The yReMEDE construct has proven to be relatively stable. Sequencing of homozygous females and hemizygous males from the stock line has not revealed any instability in the construct other than at the I-Site sequence (discussed above), or partial repair events.*

*However, the genomic target site location (i.e., the yellow gene) may play a role in the relative stability of the construct. The yellow gene is present on the X-chromosome and males are hemizygous for X. Also, male Drosophila do not undergo meiotic recombination and also exhibit transcriptional suppression of the X chromosome. Additionally, the F1 females generated from male parents are generally heterozygous. These might be mitigating factors influencing the stability of the construct, but testing the ReMEDE system in the context of other gene drives will provide more information.*

3. I recommend rearranging figures so that all information necessary for understanding this paper is presented in main figures, and supplementary figures don’t present the same information repeatedly. Now readers have to switch between main figures and supplementary figures multiple times while reading just one paragraph, e.g. the last paragraph on page 7. Also in Figure 2C, those yellow/brown fly legends only apply to yMCR crosses, but the graph also contains yReMEDE result and can be confusing.

*While we thank the reviewer for this suggestion, we simply could not figure out how to include all of the data in a single figure. However, we have tried to include all of the data necessary for understanding the paper in the main figures, with the supplemental figures simply offering more granular details or finer parsing of data in most cases. However, if the reviewer feels that a specific data set or panel is essential for understanding the study or its conclusions, then we could certainly try to bring it into the main figure set.*

*Regarding Figure 2C, the legends are actually applicable to both the yMCR and yReMEDE crosses, as the phenotypic scoring was identical. This is because with exception of the ReMEDE components, the two drives are otherwise identical.*

Minor comments:
1. In-frame mutation does not guarantee to be functional alleles, as indicated in multiple locations.

*The reviewer is correct that this was an oversight on our part, and thank you for catching this. We have corrected the key and legend for Fig. 2. We apologize if we missed any other locations, and will correct if noted.*

2. What’s the estimated number of progeny for each generation in population cage?

*We have included this information in the methods and/or results section. However, for cage studies shown in Fig. 3, 200 flies were seeded (25% wild type and 75% drive) in G0. In subsequent generations, 150 progeny from the previous generation were seeded into new cages, while another 150 flies were phenotypically scored.*

*The cage studies were conducted similarly to those described in the previously published paper by Xu, X.R.S., Bulger, E.A., Gantz, V.M., Klanseck, C., Heimler, S.R., Auradkar, A., Bennett, J.B., Miller, L.A., Leahy, S., Juste, S.S., Buchman, A., Akbari, O.S., Marshall, J.M., Bier, E., 2020. Active Genetic Neutralizing Elements for Halting or Deleting Gene Drives. Mol. Cell 80, 246-262.e4.* [*https://doi.org/10.1016/j.molcel.2020.09.003*](https://doi.org/10.1016/j.molcel.2020.09.003)*.*

3. On page 9, near the top, it’s Fig. S2E not S2D

*Thank you for catching this error, we have corrected.*

Reviewer #3: Chennuri et al. test a gene drive reversal system in Drosophila that they proposed previously in a theoretical paper (Zapletal et al., 2021). The concept of this system, which is based on a large and expanding literature in yeast, mammalian cells, mosquitoes, and flies is based on a DNA repair pathway referred to as Single Strand Annealing (SSA). This repair pathway can trigger recombination between two directly repeated sequences in the genome if one induces a double strand DNA break (DSB) between the repeats, which results in a clean deletion of sequences between them. The authors had proposed and now test whether a system might be used to cleanly eliminate a Cas9-based gene drive system that was flanked by direct sequence repeats by engineering a gene drive to carry a second nuclease (I-SceI) that cuts at a target site also included in the gene drive element lying between the two directly repeated sequences. They refer to this system as a "Repeat Mediated Excision of a Drive Element" (ReMEDE).

The authors begin by inserting a series of fluorescently marked backbone constructs lacking CRISPR components but carrying an I-SceI target site flanked with differing lengths of direct repeats into the the Drosophila yellow locus at a site used for a previously well-studied gene drive system. Insertion of these cassettes interrupts the coding region of the yellow locus resulting in yellow mutant phenotype. They generate two forms of the repeat-flanked constructs, one carrying both the I-SceI nuclease under control of a drug inducible promoter as well as the I-SceI target site (cis version), and another lacking I-SceI but carrying the nuclease target site (trans version). The authors also introduce a silent sequence polymorphism just outside of the 3' direct repeat that can be used to distinguish cis deletion events from a reference WT allele. The trans version can then be combined with a GAL4-UAS regulated form of I-SceI to induce a DSB at the target site within the test construct. SSA events can be detected by elimination of two fluorescent marker genes as well as restoration of a WT pigmentation phenotype (and presence of the linked sequence polymorphism by PCR and sequence analysis). They compare the efficiencies of these two systems and make several observations. First, using the trans version, the authors observe frequent I-SceI induced deletion of the gene cassette which increases for repeats of 500 bp (~35%) compared to those of 250 bp (~15%). Shorter 30 bp repeats yielded few SSA events. Second, they note that the frequency of such expected cis-generated events is substantially higher (> 2 fold) in F2 offspring derived from gene cassettes carried by male versus female F1 parents carrying the gene cassette and I-SceI. Third, they observe much low frequencies of SSA for the cis version of the construct and these low frequencies do not increase even upon drug induction of the I-Sce1 transgene. The authors attribute this lack of inducibility to leaky drug-independent expression of the I-Sce1 transgene, although why these levels are so much lower than for the trans arrangement remains unexplained.

The authors then use Phi-C31 mediated recombination to insert core gene drive components into a cassette carrying the cis acting components of the I-SceI SSA system (including the flanking direct repeats) at the same site in the yellow locus. The resulting composite element carryies Cas9, a guide RNA for copying the drive element, I-SceI, and the I-SceI target site (note that the authors do not generate an important control element carrying both nucleases in the absence of the direct repeats - see critique). They compare the drive performance of the core drive (marked with eGFP) with the derived ReMEDE element and find that including the hypothesized leaky I-Sce1 cassette decreases the observed biased drive from ~87% for the core element to nearly Mendelian levels for the ReMEDE element. They also observe a much greater fraction of WT alleles for the ReMEDE element compared to the core drive which is more pronounced in female than male progeny by nearly a factor of 2. The authors also perform sequence analysis of progeny with a WT phenotype to determine whether they carry the expected cis-linked sequence polymorphism expected to be associated with SSA induced events. Curiously they find a mix of different linked allelic marker sequences that suggest something more complicated than simple SSA must be taking place. The authors propose a mix of copying of the gene element and SSA may be occurring.

Finally, the authors conduct a multi-generation population experiment in which they seed cages with 75% drive (core or ReMEDE) and 25% WT. They observe a gradual reduction in the frequency of the core drive and a corresponding increase of WT alleles over 14 generations. This result is not expected since the drive is expected either copy itself or generate NHEJ mutations that should have a mutant yellow phenotype as observed in their pair mating experiments (see critique below). In contrast, the ReMEDE element disappears steadily and completely over the course of 14 generations. The authors conclude that the ReMEDE system can promote restoration of a WT phenotype under these conditions, although this experiment does not probe a key question of whether this system might be employed in tandem with successful drive of an element followed by its elimination with ReMEDE.


Major critique points:

The concept behind this study of employing a repeat flanked drive system with SSA to restore a population to a WT phenotype is interesting and merits experimental testing as was the worthy goal of this study. Unfortunately, the data presented in the current manuscript to not provide sufficient information to test this idea in an informative fashion. There are several aspects of this study would require greater analysis to arrive at a proper evaluation of this methodology as summarized in the points below.

1) It is not clear why the cis acting version of the I-SceI bearing element is so much less efficient than the trans version or than observed in a variety of other prior studies in which SSA is used for a similar purpose to delete a gene cassette (see minor points below). Since the authors go on to use this inefficient cis configuration in their ReMEDE construct understanding why is a significant issue and bears on all subsequent results in the study. One way the authors could solve this problem is to include a different conditional system for expressing I-SceI such as a nos>GAL4 driven I-SceI (the same used for their active trans version) plus a temperature sensitive GAL80 inhibitor. Another approach would be to use a CRE-LOX type of system (which works very well in Drosophila) to flip out a conditional transcriptional termination cassette.

*We also were surprised by the low efficiency of the cis configuration. However, in this study we chose to investigate the configuration where the excision of the transgene would be autocatalytic over the period of gene drive, eventually restoring the wild-type population, with drive activity gradually reduced as drive alleles are converted to wild-type alleles. Such a configuration would have a number of attractive features as a gene drive mitigation strategy, notably its potential to restore wild-type populations without additional transgenic releases or large-scale environmental modifications. Further, our previous modelling studies suggested that even low levels of SSA would be sufficient to replace drive alleles over time. However, our previous work on this topic was entirely conceptual, and as such we could not know exactly how this technology would perform. While we demonstrate here proof-of-concept, the technology may indeed be better suited for application as an in trans braking system. The system that we used is chemically regulated, by the drug mifepristone. Prior to settling on the gene switch system, we tested and evaluated multiple inducible systems, including others that were also chemically regulated (i.e., EcDHFR.I-SceI system with Trimethoprim), and all exhibited some level of leakiness. Additionally, we reached out to authors of papers describing additional systems in order to obtain constructs. Based on our own testing, and correspondence/conversation with these authors, we are not aware of any system that does not exhibit some level of leakiness. However, we thank the reviewer for trying to offer possible solutions to this problem.*

2) It is not clear that the results the authors report for the core drive versus the ReMEDE element are due to activity of the I-SceI system. This concern arises from several reasons including:

i) The issue raised above regarding the low SSA efficiency of the cis-acting I-SceI cassette.

*Sequencing of F2 progeny with wild type phenotypes confirmed the presence of TcG, which was engineered to provide a marker of SSA. Thus, the most likely explanation for the presence of this marker would seem to be that these were generated by I-SceI, the flanking direct repeats, and SSA. However, we have amended the manuscript to acknowledge various other possibilities as well.*

ii) Most critically, the absence of proper control drive constructs that lack the I-SceI cassette (or essential parts of it) in the presence of the CRISPR components (as indicated in Fig. S2, the authors added the CRISPR components to the repeat flanked I-SceI element using Phi-C31 recombineering but did not generate a repeat flanked core drive element lacking the I-SceI components). This is a serious concern since one could wonder whether all the observed results with ReMEDE construct are actually due to Cas9-mediated cleavage and recombination induced with either cis or trans repeat sequences (indeed the result seemingly indicated by their polymorphism sequence analysis) rather than by I-SceI induced SSA as the authors infer.

*With apologies, we do not completely understand the reviewer’s comment. There appears to be some misunderstanding regarding how the technology works. The engineered silent mutation disrupts the PAM site in the ReMEDE drive allele, preventing cutting by Cas9. Thus, just as with the conventional yMCR drive, only wild-type alleles are targeted by Cas9. Further, even if the Cas9 were to somehow produce a double stranded break in the target site with a mutated PAM sequence, this sequence is located outside the direct repeats. Thus, the most likely explanation for the presence of the TcG marker is SSA mediated by I-SceI. In reviewing the text of the manuscript, we identified some language that may have been the source of this confusion and have revised. We hope that these changes provide more clarity; however, we are happy to revise further if necessary.*

iii) Another good construct control would include Cas9, guide RNA, I-SceI nuclease plus I-SceI target site and no direct repeats. This construct would assess the potential role of dual nucleases potentially creating damaged target alleles as may be the case given that WT progeny are nearly twice as abundant in females that could carry such alleles in a heterozygous condition than in males where such alleles would be lethal.

*Apologies again, we are not sure that we understand the reviewer’s comment. Only one nuclease, Cas9, cuts the wild type target allele. The I-SceI nuclease can only cut the drive allele, and the Cas9 can only cut the wild type target allele (see above). Without the direct repeats being present in the gene drive SSA cannot occur. Even the 30bp direct repeats that we tested did not result in SSA, likely because they were below a minimum effective processing segment length. The double stranded break must be repaired; thus, this would increase NHEJ resulting in more rapid loss of the I-Site target sequence. I am not sure we understand the value of this control, as the choice of DNA repair pathways would be different in the absence of the direct repeats. Our sequencing did not reveal any evidence of damaged target alleles.*

*However, the genomic target site location (i.e., the yellow gene) may play a role in the relative stability of the construct. The yellow gene is present on the X-chromosome and males are hemizygous for X. Also, male Drosophila do not undergo meiotic recombination and also exhibit transcriptional suppression of the X chromosome. Additionally, the F1 females generated from male parents are generally heterozygous. These might be mitigating factors influencing the stability of the construct, but testing the ReMEDE system in the context of other gene drives will provide more information.*

3) Why do WT alleles persist in the gene drive cage experiments using the core drive element which the authors show either copies itself (87%) or generates NHEJ mutations (8-11%) in single generation crosses? Are they using a cut resistant WT allele of yellow? The results and figure legend do not specify whether this might be the case. If so, is the same WT cut resistant target allele being used for the ReMEDE construct. If this is the case, the experiment shown in Fig. 3 is not very informative since there would be no Cas9-mediated target cleavage. Perhaps I am missing something?

*A major drawback of the yMCR drive target gene “yellow” is that loss-of-function adversely affects male mating success (Sturtevant, 1915; Merrell, 1949; Bastock, 1956; Barker, 1962; Drapeau et al., 2006; Massey et al., 2019; Xu et al., 2020). Alleles containing indels, generated either through NHEJ or dominant maternal effect, occurred with similar frequency (~3%) with both the yMCR and yReMEDE drives. Any wild-type flies that arise, through the formation of resistant alleles, have an enormous courtship advantage, which results in elimination of the drive allele in population cage studies. This process plays out even more rapidly in the presence of ReMEDE, which is of course designed to generate wild-type resistant alleles. Therefore, the yMCR drive is not an ideal drive for demonstrating the ReMEDE technology in the context of population studies; although, it does have some other advantages. Notably, the sex linkage of the yellow target gene, a phenotype (yellow body color) that can be easily scored, and in the case of our modified construct, a fluorescent marker (GFP), all of which when combined permit us to track nearly every genetic outcome possible. In cage trials, yellow males are generally rejected by both wild-type and yellow females. However, In pair-mated crosses there is no such issue as the females have no mate choice. We are currently transferring the ReMEDE technology to other drives, but when we first conceived of this work no other drives were available. The studies shown in Fig. 3 were conducted as those previously published in Xu, X.R.S., Bulger, E.A., Gantz, V.M., Klanseck, C., Heimler, S.R., Auradkar, A., Bennett, J.B., Miller, L.A., Leahy, S., Juste, S.S., Buchman, A., Akbari, O.S., Marshall, J.M., Bier, E., 2020. Active Genetic Neutralizing Elements for Halting or Deleting Gene Drives. Mol. Cell 80, 246-262.e4.* [*https://doi.org/10.1016/j.molcel.2020.09.003*](https://doi.org/10.1016/j.molcel.2020.09.003)*. As such, the results of the cage trials with the yMCR drive are very similar to those obtained in the aforementioned study, which used a nearly identical drive.*

4) The authors should consider and experimentally evaluate the possibility mentioned above (point 2, section iii) that a combined action of Cas9 plus I-SceI cleavage could lead to damaged chromosome targets in which a partial copy event mediated by Cas9 was then cleaved by I-SceI leading to DNA repair failure and potential chromosome-scale damage. One way to address this important question would be to use visible or dominant markers that are closely linked to the drive element to distinguish donor from receiver chromosomes.

*The sex linkage of the yellow gene actually permits tracking of the chromosomes containing drive alleles versus wild-type alleles. For example, all male progeny of these experiments would necessarily inherit an X chromosome from the F1 female. Similarly, all female progeny would necessarily inherit one wild-type X chromosome from the male parent, and an allele from the female parent. This knowledge combined with sequencing did not produce any evidence of chromosomal-scale damage. Of course, we cannot rule out the possibility that such events may have occurred but were not observed (i.e., due to developmental lethality). However, this would also be true if the donor and receiver chromosomes were marked.*

5) A very significant limitation of the current study is that the authors do not test a ReMEDE system in the context that they are imagined to be useful namely in which a gene drive is allowed first to spread through a population (with the ReMEDE system off) and then have that cassette removed cleanly by switching the ReMEDE system on. Several suboptimal features of their system preclude such a key test including the absence of conditional activation of the ReMEDE system. This is really is an essential experiment and without it, one cannot judge the potential of the ReMEDE concept. As suggested in Point 1, the problem of developing a robust conditional system for activating I-SceI activity is one that needs to be solved in its own right for proper evaluation of ReMEDE as a viable system.

*When we started this study, we were unaware of the leakiness in the gene switch and other inducible systems. Similarly, we were unaware of any issues (i.e., mating costs) with the yMCR drive that might be less than ideal for population cage trials. The original manuscript describing this gene drive only included single pair mated crosses. We are currently transferring this technology into more robust gene drives and also planning to evaluate in the context of in trans braking system. Nevertheless, our previous work in this area was entirely conceptual, and it was unclear if the concept would work in an actual biological system. In the present study, we demonstrate proof-of-concept in a major genetic model organism with an extensively characterized gene drive. There is currently no working solution for the mitigation of gene drives. While a number of different possible strategies have been published, all of these solutions require further research and development.*

In summary, in my view, the study does not adequately resolve key mechanisms underlying the results they observe nor allow one to evaluate the potential of the ReMEDE type of drive reversal system. I therefore do not recommend publishing this manuscript in its current form. If the authors provide additional experimental evidence to resolve the issues raised above, a revised manuscript including these new results may merit re-evaluation.

Minor Points:

1) The genetic crossing scheme and color code for differing alleles in Fig. 2c are difficult to understand since the WT alleles in the parents are indicated by a blue maternal X or paternal X', but no such symbols are shown in the progeny. If all such alleles are assumed to being mutated then that should be explained and how that relates exactly to the different gray shaded X or X' alleles.

*Theoretically, no such alleles should exist in the progeny of a cross involving a gene drive, which is why they are not shown. We have edited figure legend to include this.*

2) Several prior references demonstrating SSA in insects should be cited by the authors:

1. Grigoraki L, Cowlishaw R, Nolan T, Donnelly M, Lycett G, Ranson H. CRISPR/Cas9 modified An. gambiae carrying kdr mutation L1014F functionally validate its contribution in insecticide resistance and combined effect with metabolic enzymes. PLoS Genet. 2021;17(7):e1009556. Epub 2021/07/07. doi: 10.1371/journal.pgen.1009556. PubMed PMID: 34228718; PubMed Central PMCID: PMCPMC8284791.

*There is no mention of single strand annealing (SSA) in this reference. However, we would be happy to include it in our manuscript if the reviewer would kindly tell us where it should be cited.*

2. Li X, Bai Y, Cheng X, Kalds PGT, Sun B, Wu Y, et al. Efficient SSA-mediated precise genome editing using CRISPR/Cas9. FEBS J. 2018;285(18):3362-75. Epub 2018/08/08. doi: 10.1111/febs.14626. PubMed PMID: 30085411.

*This manuscript describes a method of gene editing that uses Cas9 and SSA-mediated repair. The studies were conducted in Human embryonic kidney cells. Again, we would be happy to include the reference if the reviewer would kindly tell us where it should be cited.*

3. Morianou I, Crisanti A, Nolan T, Hammond AM. CRISPR-Mediated Cassette Exchange (CriMCE): A Method to Introduce and Isolate Precise Marker-Less Edits. CRISPR J. 2022;5(6):868-76. Epub 2022/11/16. doi: 10.1089/crispr.2022.0026. PubMed PMID: 36378258.

*This manuscript describes a variation on the well-established recombinase-mediated cassette exchange procedure using CRISPR in Anopheles gambiae. We found no mention of SSA, but would again be happy to include it if the reviewer would kindly tell us where it should be cited.*

4. Quinn C, Anthousi A, Wondji C, Nolan T. CRISPR-mediated knock-in of transgenes into the malaria vector Anopheles funestus. G3 (Bethesda). 2021;11(8). Epub 2021/12/02. doi: 10.1093/g3journal/jkab201. PubMed PMID: 34849822; PubMed Central PMCID: PMCPMC8496255.

*This article describes CRISPR-mediated knock-in of transgenes in Anopheles funestus, but makes no mention of SSA. Again, if the reviewer would kindly tell us where it should be cited, we will be happy to include it.*

Reviewer #4: Uploaded as an attachment. (Added text from attachment here)

In this study, the authors demonstrated a precise self-elimination of engineered gene drive components to restore the wild population in *D. melanogaster*. A number of modern gene drives have been developed to curb human-disease vectors and agricultural pests, and have been found efficient at lab scale, but none of them tested in field yet due to a variety of concerns including safeguards or unintended impacts upon release. A few self-limiting homing drives have been designed and developed for a controlled spread of the drive elements. However, development of a seamless self-elimination enabled homing drives as developed by the authors here, would offer more nature safe drive options. In a separate study (Zapletal et al., 2021) using mathematical models, the authors have shown the potential of such self-eliminating autonomous gene-drives in restoring the wild population over the time, at a rate of 10% drive elimination events.

Here, firstly the authors tested a Repeat Mediated Excision of a Transgene (ReMET), which relies on DSB repair by SSA repair pathway. The transgene cassette, consist of direct repeats, removal was assessed in cis (all in one) and trans (split) configurations, using UAS/GAL4 approach. The authors noted a significant SSA repair events depending on the ‘direct repeat’ sizes. Similarly, the authors designed a full CRISPR/Cas9-based gene drive cassette, consists of flanking direct repeats (250 bp) and inducible nos-GeneSwitch (RU486 induced GAL4), placed in the exon-2 of the yellow gene and successfully restored a wild type of population. The authors named this technology ‘Repeat Mediated Excision of a Drive’ (ReMEDE). The authors smartly mutated the PAM site in the right homology arm/sequence of the drive construct to score the SSA generated wild type allele upon the drive cassette removal. Moreover, the cage experiment was performed to evaluate ReMEDE potential, and authors noted absolute drive removal within 10-14 generations.

I have a couple of major and minor comments that would need to be addressed before the paper would be acceptable for publication.

Major Comments:

1. It is interesting to note that the SSA repair was relatively high for ReMET (6%-35%) in *trans* where the I-Site site was not exposed to the endonuclease prior to the F_0_ crosses (Fig 1B) whereas it was just 0.5%-1% in *cis* configuration. Importantly, with *cis* ReMET the leaky expression of I-SceI can result in an I-Site resistance allele formation. I’d suggest assessing the I-Site sequence intactness in the strains/stock (*cis* F_0_). The I-Site DSB if repaired by error prone NHEJ can reduce the SSA repair in the *cis* self-crosses shown. The authors should also cross the *cis* ReMET with *yellow* mutants to assess the SSA for male and female germlines respectively. Do the authors find mosaic phenotype in yReMET (cis) like trans (Fig 1)?

*We did in fact sequence over the I-Site sequence in the in cis yReMET stock lines prior to performing the self-crosses shown. Please see representative sequences below from seven different lines possessing various direct repeat sizes (30, 250, and 500). While the data suggests that the I-Site was intact at the time these experiments, and probably did not influence SSA repair in the crosses shown, more recent sequencing (not shown) did reveal deletions present in these lines. We have revised Fig. 3A to include sequencing data that we generated over time at the I-Site sequence in the yReMEDE line. The sequences are also included in the raw data (Supplemental Fig. 7) as a pdf (produced from a sam file) with the I-Site highlighted. The data show that, at least at this target site (i.e., the yellow gene), resistance appears to arise at relatively low frequency. However, just as observed in the yReMET lines, we do eventually see evidence of I-SceI resistance mediated by NHEJ, which could ultimately undermine the ReMEDE strategy. Given this, we have added a new simulation, which includes a parameter that accounts for disruption of the I-Site (Supplemental Fig. 5E). The modelling suggests that the strategy can still succeed if the rate of resistant allele formation is not too high. Nonetheless, other work in the published literature, and in our laboratory, suggests that the genomic context of the site (and drive element) may be important to the stability of the cut site. Thus, a drive in another location may exhibit higher rates of I-SceI resistant allele formation. Therefore, it will be important in future work to explore strategies for mitigating the formation of I-SceI resistant alleles (e.g., including multiple I-Site sequences, etc.). Many such strategies have already been proposed and investigated as Cas9 target sites face the same issue.*

*We tried to perform the requested crosses with ReMET lines assessing SSA in the male and female germlines, but discovered the aforementioned deletions present in the I-Site sequence. Therefore, we need to remake the lines. Thus, we regret that we are unable to provide this specific dataset at this time.*

*While these phenotypes were observed in the in trans experiments, we did not observe mosaic phenotypes in experiments with the in cis yReMET lines. We speculate that this may have something to do with differences in the timing of I-SceI nuclease cutting.*

3. It is not clear why did the authors decide to develop a self-elimination full drive (yReMEDE) using the inducible *nos*-GS system which showed such a little SSA and a leaky expression too. Surprisingly, here the authors noted a relatively high rate of SSA and large number of wild type flies’ generation with the yReMEDE crosses compared to yMCR drive, that’s great! were these crosses performed on RU486 or without it? should mention in the results or M&M section. Also, did the authors get the yellow F1 females for yMCR as well or it was only with the yReMEDE (Fig.2C)? do the authors think that the use of gypsy elements in ReMEDE construct (unlike yMCR) modulated Cas9/gRNA expression (more precise), and likely reduced the Cas9/RNP deposition so that paternal alleles remained un-cut for yReMEDE’s wild type offspring (F2)?

*We also were surprised by the low efficiency of the cis configuration. However, in this study we chose to investigate the configuration where the excision of the transgene would be autocatalytic over the period of gene drive, eventually restoring the wild-type population, with drive activity gradually reduced as drive alleles are converted to wild-type alleles. Such a configuration would have a number of attractive features as a gene drive mitigation strategy, notably its potential to restore wild-type populations without additional transgenic releases or large-scale environmental modifications. Further, our previous modelling studies suggested that even low levels of SSA would be sufficient to replace drive alleles over time. However, our previous work on this topic was entirely conceptual, and as such we could not know exactly how this technology would perform. While we demonstrate here proof-of-concept, as the reviewer suggests the technology may indeed be better suited for application as a braking system (i.e., in trans configuration).*

*We did perform crosses on RU486, and this data is shown in Supplemental Figures IC and 2E. However, the presence of RU486 did not significantly increase SSA. We subsequently found that the second nuclease is inducible from the construct that is included in the ReMEDE system, but is also leaky (Fig S1D). As higher levels of nuclease production did not correlate with higher levels of SSA, we concluded that the level of leaky expression already resulted in this pathway being chosen for repair of the double stranded break at the maximum frequency (at least with regard to the in cis configuration). Thus, the crosses shown in the main figures did not include RU486.*

*We did obtain yellow F1 females with yMCR. Our results with the yMCR drive, in both single pair mated and multi-generational cage trials, were nearly identical to those previously published by Ethan Bier’s laboratory (Gantz and Bier, 2015; Xu et al., 2020).*

*Flanking transgenes with insulators has been shown to block the effects of neighbouring enhancers, silencers, as well as heterochromatin (i.e., position effects), in order to ensure that expression of the transgenes is sufficient to produce a detectable phenotype. The gypsy retrovirus insulator in particular has previously been found to boost gene expression to levels severalfold greater than un-insulated loci (Markstein M, Pitsouli C, Villalta C, Celniker SE, Perrimon N. Exploiting position effects and the gypsy retrovirus insulator to engineer precisely expressed transgenes. Nat Genet. 2008 Apr;40(4):476-83. doi: 10.1038/ng.101. Epub 2008 Mar 2. PMID: 18311141; PMCID: PMC2330261.) Thus, it would be surprising if the gypsy elements were actually decreasing Cas9 expression in this context. However, we certainly cannot rule this possibility out. More precise expression of Cas9 could also be a possibility, and if true this would be very interesting. We have revised the discussion to acknowledge this possibility.*

4. Importantly, in the final yReMEDE construct a number of repetitive sequences/elements are present, I fear that these repetitive elements are prone to breakdown/rearrangements during homing, and can be lost during the repair processes (Simoni A et al, 2014 Nucleic Acids Research, Yang E et al, 2022 G3, Oberhofer et al, 2018), did the authors perform any assessment on the construct stability? or noted partial repair events?

*The yReMEDE construct has proven to be relatively stable. Sequencing of homozygous females and hemizygous males from the stock line has not revealed any instability in the construct other than at the I-Site sequence (discussed above), or partial repair events.*

*However, the genomic target site location (i.e., the yellow gene) may play a role in the relative stability of the construct. The yellow gene is present on the X-chromosome and males are hemizygous for X. Also, male Drosophila do not undergo meiotic recombination and also exhibit transcriptional suppression of the X chromosome. Additionally, the F1 females generated from male parents are generally heterozygous. These might be mitigating factors influencing the stability of the construct, but testing the ReMEDE system in the context of other gene drives will provide more information.*

**Minor comments:**

1. M&M: The authors should provide more details on the plasmid’s design/construction and cloning procedures followed.

*More detailed cartoons illustrating the design, construction, and cloning of constructs are included in the supplemental figures. Additionally, we have provided maps and the full sequences of all plasmids in the supplemental figures.*

2. M&M: The total numbers of genetic crosses performed (pair or *en masse*) for ReMET and others should be mentioned. The progeny counts can be added to the supplementary info or results.

*The total number of crosses are indicated in the figures and the rest of the text as n= x. We also have revised to include progeny counts in the supplementary raw data spreadsheet, S1.*

3. M&M: the authors should provide more details on the cage set up. Should include total cages set (control/test), insect release ratio, days allowed for the egg laying, cage dimensions, RU486 (conc.) used or not etc. The screening/phenotype, progeny counts can be added to the supplementary info.

*These details are included in the Results section. The cage trials were performed as described in previously published studies with the yMCR drive (Bishop et al., 2022; Xu et al., 2020). We also have revised to include progeny counts in the supplementary raw data spreadsheet, S1.* Cage studies: Fig 3, even with the yMCR’s high release ratio, why it never surged above the initial releases and the population remained almost steady (WT:yMCR) until gen-8-9, later the wild population gained? Did the authors note resistant alleles’ accumulation and/or drive mating/fitness costs? The later population has all the wild type of body color flies or yellow/mutants? The authors should provide more details on the observations/results etc.

*A major drawback of the yMCR drive target gene “yellow” is that loss-of-function adversely affects male mating success (Sturtevant, 1915; Merrell, 1949; Bastock, 1956; Barker, 1962; Drapeau et al., 2006; Massey et al., 2019; Xu et al., 2020). Alleles containing indels, generated either through NHEJ or dominant maternal effect, occurred with similar frequency (~3%) with both the yMCR and yReMEDE drives. Any wild-type flies that arise, through the formation of resistant alleles, have an enormous courtship advantage, which results in elimination of the drive allele in population cage studies. This process plays out even more rapidly in the presence of ReMEDE, which is of course designed to generate wild-type resistant alleles. Therefore, the yMCR drive is not an ideal drive for demonstrating the ReMEDE technology in the context of population studies; although, it does have some other advantages. Notably, the sex linkage of the yellow target gene, a phenotype (yellow body color) that can be easily scored, and in the case of our modified construct, a fluorescent marker (GFP), all of which when combined permit us to track nearly every genetic outcome possible. In cage trials, yellow males are generally rejected by both wild-type and yellow females. However, in pair-mated crosses there is no such issue as the females have no mate choice. We are currently transferring the ReMEDE technology to other drives, but when we first conceived of this work no other drives were available. The studies shown in Fig. 3 were conducted as those previously published in Xu, X.R.S., Bulger, E.A., Gantz, V.M., Klanseck, C., Heimler, S.R., Auradkar, A., Bennett, J.B., Miller, L.A., Leahy, S., Juste, S.S., Buchman, A., Akbari, O.S., Marshall, J.M., Bier, E., 2020. Active Genetic Neutralizing Elements for Halting or Deleting Gene Drives. Mol. Cell 80, 246-262.e4. https://doi.org/10.1016/j.molcel.2020.09.003. As such, the results of the cage trials with the yMCR drive are very similar to those obtained in the aforementioned study as well.*

4. Also, with the yReMEDE, it seems like the SSA dominate over the homing and the drive alleles could not sustain in the population even at initial generations. did the authors assess the SSA (TcG) in the later population or the rapid elimination is due to the associated fitness costs (if any) of the yReMEDE strains and overall reduced homing? the authors should provide more discussion, since the SSA events noted with the pair crosses were low. Did the authors note I-Site resistance alleles accumulation too in the population?

*In our cage experiments, we focused solely on scoring phenotypes, without conducting sequencing. This decision was made due to the very large numbers of wild-type flies involved, among which only a very small percentage would likely carry the TcG allele. Please also see previous responses above.*

5. A direct repeat sequence (DR 250 bp) is placed close to the right homology site to achieve SSA repair. Do the authors consider that it could impede the entire cassette’s homing being a potential left homology for the Cas9/DSB repair as well? If yes, can be discussed.

*I am not sure that we completely understand the reviewer’s comment, but the Cas9 target site where the double stranded break occurs is located outside the direct repeats. Apologies, if we have misunderstood what the reviewer is proposing.*

6. Also, the I-Site is positioned asymmetrically in the construct, won't be good to keep it a bit symmetrical or use I-Sites on both the ends? Probably, it’d shorten the DRs exposure time for SSA.

*Thank you for the suggestion, we are currently investigating this configuration. However, given the limitations of the drive targeting the yellow gene (i.e., resistance allele formation, mating cost, etc.), we are doing this work in the context of other more robust drives. As discussed above, such a strategy would likely also help mitigate the formation of I-SceI resistant alleles. Thank you again.*

7. The authors should also add a comment on the ReMEDE stock/line maintenance in the lab.

*Every 3rd generation, gene drive flies were scored and manually sorted to remove phenotypes consistent with resistant alleles before tipping into new vials and bottles. We have added this to the M&M section in the manuscript.*

9. Term ‘genetic drive’ can be replaced with ‘gene drive’ to be consistent throughout the main text.

*We have corrected all instances that we could find, but apologize if we missed any other locations, and will correct if noted.*

Reviewer #5: The article describes proof of principle for one type of 'self-extinguishing' gene drive. The self-extinguishing nature is designed to come from internal cleavage of the drive element that can repair in a way that both removes the element and leaves in its place an (almost) wild type allele that is resistant to future gene drive activity. In reality the self-extinguishing nature of the element described and validated in this article is afforded by the negative fitness costs associated with the gene drive element itself, as well as its propensity to generate drive-resistant alleles.

I think there are elements of interest here for the gene drive field, in terms of the proof of principle of this type of self extinguishing element. However, I find this article to be a real mixed bag - elements of extraordinary clarity and lucidity interspersed with long sections of non-selective descriptions of results that are often incidental to the real points of interest and novelty. For example, the Introduction is one of the best I've read in a primary research article on gene drive. The Discussion, for the most part is not a Discussion but a (good) recapitulation of the results. The same cannot be said for the Results though - in parts these are impossible to follow and I say that as someone familiar with gene drive. They are over-long, non-selective and lack explainers. A crucial element that is missing, very early on in the article, is an overview of the approach accompanied by a schematic. I was two-thirds through the article before I realised the goal was to release a goldilocks element - just the right amount of power to increase in frequency for a bit but then ultimately falls away after its magic is done, thanks to this auto-excision and resistance. (I had initially thought the idea was to show that SSA can happen in a test strain, then release an element that could 'chase' a previous gene drive that had been stacked with pre-loaded direct repeats as a potential recall).
I use the term Goldilocks deliberately because it is precious and prevaricated on quite a few things being 'just right' and so does not escape the uncertainty (just as other gene drives do not) in predicting how this will actually behave. I think this element deserves more expansion in the Discussion.

Rather than list all my comments with regards to structure and clarity here I have included an annotated version - it deliberately has some of my errors of understanding left in as I think these will usefully highlight where some problems can arise and I hope the authors will find it useful.

*We thank the reviewer for their extensive review and annotation of the manuscript. We have tried to incorporate as many of the reviewer’s suggestions as possible. In some cases, it was not always possible to clearly interpret the reviewer’s comments, and in these instances, we attempted to revise based on our best perceptions of the reviewer’s intent. However, we may not always have succeeded in adequately addressing unclear text or other elements of the manuscript. For this we apologize, and would be willing to try again.*

**Have all data underlying the figures and results presented in the manuscript been provided?**
Large-scale datasets should be made available via a public repository as described in the *PLOS Genetics* [data availability policy](https://urldefense.com/v3/__http:/journals.plos.org/plosgenetics/s/data-availability__;!!KwNVnqRv!F5-yyXLXfGG9KYzEJpFvQMzkBsnjqfUZsAS2AHRsg34ybQqFJ4CddJcdYee_GFT5z3Pvkv_W8HBmwHg$), and numerical data that underlies graphs or summary statistics should be provided in spreadsheet form as supporting information.

Reviewer #1: Yes

Reviewer #2: Yes

Reviewer #3: None

Reviewer #4: Yes

Reviewer #5: Yes

PLOS authors have the option to publish the peer review history of their article ([what does this mean?](https://urldefense.com/v3/__https:/journals.plos.org/plosgenetics/s/editorial-and-peer-review-process*loc-peer-review-history__;Iw!!KwNVnqRv!F5-yyXLXfGG9KYzEJpFvQMzkBsnjqfUZsAS2AHRsg34ybQqFJ4CddJcdYee_GFT5z3Pvkv_WFOBHwmg$)). If published, this will include your full peer review and any attached files.


If you choose “no”, your identity will remain anonymous but your review may still be made public.


**Do you want your identity to be public for this peer review?** For information about this choice, including consent withdrawal, please see our [Privacy Policy](https://urldefense.com/v3/__https:/www.plos.org/privacy-policy__;!!KwNVnqRv!F5-yyXLXfGG9KYzEJpFvQMzkBsnjqfUZsAS2AHRsg34ybQqFJ4CddJcdYee_GFT5z3Pvkv_WCWumZNw$).

Reviewer #1: No

Reviewer #2: No

Reviewer #3: No

Reviewer #4: No

Reviewer #5: No
