# Supplementary material for: Repeat mediated excision of gene drive elements for restoring wild-type populations
Source: PLoS Genet. 2024 Nov 7;20(11):e1011450. doi: 10.1371/journal.pgen.1011450 (PMC11584131; doi:10.1371/journal.pgen.1011450)
Supplement: Attachment — Submitted filename: Review of revised Chennuri et al.(1).docx [file pgen.1011450.s014.docx]

The revised manuscript by Chennuri et al. is more clearly written and discusses several issues regarding data interpretation that are improvements in the presentation of their results. Nonetheless, I still have questions regarding some important aspects of the experimental results which weaken the impact of the manuscript in its current form.

In reviewing the authors responses to my prior comments, it seems that our various intended points may have crossed in certain respects, which could in part be due to my not having been sufficiently clear. I will try to restate my key concerns here again, hopefully, more effectively.

1) I am still quite puzzled by the low levels of SSA-mediated drive deletion reported do not increase upon induction of the I-SceI gene either in the yReMet-cis configuration. The authors suggest that this might be due to basal levels of nuclease activity already being saturating such that further induction yields no higher rates of SSA. This is odd though given how low those rates are (also those rates are even lower for the 500 bp repeat than for the 250 bp version, which also does not make sense) and is very different than the rates they observe for their transacting system for inducing I-SceI using the GAL4/UAS system, which does steadily increase in efficiency as the repeat length gets longer (Fig. 1B). Similarly, efficient SSA-mediated deletion rates were reported in the pre-print by Aguilar and colleagues (reference included in revised list of recommended SSA papers to cite below). The very marginal activity of the yReMet-cis element makes it hard to understand the results for the yReMEDE, which as I understand it relies on the same genetic cassette for I-SceI expression and yet according to their analysis in Fig. 2C seems to generate a substantially higher rate of SSA events? It is also noteworthy that in the paper by Chae et al, 2022 by the same authors examining SSA in mosquitoes using a similar scheme, SSA rates using the nos:I-SceI system were also quite low. If one supposed that in the current study, I-SceI expression was no different in yReMet-cis and yReMEDE, then could Cas9 expression by the primary mechanism behind the generation of the excess of y+ in yReMEDE/+ heterozygotes?

One experiment that should shed light on this question would be to measure the rate of generating y+ progeny when the yReMEDE drive element is homozygous as compared to heterozygous in females since Cas9 would not be doing any cutting when homozygous. Similarly, analyzing the basal level of generating y+ progeny from yReMEDE males mated to an attached-X stock should be informative? Would they see comparable levels of SSA as in Fig. 2C (yReMet/WT heterozygotes)? Comparing yReMEDE males (with no paternal Cas9 deposition) to homozyous yReMEDE females (where maternal Cas9 deposition does take place) would provide an indication for whether Cas9 in the absence of target cleavage could somehow increase SSA (unlikely, but worth testing to be sure). Perhaps the authors have already done these experiments?

The most direct approach would be to generate a version of yReMEDE either without I-SceI (to unambiguously test for its requirement in SSA) or place I-SceI expression under GAL4 control as in the yReMet trans element. Then the authors could cross this element to the nos-GAL4 driver and see whether it undergoes the same efficient SSA deletion as seen with yReMet. Another informative experiment would be to place the yReMEDE-UAS element in trans to the yReMet-cis element in the presence of nos-GAL4. This experiment should be possible to do technically given the very low level of SSA observed with the yReMet-cis element by first combining yReMet-cis with nos-GAL4 and then cross their progeny to yReMEDE-UAS. By differentially marking the yReMet-cis and yReMEDE chromosomes (e.g., with either a viable achaete-scute allele or white-) the authors should be able to compare the relative frequency of SSA deletion in these two elements, which would resolve whether the low frequency for the yReMet-cis element has to do with its internal structure or its ability to induce I-SceI expression.

It also should be revealing to simply redo the experiment in Fig. 2B using differentially marked chromosomes carrying the yReMEDE versus WT alleles (again either the very closely linked achaete-scute or slightly more distant white- locus could serve as such markers). This type of analysis would additionally shed light on the question raised in point 3 below regarding the possibility that the yReMEDE system combined with Cas9 drive could induce chromosome damage or loss. Furthermore, this analysis would allow the authors to determine whether the dual action of Cas9 and I-SceI can increase recombination between the two chromosome homologs, which could offer important mechanistic clues to resolving the issue in point 2.

2) Curiously, the reported SSA-mediated deletion events often do not carry the expected cis-marked allelic variant (this contrasts with what they observe for the yReMet element either in cis- or trans configurations). The basis for this finding is not resolved? The authors discuss a variety of possible such events might be occurring (including a need for Cas9 cleavage), but they do not perform experiments to analyze the basis for this phenomenon, which most likely holds a key to how such dual nuclease systems work.

Including additional polymorphisms at different distances and both sides of the repeats (up to 10 kb away) also would be an informative way to help figure this process out. Similarly, as mentioned above, they could use a tightly linked visible marker (e.g., viable alleles of the achaete-scute complex, which is the gene immediately next to yellow) to help distinguish local gene-conversion from cross-over events. These types of studies should provide the type of data to conclusively determine the origin and requirements for various aspects of the drive process

Alternatively, since trans-acting GAL4/UAS system generate decent levels of SSA-mediated deletion, the authors might consider constructing a similar split version the yReMEDE system (with I-SceI expression under GAL4 control as suggested above) to see if they obtain different results than with the cis-acting system. In particular, what would be the fraction of yellow+ alleles carrying the TCG allele when the yReMEDE allele was homozygous versus in trans to a wt allele or a cleavage resistant allele (different from TCG)? In these experiments, it would be important to used well marked chromosomes distinguishing the one carrying the drive cassette and the target allele. Another possible approach would be to generate a females with a trans configuration of yReMet ac+ w-/yReMEDE ac- w+; nos-GAL4 to determine the relative efficiency of SSA in these two vector contexts. Such a dual marked system would also permit an assessment of whether induction of SSA events lead to greater rates of recombination between achaete and white.

3) The question of whether the dual nuclease system in yReMEDE results in genome damage while the drive casette is copying to a wt target, which could result in a potential reduction in the fraction of chromosomes carrying the drive construct, remains unresolved (the authors seem to address my previous question by referring to transmission through males, which is not the question I had in mind). The phenomenon, if it occurs, would be evident in progeny of females carrying one drive allele and a differentially marked WT allele (carrying an adjacent visible achaete-scute allele). The question would be whether the fraction of male progeny carrying the drive chromosome equaled that carrying the WT allele? If not, then some of the alleles present in females could be homozygous lethals (or hemizygous lethals in males). Such alleles could be recovered over balancers if this result was observed and further analyzed to assess their origin and nature. This type of analysis might also benefit from using a split yReMEDE system as suggested above since there would be no prior I-SceI cleavage prior to making the cross.

4) The authors argue that they cannot use the current system to adequately test their system in multigenerational cage trials primarily because of the strong competitive advantage that yellow+ males have relative to less fit yellow- males. There are several relatively simple solutions to this issue, which after all, is crucially central to testing their concept. One simple solution is to associate both test alleles with a second well separated yellow mutant allele (e.g., near 5' end of the gene). This could easily be accomplished by crossing the drive element to chromosome carrying the 5' y- allele and recovering progeny in which the element had copied to that chromosome. Then all competing individuals would be competitively equal. Alternatively, they could add recoded 3' yellow sequences to drive element to endow it with a yellow+ phenotype.

Based on modeling results, the authors suggest that their system even with its relatively low level of basal SSA-mediated cassette deletion could lead to eventual population replacement with a cassette-deleted allele. While this may true, there are several issues that still need to be considered including how would they maintain such an unstable strain in the lab. It obviously could not be kept long-term as a pure homozygous stock. The authors should also compare such scenarios to much simpler drive systems such as those designed to accomplish population suppression. Many of these systems generate low levels of functional NHEJ alleles. Such functional cleavage resistant target alleles also can take over populations as has been demonstrate experimentally by several groups. One could even use a very efficient suppression drive such as the Doublesex drive from the Crisanti group and then introduce a low fraction of individuals carrying a recoded functional target site as many commentators have suggested as an alternative which perhaps might generate less stochastic outcomes. What are the advantages that the authors see would SSA-mediated cassette deletion over just letting the drive cassette get eliminated by competition with a wild-type allele.

For all the reasons summarized above, hopefully better articulated this round, I suggest that the authors consider doing a few additional incisive experiments to resolve what I believe are key unresolved questions. This additional analysis, much of which could be done with existing stocks, would strengthen the study significantly and provide a better data set for evaluating the potential virtues and limitations of their very worthy conceptual idea.

5) Discussion and citations: There are several points deserving more consideration in the discussion section. Most notably, the authors should include a thorough comparison of the results from the current study in Drosophila with the extensive experimental findings already published by the same group in *Aedes aegypti* mosquitoes (Chae et al., 2022). While the results in both systems are similar in some respects (low levels of SSA), they also are different in others (for example in mosquitoes NHEJ alleles at the I-SceI site cause loss of DsRed function but not adjacent GFP expression thus distinguishing NHEJ from SSA alleles, which is not considered in the current study). The way the authors currently cite that prior work makes it seem as though the Chae study is only a computational paper. It would be helpful to synthesize all these results to provide readers with a sense of how ReMEDE technology might progress in the future and what issues need to be considered for developing the technology to a field applicable state.

Another odd result in this study mentioned by the authors is the apparent instability of the I-SceI target site. Since that sequence contains a 4 bp directly repeated sequence including 3Gs is this event potentially being mediated by a low frequency of SSA or perhaps MMEJ. If this this a problem intrinsic to I-SceI, which would render cassettes carrying such target site deletions refractory to I-SceI mediated SSA, might some other orthogonal nuclease be used instead?

Finally, regarding my prior list of suggested reference the authors should cite to ensure that adequate credit is assigned to prior work on exploiting SSA in Drosophila, I note that the authors are indeed correct in pointing out that I had selected the wrong citations for them to consider in this regard. My apologies on that mistake. Perhaps the authors could cite the three references indicated below, all of which have SSA in their titles. These references could all be cited in the introduction as antecedent work, and in the case of the latter two papers, also could be cited with regard to testing the dependence on the length of the flanking repeats for efficiency of SSA-mediated deletion.

**Relevant SSA References:**

Preston, C. R., Engels, W., & Flores, C. (2002). Efficient Repair of DNA Breaks in Drosophila:

Evidence for Single-Strand Annealing and Competition With Other Repair Pathways.

Genetics, 161(2), 711-720. doi: [10.1093/genetics/161.2.711](https://doi.org/10.1093%2Fgenetics%2F161.2.711)

Dewey EB, Korda Holsclaw J, Saghaey K, Wittmer ME, Sekelsky J. The effect of repeat length on Marcal1-dependent single-strand annealing in Drosophila. Genetics. 2023 Jan 12;223(1):iyac164. doi: 10.1093/genetics/iyac164.

Gustavo Aguilar, Milena Bauer, M. Alessandra Vigano, Sophie T. Schnider, Lukas Brügger, Carlos Jiménez-Jiménez, Isabel Guerrero, Markus Affolter.

*In vivo* seamless genetic engineering via CRISPR-triggered single-strand annealing

bioRxiv 2022.06.17.496589; doi: https://doi.org/10.1101/2022.06.17.496589
